# Hydrazine Photorelease from Ru(II) Chugaev-Type Dicarbene Complexes

**DOI:** 10.1021/jacs.6c13259

**Published:** 2026-09-09

**Authors:** Subhradip Kundu, Xuewen Zhang, Conor J. Doran, Sian E. Dillon, Eugene In, Ryan D. Ribson, Céline Besnard, Laure Guénée, Marcus Jones, Dimosthenis Sokaras, Elisa Biasin, Christopher B. Larsen

**Affiliations:** † Department of Inorganic and Analytical Chemistry, University of Geneva, CH-1211 Geneva, Switzerland; ‡ School of Chemical Sciences, University of Auckland, Auckland 1010, New Zealand; § SSRL, SLAC National Accelerator Laboratory, Menlo Park, California 94025, United States; ∥ Laboratory of Crystallography, University of Geneva, 24 Quai Ernest Ansermet, 1211 Geneva, Switzerland; ⊥ School of Science, 1410 Auckland University of Technology, Auckland 1010, New Zealand; # MacDiarmid Institute for Advanced Materials and Nanotechnology, Auckland 1010, New Zealand; ∇ Te Whai Ao, Dodd-Walls Centre for Photonic and Quantum Technologies, Dunedin 9016, New Zealand; ○ 6865 Pacific Northwest National Laboratory, Richland, Washington 99352, United States

## Abstract

Ruthenium­(II) polypyridyl complexes exhibit rich excited state reactivity from both photogenerated ^3^MLCT and higher energy thermally populated ligand-field (LF) excited states. In order to maximize the utility of the different reactivity of these two states, the energy gap between them must be tuned without sacrificing the favorable optical and photophysical properties of the complex. In probing design strategies aimed at enhancing the photostability of RuII complexes for nondissociative photochemical applications, we herein explore the use of Chugaev-type dicarbene ligands as strong σ-donors. Using a complementary combination of X-ray crystallography, electrochemistry, and optical and X-ray spectroscopies, we demonstrate how Chugaev-type dicarbene ligands destabilize LF states of photoactive Ru^II^ complexes without sacrificing the favorable optical and photophysical properties of the complex, and compare with π-accepting methylisocyanide ligands. Despite these design strategies aimed at suppressing dissociative excited-state ligand-field pathways, we further report an unexpected dechelative photoelimination of hydrazine from the Ru^II^ Chugaev-type dicarbene complexes that occurs directly from the ^3^MLCT excited state with quantum yields in the order of ca. 1–5%, and is sensitive to peripheral substitution of chromophoric bipyridine ligands. These findings contribute to growing understanding of molecular design principles for photoactive Ru^II^ complexes targeted at either dissociative/nondissociative photochemical applications and introduce a new potential scaffold and photorelease mechanism for photoactivated chemotherapy.

## Graphical abstract

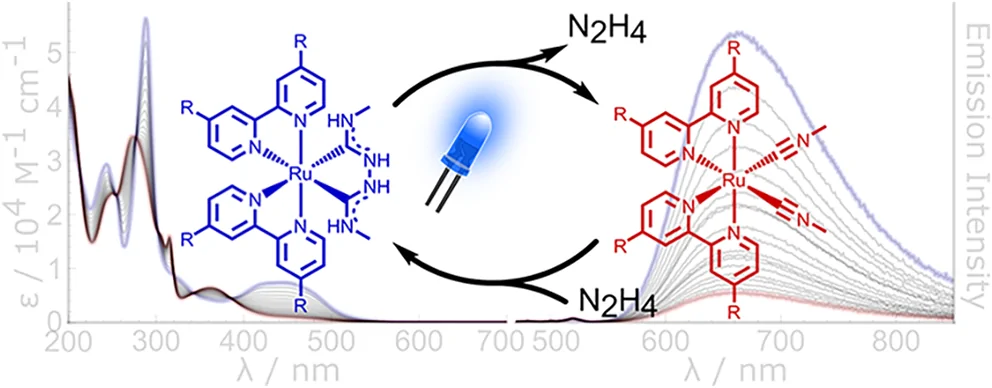

## Introduction

Ruthenium­(II) polypyridyl complexes have been extensively studied for over half a century due to their remarkable photophysical and photochemical properties,^1–3^ which originate from highly tunable photoluminescent triplet metal-to-ligand charge-transfer (^3^MLCT) excited states.^4,5^ The intra- and bimolecular electron- and energy-transfer reactivity of these emissive ^3^MLCT states has led to the widespread utilization of Ru^II^ polypyridyl complexes in various photochemical, photophysical and photobiological applications.^6–15^

In addition to the electron- and energy-transfer reactivity of the ^3^MLCT state, Ru^II^ polypyridyl complexes can also undergo ligand photosubstitution via thermal population of higher energy, dissociative ligand-field (LF) states.^16–19^ Although this photolability is detrimental to the above-mentioned applications that require photosensitizer integrity, it does allow the release of ligated drug molecules with high spatiotemporal control, which has led to the application of such complexes in photoactivated chemotherapy (PACT).^9,20^

Ru^II^ polypyridyl complexes therefore have a wide range of accessible reactivity, but in order to tailor complexes for either dissociative or nondissociative applications, methods to reliably control the energy gap (ΔE) between the ^3^MLCT and LF states, without sacrificing the favorable optical/photophysical properties of the complex (namely, visible absorption leading to high energy, long-lived excited states) are yet to be realized. There has been significant success in lessening ΔE for Ru^II^ PACT complexes by incorporating steric hindrance around the metal and by tuning the electronic impact of chromophoric ligands.^9^ On the other hand, increasing ΔE to access more photostable photosensitizers has been less well studied. There has been substantial work on destabilizing LF states in related Fe^II^ complexes,^21–23^ with the main approach focusing on increasing the ligand-field strength through incorporation of strongly σ-donating coordinating moieties such as *N*-heterocyclic carbenes (NHCs), mesoionic carbenes (MICs) and carbanions (cyclometalating ligands) into either bidentate or tridentate ligand scaffolds. More recent studies have also highlighted the role of nephelauxetism in tuning LF state energies.^24,25^

Although less mature than the studies into destabilizing LF states in Fe^II^ complexes, analogous studies on isoelectronic Ru^II^ complexes have generally followed the same approach, introducing strongly σ-donating NHC, MIC and cyclometalating coordinating motifs. A different approach of utilizing π-accepting isocyanide and isocyanoborato ligands has also been documented.^26–28^ Cyclometalation typically results in significant bathochromic shifts of MLCT absorption and emission features relative to [Ru­(bpy)_3_]^2+^ (bpy = 2,2′-bipyridine) and substantially reduced excited state lifetimes as a consequence of the Energy Gap Law.^29–34^ In contrast, incorporation of NHC and MIC coordination motifs into tridentate ligands has yielded complexes with optical properties reminiscent of [Ru­(bpy)_3_]^2+^, including ^3^MLCT emission quantum yields and excited state lifetimes on the same order of magnitude of, and in some cases exceeding, those of [Ru­(bpy)_3_]^2+^.^29,35–40^ This was attributed to the strongly σ-donating ligands increasing ΔE relative to [Ru­(terpy)_2_]^2+^ (terpy = 2,2′;6′,2″-terpyridine), which suffers from substantially stabilized LF states relative to [Ru­(bpy)_3_]^2+^ arising from unfavorable ligand bite angles.^38^

Recently, McCusker and colleagues reported that ancillary “Chugaev-type” dicarbene ligands can destabilize LF states in Fe^II^ polypyridyl complexes, while the MLCT optical properties are essentially unaffected.^41^ The photophysics of transition metal complexes bearing Chugaev-type dicarbene and related acyclic diaminocarbene ligands has largely been limited to luminescent Ir^III^, Pt^II^ and Au^III^ complexes.^42^ We were therefore interested if Chugaev-type dicarbene ligands could be used to destabilize LF states in Ru^II^ complexes and thus enhance their photophysical properties for nondissociative photochemical applications.

In this work, we report two novel Ru^II^ Chugaev-type dicarbene complexes, [Ru­(bpy)_2_(C_4_H_10_N_4_)]­(PF_6_)_2_ (bpy = 2,2′-bipyridine) and [Ru­(dtbbpy)_2_(C_4_H_10_N_4_)]­(PF_6_)_2_ (dtbbpy = 4,4′-di-*tert*-butyl-2,2′-bipyridine), and their electronic structure, photophysical and photochemical properties. Using a combination of X-ray crystallography, electrochemistry, optical spectroscopy and X-ray spectroscopy, we show how dissociative ligand-field states are destabilized in the Chugaev-type dicarbene complexes without sacrificing the favorable optical and photophysical properties attributed to Ru^II^ polypyridyl complexes. Additionally, using a combination of electronic absorption, emission and ^1^H NMR photolysis experiments, we report the unexpected, efficient photoinduced dechelative elimination of hydrazine from the Chugaev-type dicarbene complexes directly from the ^3^MLCT excited state upon visible light irradiation.

## Results and Discussion

### Synthesis and Characterization

The two Chugaev-type dicarbene complexes [Ru­(bpy)_2_(C_4_H_10_N_4_)]­(PF_6_)_2_ and [Ru­(dtbbpy)_2_(C_4_H_10_N_4_)]­(PF_6_)_2_ were prepared in two steps (Scheme 1) from their corresponding *cis*-dicyanido complexes, [Ru­(bpy)_2_(CN)_2_] and [Ru­(dtbbpy)_2_(CN)_2_], respectively. The cyanido ligands were first methylated using dimethyl sulfate, followed by counterion exchange with KPF_6_ to afford the *cis*-di­(methylisocyanide) complexes, [Ru­(bpy)_2_(MeNC)_2_]­(PF_6_)_2_ and [Ru­(dtbbpy)_2_(MeNC)_2_]­(PF_6_)_2_. Finally, chelative addition of hydrazine across the coordinated methylisocyanide ligands yields the target Chugaev-type dicarbene complexes. The identity and purity of the *cis*-di­(methylisocyanide) and Chugaev-type dicarbene complexes are confirmed by ^1^H and ^13^C NMR spectroscopy, HRMS, elemental analysis and X-ray crystallography.

**Scheme 1:**
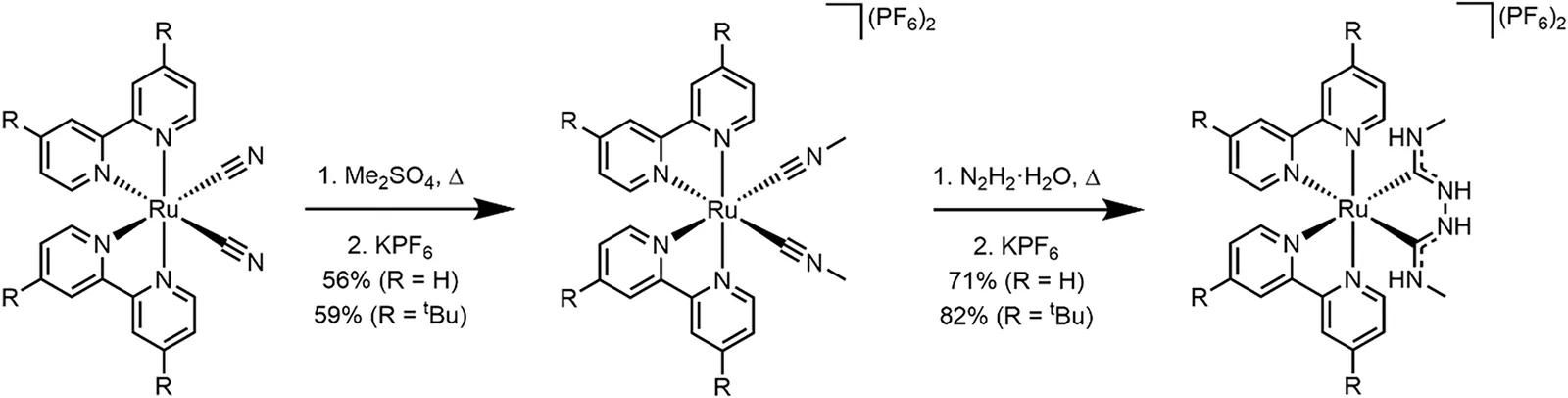
Synthesis of Target Ru^II^ Chugaev-Type Carbene Complexes

Single crystals were obtained for both *cis*-di­(methylisocyanide) and Chugaev-type dicarbene complexes (Tables S1–S4). Each complex (Figure 1) crystallized as the racemate, with both enantiomers present in the unit cell (Figures S1–S4).

**Figure 1:**
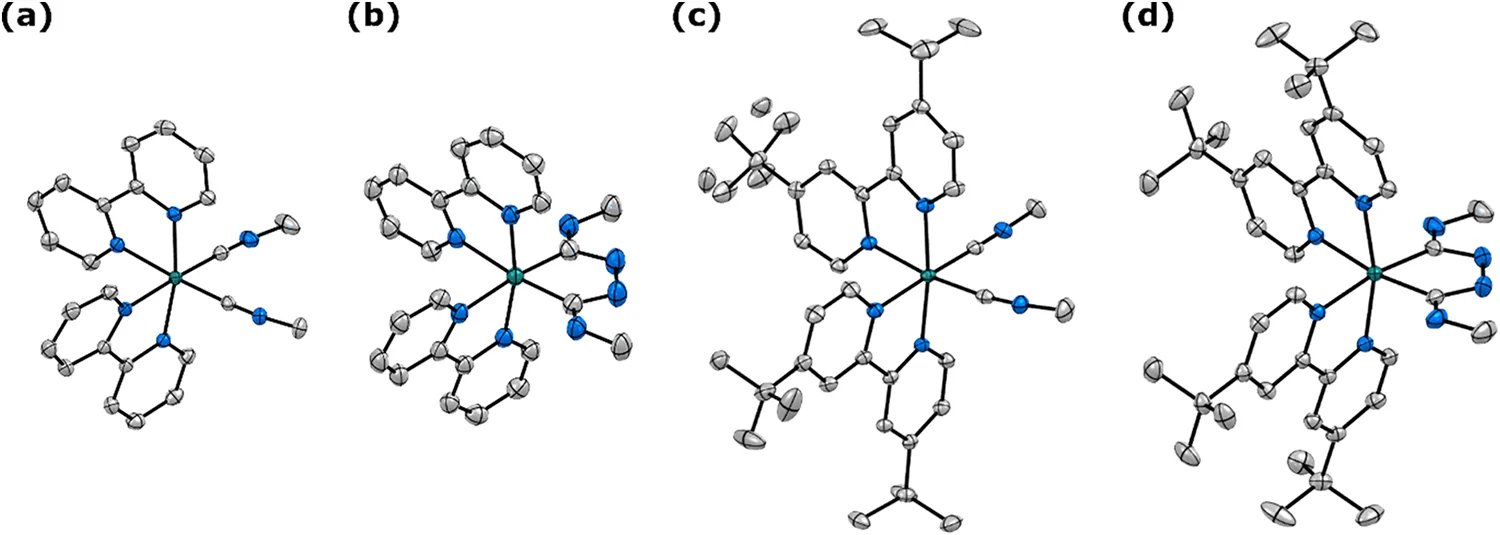
Molecular structures of (a) [Ru­(bpy)_2_(MeNC)_2_]­(PF_6_)_2_, (b) [Ru­(bpy)_2_(C_4_H_10_N_4_)]­(PF_6_)_2_, (c) [Ru­(dtbbpy)_2_(MeNC)_2_]­(PF_6_)_2_, and (d) [Ru­(dtbbpy)_2_(C_4_H_10_N_4_)]­(PF_6_)_2_ obtained from single crystal X-ray diffraction. Ellipsoids are depicted at the 50% probability level. Hydrogen atoms, PF_6_^–^ counterions and cocrystallized solvent molecules are omitted for clarity.

In the X-ray crystal structures, both the *cis*-di­(methylisocyanide) and Chugaev-type dicarbene complexes exhibit shorter Ru–C than Ru–N distances (Table 1), indicative of stronger metal–ligand interactions with the nonchromophoric C-donor ligands than the chromophoric N-donor ligands. The Ru–C bond lengths are shorter for the *cis*-di­(methylisocyanide) complexes than for the Chugaev-type dicarbene complexes, indicating greater covalency between the Ru and nonchromophoric ligands in the former. This observation is consistent with the known π-acceptor character of isocyanide ligands, in which metal–ligand covalency is enhanced through π-back-donation. On the other hand, carbenes are typically strong σ-donor ligands, which also results in enhanced metal–ligand covalency relative to the chromophoric N-donor ligands, albeit to a lesser extent than π-acceptor ligands.

**Table 1:** Selected Bond Lengths and Angles from X-Ray Crystal Structures of Investigated Complexes

complex	average Ru–C bond length/Å	average Ru–N_eq_ bond length/Å	average Ru–N_ax_ bond length/Å	C–Ru–C angle/°
[Ru(bpy)_2_(MeNC)_2_](PF_6_)_2_	1.954(2)	2.107(3)	2.071(3)	87.60(9)
[Ru(bpy)_2_(C_4_H_10_N_4_)](PF_6_)_2_	2.000(3)	2.134(3)	2.061(3)	79.4(2)
[Ru(dtbbpy)_2_(MeNC)_2_](PF_6_)_2_	1.954(2)	2.109(2)	2.077(2)	87.17(8)
[Ru(dtbbpy)_2_(C_4_H_10_N_4_)](PF_6_)_2_	2.002(3)	2.132(3)	2.066(3)	79.9(2)

The Ru–N bond lengths of both *cis*-di­(methylisocyanide) and Chugaev-type dicarbene complexes are larger than those of [Ru­(bpy)_3_]^2+^ (2.056 Å),^43^ with only negligible differences between the bpy- and dtbbpy-based complexes. The axial Ru–N bond lengths (oriented *cis* to nonchromophoric ligands) are substantially less perturbed than the equatorial Ru–N bond lengths relative to those of [Ru­(bpy)_3_]^2+^, consistent with a *trans* influence. Despite shorter Ru–C bonds in the *cis*-di­(methylisocyanide) complexes, a stronger *trans* influence is observed for the Chugaev-type dicarbene complexes, consistent with its stronger σ-donor strength.^44,45^

### Electrochemical and Optical Characterization

In MLCT excited states of Ru^II^ polypyridyl complexes, the Ru center is formally oxidized to its +3 oxidation state and the polypyridyl ligands are reduced to a radical anion. These two redox processes can be independently probed using electrochemical measurements to understand how nonchromophoric ligands energetically perturb relevant frontier molecular orbitals, and the difference in energy between metal oxidation and ligand reduction correlated to MLCT absorption/emission energies.^41,46–49^ DFT calculations (Figure S44) indicate that for all complexes investigated, the HOMO is nominally a Ru t_2g_ orbital and the first several unoccupied orbitals are based on the polypyridyl ligands.

The electrochemical properties of the investigated complexes are summarized in Table 2, with cyclic and differential pulse voltammograms presented in Figure S5. In Table 2, we report only the first ligand reduction, as subsequent reductions are increasingly destabilized by Coulombic repulsion, and thus do not accurately reflect the energies of frontier molecular orbitals relevant to the excited state properties of the investigated complexes.

**Table 2:** Electrochemical and Optical Properties of the Investigated Complexes in Acetonitrile

complex	E^0^ (L^0/•‑^)/ V vs SCE^a^	E^0^ (Ru^III/II^)/ V vs SCE^a^	ΔE^0^/eV^b^	λ_abs_ (ε)/ nm(10^3^ M^–1^ cm^–1^)	*E*_abs_/eV
[Ru(bpy)_3_](PF_6_)_2_	–1.35	1.27	2.62	455 (13.4)	2.72
[Ru(bpy)_2_(MeNC)_2_](PF_6_)_2_	–1.27	1.92	3.19	371 (6.42)	3.34
[Ru(bpy)_2_(C_4_H_10_N_4_)](PF_6_)_2_	–1.65	1.03^c^	2.68	466 (6.58)	2.66
[Ru(dtbbpy)_3_](PF_6_)_2_	–1.37^c^	1.12	2.49	463 (12.7)	2.68
[Ru(dtbbpy)_2_(MeNC)_2_](PF_6_)_2_	–1.39	1.79	3.18	372 (4.70)	3.33
[Ru(dtbbpy)_2_(C_4_H_10_N_4_)](PF_6_)_2_	–1.72	0.96	2.68	466 (6.64)	2.67

a Recorded as 1 mM solutions with 0.1 M [Bu_4_N]­PF_6_, 100 mV s^–1^ scan rate.

b E^0^ (Ru^III/II^) - E^0^ (L^0/•‑^).

c Irreversible.

Comparing the *cis*-di­(methylisocyanide) complexes to their corresponding homoleptic analogues, [Ru­(bpy)_3_]­(PF_6_)_2_ and [Ru­(dtbbpy)_3_]­(PF_6_)_2_, respectively, a large anodic shift is observed for the Ru^III/II^ redox couple (+0.65 V for the bpy ligand systems, + 0.67 V for the dtbbpy systems). This indicates a substantial withdrawal of electron density from the metal center through π-backbonding with the strongly π-accepting isocyanide ligands, stabilizing the nominally Ru t_2g_ orbital set, and is consistent with the short Ru–C bond lengths observed crystallographically for the *cis*-di­(methylisocyanide) complexes. The first bpy/dtbbpy reduction is relatively unchanged between the *cis*-di­(methylisocyanide) complexes and their homoleptic analogues, resulting in a wider metal-based HOMO – ligand-based LUMO gap in the *cis*-di­(methylisocyanide) complexes, and correlates with a hypsochromic shift of the characteristic MLCT absorption from 455 to 371 nm (0.62 eV) for the bpy ligand systems, and 463 to 372 nm (0.66 eV) for the dtbbpy systems in UV–vis electronic absorption spectra (*vide infra*). DFT calculations support these assignments, predicting a stabilization of the nominally Ru t_2g_ orbital set (Figure S44), arising from mixing with isocyanide π* orbitals (Table S16), and negligible stabilization of the bpy-based unoccupied orbitals. TD-DFT calculations further accurately predict the hypsochromic shift of the MLCT absorption band (Figure S45).

In contrast, comparing the Chugaev-type dicarbene complexes to their corresponding homoleptic analogues, a cathodic shift is observed for both the Ru^III/II^ redox couple (−0.24 and −0.16 V for the bpy/dtbbpy ligand systems, respectively) and the first chromophoric ligand reduction (−0.30 and −0.35 V for the bpy/dtbbpy ligand systems, respectively). The cathodic shift of the Ru^III/II^ redox couple arises from increased electron density at the metal center, consistent with the strong σ-donor character of the Chugaev-type dicarbene ligand and prior reports of Ru^II^ NHC/MIC complexes.^36,37,39,40^

McCusker and co-workers postulated that a similar cathodic shift of the first ligand reduction for the related Fe^II^ complex, [Fe­(phen)_2_(C_4_H_10_N_4_)]­(BF_4_)_2_ (phen = 1,10-phenanthroline), relative to [Fe­(phen)_3_]­(BF_4_)_2_, could arise from increased π-backbonding from the Fe to phen ligand due to the enhanced electron density at the metal center.^41^ However, this enhanced backbonding would be expected to result in contracted Ru–N bonds for the Chugaev-type dicarbene complexes relative to their homoleptic analogues, and here we report the opposite. In the X-ray crystal structures of the Chugaev-type dicarbene complexes (*vide supra*), all Ru–N bonds are elongated compared to those of [Ru­(bpy)_3_]^2+^, with the equatorial Ru–N bond lengths substantially more perturbed than the axial. This results in a weaker interaction between the chromophoric ligands and the metal, minimizing the stabilization of chromophoric ligand π* orbitals associated with coordination to a π-acid.

Given that both metal oxidation and ligand reduction cathodically shift from the homoleptic to Chugaev-type dicarbene complexes by similar extents, the MLCT absorption wavelength is relatively unchanged relative to the corresponding homoleptic complexes, albeit ∼33% weaker, consistent with possessing one less chromophoric ligand, and is further predicted by TD-DFT calculations (Figure S45). DFT calculations (Figure S44) indicate little involvement of the Chugaev-type dicarbene in the Ru-based HOMO and HOMO–1 orbitals, with a small extent of π-backbonding evident in the HOMO–2 orbital, indicating that the Chugaev-type dicarbene does not alter the nature of the MLCT electronic transitions. The HOMO–3 orbital is carbene-based and close in energy to the nominally Ru t_2g_ orbital set, but according to TD-DFT calculations (Table S19), is not involved in any electronic transitions at wavelengths >300 nm.

### Emission Spectroscopy

Electronic absorption and emission spectra of all investigated complexes are presented in Figure 2, and the emission properties summarized in Table 3.

**Figure 2:**
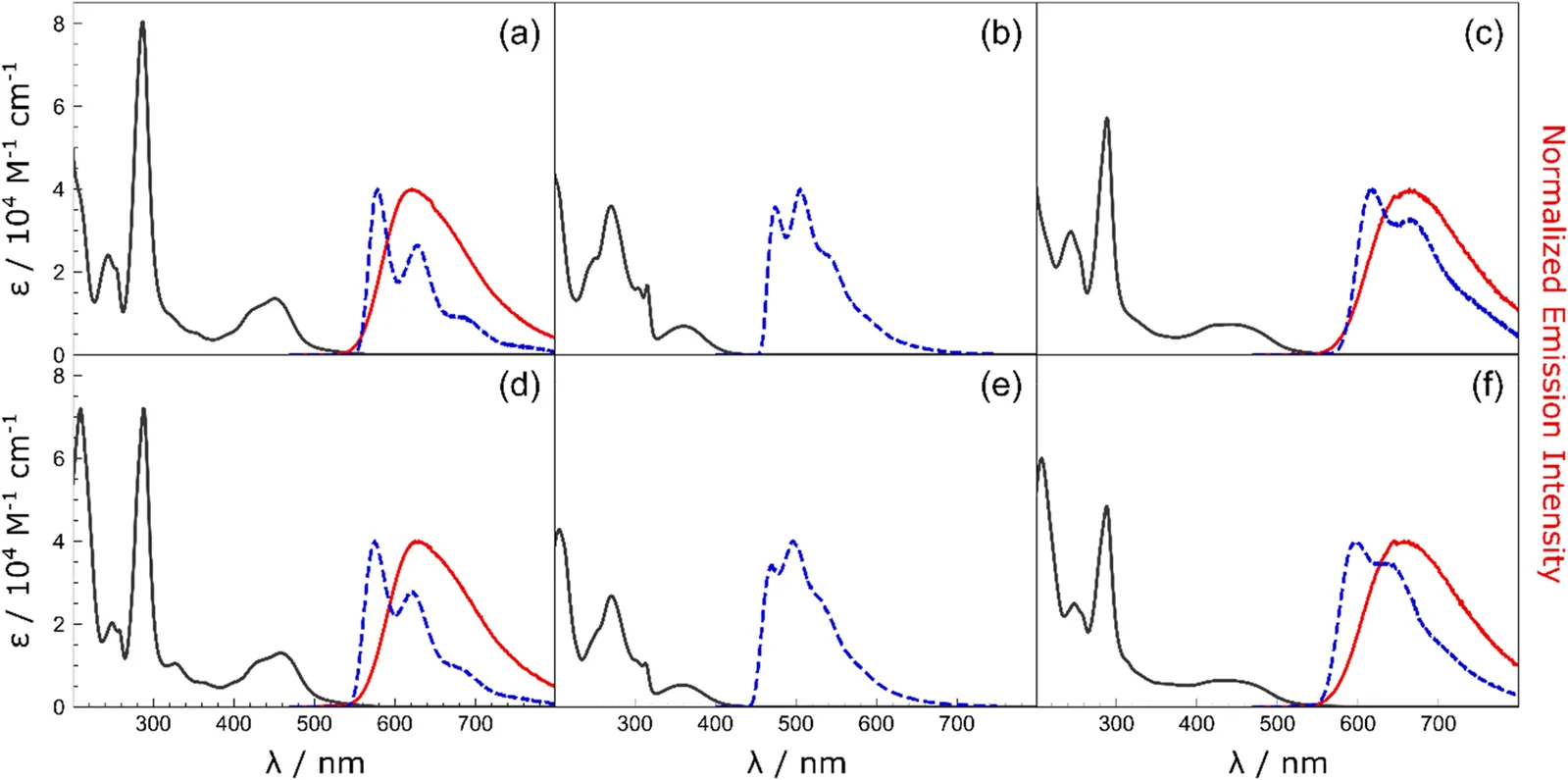
UV–vis electronic absorption spectra (black traces), normalized emission spectra recorded in deaerated acetonitrile at room temperature (red traces, λ_ex_ = 450 nm) and as frozen butyronitrile glasses at 77 K (blue dashed traces, λ_ex_ = 450 nm for homoleptic and Chugaev-type dicarbene complexes, λ_ex_ = 350 nm for *cis*-di­(methylisocyanide) complexes) of (a) [Ru­(bpy)_3_]­(PF_6_)_2_, (b) [Ru­(bpy)_2_(MeNC)_2_]­(PF_6_)_2_, (c) [Ru­(bpy)_2_(C_4_H_10_N_4_)]­(PF_6_)_2_, (d) [Ru­(dtbbpy)_3_]­(PF_6_)_2_, (e) [Ru­(dtbbpy)_2_(MeNC)_2_]­(PF_6_)_2_, and (f) [Ru­(dtbbpy)_2_(C_4_H_10_N_4_)]­(PF_6_)_2_.

**Table 3:** Summary of Room Temperature Emission Properties for Investigated Complexes in Acetonitrile^b^

complex	λ_em_/nm	φ	E_0_/eV^a^	τ/ns	*k*_r_/10^5^ s^–1^	*k*_nr_/10^6^ s^–1^	*k*_nr_(calc)/10^6^ s^–1^ ^b^
[Ru(bpy)_3_](PF_6_)_2_	613	0.095^c^	2.02	855^d^	1.11	1.16	1.16
[Ru(bpy)_2_(C_4_H_10_N_4_)](PF_6_)_2_	641	0.021	1.93	227	0.93	4.40	4.50
[Ru(dtbbpy)_3_](PF_6_)_2_	617	0.091^e^	2.00	730^f^	1.25	1.36	0.95
[Ru(dtbbpy)_2_(C_4_H_10_N_4_)](PF_6_)_2_	636	0.033	1.95	296	1.11	3.37	3.89

a Obtained from Franck–Condon band shape fitting (see Figure S19 and Table S6).

b Obtained from Energy-Gap Law analysis (see SI, p. S26–S35).

c From ref 53.

d From references 6,54 .

e From ref 55.

f From ref 56.

In stark contrast to their homoleptic analogues, the *cis*-di­(methylisocyanide) complexes do not emit in deaerated acetonitrile at room temperature upon excitation into their MLCT absorption bands at 350 nm, although they do in frozen butyronitrile glass at 77 K. This has been previously attributed to a larger increase in the ^3^MLCT state energy than that of the nominally ^3^T_1g_ ligand field (LF) state in going from the homoleptic to *cis*-di­(methylisocyanide) complexes.^47^ This was proposed to result in a scenario in which the ^3^MLCT state is slightly lower in energy than the LF states in the *cis*-di­(methylisocyanide) complexes, such that at room temperature, there is sufficient thermal energy to populate the LF states and facilitate rapid excited-state deactivation. Whereas at 77 K, there is insufficient thermal energy to overcome the activation barrier to access the ^3^LF states, hence the observation of emission in frozen butyronitrile glass at 77 K.

Unlike the *cis*-di­(methylisocyanide) complexes, emission is observed at ∼640 nm in deaerated acetonitrile at room temperature for the Chugaev-type dicarbene complexes upon excitation into their MLCT absorption bands at 450 nm. Emission is partially quenched in aerated solution, consistent with phosphorescence from a state with predominantly triplet character. The Stokes shifts, lack of resolved vibrational fine structure, and emission quantum yields and lifetimes are consistent with ^3^MLCT phosphorescence, indicating that there is no fundamental change in the character of the emissive excited state between the homoleptic and corresponding Chugaev-type dicarbene complexes. This is further supported by calculated spin densities in the T_1_ states, which are all ^3^MLCT in character with no spin density on ancillary ligands. Compared to their homoleptic analogues, the Chugaev-type dicarbene complexes emit at longer wavelengths (641 and 636 nm for the bpy and dtbbpy systems, respectively, versus 613 and 617 nm for the corresponding homoleptic analogues).

Despite only minor changes in the optical properties of the Chugaev-type dicarbene complexes relative to the corresponding parent homoleptic complexes, they exhibit markedly lower emission quantum yields and lifetimes (Table 3). The radiative decay rate for the Chugaev-type dicarbene complexes is ∼10% slower than for the corresponding parent homoleptic complexes, consistent with the cubic dependence of the radiative rate constant on emission energy.^50–52^ The substantial decrease in emission quantum yields and lifetimes thus originates from the 3–4 fold increase in the nonradiative rate constant, *k*_nr_. Although *k*_nr_ includes contributions from both direct (vibrational) and indirect (thermally activated) processes, a nonradiative decay theory analysis (see SI p. S28–S37) indicates that the modest stabilization of the ^3^MLCT excited state in the Chugaev-type dicarbene complexes relative to their corresponding homoleptic analogues is sufficient to explain the concomitant increase in *k*_nr_, as described by the Energy-Gap Law. Variable temperature emission spectra and lifetimes, as well as Ru 2p4d resonant inelastic X-ray scattering (*vide infra*) further support the lack of excited state deactivation through indirect nonradiative decay. This means that improving the emission quantum yield and lifetime can be achieved simply through use of chromophoric ligands with more negative reduction potentials.

The Chugaev-type dicarbene complexes were also found to be particularly photosensitive in solution, with great care being required to avoid photolysis during emission quantum yield and lifetime measurements. This was a particularly surprising result, given that the X-ray crystal structures, electrochemical measurements and nonradiative decay theory analysis are all consistent with the Chugaev-type dicarbene ligand being a strong σ-donor, and thus expected to destabilize the dissociative LF states that result in ligand photorelease in previously studied Ru^II^ complexes – although the weaker interaction between the metal and chromophoric ligands could theoretically result in enhanced photolability of the latter.

We therefore sought to probe the thermal accessibility of higher lying LF states using variable temperature emission spectroscopy. The energy difference between the ^3^MLCT and LF states, ΔE, has been previously determined for [Ru­(bpy)_3_]^2+^ using Boltzmann analysis of variable temperature emission lifetimes in acetonitrile as 3,800 cm^–1^.^54^ Originally, the derivation of ΔE using Boltzmann analysis assumed thermal equilibrium between potential energy surface minima (ΔE_b_ in Figure 3).^16,54,57^ However, more recent interpretations treat ΔE as the activation energy for the rate limiting step in an Arrhenius analysis (Δ*E*_a_ in Figure 3).^58–60^ Recently, González and colleagues have reinterpreted ΔE using the *energetic span model* ^61–64^ as an effective energy barrier for thermally activated excited state deactivation through the ^3^LF states,^65^ i.e., the difference in energy between the ^3^MLCT potential energy surface minimum and the minimum energy crossing point (MECP) between the Jahn–Teller distorted ^3^T_1g_ LF state and the singlet ground state (ΔE_e_ in Figure 3). Using an Arrhenius-like model, they obtained ΔE_e_ of 3,500 cm^–1^ for [Ru­(bpy)_3_]^2+^ in acetonitrile.

**Figure 3:**
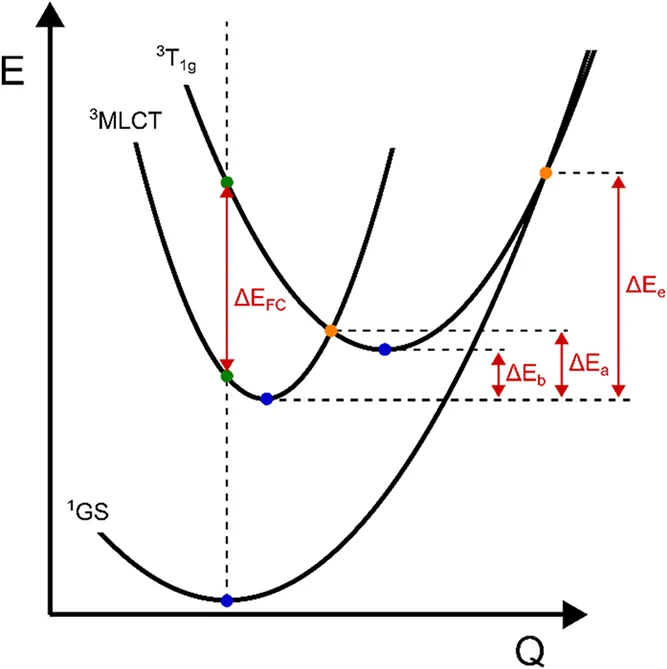
General schematic depiction of ^1^GS, ^3^MLCT and ^3^T_1g_ potential energy surfaces. Blue dots represent potential energy surface minima, green dots the Franck–Condon states and orange dots the minimum energy crossing points. Red arrows depict various energy differences between the ^3^MLCT and ^3^T_1g_ excited states discussed in the text.

In this work, we performed time-correlated single photon counting (TCSPC) to monitor the MLCT emission decay of [Ru­(bpy)_3_]­(PF_6_)_2_ and the Chugaev-type dicarbene complexes in deaerated acetonitrile over a 0–50 °C temperature range. Boltzmann- and Arrhenius-like analyses (Tables S10–S11) yield ΔE of 4,490 ± 20 and 4,650 ± 20 cm^–1^ respectively for [Ru­(bpy)_3_]­(PF_6_)_2_. These values exceed those previously reported, and may reflect the more limited temperature range accessible in this work. Compared to [Ru­(bpy)_3_]^2+^, the emission lifetimes of the Chugaev-type dicarbene complexes exhibit a much weaker temperature-dependence (Figure S25), consistent with thermal inaccessibility of destabilized LF states. Boltzmann and Arrhenius-like analyses provide activation energies between 4,600 and 5,000 cm^–1^ (Tables S10–S11) for both Chugaev-type dicarbene complexes.

To gain further insight into the excited state landscape, we recorded emission spectra of [Ru­(bpy)_3_]­(PF_6_)_2_ and the Chugaev-type dicarbene complexes in butyronitrile using a fluorimeter equipped with an optical cryostat (Figure 4). Consistent with previous reports, for [Ru­(bpy)_3_]­(PF_6_)_2_ we observe a dramatic decrease in emission intensity with increasing temperature above 250 K, consistent with deactivation via high energy LF states. However, below 260 K, we observe a second temperature-dependent behavior - a weaker decrease in emission intensity with a concomitant hypsochromic shift, exhibiting an isoemissive point at 588 nm. It has long been established that [Ru­(bpy)_3_]­(PF_6_)_2_ possesses three low-lying ^3^MLCT states within 100 cm^–1^ of one another,^66^ with a fourth ^3^MLCT state lying 500–600 cm^–1^ higher in energy.^67–70^ We therefore attribute this low temperature behavior to thermal population of the fourth ^3^MLCT state.

**Figure 4:**
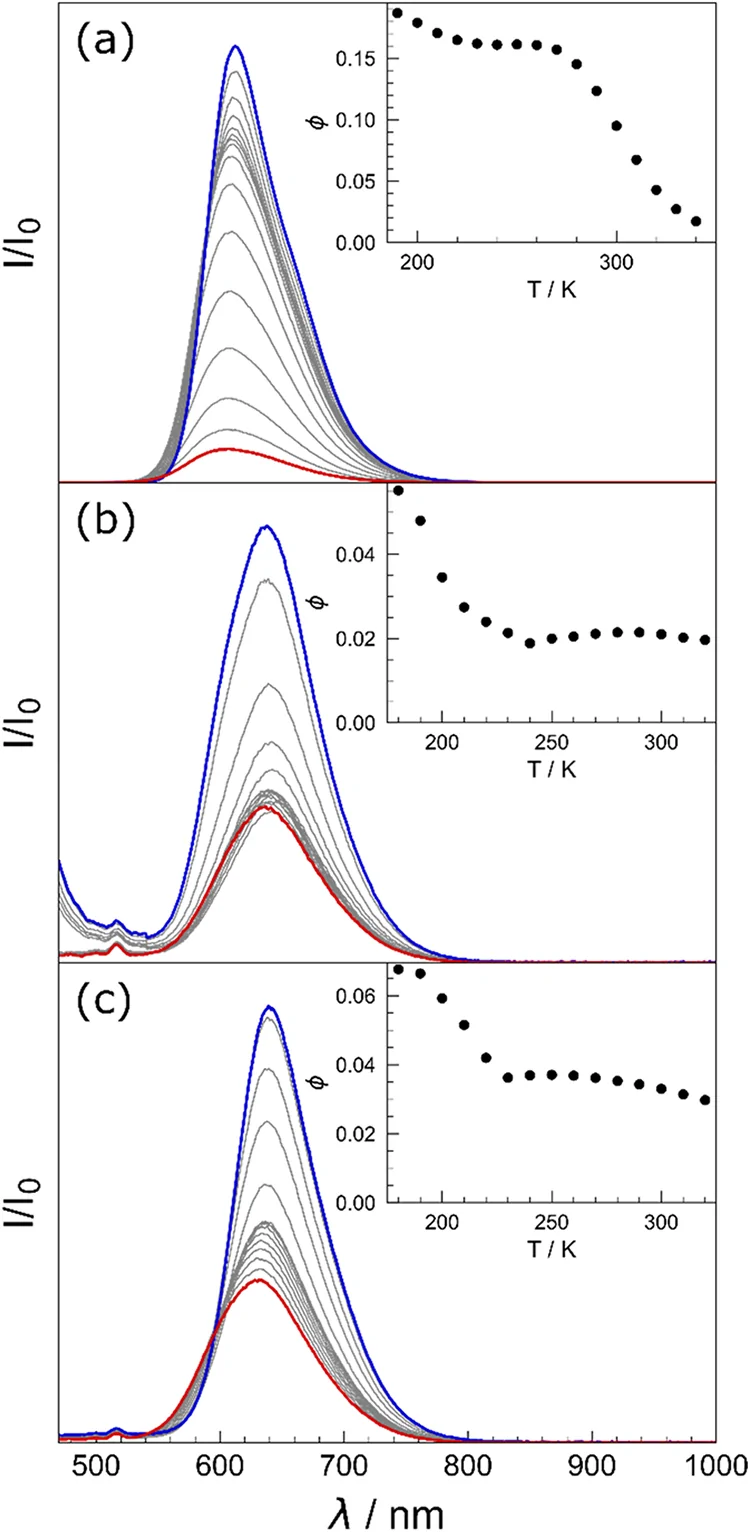
Variable temperature emission spectra of (a) [Ru­(bpy)_3_]­(PF_6_)_2_, (b) [Ru­(bpy)_2_(C_4_H_10_N_4_)]­(PF_6_)_2_ and (c) [Ru­(dtbbpy)_2_(C_4_H_10_N_4_)]­(PF_6_)_2_ in butyronitrile (λ_ex_ = 450 nm), recorded in 10 K intervals (blue = lowest temperature, red = highest temperature). Inset: emission quantum yields.

Similar to [Ru­(bpy)_3_]­(PF_6_)_2_, the Chugaev-type dicarbene complexes exhibit a decrease in emission intensity and concomitant hypsochromic shift at low temperatures that suggests interconversion between close-lying ^3^MLCT states, followed by a substantially weaker response at higher temperatures consistent with the observed weak temperature-dependence of the corresponding emission lifetimes. Combined, and further supported by Ru 2p4d resonant inelastic X-ray scattering (*vide infra*), these results suggest thermal inaccessibility of deactivating LF states in the relevant temperature regime.

Indirect (thermally activated) processes are thus unable to account for the increased rate of nonradiative decay in the Chugaev-type dicarbene complexes compared to the corresponding homoleptic analogues, supporting arguments from nonradiative decay theory that the increase in *k*_nr_ arises from Energy Gap Law effects alone. This further strongly supports nonparticipation of LF states in the photoreactivity of the Chugaev-type dicarbene complexes, which deductively must arise from the ^3^MLCT state. Although not unprecedented for photosubstitution reactions,^9,71^ direct photolability from MLCT states of Ru^II^ complexes is rare.

### Ru L_3_-Edge X-ray Spectroscopies

While emission spectroscopy demonstrates that the ^3^LF states are destabilized in the Chugaev-type dicarbene complexes, we were interested in gaining deeper understanding into the relative energies of LF states between the homoleptic, *cis*-di­(methylisocyanide) and Chugaev-type dicarbene complexes, and the determinative underlying electronic structure. We therefore employed X-ray absorption (XAS) and 2p4d resonant inelastic X-ray scattering (RIXS) at the Ru L_3_ edge to directly probe the higher energy LF states (Figure 5).

**Figure 5:**
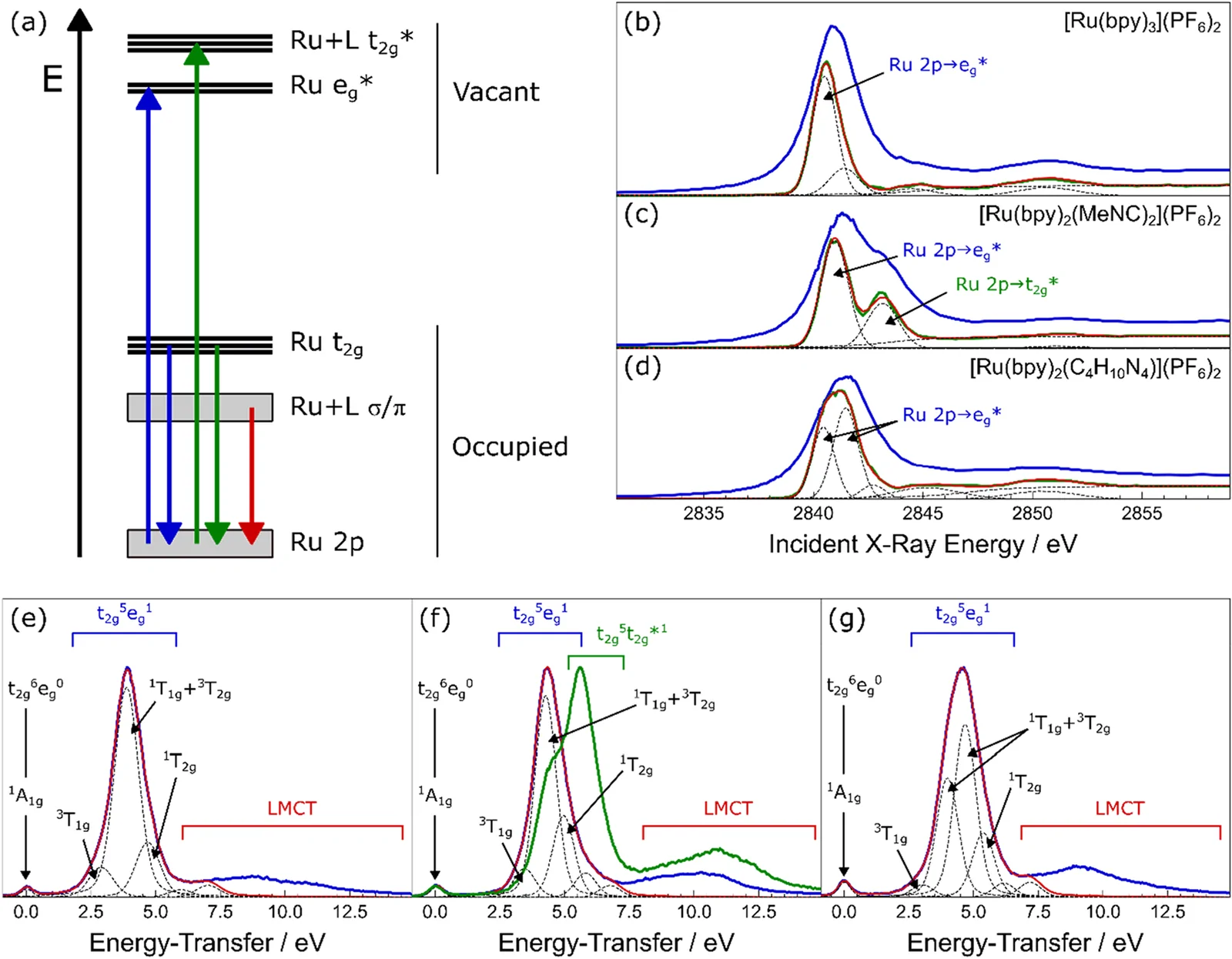
Ru L_3_-edge X-ray data. (a) Ru 2p→4d transitions observed in XAS spectra (upward oriented arrows) and Ru 4d→2p emission processes that give rise to valence final states observed in RIXS spectra (downward oriented arrows). Blue arrows give rise to t_2g_^5^e_g_^1^, green to t_2g_^5^t_2g_*^1^, and red to predominantly LMCT final states. Ru L_3_-edge XAS spectra (solid blue traces = PFY, solid green traces = HERFD) of (b) [Ru­(bpy)_3_]­(PF_6_)_2_, (c) [Ru­(bpy)_2_(MeNC)_2_]­(PF_6_)_2_ and (d) [Ru­(bpy)_2_(C_4_H_10_N_4_)]­(PF_6_)_2_. Solid red traces correspond to fits of the experimental HERFD data to a series of pseudo-Voigt peaks (with the edge-jump modeled as an arctangent function), and dashed black traces to individual fit components. Ru 2p4d RIXS spectra of (e) [Ru­(bpy)_3_]­(PF_6_)_2_, (f) [Ru­(bpy)_2_(MeNC)_2_]­(PF_6_)_2_ and (g) [Ru­(bpy)_2_(C_4_H_10_N_4_)]­(PF_6_)_2_. Blue traces have been measured with incident X-ray energies corresponding to the e_g_* resonance in XAS spectra, green to the t_2g_* resonance. Solid red traces correspond to fits of the experimental data to a series of pseudo-Voigt peaks, and dashed black traces to individual fit components. Assignments are provided for t_2g_^5^e_g_^1^ final states.

Ru L_3_ XAS spectra recorded as partial fluorescence yield (PFY) and high energy-resolution frequency detection (HERFD) are presented in Figure 5b–d for the bpy-based complexes and Figures S26–S28 for the dtbbpy-based complexes. At the Ru L_3_ edge, the incident X-ray energy is resonant with Ru 2p_3/2_→4d electronic transitions. For clarity, we herein adopt octahedral symmetry labels for interpreting and discussing Ru L_3_ XAS and RIXS results. All Ru^II^ complexes investigated possess a low-spin d^6^ electronic configuration. The Ru 4d orbitals are therefore split by bonding with σ-symmetry ligand group orbitals into the nonbonding t_2g_ and antibonding e_g_* orbital sets (with the corresponding bonding e_g_ set possessing predominantly ligand character). The 6 *d*-electrons fully occupy the t_2g_ orbital set, such that, assuming minimal π-backbonding, the only vacant orbitals to accept an electron in the 2p→4d electronic transition are the e_g_* orbitals (Figure 5a).

In the PFY XAS spectra, peaks are broadened by both the experimental resolution (Gaussian broadening) and the 2p core hole lifetime broadening (Lorentzian, 1.75 eV^72^). Fits of the experimental PFY XAS spectra to pseudo-Voigt peaks with the Lorentzian component set at 1.75 eV are presented in Figure S29. In the HERFD XAS spectrum, by selectively integrating a narrow emission range centered on the peak of the nonresonant Lα_1_ emission line (3d_5/2_ → 2p_3/2_), lifetime broadening is instead determined by the longer-lived 3d hole (∼0.25 eV), resulting in narrower spectra and allowing deconvolution of the PFY XAS peaks. Indeed, the experimental HERFD XAS spectra were adequately modeled using Gaussian peaks (Figure S30), indicating that lifetime broadening is narrower than the experimental resolution.

For [Ru­(bpy)_3_]­(PF_6_)_2_ (Figure 5b), the Ru L_3_ XAS spectrum is dominated by a single peak, corresponding to the Ru 2p→e_g_* transition. It should be noted that Ru^II^ polypyridyl complexes possess a not insignificant degree of metal–ligand covalency, and computational work has suggested that for [Ru­(bpy)_3_]­(PF_6_)_2_ this peak contains a significant contribution from Ru 2p→bpy­(π*) transitions (up to ∼30%).^73^ The higher energy tail of this peak is tentatively assigned to a 2p→t_2g_* transition arising due to splitting of the t_2g_ orbital set as a consequence of π-backbonding. The low intensity of this transition can be explained by the t_2g_* orbital set being predominantly ligand-based and the intensity of the XAS signal being proportional to the Ru 4d character of the accepting orbital.

For the *cis*-di­(methylisocyanide) complexes (Figures 5c andS28–S30), this higher energy t_2g_* resonance is significantly more pronounced, appearing as a high energy shoulder to the main e_g_* resonance in the PFY XAS spectra, and as a distinct separate peak in the HERFD XAS spectra. The greater intensity of this peak likely arises because of the strong mixing of Ru t_2g_ and methylisocyanide π* orbitals due to π-backbonding, consistent with the structural and electrochemical data reported above. Further consistent with this assignment is the observation of a similar feature in the Ru L_3_-edge XAS spectrum of [Ru­(CN)_6_]^4–^.^73^

For the Chugaev-type dicarbene complexes, the main e_g_* resonance appears as a single, broad feature in the PFY XAS spectra, resolving into two overlapping bands in the HERFD XAS spectra, separated by ∼1 eV (Tables S9–S10). This indicates splitting of the degenerate e_g_* orbitals due to differential interaction with the strongly σ-donating Chugaev-type dicarbene ligands. Notably, such splitting of the e_g_* resonance is not observed for the *cis*-di­(methylisocyanide) complexes, which formally possess the same symmetry as the Chugaev-type dicarbene complexes. Such splitting of the e_g_ orbital sets could also justify the observed *trans* influence in the X-ray crystal structures – by splitting the Ru d_*z* ^2^_ and d_*x* ^2^–*y* ^2^_ orbitals, they interact to different extents with the chromophoric ligand σ orbitals, resulting in differential bond lengths between axial and equatorial positions.

In the Ru L_3_-edge XAS spectra of all investigated complexes, an additional feature above the ionization potential (∼2851 eV) can be observed, which has been previously assigned in related complexes as a quasi-bound above ionization resonance arising from multiple scattering of the photoelectron with surrounding ligand atoms.^74^

Valence-to-core (4d→2p) X-ray emission spectra were further collected at incident energies resonant with X-ray absorption features. This process is illustrated in Figure 5a, yielding 2p4d RIXS spectra that are interpreted on an Energy-Transfer axis, i.e., the energy difference between the incident and emitted X-rays, and contain information on valence excitations. The 2p4d RIXS spectra of the bpy-based complexes are presented in Figure 5e–g, and the dtbbpy-based complexes in Figures S26 and S29.

When the incident X-ray energy is tuned to the main e_g_* resonance of the Ru L_3_ XAS spectrum, populating the 2p^5^e_g_^1^ core excited state, and the 2p core hole is refilled with an electron from the t_2g_ orbital set, a t_2g_^5^e_g_^1^ final valence excited state is formed corresponding to a single electron interconfigurational LF excitation (Figure 5a). The Raman selection rules and atomic specificity of RIXS dictate that the occupied orbital involved in the scattering process necessarily possesses the same character as the unoccupied orbital in the resonant electronic transition (i.e., Ru 4d for Ru 2p4d RIXS) and that parity be conserved, which gives rise to the unique sensitivity of metal L-edge RIXS to single electron excitation interconfigurational LF transitions. In this, RIXS is complementary to UV–vis electronic absorption spectroscopy, in which the electric-dipole forbidden LF transitions of Ru^II^ polypyridyl complexes cannot be observed due to spectral overlap with much more intense CT and π,π* transitions. As with UV–vis electronic absorption spectroscopy, it should be noted that RIXS obeys the Franck–Condon principle, and therefore that observed transitions represent vertical excitations on a potential surface energy diagram, not conformationally relaxed potential energy surface minima.

Although 2p3d RIXS at 3d metal L_3_ edges is now reasonably well-established for investigating LF states in first row transition metal complexes,^24,75–80^ the unavailability of high energy-resolution emission spectrometers operating in the tender X-ray region has historically limited such studies on second row transition metal complexes.^81,82^ However, recent advances in high energy-resolution tender X-ray spectroscopy at the Stanford Synchrotron Radiation Lightsource (SSRL)^83^ have enabled 2p4d and 2p3d RIXS investigation of Ru complexes.^73,84,85^

Upon excitation into the e_g_ resonance at the Ru L_3_ edge, qualitatively similar spectra (blue traces in Figures 5e–g andS28) are obtained for all complexes, with the main features corresponding to the above-mentioned t_2g_^5^e_g_^1^ interconfigurational LF states. As with e_g_* resonance in the Ru L_3_ edge XAS, computational work has assigned some MLCT character to this predominantly LF feature in related complexes.^73^ The less intense, higher energy-transfer features arise from emission from deeper occupied ligand π and/or σ orbitals with mixed Ru 4d character, and correspond then to predominantly ligand-to-metal charge-transfer (LMCT) final states. Upon excitation into the high energy shoulder (t_2g_* resonance) of the methylisocyanide complexes (green traces Figures 5f and S28e), a higher energy-transfer resonant feature is observed, corresponding to t_2g_^5^t_2g_*^1^ final states, likely with significant CT character due to the inherently high ligand character of the t_2g_* orbital set. As with the XAS spectra, similar RIXS features have been reported for [Ru­(CN)_6_]^4–^.^73^

The 2p4d RIXS spectra generated upon excitation into the e_g_* resonance at the Ru L_3_ edge, corresponding to t_2g_^5^e_g_^1^ LF states, were fitted to a series of pseudo-Voigt peaks (Figure 5e–g). Negative second derivative spectra were used to determine the number of bands comprising the main RIXS feature, and to assess fit accuracy (Figure S31). For the homoleptic and *cis*-di­(methylisocyanide) complexes, three bands were required to accurately model the main features, which we assign to the ^3^T_1g_, a convolution of ^3^T_2g_ and ^1^T_1g_, and ^1^T_2g_ LF transitions, analogous to isoelectronic Fe^II^ complexes.^24,75^ For the Chugaev-type dicarbene complexes, however, the negative second derivative spectra clearly indicate splitting of the main ^3^T_2g_/^1^T_1g_ band. This splitting is too large to arise from separation of the ^3^T_2g_ and ^1^T_1g_ states, with the two bands also exhibiting relative intensities inconsistent with expectation, and must therefore derive from the splitting of the e_g_* resonance observed in the XAS spectra. Due to their extent of overlap, we are unable to selectively excite into these two e_g_* bands. Although the splitting of main ^3^T_2g_/^1^T_1g_ band is the most evident consequence of the splitting of the e_g_* orbitals, the lower energy ^3^T_1g_ state will also undergo similar splitting, with the higher energy state masked by the more intense lower energy ^3^T_2g_/^1^T_1g_ feature.

From the RIXS spectra, the nominally ^3^T_2g_ state appears to lie approximately 0.8 – 1 eV higher in energy than the ^3^T_1g_ for all investigated complexes (Table S14). The ^3^T_1g_ LF state is thus most likely primarily responsible for indirect (thermally activated) deactivation of the MLCT state, with negligible participation from higher ^3^LF states. Comparing the energies of the ^3^T_1g_ LF state, it can be observed that both the methylisocyanide and Chugaev-type dicarbene ligands destabilize the ^3^T_1g_ state relative to the homoleptic complexes, but the effect is substantially greater for the *cis*-di­(methylisocyanide) complexes (∼0.5 eV) than the Chugaev-type dicarbene complexes (∼0.1 eV) (Table S14). That strong π-acceptor ligands are more effective at destabilizing LF states than strong σ-donor ligands has some important implications for the design of photosensitizers with enhanced photostability, as well as in the design of earth-abundant photosensitizers wherein LF states provide efficient excited state deactivation channels.

Given the complementarity between electronic absorption spectroscopy, sensitive to charge-transfer excitations and 2p4d RIXS, sensitive to LF excitations, both providing vertical excitation (Franck–Condon) energies, the difference in Franck–Condon energies (ΔE_FC_ in Figure 3) can be elucidated from the corresponding spectral fits. ΔE_FC_ values are reported for the homoleptic and Chugaev-type dicarbene complexes in Table 4. We were unable to extract vertical ^3^MLCT excitation energies from UV–vis data for the *cis*-di­(methylisocyanide) complexes, which were thus unable to be included. It can be observed that the Chugaev-type dicarbene ligands both stabilize the ^3^MLCT and destabilize the ^3^T_1g_ Franck–Condon state, resulting in substantially larger ΔE_FC_ values compared to the corresponding homoleptic complexes. Given that the two methods (UV–vis/RIXS versus variable temperature emission lifetimes) probe different aspects of the excited state landscape, neither of which is the difference in energy between vibrationally relaxed potential energy surface minima, it is clear that caution must be exercised when drawing conclusions about excited state energies from either method. However, the increase in both ΔE_FC_ and ΔE_e_ from the homoleptic to Chugaev-type dicarbene complexes strongly supports destabilization of the ^3^LF states relative to the ^3^MLCT, consistent with expectation for strong σ-donor ligands.^21,86^

**Table 4:** ^3^MLCT and ^3^T_1g_ Franck-Condon State Energies Determined Through Spectral Bandshape Analysis of UV–vis Electronic Absorption and Ru 2p4d RIXS Spectra^c^

complex	^3^MLCT (FC)/cm^–1^ ^a^	^3^T_1g_ (FC)/cm^–1^ ^b^	ΔE_FC_/cm^–1^
[Ru(bpy)_3_](PF_6_)_2_	20,190(50)	23,070(80)	2,900(100)
[Ru(bpy)_2_(C_4_H_10_N_4_)](PF_6_)_2_	18,380(30)	24,400(200)	6,000(300)
[Ru(dtbbpy)_3_](PF_6_)_2_	19,650(50)	22,510(70)	2,860(90)
[Ru(dtbbpy)_2_(C_4_H_10_N_4_)](PF_6_)_2_	18,470(40)	23,500(200)	5,000(300)

a Obtained from spectral band shape fitting of electronic absorption spectra.

b Obtained from fitting of Ru 2p4d RIXS spectra.

c Uncertainties are reported at the 95% confidence interval.

### Hydrazine Photorelease

Intrigued by the unexpectedly high photosensitivity of the Chugaev-type dicarbene complexes, with photolysis unusually occurring directly from the ^3^MLCT excited state, we sought to understand the photochemistry of the Chugaev-type dicarbene complexes in more detail.

Solutions of the Chugaev-type dicarbene and corresponding homoleptic complexes in acetonitrile were irradiated at 450 nm using the monochromated output of a 450 W xenon lamp, and electronic absorption and emission spectra recorded at 5 min intervals (Figures 6 and S32). Over the course of 90 min, the [Ru­(bpy)_2_(C_4_H_10_N_4_)]­(PF_6_)_2_^3^MLCT emission is quenched by ∼90% (Figure 6c). There is no observable concomitant growth of another emission signal, suggesting formation of a nonemissive photoproduct. This is consistent with the photosubstitution of [Ru­(bpy)_3_]­(PF_6_)_2_, which also produces a nonemissive photoproduct. However, under identical conditions, the [Ru­(bpy)_3_]­(PF_6_)_2_^3^MLCT emission is quenched by only ∼ 8.5% (Figure S32). The stark difference in photolysis rates between the two complexes is suggestive of different operative mechanisms.

**Figure 6:**
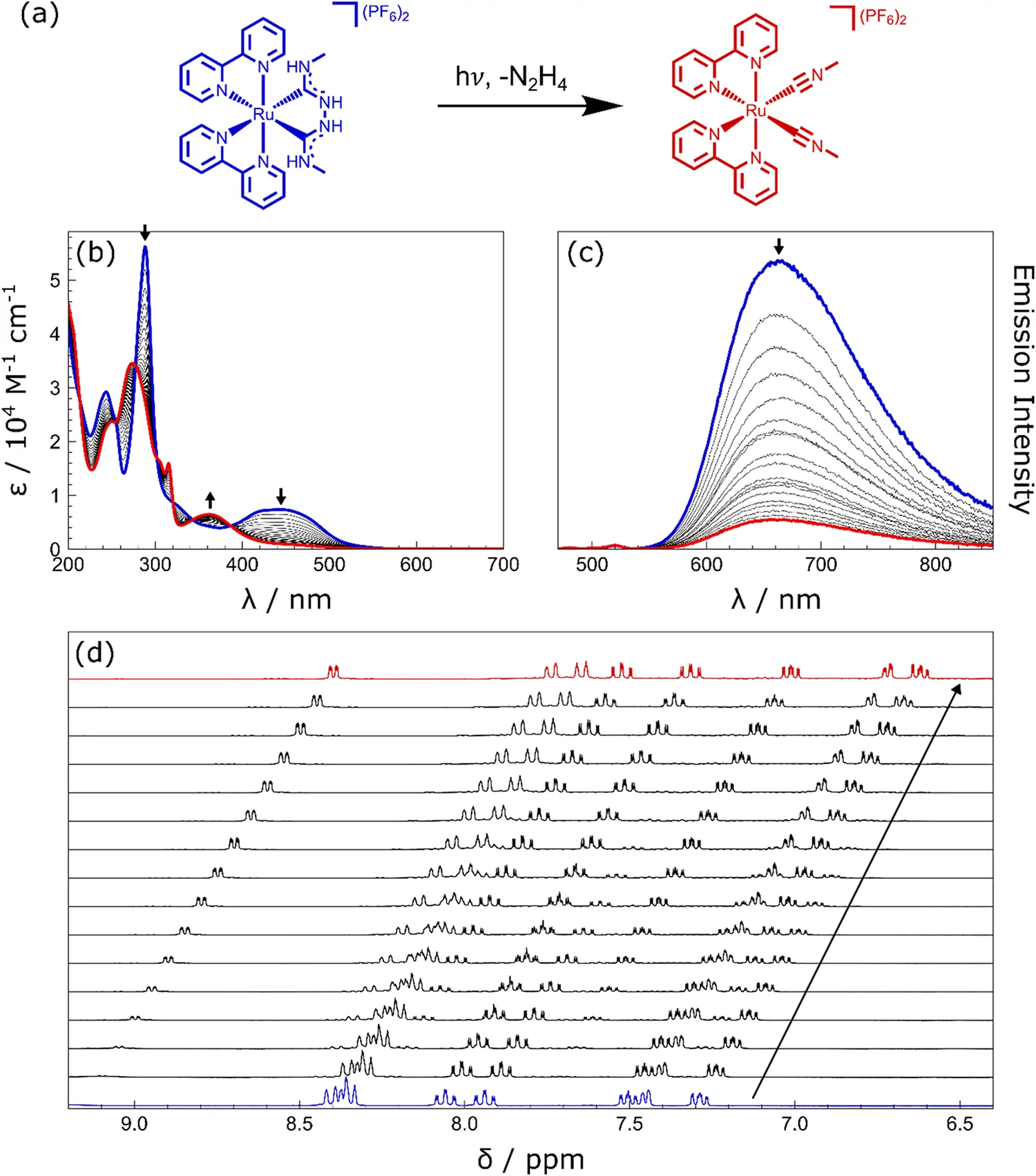
Blue light photolysis of [Ru­(bpy)_2_(C_4_H_10_N_4_)]­(PF_6_)_2_. (a) Proposed photolysis reaction scheme. (b) Electronic absorption and (c) emission (λ_ex_ = 450 nm) spectra of deaerated acetonitrile solutions of [Ru­(bpy)_2_(C_4_H_10_N_4_)]­(PF_6_)_2_ upon irradiation with 450 nm light. Solutions had an initial optical density of 0.1 at 450 nm. Spectra were recorded every 5 min for 90 min. (d) ^1^H NMR spectra of [Ru­(bpy)_2_(C_4_H_10_N_4_)]­(PF_6_)_2_ in CD_3_CN upon irradiation with 460 nm light. Spectra were recorded every 10 min for 150 min. Blue traces are the initial spectrum at *t* = 0 min, and red traces the final spectra. Black arrows denote direction of photoinduced changes.

The change in UV–vis spectra of [Ru­(bpy)_2_(C_4_H_10_N_4_)]­(PF_6_)_2_ upon irradiation is more informative in this respect. Clear isosbestic points are observed, indicating a clean reactant → photoproduct photoreaction. The red spectrum in Figure 6b, after 90 min of irradiation, is qualitatively similar to that of [Ru­(bpy)_2_(MeNC)_2_]­(PF_6_)_2_, the direct synthetic precursor of [Ru­(bpy)_2_(C_4_H_10_N_4_)]­(PF_6_)_2_.

To more thoroughly assign the photoproduct, we irradiated a sample of [Ru­(bpy)_2_(C_4_H_10_N_4_)]­(PF_6_)_2_ in CD_3_CN with 460 nm LEDs (average power 20 mW cm^–2^), and recorded ^1^H NMR spectra in 10 min intervals (Figures 6d and S37–S38). The photoreaction proceeded cleanly, with loss of the original [Ru­(bpy)_2_(C_4_H_10_N_4_)]­(PF_6_)_2_ signals, and growth of a single set of new signals that can be unambiguously assigned to [Ru­(bpy)_2_(MeNC)_2_]­(PF_6_)_2_ (Figure S41). As [Ru­(bpy)_2_(C_4_H_10_N_4_)]­(PF_6_)_2_ is prepared from [Ru­(bpy)_2_(MeNC)_2_]­(PF_6_)_2_ by chelative addition of hydrazine across the *cis*-coordinated methylisocyanide ligands, our results suggest a photoinduced dechelative elimination of hydrazine. There is no evidence of photolability or any form of photosubstitution reaction typical for Ru^II^ polypyridyl complexes. The Chugaev-type dicarbene complexes can therefore be considered to photocage hydrazine.

The photoinduced elimination of hydrazine from the Chugaev-type dicarbene complexes occurring directly from the ^3^MLCT excited state is fascinating for several reasons. First, Chugaev-type dicarbene complexes have historically been studied both for their strong σ-donor characteristics and high stability, particularly in photoluminescence-based applications.^87^ The origin of this anomalous ‘ligand-centered’ photochemical process in Ru^II^ complexes is therefore intriguing. Second, in traditional Ru^II^ photocages, the photoproduct is coordinatively unsaturated (with vacant coordination sites occupied by solvent molecules),^9,19^ whereas for the Chugaev-type dicarbene complexes, it is coordinatively saturated, which could have interesting connotations for photoactivated chemotherapy applications. Indeed, that the Chugaev-type dicarbene can be regenerated from the photoproduct introduces the potential for recyclable photocages. Finally, by photoreleasing directly from the photogenerated ^3^MLCT excited state, enhanced/fine-tuned photorelease efficiencies could be achieved.

To understand why hydrazine photorelease might occur from the thermally equilibrated ^3^MLCT states, we performed TD-DFT calculations from the T_1_ state (assigned as ^3^MLCT from spin density, Table S20). TD-DFT calculations suggest that even though there is no carbene involvement in the T_1_ state, there are an additional three ^3^MLCT states within 5,000 cm^–1^ (Franck–Condon energies) that are likely thermally populated at room temperature (Table S24). One of these states has substantial ligand-to-ligand charge-transfer (LLCT) character (carbene → bpy), which may tentatively be responsible for the observed hydrazine photorelease.

To understand photorelease efficiencies of the Chugaev-type dicarbene complexes, the spectral changes in both absorption and emission spectra were globally fitted to the same two-state kinetic model (see SI P.S49–S55):^88,89^

1 $$-\frac{\partial \left[R\right]}{\partial t}=q_{V}\cdot \phi_{q}\cdot \left(1-10^{{-A}_{t}}\right)\cdot \frac{\varepsilon_{R}l\left[R\right]}{A_{t}}$$

Where ϕ_PR_ is the quantum yield of the photoreaction, q_V_ the incident volumetric photon flux (in einstein L^–1^ s^–1^), A_t_ the absorbance at the irradiation wavelength at a given time, ε_R_ the molar absorption coefficient of the reactant at the irradiation wavelength, and *l* the optical path length. A similar analysis could not be performed on the NMR data due to the appropriate concentration range imbuing a high initial optical density, resulting in a strong inner-filter effect (see SI P.S56–S60).

The species-associated spectra and irradiation time-dependent concentrations for the photoinduced hydrazine elimination of [Ru­(bpy)_2_(C_4_H_10_N_4_)]­(PF_6_)_2_ from global fitting are presented in Figure 7, and for [Ru­(dtbbpy)_2_(C_4_H_10_N_4_)]­(PF_6_)_2_ in Figure S38. In both cases, the species-associated spectrum of the photoproduct (solid red traces) clearly aligns with the corresponding *cis*-di­(methylisocyanide) complex (dashed black traces).

**Figure 7:**
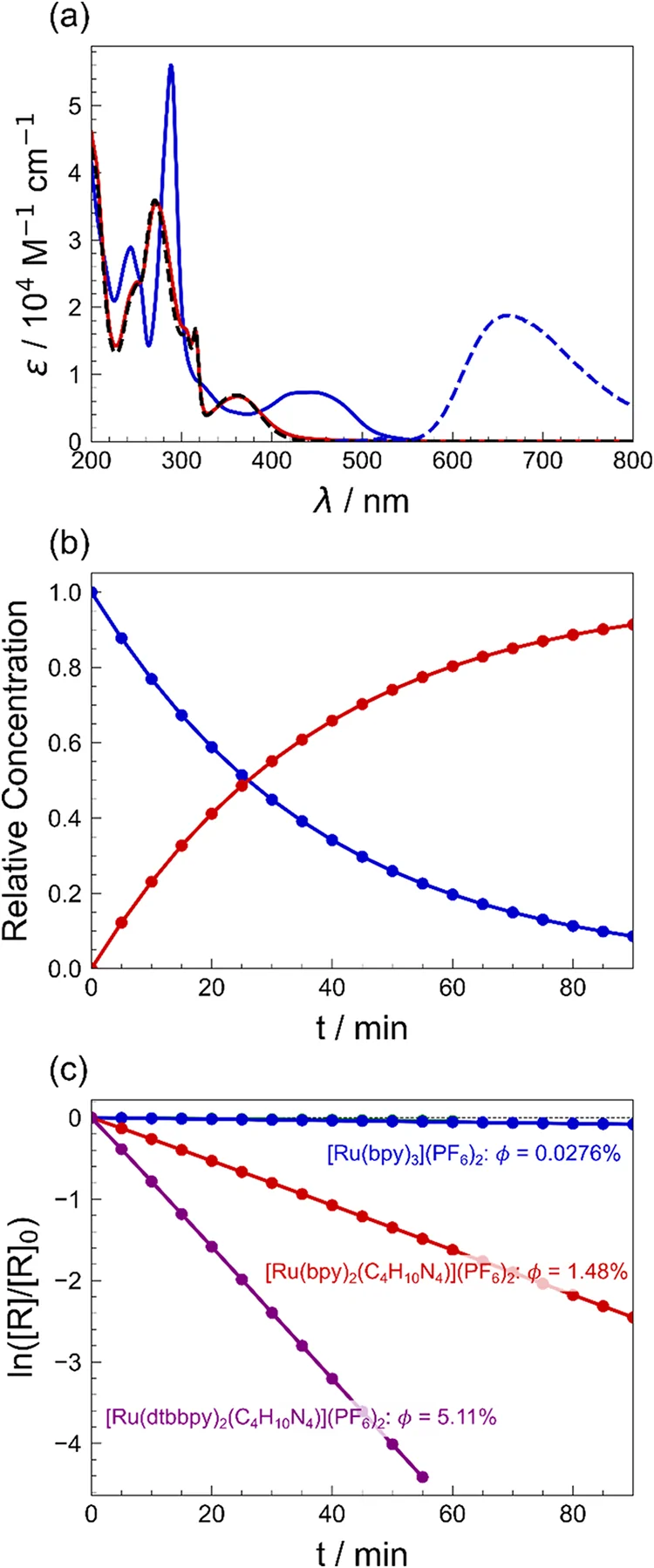
(a) Species associated spectra (SAS) from global fitting of electronic absorption and emission spectra of deaerated acetonitrile solutions of [Ru­(bpy)_2_(C_4_H_10_N_4_)]­(PF_6_)_2_ upon irradiation with 450 nm light. Solid blue trace is the electronic absorption SAS of reactant, solid red trace is the electronic absorption SAS of photoproduct, dashed blue trace is the emission SAS of reactant, black dashed trace is the UV–vis electronic absorption spectrum of [Ru­(bpy)_2_(MeNC)_2_]­(PF_6_)_2_ from Figure 2. (b) Relative concentrations of reactant (blue) and photoproduct (red) over the course of the photoreaction obtained from global fitting. (c) Relative concentrations of reactant over the course of the photoreaction obtained from global fitting of electronic absorption and emission spectra of deaerated acetonitrile solutions of [Ru­(bpy)_3_]­(PF_6_)_2_, [Ru­(bpy)_2_(C_4_H_10_N_4_)]­(PF_6_)_2_ and [Ru­(dtbbpy)_2_(C_4_H_10_N_4_)]­(PF_6_)_2_ upon irradiation with 450 nm light, and photoreaction quantum yields obtained by relative actinometry (see SI p.S49–S55).

Using the reported quantum yield for photorelease (ϕ_PR_) in [Ru­(bpy)_3_]­(PF_6_)_2_ (0.0276%) as reference,^28^ ϕ_PR_ values for the other complexes were determined by a relative actinometric approach (see SI P.S49–S55, Table S15). The relative reactant concentrations as a function of irradiation time are presented in Figure 7c. [Ru­(dtbbpy)_3_]­(PF_6_)_2_ is slightly more photostable than [Ru­(bpy)_3_]­(PF_6_)_2_, with ϕ_PR_ = 0.0232%. For [Ru­(bpy)_2_(C_4_H_10_N_4_)]­(PF_6_)_2_, ϕ_PR_ = 1.48%, and for [Ru­(dtbbpy)_2_(C_4_H_10_N_4_)]­(PF_6_)_2_, ϕ_PR_ = 5.11%. It is notable that [Ru­(bpy)_2_(C_4_H_10_N_4_)]­(PF_6_)_2_ has a photorelease quantum yield ∼50× greater than that of [Ru­(bpy)_3_]­(PF_6_)_2_. This can largely be attributed to the mechanism of photorelease in [Ru­(bpy)_3_]­(PF_6_)_2_ requiring thermal population of higher energy dissociative LF states, whereas it occurs directly from the photogenerated ^3^MLCT excited state for [Ru­(bpy)_2_(C_4_H_10_N_4_)]­(PF_6_)_2_.

Interestingly, while *tert*-butyl groups imbue additional stability against ligand photosubstitution via thermal population of LF states in the homoleptic complexes, they increase the photorelease quantum yield by a factor of 3 in the Chugaev-type dicarbene complexes. Given that the *tert*-butyl groups are located at the periphery of the chromophoric ligands, their influence is unlikely to be steric, but rather electronic. However, it is intriguing to note that differences in the electronic properties of the two complexes are mostly subtle. The most obvious difference appears to be that [Ru­(dtbbpy)_2_(C_4_H_10_N_4_)]­(PF_6_)_2_ stores ∼90 meV more energy than [Ru­(bpy)_2_(C_4_H_10_N_4_)]­(PF_6_)_2_ in its ^3^MLCT excited state, resulting in a ∼ 30% increase in excited state lifetime. A more comprehensive investigation into the mechanism of photoinduced hydrazine elimination and the role of chromophoric ligands therein will be the focus of upcoming work.

The quantum yields for photoinduced hydrazine elimination from the Chugaev-type dicarbene complexes fall squarely in the ideal range for PACT recently proposed by Bonnet.^9^ This raises the potential for this class of complex as an alternative scaffold for photocaging bioactive molecules or chemotherapeutics through substituted hydrazines. Indeed, several hydrazine-based drugs show anticancer activity (e.g., procarbazine).

### Conclusions

In the pursuit of photoactive Ru^II^ complexes with similar photophysical properties to [Ru­(bpy)_3_]^2+^ and related complexes, but with enhanced photostability, we report two photoactive Ru^II^ complexes bearing strongly σ-donating Chugaev-type dicarbene ligands. Using a combination of X-ray crystallography, electrochemistry, optical spectroscopy and X-ray spectroscopy, we compared how the Chugaev-type dicarbene ligands perturb the electronic structure and consequent photophysical properties of the complex to π-accepting methylisocyanide ligands. From Ru 2p4d RIXS, we were able to demonstrate that the π-acceptor isocyanide ligands more effectively destabilize LF states than the σ-donor Chugaev-type dicarbene ligands, which has important connotations for future design of photoactive first-row transition metal complexes. Although isocyanide ligands more effectively destabilize LF states, they also destabilize MLCT states, leading to poorer visible absorption and photophysical properties, whereas the Chugaev-type dicarbene ligands exhibit comparable optical properties to [Ru­(bpy)_3_]^2+^. From Ru 2p4d RIXS and variable temperature emission spectroscopy, we were able to show that higher energy LF states are not thermally accessible from photogenerated ^3^MLCT excited states in the Chugaev-type dicarbene complexes. Lower emission quantum yields and shorter excited state lifetimes could therefore be attributed exclusively to Energy-Gap Law effects, and thus can be overcome by use of different chromophoric ligands.

Despite the photophysical properties of the Chugaev-type dicarbene complexes being extremely promising for our original objective of developing photostable photosensitizers with similar photochemical properties to [Ru­(bpy)_3_]^2+^, the complexes were found to be highly photosensitive. This arises from an unexpected photoinduced dechelative elimination of hydrazine directly from the ^3^MLCT excited state to form the corresponding *cis*-di­(methylisocyanide) complexes – the immediate synthetic precursors to the Chugaev-type dicarbene complexes.

Our findings contribute to understanding of molecular design principles for Ru-based photosensitizers targeted at either dissociative or nondissociative photophysical, photochemical or photobiological applications, and highlight the utility of X-ray spectroscopies as complementary tools to optical spectroscopies for understanding electronic structure and excited state landscapes of photoactive coordination compounds.

Given the purported photostability of previously reported Chugaev-type dicarbene complexes, our findings also introduce new considerations for their application in luminescence-based applications, as well as those of related acyclic diaminocarbenes. Furthermore, our results highlight the potential of Ru^II^ Chugaev-type dicarbene complexes as a potential new scaffold for PACT applications with a fundamentally different photorelease mechanism.

## Supplementary Material



## Data Availability

The research data supporting this publication can be accessed at DOI: 10.17608/k6.auckland.29378540.

## Notes

Experimental details; crystallography; electrochemistry; optical spectroscopy data tables; figures and additional discussion; time-correlated single photon counting; X-ray spectroscopy data tables; figures and additional discussion; photolysis data tables; figures and additional discussion; and NMR characterization data (PDF)
