# Supplementary material for: Hydrazine Photorelease from Ru(II) Chugaev-Type Dicarbene Complexes
Source: J Am Chem Soc. 2026 Sep 9;148(37):40277–91. doi: 10.1021/jacs.6c13259 (PMC13614201; doi:10.1021/jacs.6c13259)
Supplement: Supplementary file 1 [file ja6c13259_si_001.pdf]

Hydrazine Photorelease from Ru(II) Chugaev-Type Dicarbene Complexes

SUPPORTING INFORMATION

Subhradip Kundu,[1] Xuewen Zhang,[2] Conor J. Doran,[2] Sian E. Dillon,[2] Eugene In,[2] Ryan D. Ribson,[3] Céline Besnard,[4] Laure Guénée,[4] Marcus Jones,[5,6,7] Dimosthenis Sokaras,[3] Elisa Biasin*,[8] and Christopher B. Larsen*[1,2,6]

christopher.larsen@auckland.ac.nz

elisa.biasin@pnnl.gov

[1] *Department of Inorganic and Analytical Chemistry, University of Geneva, CH-1211 Geneva, Switzerland*

[2] *School of Chemical Sciences, University of Auckland, Auckland 1010, New Zealand*

[3] *SSRL, SLAC National Accelerator Laboratory, Menlo Park, California 94025, USA*

[4] *Laboratory of Crystallography, University of Geneva, 24 quai Ernest Ansermet, 1211 Geneva, Switzerland*

[5] *School of Science, Auckland University of Technology, Auckland 1010, New Zealand*

[6] *MacDiarmid Institute for Advanced Materials and Nanotechnology, Auckland 1010, New Zealand*

[7] *Te Whai Ao – Dodd-Walls Centre for Photonic and Quantum Technologies, Dunedin 9016, New Zealand*

[8] *Pacific Northwest National Laboratory, Richland, Washington 99352, USA*

Table of Contents

Experimental ... S3

General Experimental ... S3

Materials ... S3

$[Ru(bpy)_2(MeNC)_2](PF_6)_2$ ... S3

$[Ru(^tBu_2bpy)_2(MeNC)_2](PF_6)_2$ ... S4

$[Ru(bpy)_2(C_4H_{10}N_4)](PF_6)_2$ ... S5

$[Ru(^tBu_2bpy)_2(C_4H_{10}N_4)](PF_6)_2$ ... S7

Physical and Spectroscopic Measurements ... S8

NMR and Analytical Measurements ... S8

X-ray structure analyses ... S8

Electrochemistry ... S9

Optical Spectroscopy ... S9

X-Ray Spectroscopy ... S10

Computational Details ... S11

X-Ray Crystallography ... S13

Electrochemical Measurements ... S19

Optical Spectroscopy ... S20

Molar Absorption Coefficient Determination ... S20

Photoluminescence Quantum Yield Determination ... S23

Spectral Bandshape and Nonradiative Decay Theory Analysis ... S28

Time Correlated Single Photon Counting ... S38

Ru L_3-Edge X-Ray Spectroscopy ... S42

Photolysis Studies ... S49

Computational Results ... S61

Ground State (S_0) DFT Results ... S62

Ground State (S_0) TD-DFT Results ... S64

Excited State (T_1) DFT Results ... S68

Excited State (T_1) TD-DFT Results ... S70

XYZ Coordinates of Geometry-Optimized Structures ... S73

NMR Spectroscopy ... S89

$[Ru(bpy)_2(MeNC)_2](PF6)_2$ ... S89

$[Ru(bpy)_2(C_4H_{10}N_4)_2](PF6)_2$ ... S93

$[Ru(dtbbpy)_2(MeNC)_2](PF6)_2$ ... S97

$[Ru(dtbbpy)_2(C_4H_{10}N_4)_2](PF6)_2$ ... S101

References ... S105

Experimental

General Experimental

Commercially available reagents and solvents were used as received. *Cis*-[Ru(bpy)$_2$(CN)$_2$] and *cis*-[Ru(^{t}Bu$_2$bpy)$_2$(CN)$_2$] were prepared following literature procedures.[1, 2]

Materials

[Ru(bpy)$_2$(MeNC)$_2$](PF$_6$)$_2$

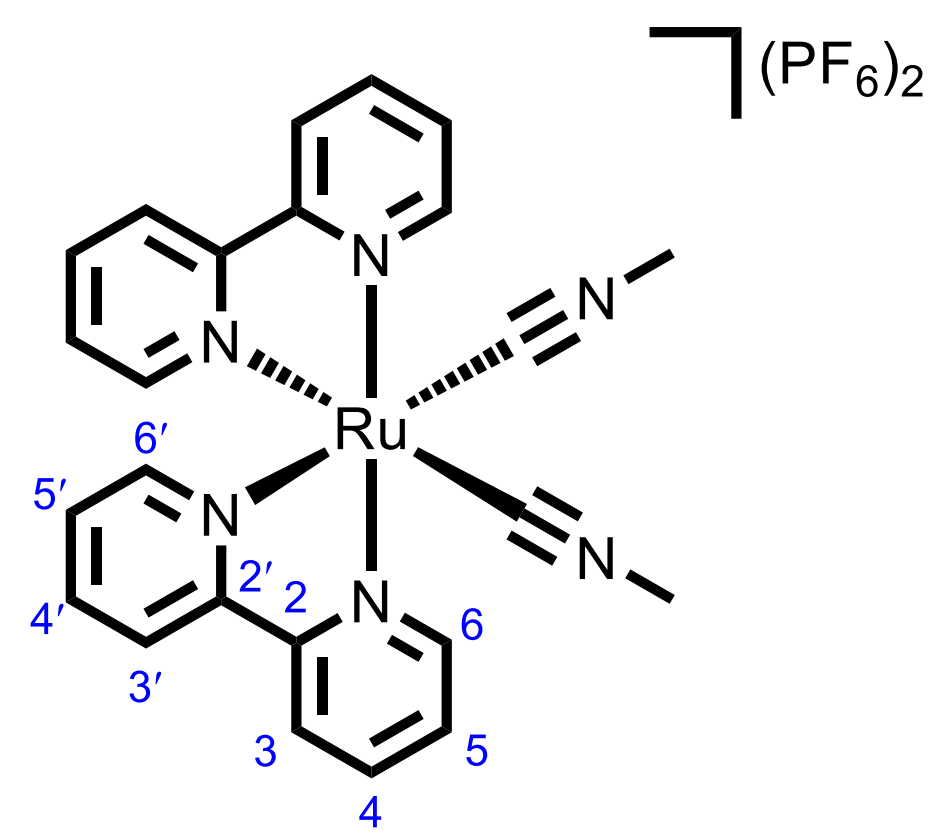

A suspension of [Ru(bpy)$_2$(CN)$_2$] (0.860 g, 1.85 mmol) in dimethyl sulfate (20 mL, 211 mmol) was heated at 70 ºC for 2 h. The resulting yellow solution was allowed to cool, extracted with water, and the product precipitated through addition of aq. KPF$_6$ (sat.). The product was filtered, then washed with copious amounts of water, 40:60 petroleum ether (to remove residual dimethyl sulfate) and diethyl ether. The product was further dried at 45 ºC under high vacuum for 6 h to afford the title compound (0.812 g, 56%) as a yellow solid. Single crystals for x-ray diffraction were grown by slow vapor diffusion of diethyl ether into an approximately 5 mM solution of the title compound in acetonitrile.

^{1}H NMR (400 MHz, CD$_3$CN): δ 9.17 (d, J = 5.8 Hz, 2H, H$_6$), 8.50 (d, J = 8.2 Hz, 2H, H$_3$), 8.41 (d, J = 8.2 Hz, 2H, H$_{3'}$), 8.28 (td, J = 8.0, 1.6 Hz, 2H, H$_4$), 8.07 (td, J = 8.0, 1.6 Hz, 2H, H$_{4'}$), 7.77 (ddd, J = 7.7, 5.6, 1.3 Hz, 2H, H$_5$), 7.49 (d, J = 5.5 Hz, 2H, H$_{6'}$), 7.38 (ddd, J = 7.7, 5.6, 1.2 Hz, 2H, H$_{5'}$), 3.44 (s, 6H, *Me*NC) ppm.

^{13}C NMR (101 MHz, CD$_3$CN): δ 157.3 (C$_2$), 156.7 (C$_6$), 156.4 (C$_{2'}$), 150.5 (C$_{6'}$), 146.1 (MeN*C*), 140.6 (C$_{4'}$), 140.2 (C$_4$), 129.0 (C$_5$), 128.4 (C$_{5'}$), 125.5 (C$_3$), 124.8 (C$_{3'}$), 31.30 (*Me*NC) ppm.

^{31}P NMR (162 MHz, CD_3CN): δ -144.6 (sept, J = 707 Hz, PF_6) ppm.

^{19}F NMR (376 MHz, CD_3CN): δ -72.8 (sept, J = 707 Hz, PF_6) ppm.

HRMS (ESI) calcd for $C_{24}H_{22}N_6RuPF_6$ ($[M+PF_6]^+$): m/z 641.0591. Found: m/z 641.0605.

Elemental analysis calcd for $C_{24}H_{22}F_{12}N_6P_2Ru \cdot H_2O$: C, 35.88; H, 3.01; N, 10.46. Found: C, 35.77; H, 2.79, N, 10.25.

Crystal data: $C_{24}H_{22}N_6Ru \cdot 2PF_6$, M = 785.48, yellow plate, 0.084 × 0.065 × 0.043 mm^3, monoclinic, a = 16.1906(2) Å, b = 10.45860(10) Å, c = 17.2888(2) Å, α = 90º, β = 96.6640(10)º, γ = 90º, V = 2907.75(6) Å^3, space group $P2_1/n$ (#14), Z = 4, μ(Cu Kα) = 6.389 mm^{-1}, $2\theta_{max}$ = 148.636º, 27 288 reflections measured, 5 745 independent reflections (R_{int} = 0.0195). The final $R_1(F)$ = 0.0261 ($I > 2\sigma(I)$); 0.0297 (all data). The final $wR_2(F^2)$ = 0.0664 ($I > 2\sigma(I)$); 0.0674 (all data). GoF = 1.068. CCDC 2425385.

[Ru(^{t}Bu$_2$bpy)$_2$(MeNC)$_2$](PF$_6$)$_2$

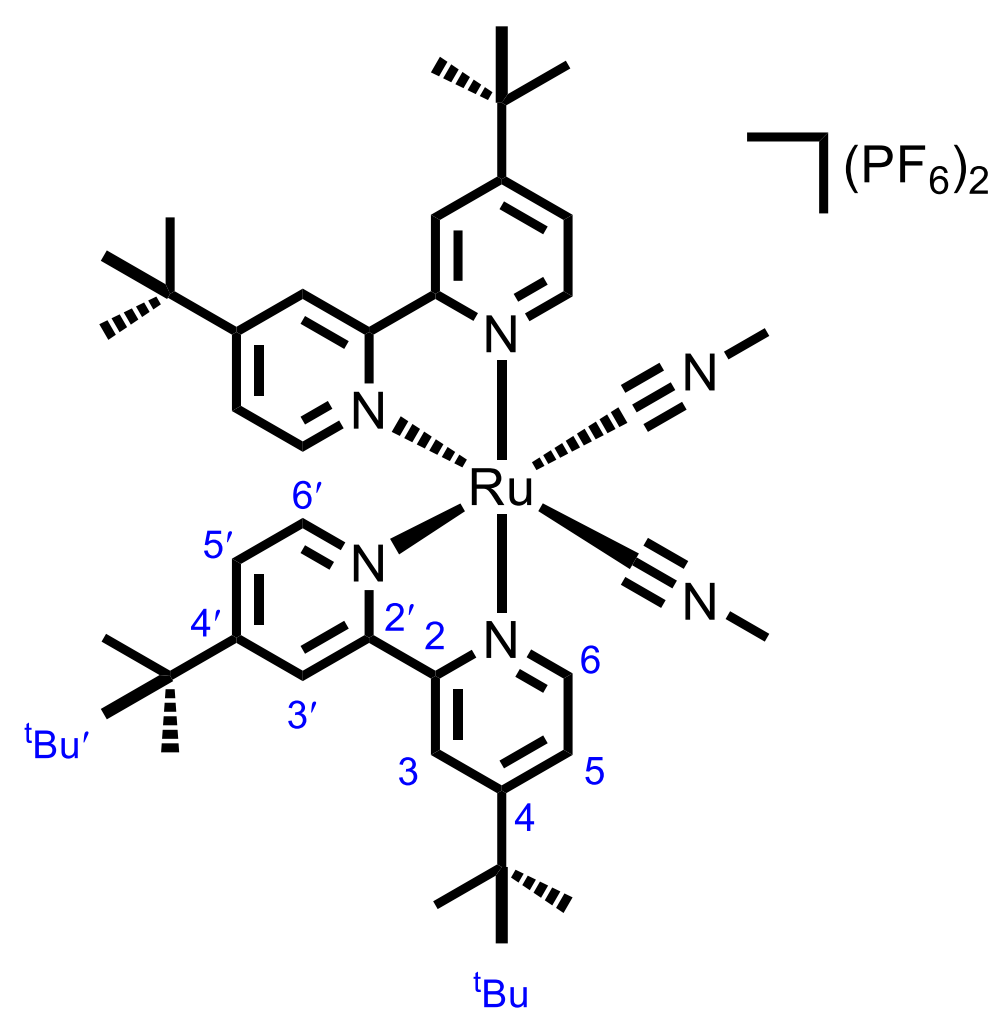

A suspension of [Ru(^{t}Bu$_2$bpy)$_2$(CN)$_2$] (1.00 g, 1.45 mmol) in dimethyl sulfate (25 mL) was heated at 70 ºC for 2 h under an Ar atmosphere. The resulting yellow solution was allowed to cool, extracted with water, and the product precipitated through addition of aq. KPF_6 (sat.). The product was filtered, then washed with copious amounts of water, 40:60 petroleum ether (to remove residual dimethyl sulfate) and diethyl ether. The product was further dried at 45 ºC under high vacuum for 6 h to afford the title compound (0.861 g, 59%) as a yellow solid. Single crystals for x-ray diffraction were grown by slow vapor diffusion of diethyl ether into an approximately 5 mM solution of the title compound in acetonitrile.

^{1}H NMR (400 MHz, CD_3CN): δ 9.01 (d, J = 6.0 Hz, 2H, H_6), 8.46 (d, J = 2.0 Hz, 2H, H_3), 8.37 (d, J = 1.7 Hz, 2H, $H_{3'}$), 7.75 (dd, J = 6.0, 2.1 Hz, 2H, H_5), 7.38 (dd, J = 6.0, 2.0 Hz, 2H, $H_{5'}$), 7.32 (d, J = 6.0 Hz, 2H, $H_{6'}$), 3.45 (s, 6H, *Me*NC), 1.53 (s, 18H, ^{t}Bu), 1.35 (s, 18H, ^{t}Bu′) ppm.

^{13}C NMR (101 MHz, CD_3CN): δ 165.3 ($C_{4'}$), 165.1 (C_4), 157.1 (C_2), 156.3 ($C_{2'}$), 156.0 (C_6), 149.7($C_{6'}$), 147.2 (MeN*C*), 126.0 (C_5), 125.3 ($C_{5'}$), 122.7 (C_3), 122.0 ($C_{3'}$), 36.5 (^{t}Bu-*quart*/ ^{t}Bu′-*quart*), 36.4 (^{t}Bu-*quart*/ ^{t}Bu′-*quart*), 31.3 (*Me*NC), 30.5 (^{t}Bu/ ^{t}Bu′), 30.3 (^{t}Bu/ ^{t}Bu′) ppm.

^{31}P NMR (162 MHz, CD_3CN): δ -144.6 (sept, J = 707 Hz, *P*F_6) ppm.

^{19}F NMR (376 MHz, CD_3CN): δ -72.8 (sept, J = 707 Hz, P*F_6*) ppm.

HRMS (ESI) calcd for $C_{40}H_{54}N_6RuPF_6$ ($[M+PF_6]^+$): *m/z* 865.3095. Found: *m/z* 865.3099.

Elemental analysis was unsuccessful for [Ru($^{t}Bu_2$bpy)$_2$(MeNC)$_2$](PF_6)$_2$, yielding widely inconsistent elemental compositions from the same batch of compound.

Crystal data: $C_{40}H_{54}N_6Ru\cdot 2PF_6\cdot 0.5C_4H_{10}O\cdot 0.5C_2H_3N$, M = 1067.49, yellow prism, 0.23 × 0.14 × 0.1 mm^3, monoclinic, a = 13.64724(5) Å, b = 20.69306(7) Å, c = 17.36130(6) Å, α = 90°, β = 90.7063(3)°, γ = 90°, V = 4902.52(3) Å^3, space group P2$_1$/c (#14), Z = 4, μ(Cu Kα) = 3.950 mm^{-1}, $2\theta_{max}$ = 149.22°, 87 294 reflections measured, 9 806 independent reflections (R_{int} = 0.0298). The final $R_1(F)$ = 0.0313 ($I > 2\sigma(I)$); 0.0325 (all data). The final $wR_2(F^2)$ = 0.0797 ($I > 2\sigma(I)$); 0.0806 (all data). GoF = 1.040. CCDC 2425388.

[Ru(bpy)$_2$($C_4H_{10}N_4$)](PF_6)$_2$

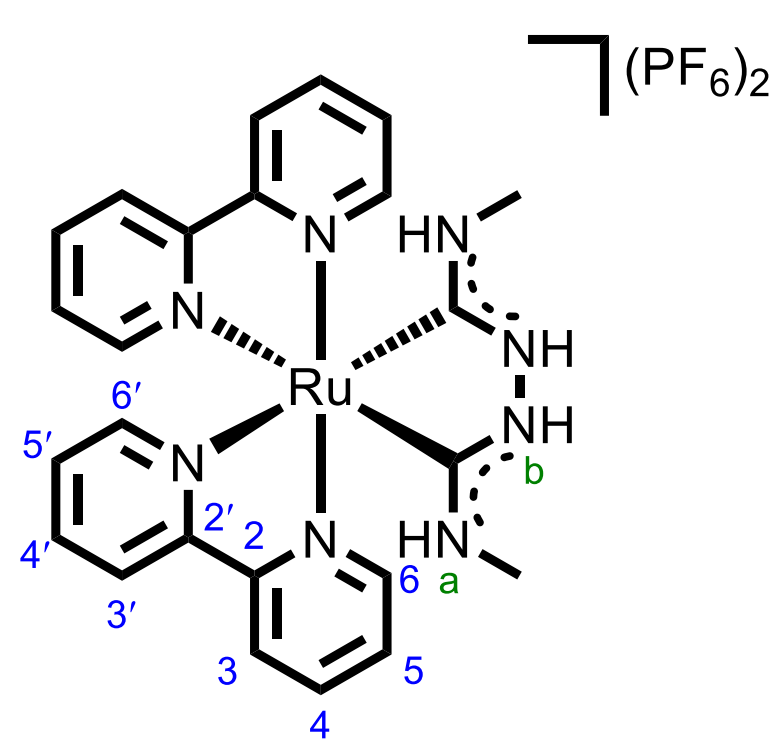

Hydrazine monohydrate (0.5 mL, 10.3 mmol) was added to a solution of [Ru(bpy)$_2$(MeNC)$_2$](PF_6)$_2$ (0.419 g, 0.534 mmol) in acetonitrile (10 mL). The reaction mixture was heated at reflux under an Ar atmosphere for 2 h. The resultant orange solution was allowed to cool, and the product precipitated through addition of aq. KPF_6 (sat.). The product was filtered, then washed with copious amounts of water, then

diethyl ether. The product was further dried at 45 ºC under high vacuum for 6 h to afford the title compound (0.310 g, 71%) as an orange solid. Single crystals for x-ray diffraction were grown by slow vapor diffusion of diethyl ether into an approximately 5 mM solution of the title compound in acetonitrile.

^{1}H NMR (400 MHz, CD_3CN): δ 9.22 (s, 2H, NH_b), 8.41 (d, J = 8.2 Hz, 2H, H_3), 8.37 (d, J = 5.9 Hz, 2H, H_6), 8.35 (d, J = 8.3 Hz, 2H, $H_{3'}$), 8.06 (td, J = 8.0, 1.6 Hz, H_4), 7.95 (td, J = 7.9, 1.6 Hz, 2H, $H_{4'}$), 7.51 (ddd, J = 7.6,5.6,1.5 Hz, 2H, H_5), 7.46 (d, J = 5.4 Hz, 2H, $H_{6'}$), 7.30 (ddd, J = 7.7, 5.5, 1.4 Hz, 2H, $H_{5'}$), 6.03 (s, 2H, NH_a), 2.76 (d, J = 5.1 Hz, 6H, Me) ppm.

^{13}C NMR (101 MHz, CD_3CN): δ 216.0 (carbene), 157.6 (C_2), 155.5 ($C_{2'}$), 155.0 (C_6), 149.7 ($C_{6'}$), 138.5 ($C_{4'}$), 137.9 (C_4), 128.1 (C_5/$C_{5'}$), 128.0 (C_5/$C_{5'}$), 124.8 (C_3), 124.3 ($C_{3'}$), 30.6 (Me) ppm.

^{31}P NMR (162 MHz, CD_3CN): δ -144.6 (sept, J = 707 Hz, PF_6) ppm.

^{19}F NMR (376 MHz, CD_3CN): δ -72.8 (sept, J = 707 Hz, PF_6) ppm.

HRMS (ESI) calcd for $C_{24}H_{26}N_8Ru$ ($[M]^{2+}$): m/z 264.0662. Found: m/z 264.0657

Elemental analysis calcd for $C_{24}H_{26}F_{12}N_8P_2Ru \cdot H_2O$: C, 34.58; H, 3.14; N, 13.44. Found: C, 34.77; H, 3.16; N, 13.53.

Crystal data: $C_{24}H_{26}F_{12}N_8P_2Ru$, M = 817.54, orange plate, 0.235 × 0.171 × 0.023 mm^3, monoclinic, a = 17.2938(2) Å, b = 10.40560(10) Å, c = 16.8517(2) Å, α = 90º, β = 100.1600(10)º, γ = 90º, V = 2984.95(6) Å^3, space group C2/c (#15), Z = 4, μ(Cu Kα) = 6.271 mm^{-1}, $2\theta_{max}$ = 146.696º, 46 329 reflections measured, 2938 independent reflections (R_{int} = 0.0612). The final $R_1(F)$ = 0.0420 ($I > 2\sigma(I)$); 0.0425 (all data). The final $wR_2(F^2)$ = 0.1133 ($I > 2\sigma(I)$); 0.1137 (all data). GoF = 1.048. CCDC 2425386.

$[Ru(^tBu_2bpy)_2(C_4H_{10}N_4)](PF_6)_2$

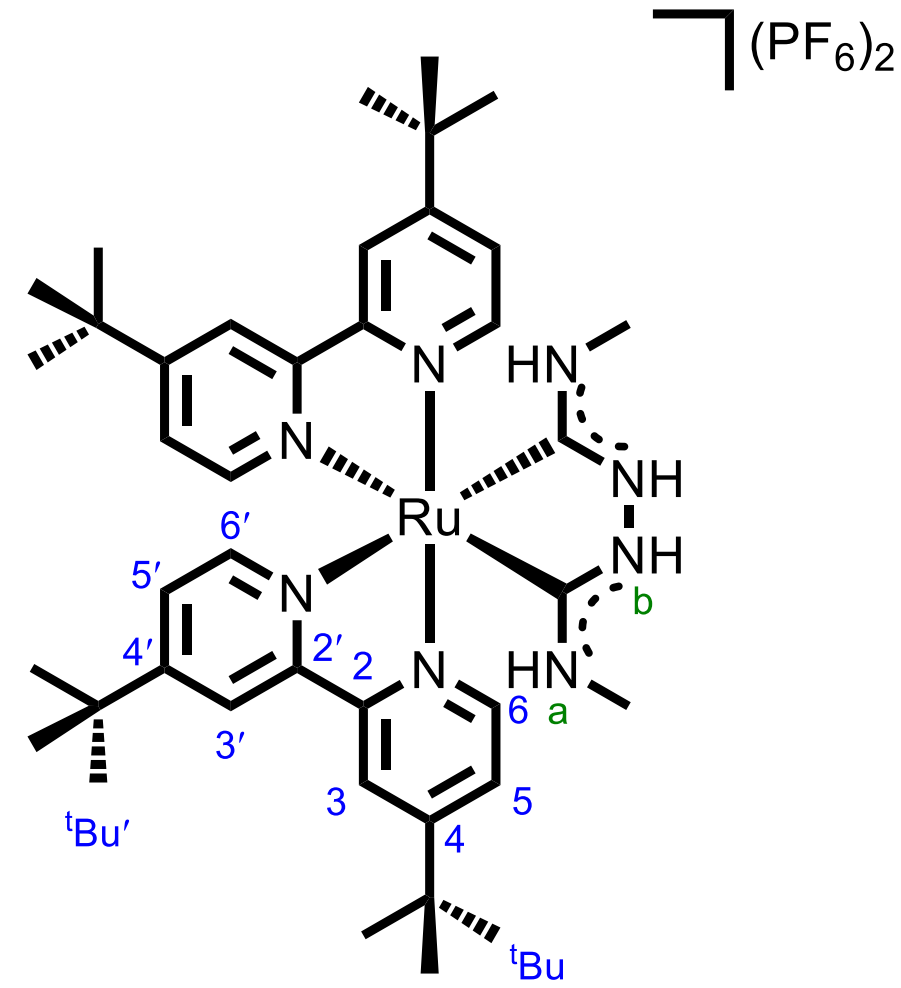

Hydrazine monohydrate (0.7 mL, 14.4 mmol) was added to a solution of $[Ru(^tBu_2bpy)_2(MeNC)_2](PF_6)_2$ (0.636 g, 0.629 mmol) in acetonitrile (10 mL). The reaction mixture was heated at reflux under an Ar atmosphere for 2 h. The resultant orange solution was allowed to cool, and the product precipitated through addition of aq. KPF_6 (sat.). The product was filtered, then washed with copious amounts of water, then diethyl ether. The product was further dried at 45 ºC under high vacuum for 6 h to afford the title compound (0.538 g, 82%) as an orange solid. Single crystals for x-ray diffraction were grown by slow vapor diffusion of diethyl ether into an approximately 5 mM solution of the title compound in acetonitrile.

^{1}H NMR (400 MHz, CD_3CN): δ 9.19 (s, 2H, NH_b), 8.38 (d, J = 2.1 Hz, 2H, H_3), 8.33 (s, 2H, $H_{3'}$), 8.24 (d, J = 6.0 Hz, 2H, H_6), 7.52 (dd, J = 6.0, 2.1 Hz, 2H, H_5), 7.32 (4H, m, $H_{5',6'}$), 5.94 (q, J = 5.3 Hz, 2H, NH_a), 2.80 (d, J = 5.1 Hz, 6H, Me), 1.48 (s, 18H, ^{t}Bu), 1.35 (s, 18H, ^{t}Bu′) ppm.

^{13}C NMR (101 MHz, CD_3CN): δ 216.9 (carbene), 162.9 ($C_{4'}$), 162.5 (C_4), 157.4 (C_2), 155.3 ($C_{2'}$), 154.4 (C_6), 149.0 ($C_{6'}$), 125.2 ($C_{5/5'}$), 125.1 ($C_{5/5'}$), 121.8 (C_3), 121.3 ($C_{3'}$), 36.3 (^{t}Bu-*quart*/ ^{t}Bu′-*quart*), 36.2 (^{t}Bu-*quart*/ ^{t}Bu′-*quart*), 30.7 (Me), 30.53 (^{t}Bu/ ^{t}Bu′), 30.48 (^{t}Bu/ ^{t}Bu′) ppm.

^{31}P NMR (162 MHz, CD_3CN): δ -144.6 (sept, J = 707 Hz, PF_6) ppm.

^{19}F NMR (376 MHz, CD_3CN): δ -72.8 (sept, J = 707 Hz, PF_6) ppm.

HRMS (ESI) calcd for $C_{40}H_{58}N_8Ru$ ($[M]^{2+}$): *m/z* 376.1914. Found: *m/z* 376.1954.

Elemental analysis calcd for $C_{40}H_{58}F_{12}N_8P_2Ru\cdot H_2O$: C, 45.33; H, 5.70; N, 10.57. Found: C, 45.17; H, 5.73; N, 10.50.

Crystal data: $C_{46}H_{69}F_{12}N_{10}OP_2Ru$, M = 1169.12, orange prism, 0.22 × 0.15 × 0.14 mm^3, monoclinic, a = 21.6383(4) Å, b = 11.9032(2) Å, c = 21.9571(4) Å, α = 90°, β = 91.0639(16)°, γ = 90°, V = 5654.39(17) $Å^3$, space group I2/a (#15), Z = 4, μ(Cu Kα) = 3.498 mm^{-1}, $2\theta_{max}$ = 149.468°, 53 883 reflections measured, 5680 independent reflections (R_{int} = 0.0569). The final $R_1(F)$ = 0.0484 ($I > 2\sigma(I)$); 0.0487 (all data). The final $wR_2(F^2)$ = 0.1349 ($I > 2\sigma(I)$); 0.1351 (all data). GoF = 1.087. CCDC 2425387.

Physical and Spectroscopic Measurements

NMR and Analytical Measurements

^1H, ^{13}C, ^{19}F and ^{31}P NMR spectra were recorded at 293 K in 5 mm diameter tubes on a Bruker Avance 400 spectrometer. Chemical shifts were referenced internally to residual non-perdeuterated solvent using δ values as originally reported by Gottlieb *et al.*[3-5] Coupling constants are rounded to the nearest 0.1 Hz. Assignment of signals is assisted through the use of 2D NMR techniques (COSY, NOESY, ^1H-^{13}C HSQC and ^1H-^{13}C HMBC) using standard pulse sequences. ^1H NMR spectra recorded for photolysis experiments were recorded at 293 K in 5 mm diameter tubes on a Bruker AV300 spectrometer. The residual non-perdeuterated solvent was used as an internal reference.

Pneumatically-assisted electrospray (ESI) mass spectra were recorded on an API 150EX (AB/MDS Sciex) equipped with a Turbo Ionspray source.

Elemental analyses were performed by Ms. Kerry-Lee Buchwalder from the Microchemical Laboratory of the University of Geneva.

X-ray structure analyses

Suitable crystals were selected, mounted on a cryoloop under protection oil and X-ray intensity data were collected on a Rigaku XtaLAB Synergy, Dualflex, HyPix-Arc 150° diffractometer using Cu Kα radiation (λ = 1.54184 Å).

Using Olex2,[6] the structures were solved with the SHELXT structure solution program[7] using dual space methods and refined with the SHELXL[8] refinement package using Least Squares minimization. Summaries of crystal data and structure refinement are given in Tables S1-4.

CCDC 2425385-2425388 contain the supplementary crystallographic data. These data can be obtained free of charge from the Cambridge Crystallographic Data Centre via www.ccdc.cam.ac.uk/.

Electrochemistry

The electrochemical cell for electrochemical measurements was comprised of a platinum disk working electrode, with a platinum wire counter electrode and a saturated calomel electrode (SCE) reference. The potential of the cell was controlled by a Metrohm DropSens potentiostat. Solutions were typically 1 mM in acetonitrile with 0.1 M tetrabutylammonium hexafluorophosphate (Bu_4NPF_6) as supporting electrolyte. Solutions were purged with argon for approximately 5 min prior to, and kept under a gentle flow of argon during measurement.

Optical Spectroscopy

Steady-state electronic absorption spectra were recorded as acetonitrile solutions in 1 cm pathlength quartz cuvettes on a Cary 5000 UV-Vis-NIR spectrometer from Varian.

Steady-state emission spectra were recorded as solutions at room temperature in 1 cm pathlength quartz cuvettes using a Fluorolog 3-222 spectrometer from Horiba Jobin-Yvon equipped with a Peltier-cooled R13456 photomultiplier tube. Measurements recorded in frozen solvent glass were recorded in 5 mm diameter quartz EPR tubes immersed in a quartz liquid nitrogen dewar.

Variable temperature emission spectra were recorded as solutions in 1 cm pathlength quartz cuvettes using a JASCO FP-8600 NIR spectrofluorometer equipped with an Oxford Instruments OptistatDN cryostat.

Room temperature time-correlated single photon counting (TCSPC) was performed using a modified version of a previously described setup.[9] Samples were de-aerated by bubbling with argon for 15 min in PTFE seal-capped cuvettes prior to measurement, with optical densities at the excitation wavelength of 0.1

or lower. Samples were then excited at 440 nm using a PicoQuant LD-440 (1 MHz repetition rate). Neutral density filters were used to tune the detector count rate to between 1-2% of the laser repetition rate. Emitted light was collected using a Cassegrain-type collection optic, passed through a 532 nm long-pass filter and detected using a single-photon avalanche photodiode (Micro Photon Devices, MPD-100-CTE). The time-correlated single photon counting was performed with a PicoHarp-300 (PicoQuant).

For variable temperature TCSPC, pulsed excitation light was provided by a SuperK Extreme (NKT) supercontinuum laser with a repetition rate of 614 kHz for the Chugaev-type dicarbene complexes and 307 kHz for $[Ru(bpy)_3](PF_6)_2$, which outputs broadband light in the visible and near IR. A SuperK Select photo-acoustic module (also NKT) was used to select a narrow band of wavelengths, centered at 450 nm. Sample emission was passed through a 500 nm long-pass filter (ThorLabs FELH0500) to remove scattered laser light and was detected using an ID100 single photon detector (ID Quantique). An ID900 Time Controller was used to time pulses. Samples were de-aerated using three freeze-pump-thaw cycles in custom-built Schlenk cuvettes (1 cm pathlength). Temperature control was achieved using a Quantum Northwest qpod temperature-controlled cuvette holder.

X-Ray Spectroscopy

Ru 2p3d and 2p4d RIXS measurements were collected at beamline 6-2a at the Stanford Synchrotron Radiation Lightsource (SSRL). A liquid nitrogen cooled Si(111) monochromator delivered an incident beam flux of $\sim 3 \times 10^{12}$ photons per s at 3.0 keV (vicinity of Ru L_3 absorption edge) with an energy resolution of about 400 meV and a beamsize with a full width half maximum (FWHM) of 400×250 μm^2 (v × h). The measurements were performed using a high-resolution under-vacuum Johansson-type spectrometer equipped with a cylindrically bent Si(111) analyzer with energy resolution of ~0.3 eV and a CCD camera detector, all enclosed in a vacuum chamber..[10] The energy calibration of the spectrometer was performed with elastic scattering measurements. The monochromator energy was calibrated using the published L_3 XAS spectrum of $[Ru(bpy)_3]^{2+}$.[11] The spectrometer CCD images were analyzed as described in reference [10]. Samples were mounted in a helium-filled sample chamber as 7 mm diameter pressed powder

pellets. For the 2p4d RIXS measurements, pellets were composed of 100% Ru complex, while for the 2p3d RIXS measurements, the Ru complex was diluted with sucrose to achieve a 2% Ru mass concentration. Radiation damage was carefully monitored and avoided by collecting spectra at multiple positions across the pellets. On average, 2p3d RIXS maps were acquired by summing data from different positions, with a total exposure time of 15 seconds per incident energy point, across 110 energy points. Each 2p4d RIXS spectrum was similarly obtained by summing measurements from multiple positions, with a total acquisition time of approximately 30 minutes. XAS spectra were extracted by projecting the full 2p3d RIXS maps (shown in Figure S26) along the incident energy axis to obtain partial fluorescence yield (PFY) XAS. High energy resolution fluorescence detection (HERFD) XAS spectra were derived by integrating over a narrow energy window (~0.75 eV) centered on the peak of the non-resonant $L\alpha_1$ emission line, as shown in Figure S27. Narrower integration windows did not improve spectral resolution.

Data were processed and analyzed using home-written Python scripts. Data fitting was performed using nonlinear least squares as implemented in the SciPy library[12]. Second derivative spectra were obtained using the Savitzky-Golay method as implemented in the SciPy library. Window sizes and polynomial orders were optimized to enhance signal clarity without loss of information. Typical windows of 31 data points were used for XAS and 41 for RIXS second derivatives, both with 7th order polynomials. Singular value decomposition was performed as implemented in the NumPy library.[13]

Computational Details

All calculations were performed using ORCA (version 6.0.1 with the aid of OpenMPI 4.1.5),[14] accessed through the eResearch Infrastructure Platform hosted by the New Zealand Crown company, Research and Education Advanced Network New Zealand (REANNZ) Ltd.

All calculations were performed using density functional theory (DFT) employing the B3LYP functional[15-18] with the RIJCOSX approximation,[19, 20] Grimme's D3-BJ dispersion correction,[21, 22] and the def2-tzvp(-f)

basis set[23, 24] with the def2/J auxiliary basis set[25-27] in a conductor-like polarised continuum solvation model (SMD, solvent = acetonitrile).[28, 29]

Time-dependent DFT (TD-DFT) calculations were performed on geometry-optimised structures. Molecular orbital plots were generated using GabEdit (version 2.5.1).[30]

X-Ray Crystallography

Table S1. Crystal data and structure refinement for $[Ru(bpy)_2(MeNC)_2](PF_6)_2$.

**CCDC number**	**2425385**	
Empirical formula	C24 H22 F12 N6 P2 Ru	
Formula weight	785.48	
Temperature	119.9(2) K	
Wavelength	1.54184 Å	
Crystal system	Monoclinic	
Space group	P 2_1/n	
Unit cell dimensions	a = 16.1906(2) Å	α= 90°
	b = 10.45860(10) Å	β= 96.6640(10)°
	c = 17.2888(2) Å	γ = 90°
Volume	2907.75(6) Å^3	
Z	4	
Density (calculated)	1.794 Mg/m^3	
Absorption coefficient	6.389 mm^{-1}	
F(000)	1560	
Crystal size	0.084 x 0.065 x 0.043 mm^3	
Theta range for data collection	3.541 to 74.318°	
Index ranges	-18<=h<=20, -12<=k<=12, -21<=l<=21	
Reflections collected	27288	
Independent reflections	5745 [R(int) = 0.0195]	
Completeness to theta = 67.684°	99.9 %	
Absorption correction	Gaussian	
Max. and min. transmission	0.877 and 0.670	
Refinement method	Full-matrix least-squares on F^2	
Data / restraints / parameters	5745 / 0 / 408	
Goodness-of-fit on F^2	1.068	
Final R indices [I>2sigma(I)]	R1 = 0.0261, wR2 = 0.0664	
R indices (all data)	R1 = 0.0297, wR2 = 0.0674	
Largest diff. peak and hole	1.075 and -0.561 e.Å^{-3}	

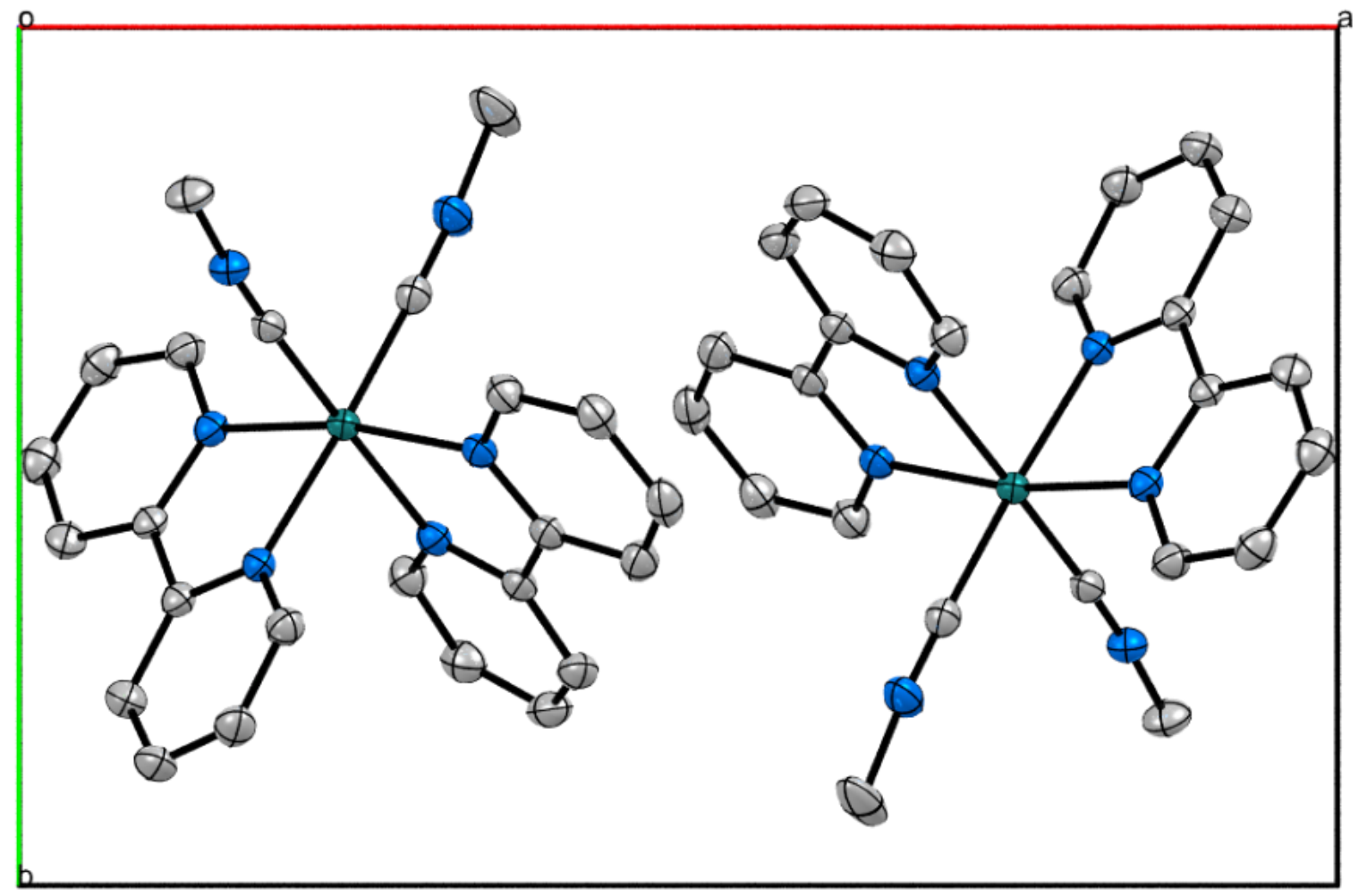

Figure S1. Unit cell from single crystal X-ray diffraction of $[Ru(bpy)_2(MeNC)_2](PF_6)_2$, viewed along the *c* axis. Ellipsoids are depicted at the 50% probability level. Hydrogen atoms, PF_6^- counterion and co-crystallized solvent molecules are omitted for clarity.

Table S2. Crystal data and structure refinement for $[Ru(bpy)_2(C_4H_{10}N_4)](PF_6)_2$.

**CCDC Number**	**2425386**	
Empirical formula	C24 H26 F12 N8 P2 Ru	
Formula weight	817.54	
Temperature	100.00(10) K	
Wavelength	1.54184 Å	
Crystal system	Monoclinic	
Space group	C 2/c	
Unit cell dimensions	a = 17.2938(2) Å	α= 90°
	b = 10.40560(10) Å	β= 100.1600(10)°
	c = 16.8517(2) Å	γ = 90°
Volume	2984.95(6) $Å^3$	
Z	4	
Density (calculated)	1.819 Mg/m^3	
Absorption coefficient	6.271 mm^{-1}	
F(000)	1632	
Crystal size	0.235 x 0.171 x 0.023 mm^3	
Theta range for data collection	4.981 to 73.348°.	
Index ranges	-21<=h<=21, -11<=k<=12, -20<=l<=20	
Reflections collected	46329	
Independent reflections	2938 [R(int) = 0.0612]	

Completeness to theta = 67.684°	100.0 %
Absorption correction	Analytical
Max. and min. transmission	0.869 and 0.310
Refinement method	Full-matrix least-squares on F^2
Data / restraints / parameters	2938 / 2 / 220
Goodness-of-fit on F^2	1.048
Final R indices [I>2sigma(I)]	R1 = 0.0420, wR2 = 0.1133
R indices (all data)	R1 = 0.0425, wR2 = 0.1137
Largest diff. peak and hole	0.780 and -1.378 e.Å^{-3}

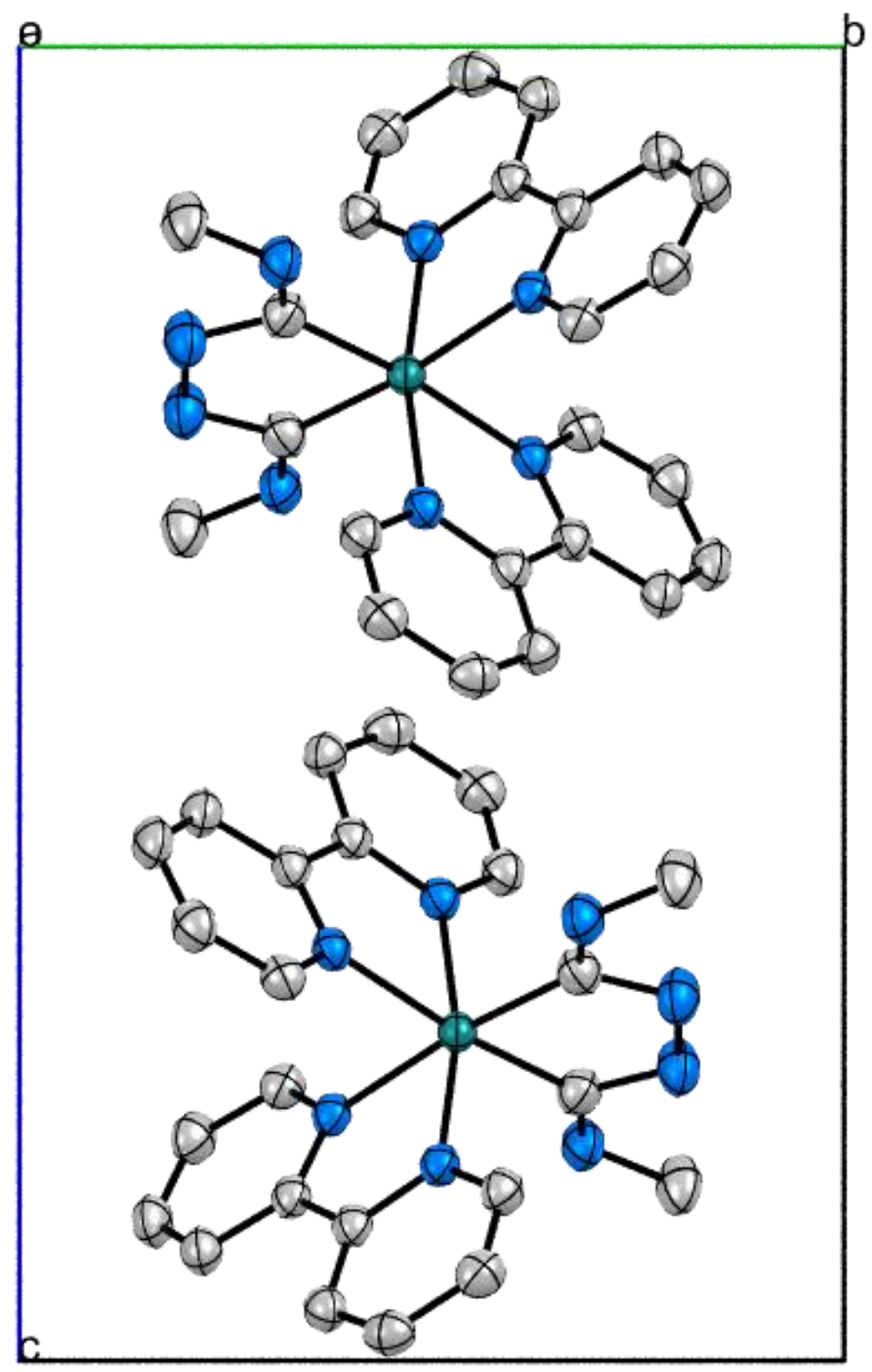

Figure S2. Unit cell from single crystal X-ray diffraction of $[Ru(bpy)_2(C_4H_{10}N_4)](PF_6)_2$, viewed along the a axis. Ellipsoids are depicted at the 50% probability level. Hydrogen atoms, PF_6^- counterion and co-crystallized solvent molecules are omitted for clarity.

Table S3. Crystal data and structure refinement for $[Ru(dtbbpy)_2(MeNC)_2](PF_6)_2$.

**CCDC Number**	**2425388**	
Empirical formula	C43 H60.50 F12 N6.50 O0.50 P2 Ru	
Formula weight	1067.49	
Temperature	99.98(10) K	
Wavelength	1.54184 Å	
Crystal system	Monoclinic	
Space group	P 2_1/c	
Unit cell dimensions	a = 13.64724(5) Å	α= 90°.
	b = 20.69306(7) Å	β= 90.7063(3)°.
	c = 17.36130(6) Å	γ = 90°.
Volume	4902.52(3) Å^3	
Z	4	
Density (calculated)	1.446 Mg/m^3	
Absorption coefficient	3.950 mm^{-1}	
F(000)	2200	
Crystal size	0.23 x 0.14 x 0.1 mm^3	
Theta range for data collection	3.239 to 74.610°.	
Index ranges	-16<=h<=16, -24<=k<=25, -21<=l<=20	
Reflections collected	87294	
Independent reflections	9806 [R(int) = 0.0298]	
Completeness to theta = 67.684°	100.0 %	
Absorption correction	Analytical	
Max. and min. transmission	0.733 and 0.470	
Refinement method	Full-matrix least-squares on F^2	
Data / restraints / parameters	9806 / 0 / 658	
Goodness-of-fit on F^2	1.040	
Final R indices [I>2sigma(I)]	R1 = 0.0313, wR2 = 0.0797	
R indices (all data)	R1 = 0.0325, wR2 = 0.0806	
Extinction coefficient	n/a	
Largest diff. peak and hole	0.863 and -0.652 e.Å^{-3}	

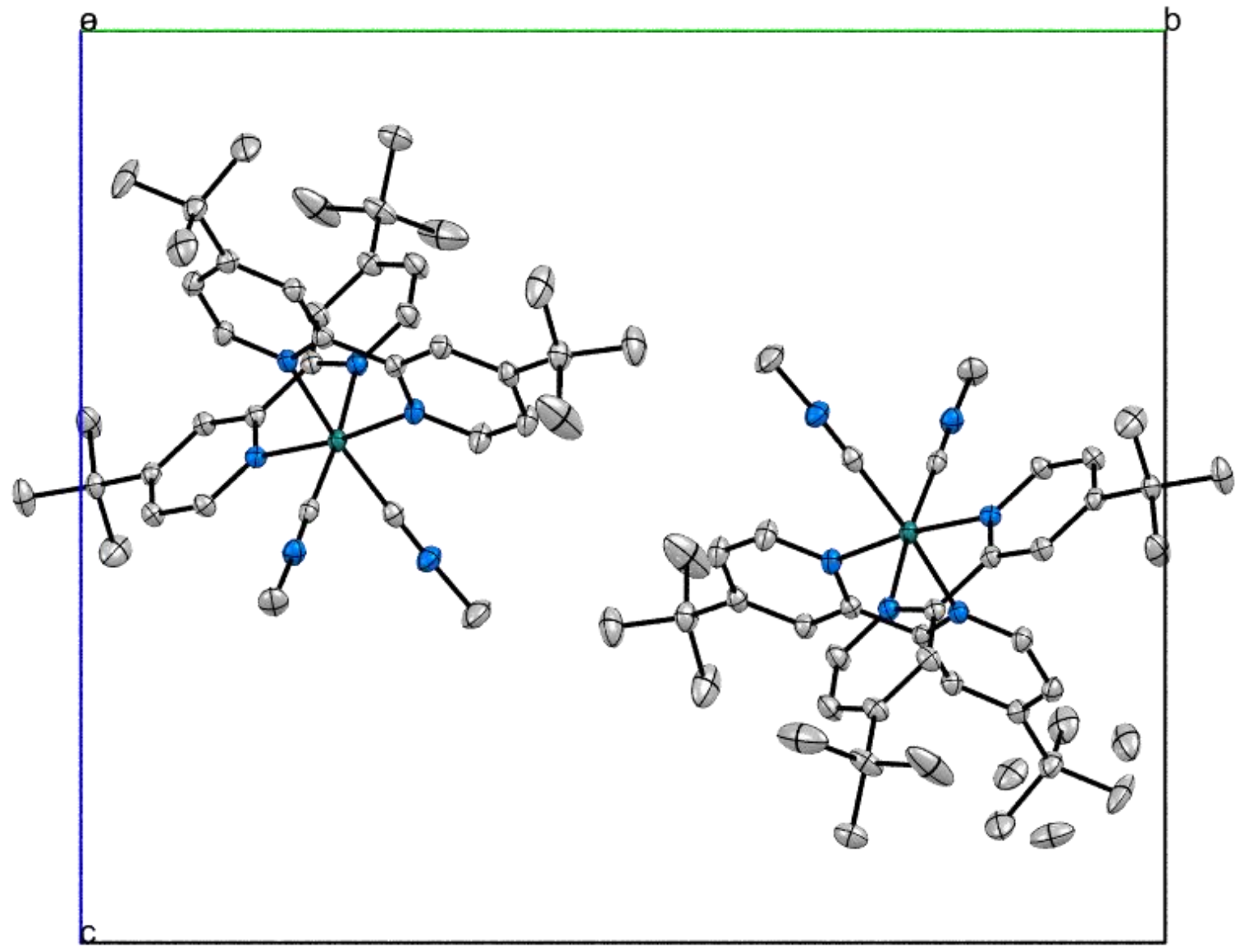

Figure S3. Unit cell from single crystal X-ray diffraction of $[Ru(dtbbpy)_2(MeNC)_2](PF_6)_2$, viewed along the *a* axis. Ellipsoids are depicted at the 50% probability level. Hydrogen atoms, PF_6^- counterion and co-crystallized solvent molecules are omitted for clarity.

Table S4. Crystal data and structure refinement for $[Ru(dtbbpy)_2(C_4H_{10}N_4)](PF_6)_2$.

**CCDC Number**	**2425387**	
Empirical formula	C46 H69 F12 N10 O P2 Ru	
Formula weight	1169.12	
Temperature	100.00(10) K	
Wavelength	1.54184 Å	
Crystal system	Monoclinic	
Space group	*I* 2/*a*	
Unit cell dimensions	a = 21.6383(4) Å	α= 90°.
	b = 11.9032(2) Å	β= 91.0639(16)°.
	c = 21.9571(4) Å	γ = 90°.
Volume	5654.39(17) $Å^3$	
Z	4	
Density (calculated)	1.373 Mg/m^3	
Absorption coefficient	3.498 mm^{-1}	
F(000)	2420	
Crystal size	0.22 x 0.15 x 0.14 mm^3	
Theta range for data collection	4.027 to 74.734°.	

Index ranges	-26<=h<=26, -14<=k<=14, -27<=l<=25
Reflections collected	53883
Independent reflections	5680 [R(int) = 0.0569]
Completeness to theta = 67.684°	100.0 %
Absorption correction	Analytical
Max. and min. transmission	0.702 and 0.612
Refinement method	Full-matrix least-squares on F^2
Data / restraints / parameters	5680 / 3 / 338
Goodness-of-fit on F^2	1.087
Final R indices [I>2sigma(I)]	R1 = 0.0484, wR2 = 0.1349
R indices (all data)	R1 = 0.0487, wR2 = 0.1351
Extinction coefficient	n/a
Largest diff. peak and hole	1.894 and -0.786 e.Å^{-3}

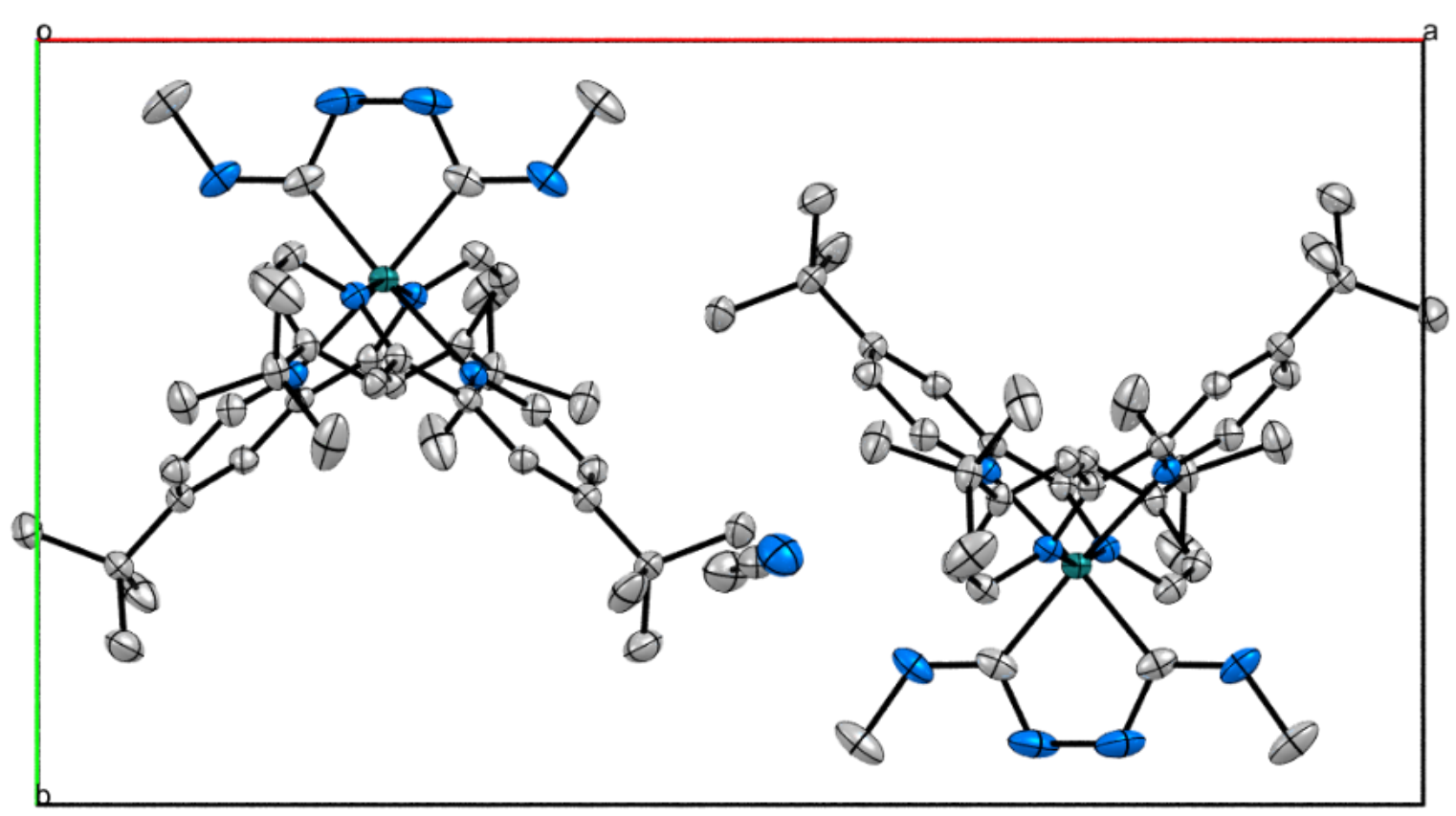

Figure S4. Unit cell from single crystal X-ray diffraction of [Ru(dtbbpy)$_2$($C_4H_{10}N_4$)](PF_6)$_2$, viewed along the *c* axis. Ellipsoids are depicted at the 50% probability level. Hydrogen atoms, PF_6^- counterion and co-crystallized solvent molecules are omitted for clarity.

Electrochemical Measurements

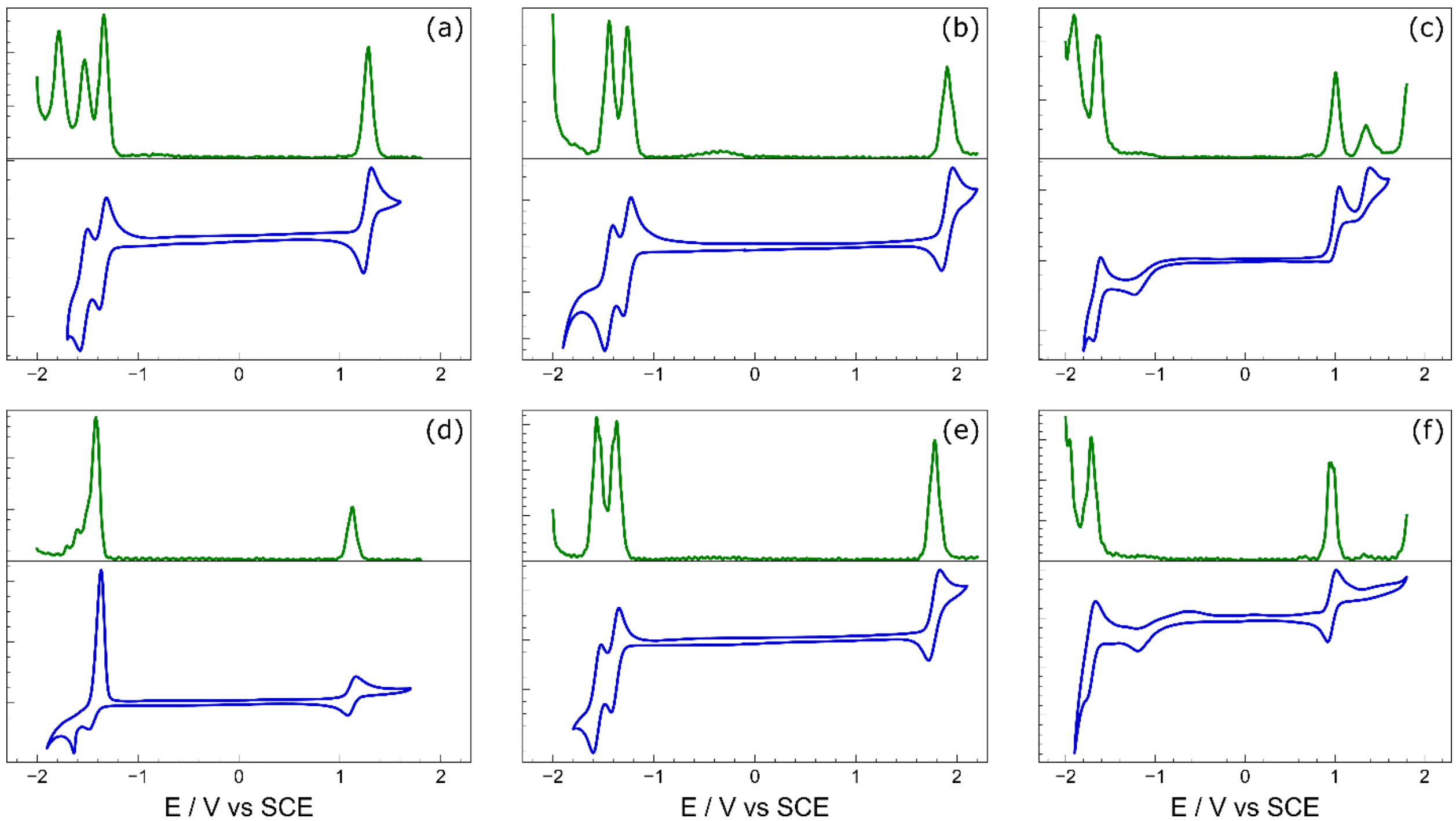

Figure S5. Cyclic (blue) and differential pulse (green) voltammograms for (a) $[Ru(bpy)_3](PF_6)_2$, (b) $[Ru(bpy)_2(MeNC)_2](PF_6)_2$, (c) $[Ru(bpy)_2(C_4H_{10}N_4)](PF_6)_2$, (d) $[Ru(dtbbpy)_3](PF_6)_2$, (e) $[Ru(dtbbpy)_2(MeNC)_2](PF_6)_2$, and (f) $[Ru(^tBu_2bpy)_2(C_4H_{10}N_4)](PF_6)_2$, recorded as ~1 mM solutions in acetonitrile with 100 mM $[Bu_4N]PF_6$ as electrolyte. The scan rate was 100 mV s^{-1} for cyclic voltammetry and 10 mV s^{-1} for differential pulse voltammetry.

Optical Spectroscopy

Molar Absorption Coefficient Determination

Molar absorption coefficients were determined by recording UV-vis spectra of a dilution series of the complex of interest in acetonitrile (Figure S6), which were then globally fitted to the Beer-Lambert equation:

$$A_\lambda = \varepsilon_\lambda \cdot c \cdot l \qquad Eq. S1$$

Specifically, the concentration-dependent absorption spectra were spectrally deconvolved using singular value decomposition (SVD):

$$A_{\lambda,c} = U_\lambda \cdot S \cdot {V_c}^T \qquad Eq. S2$$

Where $A_{\lambda,c}$ is the full data matrix containing the concentration-dependent absorption spectra, U_λ are the left singular vectors containing the spectral information (i.e. basis spectra), S are the singular values, and ${V_c}^T$ the right singular vectors containing the concentration-dependent information. It is evident from the singular values (Figure S7-Figure S12) that for every complex investigated, the data is best described by a single component that exhibits a linear concentration dependence, with all other components describing stochastic noise or artefacts.

The first right singular vector was then fitted to the Beer-Lambert equation using linear regression:

$${V_c^T}_i = a_\varepsilon \cdot c \cdot l \qquad Eq. S3$$

Where i is the index of the first right singular vector, a_ε is a fit coefficient representing a wavelength-independent molar absorption coefficient weighting factor. The molar absorption coefficients, ε_λ, are then obtained using the properly weighted basis spectrum through:

$$\varepsilon_\lambda = a_\varepsilon \cdot S_i \cdot U_{\lambda_i} \qquad Eq. S4$$

The resulting UV-vis spectra in molar absorption coefficient are presented in Figure 2 in the main paper.

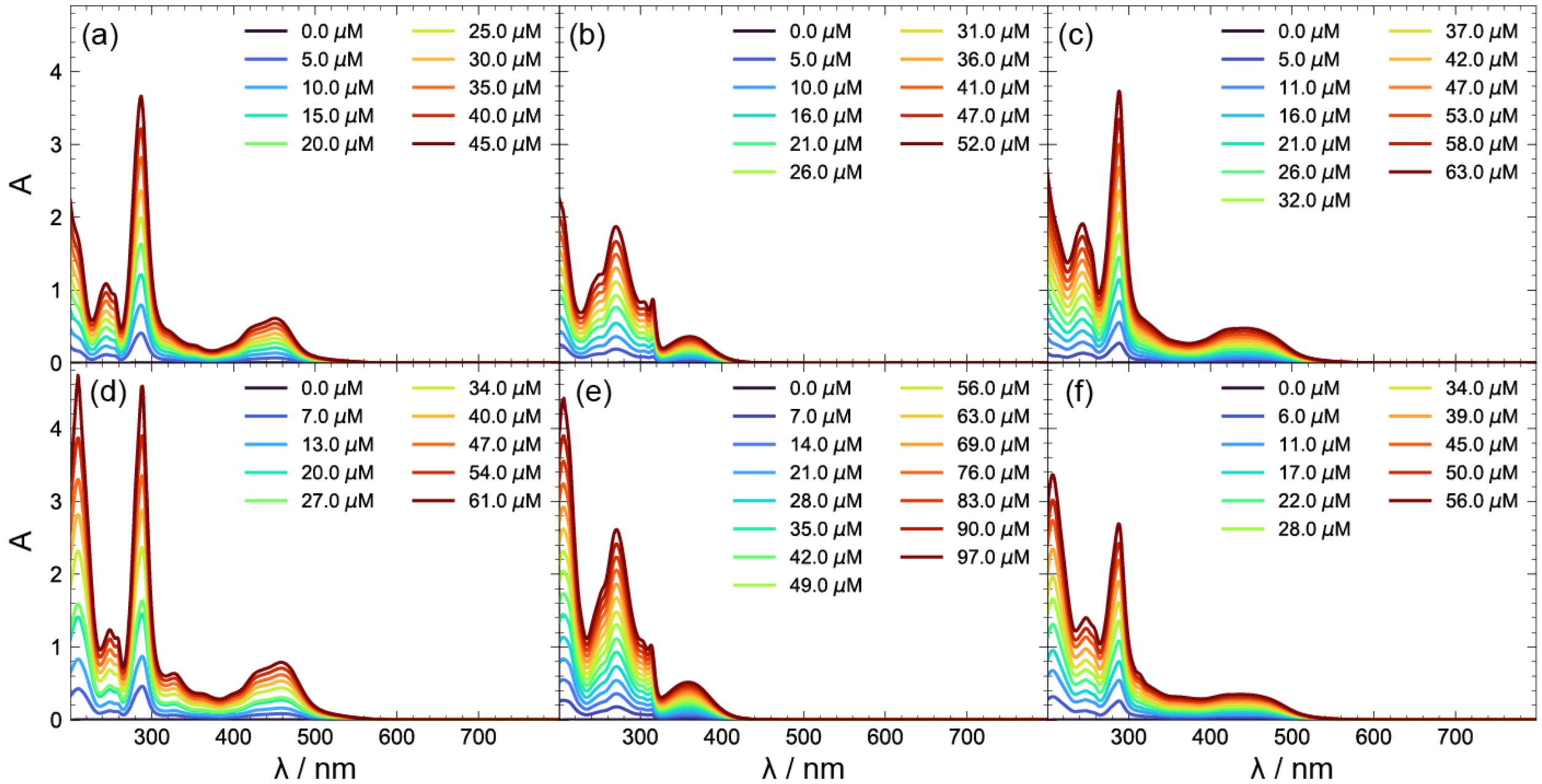

Figure S6. UV-vis spectra of (a) $[Ru(bpy)_3](PF_6)_2$, (b) $[Ru(bpy)_2(MeNC)_2](PF_6)_2$, (c) $[Ru(bpy)_2(C_4H_{10}N_4)](PF_6)_2$, (d) $[Ru(^tBu_2bpy)_3](PF_6)_2$, (e) $[Ru(^tBu_2bpy)_2(MeNC)_2](PF_6)_2$, and (f) $[Ru(^tBu_2bpy)_2(C_4H_{10}N_4)](PF_6)_2$ at various concentrations in acetonitrile.

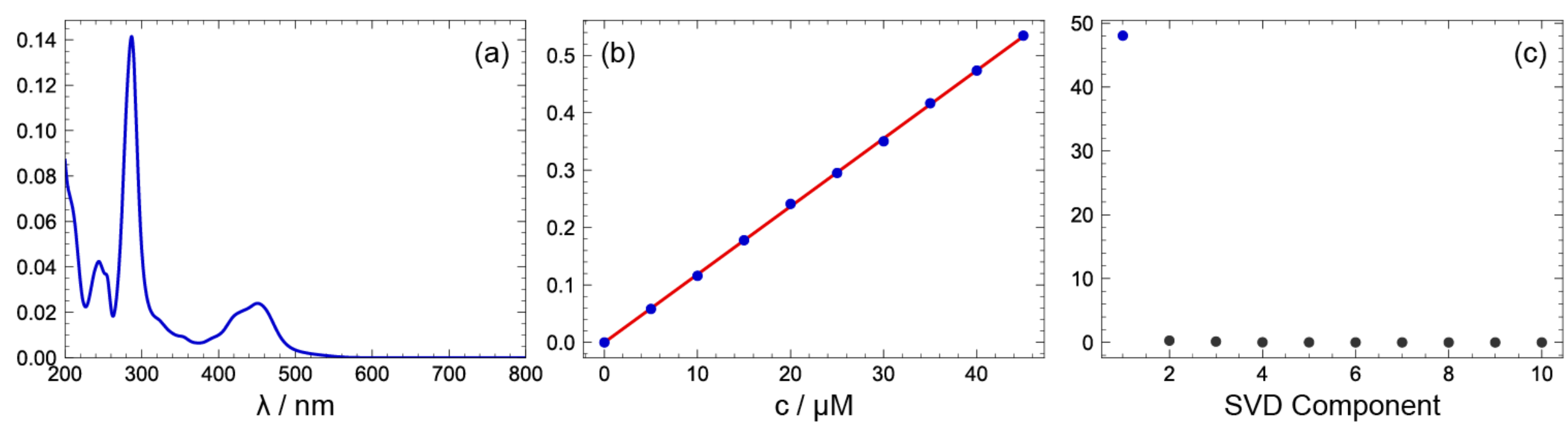

Figure S7. SVD and global fitting of the concentration-dependent UV-vis spectra of $[Ru(bpy)_3](PF_6)_2$ in acetonitrile (Figure S6, panel a). (a) First left singular vector (basis spectrum); (b) First right singular vector (blue) and linear regression fit to Eq. S3 (red); (c) singular values. The blue singular value in panel (c) corresponds to the blue left and right singular vectors in panels (a) and (b).

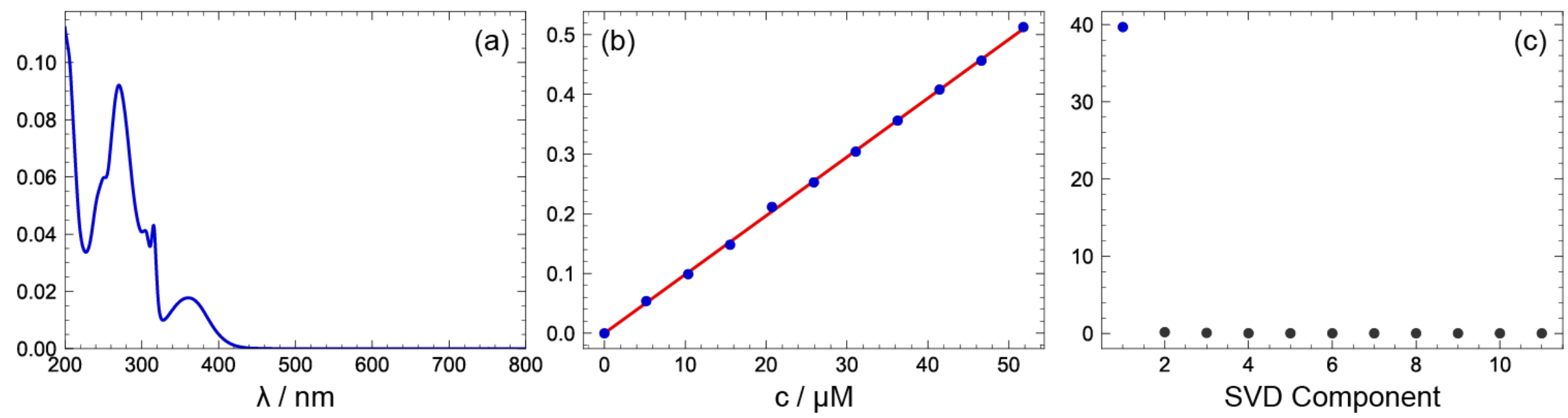

Figure S8. SVD and global fitting of the concentration-dependent UV-vis spectra of [Ru(bpy)$_2$(MeNC)$_2$](PF$_6$)$_2$ in acetonitrile (Figure S6, panel b). (a) First left singular vector (basis spectrum); (b) First right singular vector (blue) and linear regression fit to Eq. S3 (red); (c) singular values. The blue singular value in panel (c) corresponds to the blue left and right singular vectors in panels (a) and (b).

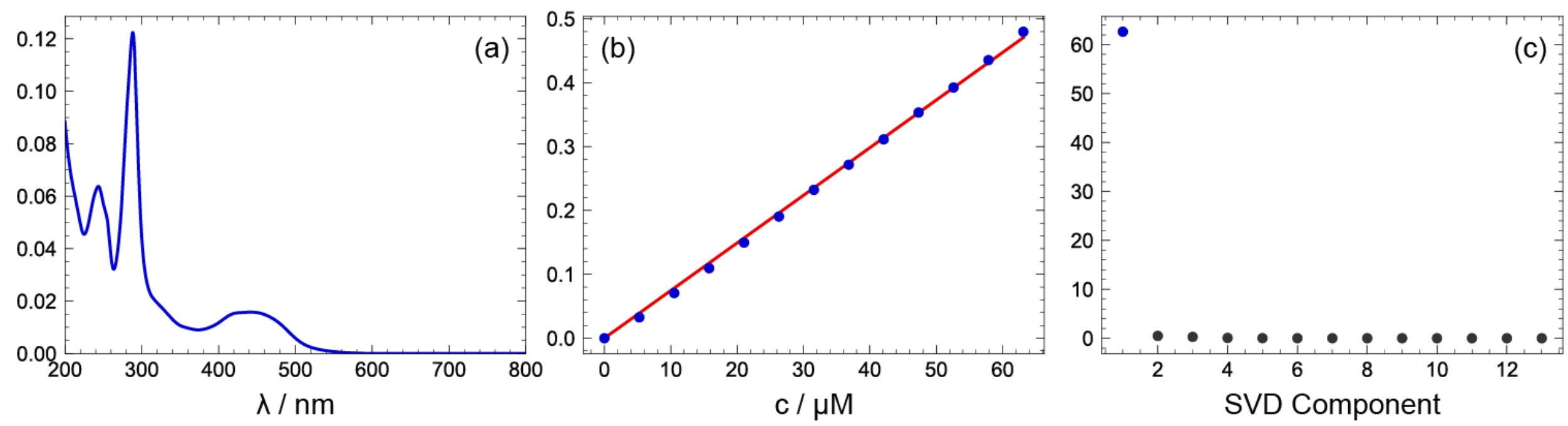

Figure S9. SVD and global fitting of the concentration-dependent UV-vis spectra of [Ru(bpy)$_2$(C$_4$H$_{10}$N$_4$)](PF$_6$)$_2$ in acetonitrile (Figure S6, panel c). (a) First left singular vector (basis spectrum); (b) First right singular vector (blue) and linear regression fit to Eq. S3 (red); (c) singular values. The blue singular value in panel (c) corresponds to the blue left and right singular vectors in panels (a) and (b).

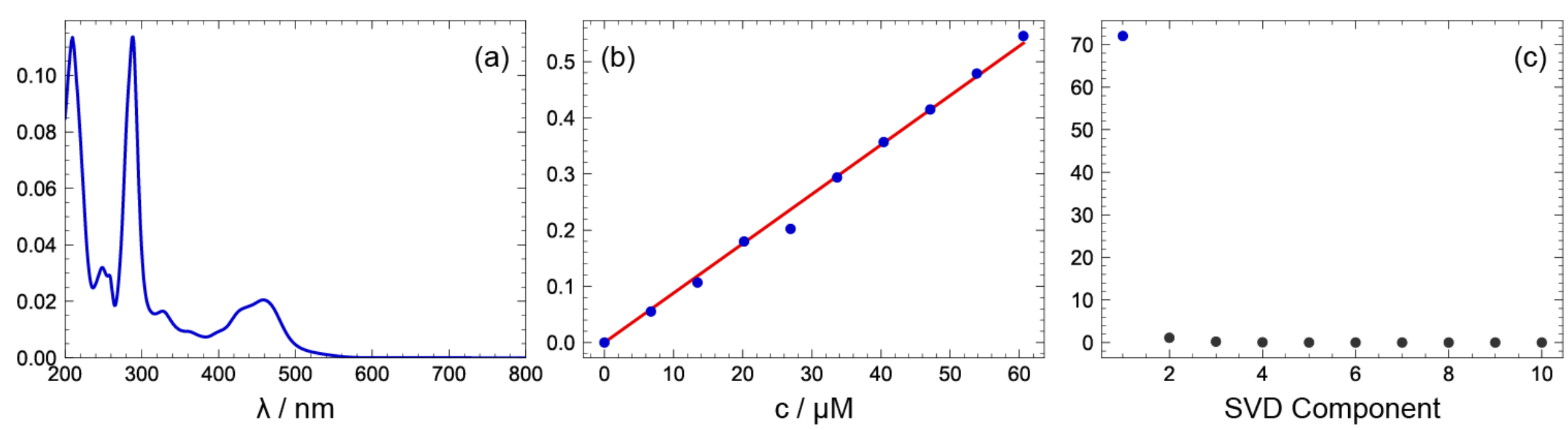

Figure S10. SVD and global fitting of the concentration-dependent UV-vis spectra of [Ru(^{t}Bu$_2$bpy)$_3$](PF$_6$)$_2$ in acetonitrile (Figure S6, panel d). (a) First left singular vector (basis spectrum); (b) First right singular vector (blue) and linear regression fit to Eq. S3 (red); (c) singular values. The blue singular value in panel (c) corresponds to the blue left and right singular vectors in panels (a) and (b).

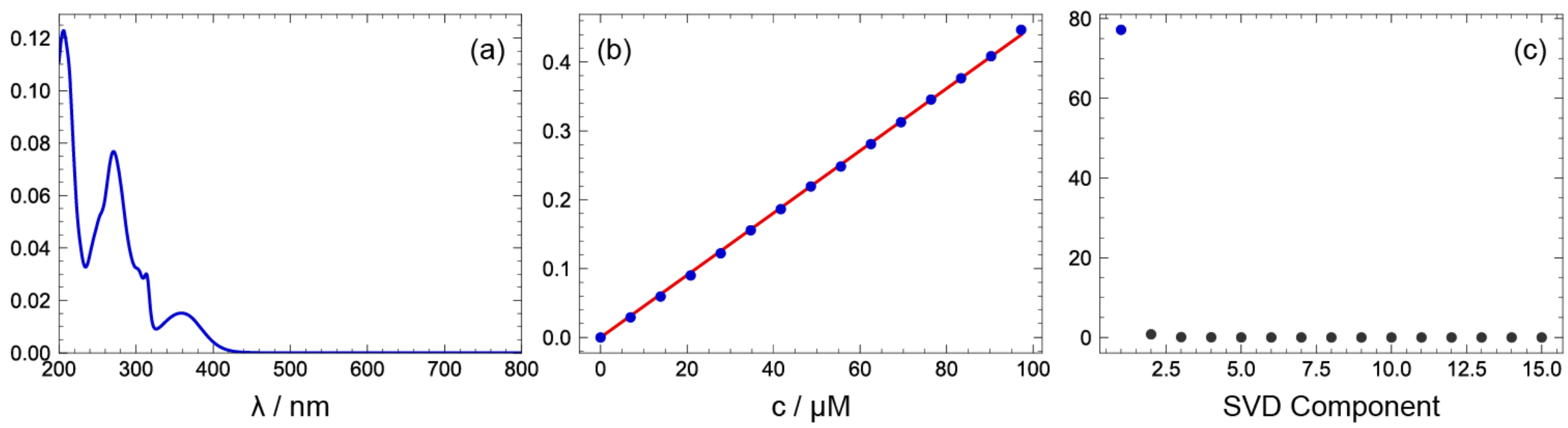

Figure S11. SVD and global fitting of the concentration-dependent UV-vis spectra of $[Ru(^tBu_2bpy)_2(MeNC)_2](PF_6)_2$ in acetonitrile (Figure S6, panel e). (a) First left singular vector (basis spectrum); (b) First right singular vector (blue) and linear regression fit to Eq. S3 (red); (c) singular values. The blue singular value in panel (c) corresponds to the blue left and right singular vectors in panels (a) and (b).

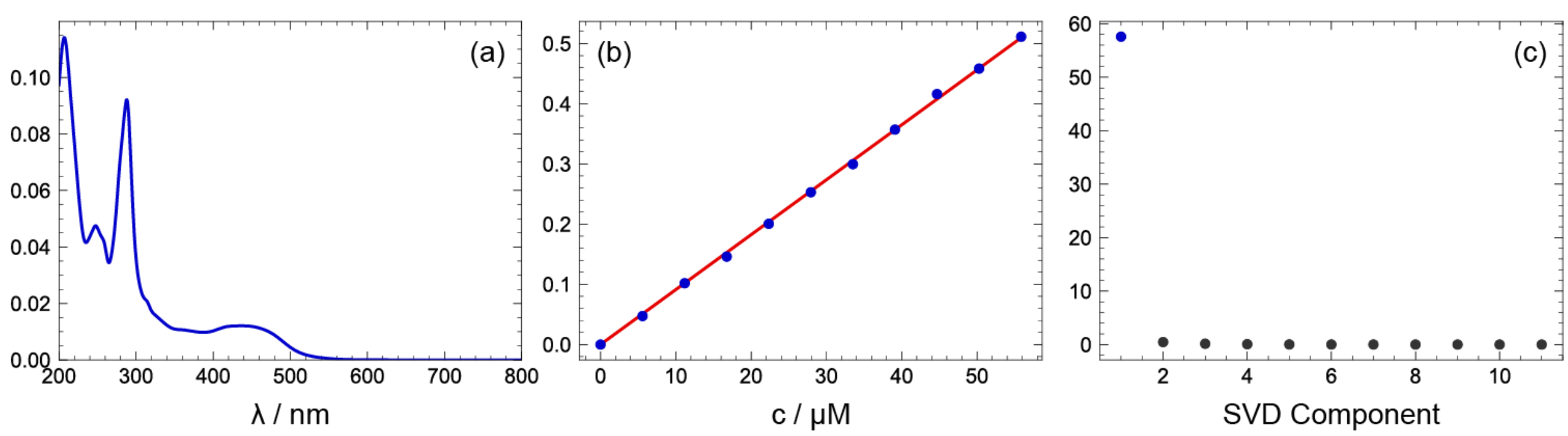

Figure S12. SVD and global fitting of the concentration-dependent UV-vis spectra of $[Ru(^tBu_2bpy)_2(C_4H_{10}N_4)](PF_6)_2$ in acetonitrile (Figure S6, panel f). (a) First left singular vector (basis spectrum); (b) First right singular vector (blue) and linear regression fit to Eq. S3 (red); (c) singular values. The blue singular value in panel (c) corresponds to the blue left and right singular vectors in panels (a) and (b).

Photoluminescence Quantum Yield Determination

Photoluminescence spectra for quantum yield determination were recorded as acetonitrile solutions. All samples were de-aerated by bubbling with argon for 15 min in PTFE seal-capped cuvettes prior to measurement, with optical densities at the excitation wavelength (450 nm) of 0.1 or lower.

Given the photosensitivity of the Chuagev-type dicarbene complexes, care was taken to prevent photodamage, including use of fresh sample for each measurement and careful optimization of instrument parameters to minimize light exposure. For all samples, spectra were recorded at 1 nm intervals, with an

excitation monochromator slitwidth of 5 nm, an emission monochromator slitwidth of 1 nm, and an integration time of 0.2 s. UV-vis spectra recorded before and after measurement confirmed negligible photodegradation under these conditions.

Photoluminescence quantum yields were determined relative to $[Ru(bpy)_3](PF_6)_2$ (ϕ = 0.095),[31, 32] using the equation:

$$\phi_{sample} = \phi_{ref} \frac{I_{sample}}{I_{ref}} \frac{OD_{ref}}{OD_{sample}} \frac{{n_{sample}}^2}{{n_{ref}}^2} \qquad Eq.S5$$

Where I is the integrated emission intensity, OD the optical density at the excitation wavelength, and *n* the solvent refractive index. In this work, the same solvent was used for both sample and reference samples, obviating the final term of the equation.

For both the Chuagev-type dicarbene complexes and $[Ru(bpy)_3](PF_6)_2$, the fluorimeter detection limit (850 nm) prevented complete integration of the photoluminescence signal. The following approach was therefore employed to improve the reliability of the determined quantum yields:

Photoluminescence spectra were recorded (using the above parameters) at six different concentrations, with optical densities ranging from 0 – 0.1 at the excitation wavelength (450 nm). SVD was used to decompose the concentration-dependent signal into left and right singular vectors containing spectral and concentration-dependent information, respectively (Figure S13-Figure S15). It is evident from the singular values, that for every complex investigated, the data is best described by a single component that exhibits a linear dependence on the optical density at the excitation wavelength, and with the spectral component matching the photoluminescence profile.

The spectral component was then fitted to a multi-mode Franck-Condon vibration progression:[33, 34]

$$I(\tilde{\nu}) = \sum_i \left(\sum_{\nu_i=0}^{\nu_i=m} \left(\left(\frac{\tilde{\nu}_0 - \sum_i \nu_i \hbar\omega_i}{\tilde{\nu}_0} \right)^3 \cdot \prod_i \left(\frac{{S_i}^{n_i}}{\nu_i!} \right) \cdot e^{\left(-4\ln(2) \cdot \left(\frac{\tilde{\nu} - \tilde{\nu}_0 + \nu_i \hbar\omega_i}{\Gamma} \right)^2 \right)} \right) \right) \qquad Eq.S6$$

Where $\tilde{\nu}$ is the measured frequency, $\tilde{\nu}_0$ is the electronic origin, $\hbar\omega_i$, S_i and ν_i the frequency, Huang-Rhys factor and vibrational quantum number of the ith vibrational mode, respectively, and Γ the Gaussian broadening (full-width half-maximum).

Before the spectral component obtained from SVD can be fitted using Eq. S6, it needs to be converted from wavelength to wavenumber scale. The emission intensity was therefore corrected using:

$$I(\tilde{\nu}) = \lambda^2 \cdot I(\lambda) \qquad Eq. S7$$

It is important to note that the purpose of this fit is not to yield physically meaningful parameters, but to model the experimental data with high fidelity. We found that two vibrational modes with twenty vibrational quantum numbers each (m=20) were sufficient to accurately model the spectral component obtained from SVD for all investigated complexes. The fit was then extrapolated beyond the fluorimeter detection limit, and converted back into wavelength scale (Figure S13a-Figure S15a).

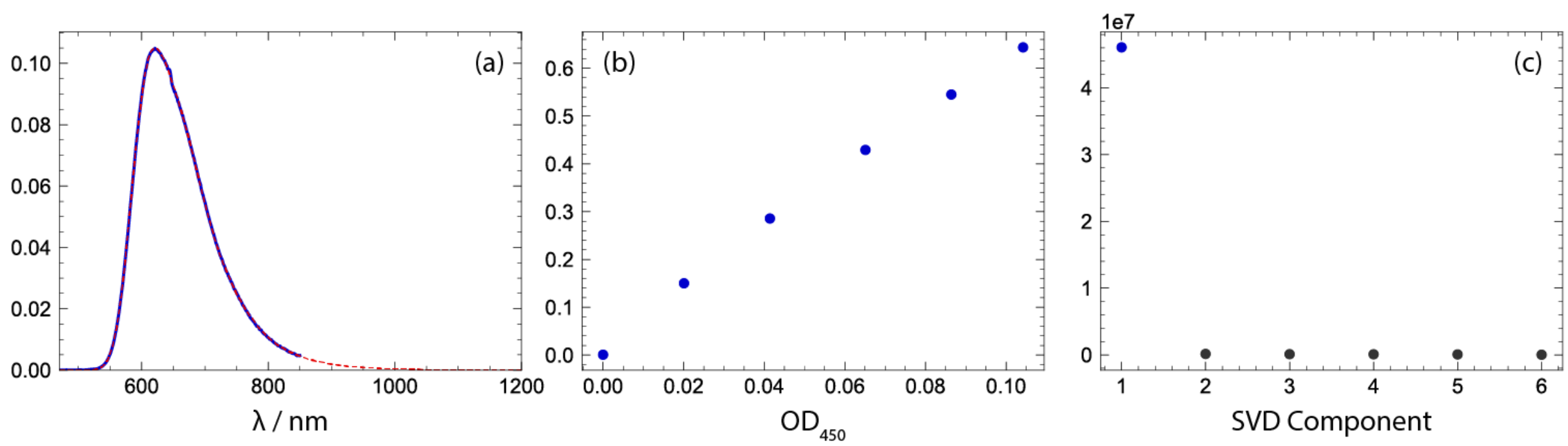

Figure S13. SVD and global fitting of the concentration-dependent room temperature photoluminescence spectra of $[Ru(bpy)_3](PF_6)_2$ in de-aerated acetonitrile (λ_{ex} = 450 nm). (a) First left singular vector (solid blue trace, basis spectrum) and fit to Eq. S6 (red dashed trace); (b) First right singular vector; (c) singular values. The blue singular value in panel (c) corresponds to the blue left and right singular vectors in panels (a) and (b).

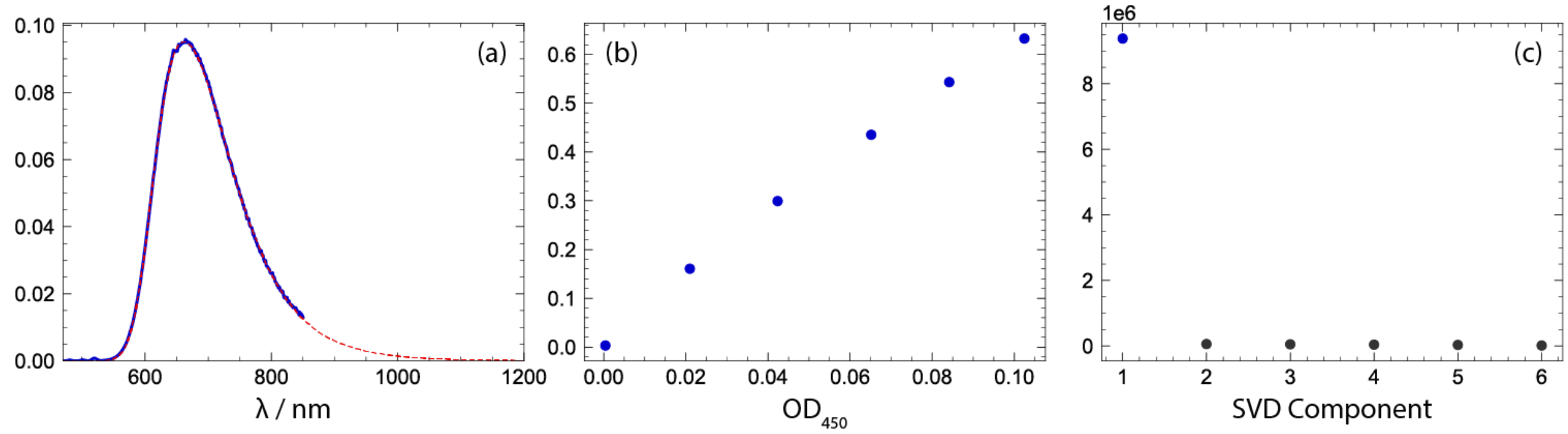

Figure S14. SVD and global fitting of the concentration-dependent room temperature photoluminescence spectra of [Ru(bpy)$_2$($C_4H_{10}N_4$)](PF_6)$_2$ in de-aerated acetonitrile (λ_{ex} = 450 nm). (a) First left singular vector (solid blue trace, basis spectrum) and fit to Eq. S6 (red dashed trace); (b) First right singular vector; (c) singular values. The blue singular value in panel (c) corresponds to the blue left and right singular vectors in panels (a) and (b).

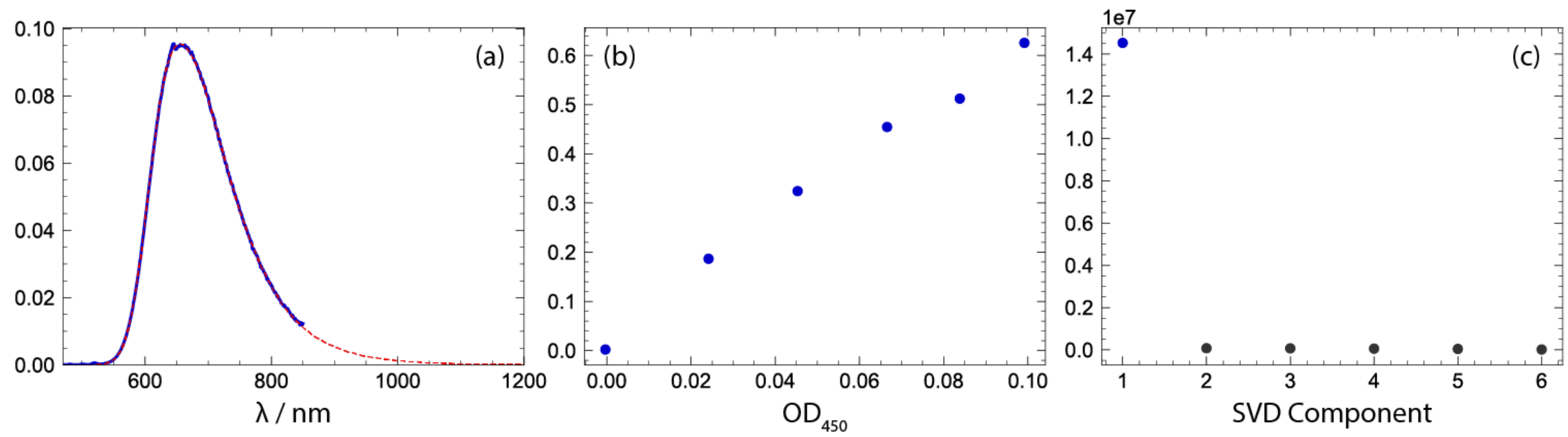

Figure S15. SVD and global fitting of the concentration-dependent room temperature photoluminescence spectra of [Ru(^{t}Bu$_2$bpy)$_2$($C_4H_{10}N_4$)](PF_6)$_2$ in de-aerated acetonitrile (λ_{ex} = 450 nm). (a) First left singular vector (solid blue trace, basis spectrum) and fit to Eq. S6 (red dashed trace); (b) First right singular vector; (c) singular values. The blue singular value in panel (c) corresponds to the blue left and right singular vectors in panels (a) and (b).

The concentration-dependent spectra were then rebuilt from the extrapolated fits using:

$$I(\lambda, c) = I(\lambda)_{fit} \cdot S \cdot V_c^{\,T} \qquad Eq. S8$$

Where S is the first singular value (Figure S13c-Figure S15c), and V_c the first right singular vectors (Figure S13b-Figure S15b). It can be observed in Figure S16a,b-Figure S17a,b, that this global fitting procedure yields excellent fits to the experimental data.

The integrated intensity of the extrapolated fits was then plotted against the optical density at the excitation wavelength, yielding linear plots (Figure S16c-Figure S17c). As the sample and reference complexes were both measured in acetonitrile, Eq. S5 can be rearranged to:

$$\phi_{sample} = \phi_{ref} \frac{I_{sample}/OD_{sample}}{I_{ref}/OD_{ref}} \qquad Eq. S9$$

The slopes obtained by linear regression of the integrated intensity for the sample and reference against optical density at the excitation wavelength yield the numerator and denominator, respectively, of Eq. S9. Determined quantum yields are presented in Table 3 in the main paper.

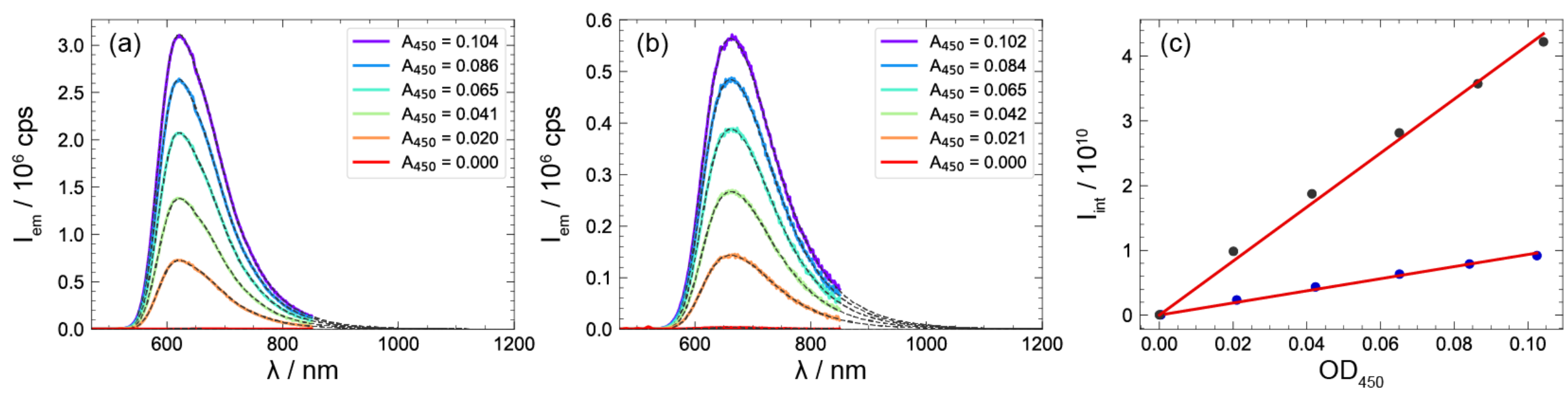

Figure S16. Concentration-dependent photoluminescence spectra of (a) $[Ru(bpy)_3](PF_6)_2$ and (b) $[Ru(bpy)_2(C_4H_{10}N_4)](PF_6)_2$ in de-aerated acetonitrile (colored traces), with extrapolated fits to Eq. S6. (c) Plot of integrated photoluminescence intensity from extrapolated fits to Eq. S6 against the optical density at the excitation wavelength (OD_{450}). Black points correspond to $[Ru(bpy)_3](PF_6)_2$ and blue points to $[Ru(bpy)_2(C_4H_{10}N_4)](PF_6)_2$. Red lines are linear regression fits to the data sets.

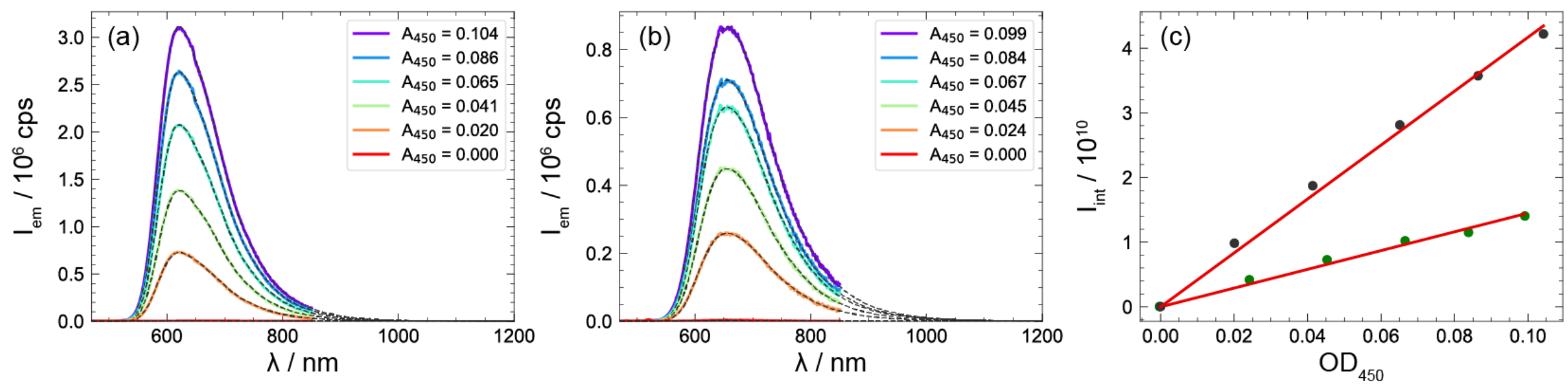

Figure S17. Concentration-dependent photoluminescence spectra of (a) $[Ru(bpy)_3](PF_6)_2$ and (b) $[Ru(dtbbpy)_2(C_4H_{10}N_4)](PF_6)_2$ in de-aerated acetonitrile (colored traces), with extrapolated fits to Eq. S6. (c) Plot of integrated photoluminescence intensity from extrapolated fits to Eq. S6 against the optical density at the excitation wavelength (OD_{450}). Black points correspond to $[Ru(bpy)_3](PF_6)_2$ and green points to $[Ru(dtbbpy)_2(C_4H_{10}N_4)](PF_6)_2$. Red lines are linear regression fits to the data sets.

Spectral Bandshape and Nonradiative Decay Theory Analysis

Both electronic absorption and emission spectral lineshapes can be described by single-mode Franck-Condon vibrational progressions:[33, 35-37]

$$A(\tilde{\nu}) = \sum_{\nu}^{\infty} \frac{\tilde{\nu}_0 - \nu \cdot \hbar\omega}{\tilde{\nu}_0} \cdot \frac{S^\nu}{\nu!} \cdot e^{\left(-4\ln(2)\cdot\left(\frac{\tilde{\nu}-\tilde{\nu}_0-\nu\cdot\hbar\omega}{\Gamma}\right)^2\right)} \qquad Eq.S10a$$

$$I(\tilde{\nu}) = \sum_{\nu}^{\infty} \left(\frac{\tilde{\nu}_0 - \nu \cdot \hbar\omega}{\tilde{\nu}_0}\right)^3 \cdot \frac{S^\nu}{\nu!} \cdot e^{\left(-4\ln(2)\cdot\left(\frac{\tilde{\nu}-\tilde{\nu}_0+\nu\cdot\hbar\omega}{\Gamma}\right)^2\right)} \qquad Eq.S10b$$

Where $\tilde{\nu}$ is the measured frequency, $\tilde{\nu}_0$ is the electronic origin, $\hbar\omega_i$, S_i and ν_i the frequency, Huang-Rhys factor and vibrational quantum number of the ith vibrational mode, respectively, and Γ the Gaussian broadening (full-width half-maximum), which is related to the classical reorganization energy (λ_{cl}) through:

$$\lambda_{cl} = \frac{\Gamma^2}{16\ln(2)\,k_B T} \qquad Eq.S11$$

and includes classically treated low frequency coupled modes.

Although electronic transitions are generally coupled to multiple vibrational modes, the single mode description is generally adequate, instead describing a weighted average of coupled modes:[33]

$$S = \sum_i S_i \qquad Eq.S12a$$

$$\hbar\omega = \frac{\sum_i S_i \hbar\omega_i}{\sum_i S_i} \qquad Eq.S12b$$

Where $\hbar\omega_i$ and S_i are the frequency and Huang-Rhys factor for individual coupled modes, respectively. This averaging approach is valid if the range of contributing mode frequencies is small relative to the average. The description for multiple coupled modes is described by Eq. S6.

Electronic absorption spectra of the investigated complexes in acetonitrile are in Figure 2 of the main paper. Magnifications of the MLCT absorption manifold are presented in Figure S18.

The MLCT absorption features of the homoleptic and Chugaev-type dicarbene complexes in acetonitrile were fitted to a single (averaged) mode Franck-Condon vibrational progression (Eq. S10a). An additional Gaussian band was also required to adequately model the low energy tail of the MLCT absorption features, which are assigned as ^{3}MLCT in character (Figure S18). It should be noted that a Franck-Condon vibrational progression was unable to fit the MLCT absorption features of the *cis*-di(methylisocyanide) complexes, which occur at significantly higher energy. These were instead modelled using two Gaussian peaks. The fit parameters for the ^{1}MLCT absorptions of homoleptic and Chugaev-type dicarbene complexes are reported in Table S5.

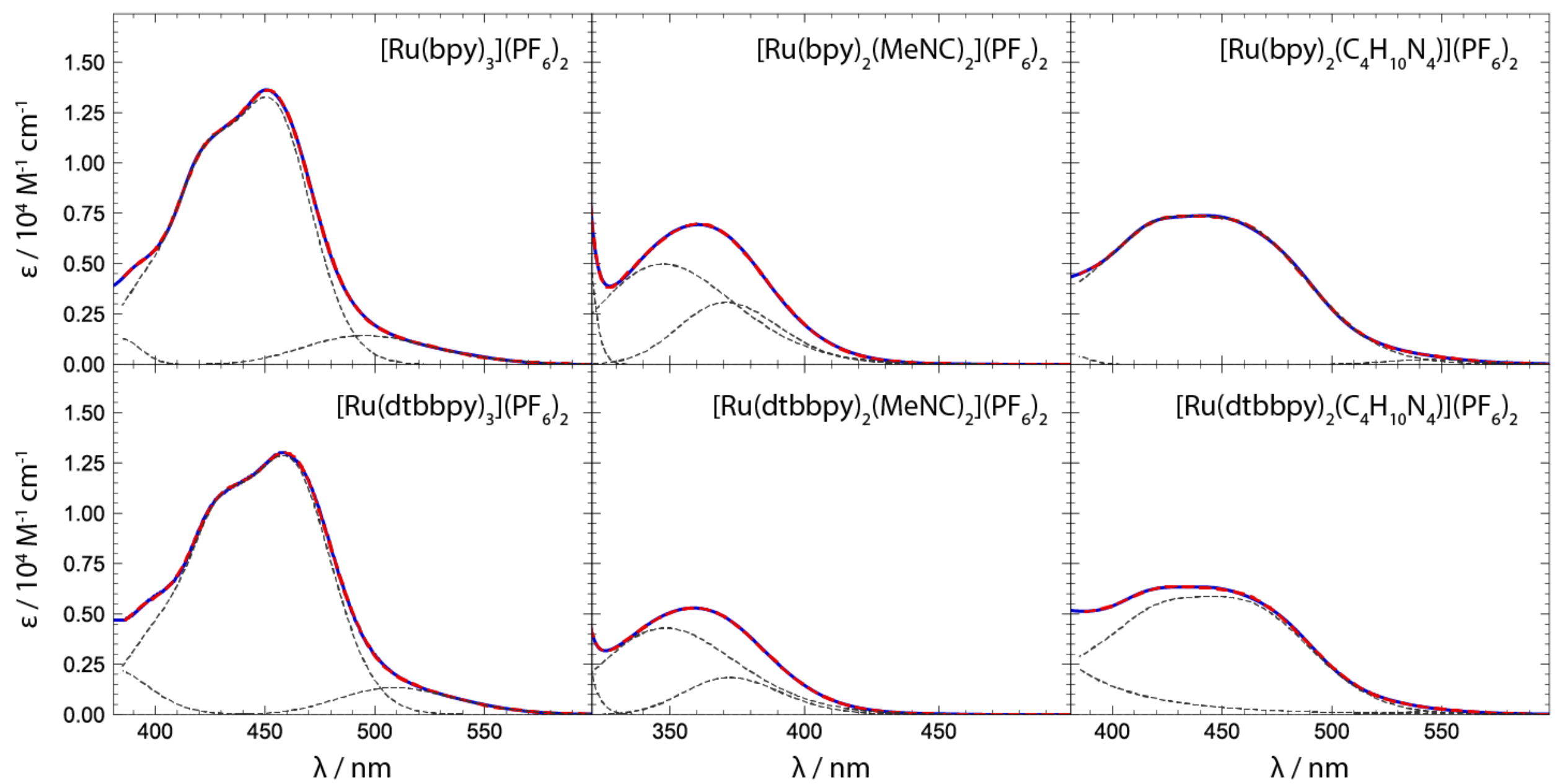

Figure S18. UV-vis electronic absorption spectra (solid blue traces) of the MLCT absorptions of the investigated complexes in acetonitrile, and corresponding fits as described above (red dashed traces) and individual fit contributions (black dashed traces).

Table S5. Parameters derived from least squares fitting of the ^{1}MLCT absorption feature of investigated complexes in acetonitrile to a single (averaged) mode Franck-Condon vibrational progression (Eq. S10a). Uncertainties are reported to the 95% confidence interval.

Complex	$\tilde{\nu}_0$ / cm^{-1}	hω / cm^{-1}	S	λ / cm^{-1}	Γ / cm^{-1}	λ_{cl} / cm^{-1}
$[Ru(bpy)_3](PF_6)_2$	21,977(3)	1,649(4)	0.837(4)	1,380(7)	1,855(6)	1,498(5)
$[Ru(bpy)_2(C_4H_{10}N_4)](PF_6)_2$	21,480(6)	2,306(7)	1.124(5)	2,590(20)	2,850(20)	3,540(30)
$[Ru(dtbbpy)_3](PF_6)_2$	21,584(5)	1,700(7)	0.862(4)	1,465(9)	1,901(8)	1,573(7)
$[Ru(dtbbpy)_2(C_4H_{10}N_4)](PF_6)_2$	21,438(6)	2,240(10)	1.060(7)	2,380(20)	2,780(20)	3,360(30)

Consistent with previous studies, we find that $[Ru(bpy)_3](PF_6)_2$ has its ^{1}MLCT absorption coupled to high frequency modes with a weighted average frequency of 1,650 cm^{-1}.[36, 38] Unsurprisingly, the fit results for $[Ru(dtbbpy)_3](PF_6)_2$ are very similar to those of $[Ru(bpy)_3](PF_6)_2$.

Interestingly, the fit results for the Chugaev-type dicarbene complexes deviate substantially from those of their corresponding homoleptic analogues. The coupled high frequency vibrational modes have a weighted average frequency of ~2,300 cm^{-1}, indicating contribution from the non-chromophoric Chugaev-type

dicarbene ligand. The Huang-Rhys factors also increase substantially, indicating greater structural distortion in the ^{1}MLCT excited state, as the Huang-Rhys factor for an individual vibrational mode is related to its reorganization energy (λ_i):

$$S_i = \frac{\lambda_i}{\hbar\omega_i} \qquad Eq. S13$$

Reorganization energies obtained through application of Eq. S13 highlight that the combination of a larger Huang-Rhys factor and higher frequency coupled modes results in a substantially increased excited state distortion. This also manifests in a larger solvent reorganization energy, consistent with greater directionality of the ^{1}MLCT excited state due to the descent in symmetry.

Steady-state emission spectra (λ_{ex} = 450 nm) of the investigated complexes in acetonitrile at room temperature are presented in Figure 2 of the main paper, and Figure S19 with corresponding fits to a single (averaged) mode Franck-Condon vibrational progression (Eq. S10b). The fit parameters are reported in Table S6.

There are some important differences in the Franck-Condon parameters determined from spectral lineshape fitting of the steady-state emission spectra versus electronic absorbance spectra, arising from the different states being probed (^{1}MLCT for absorption, ^{3}MLCT for emission). It has been previously reported for $[Ru(bpy)_3]^{2+}$ that the vibrational modes coupled with the ^{3}MLCT emission have a lower weighted average frequency and larger Huang-Rhys factor than those coupled with the ^{1}MLCT absorption. Our fit results are consistent with previous reports. Application of Eq. S13, however, yields reorganization energies of similar magnitude to those obtained from Franck-Condon lineshape analysis of the ^{1}MLCT absorption, and the classical reorganization energies are likewise similar, indicating similar extents of structural distortion between the $^{1/3}$MLCT excited states.

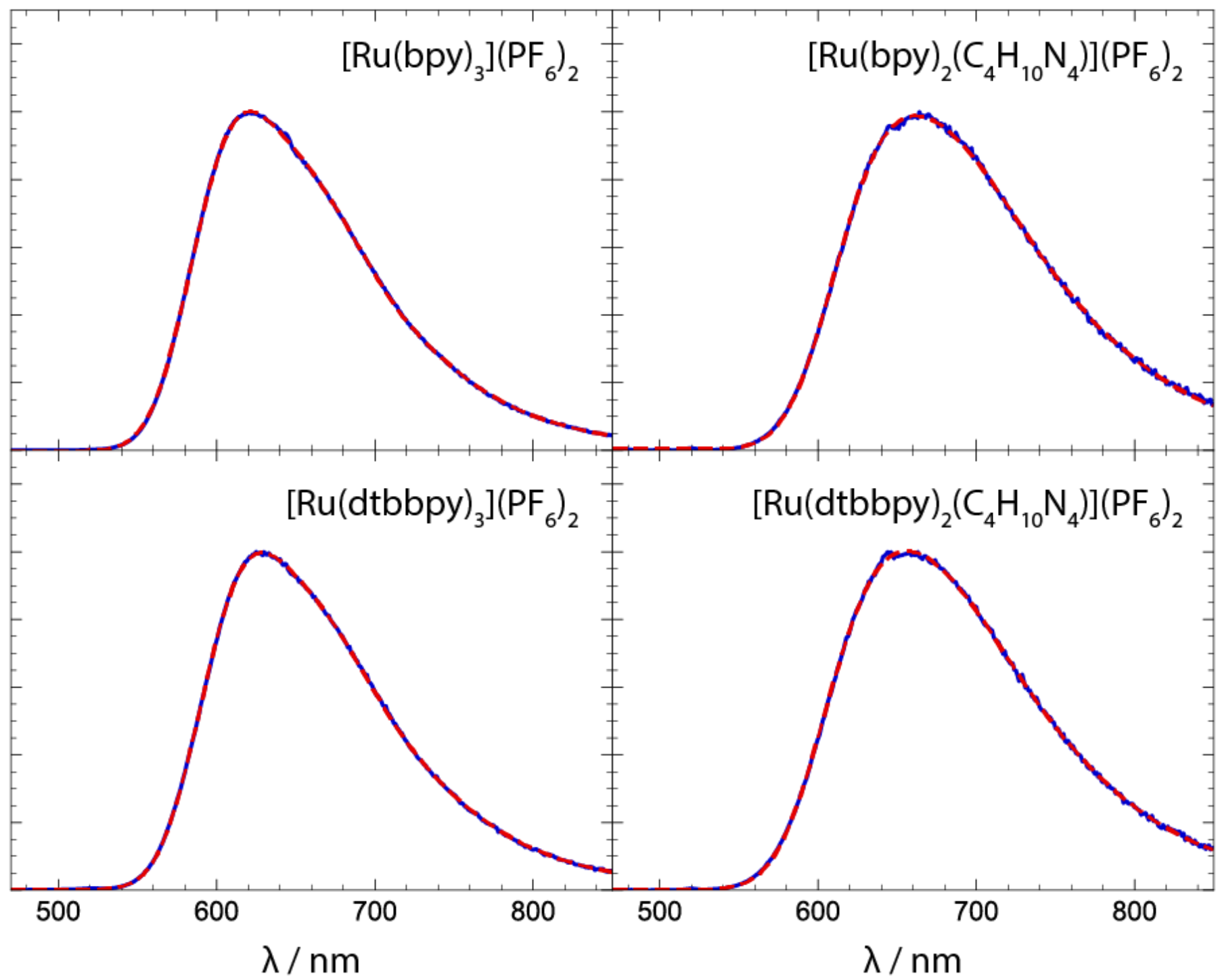

Figure S19. Normalized steady-state emission spectra (λ_{ex} = 450 nm) of the investigated complexes recorded at room temperature in acetonitrile (solid blue traces), and corresponding fits to a single (averaged) mode Franck-Condon vibrational progression (red dashed traces, Eq. S10b). Note, $[Ru(bpy)_2(MeNC)_2](PF_6)_2$ and $[Ru(dtbbpy)_2(MeNC)_2](PF_6)_2$ are non-emissive.

Table S6. Parameters derived from least squares fitting of the ^{3}MLCT emission of investigated complexes at room temperature in acetonitrile to a single (averaged) mode Franck-Condon vibrational progression (Eq. S10b). Uncertainties are reported to the 95% confidence interval.

Complex	$\tilde{\nu}_0$ / cm^{-1}	hω / cm^{-1}	S	λ / cm^{-1}	Γ / cm^{-1}	λ_{cl} / cm^{-1}	$E_{0,0}$ / cm^{-1} [a]
$[Ru(bpy)_3](PF_6)_2$	16,326(6)	1,376(6)	1.072(9)	1,480(20)	1,792(9)	1,398(7)	17,724(9)
$[Ru(bpy)_2(C_4H_{10}N_4)](PF_6)_2$	15,590(40)	1,280(30)	1.30(5)	1,670(80)	1,900(40)	1,570(40)	17,160(60)
$[Ru(dtbbpy)_3](PF_6)_2$	16,156(7)	1,354(7)	1.09(1)	1,480(20)	1,800(10)	1,411(8)	17,570(20)
$[Ru(dtbbpy)_2(C_4H_{10}N_4)](PF_6)_2$	15,720(40)	1,300(20)	1.28(5)	1,660(70)	1,970(40)	1,690(40)	17,410(60)

[a] Obtained from $\tilde{\nu}_0 + \lambda_{cl}$

More interesting are the large discrepancies between the $^{1/3}$MLCT Franck-Condon parameters obtained for the Chugaev-type dicarbene complexes. In stark contrast to the absorption parameters, those obtained from fitting the ^{3}MLCT emission are much more similar to those of the homoleptic complexes, indicating that although there are significant differences in the initially populated Franck-Condon excited state, the excited

state relaxes into a structurally similar lowest ^{3}MLCT state. Although the *cis*-di(methylisocyanide) complexes could not be studied at room temperature, 77 K measurements (see below) suggest that this is also true for those complexes.

Steady-state emission spectra (λ_{ex} = 450 nm) of the investigated complexes in frozen butyronitrile glass at 77 K are presented in Figure 2 of the main paper, and Figure S20 with corresponding fits to a two-mode Franck-Condon vibrational progression (Eq. S6). The fit parameters are reported in Table S7.

It has been previously demonstrated that room temperature photoluminescence spectra can be satisfactorily modelled using a single high-frequency vibrational mode, which is actually a weighted average of several high-frequency modes, whereas a second low-frequency mode is required to model 77 K emission spectra.[39] This arises because low frequency modes can be treated classically when the thermal energy $k_BT \approx \lambda$ (k_BT = 207 cm^{-1} at 298 K), appearing as additional broadening, but must be explicitly accounted for when $k_BT << \lambda$ (k_BT = 54 cm^{-1} at 77 K). Consistent with prior reports, inclusion of a second mode was required to model the 77 K emission spectra in this work.

Comparison of room temperature to 77 K spectra shows good general agreement. Differences in frequencies and Huang-Rhys factors / reorganization energies arise from the differential treatment of low-to-medium frequency modes in the single- versus two-mode treatment.

In frozen glass at 77 K, librational motion is severely restricted such that solvent cannot reorient.[40] Therefore, in 77 K measurements, the electronic origin is approximately equal to $E_{0,0}$, the difference in energy between the ground and excited state. Indeed, there is generally good agreement between the $E_{0,0}$ values determined at room temperature and at 77 K, with the latter consistently slightly smaller. This is likely due to limited solvent motion still being possible even in frozen glass.

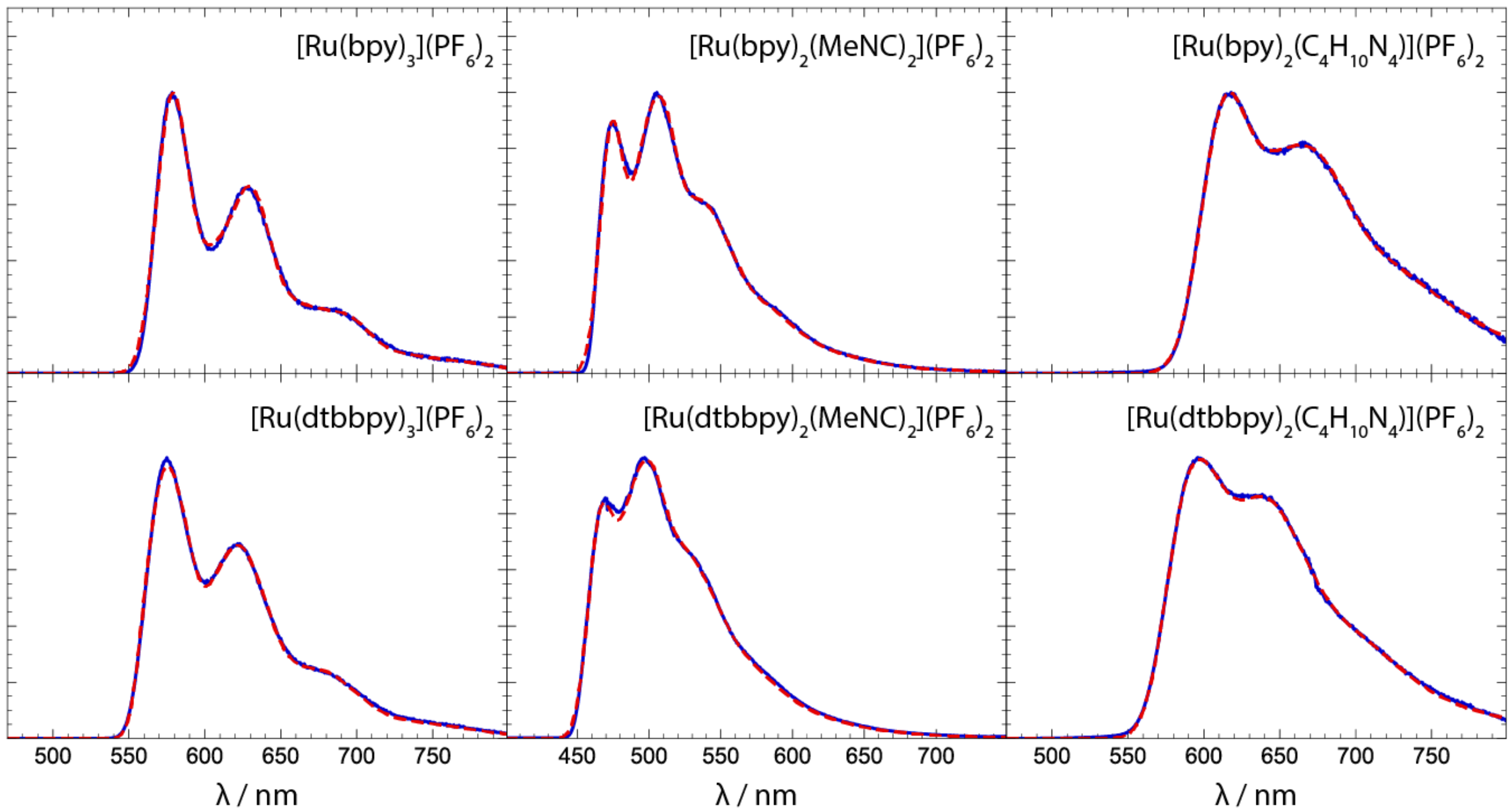

Figure S20. Normalized steady-state emission spectra of the investigated complexes in frozen butyronitrile glass at 77 K (solid blue traces), and corresponding fits to a two-mode Franck-Condon vibrational progression (Eq. S6, red dashed traces).

Table S7. Parameters derived from least squares fitting of the ^{3}MLCT emission of investigated complexes at 77 K in butyronitrile glasses to a two- mode Franck-Condon vibrational progression (Eq. S6). Uncertainties are reported to the 95% confidence interval.

Complex	$E_{0,0}$ / cm^{-1}	$h\omega_1$ / cm^{-1}	S_1	λ_1 / cm^{-1}	$h\omega_2$ / cm^{-1}	S_2	λ_2 / cm^{-1}	Γ / cm^{-1}
$[Ru(bpy)_3](PF_6)_2$	17,269(3)	1,432(5)	0.87(1)	1,250(20)	800(20)	0.430(8)	340(20)	765(6)
$[Ru(bpy)_2(MeNC)_2](PF_6)_2$	21,078(5)	1,501(9)	1.05(3)	1,580(50)	930(20)	0.85(2)	790(30)	890(10)
$[Ru(bpy)_2(C_4H_{10}N_4)](PF_6)_2$	16,267(6)	1,547(9)	0.79(2)	1,220(40)	890(20)	0.76(2)	680(30)	1,014(9)
$[Ru(^tBu_2bpy)_3](PF_6)_2$	17,750(20)	1,381(3)	0.965(4)	1,333(7)	353(9)	1.7(1)	600(40)	530(20)
$[Ru(^tBu_2bpy)_2(MeNC)_2](PF_6)_2$	21,423(8)	1,510(10)	1.06(3)	1,600(50)	890(20)	0.94(2)	840(30)	980(20)
$[Ru(^tBu_2bpy)_2(C_4H_{10}N_4)](PF_6)_2$	17,020(50)	1,424(8)	1.00(2)	1,420(30)	570(50)	1.0(2)	600(200)	970(40)

Assuming negligible contributions from indirect (thermally activated) nonradiative decay, the Franck-Condon fit parameters for the ^{3}MLCT emission at room temperature can be applied to understand direct (vibrationally coupled) nonradiative decay from the same state through application of the "Energy Gap Law".[33, 39-43]

The main prediction of the energy gap law is that the rate of nonradiative decay is proportional to the negative exponential of the emission energy:

$$k_{nr} \propto e^{-E_0} \qquad Eq.S14$$

To initially assess the reliability of an Energy Gap Law analysis, ln(k_{nr}) was plotted against E_0 (Figure S21). The apparent linear relationship is suggestive that the direct (vibrationally coupled) mechanism is the major contributor to the rate of nonradiative decay.

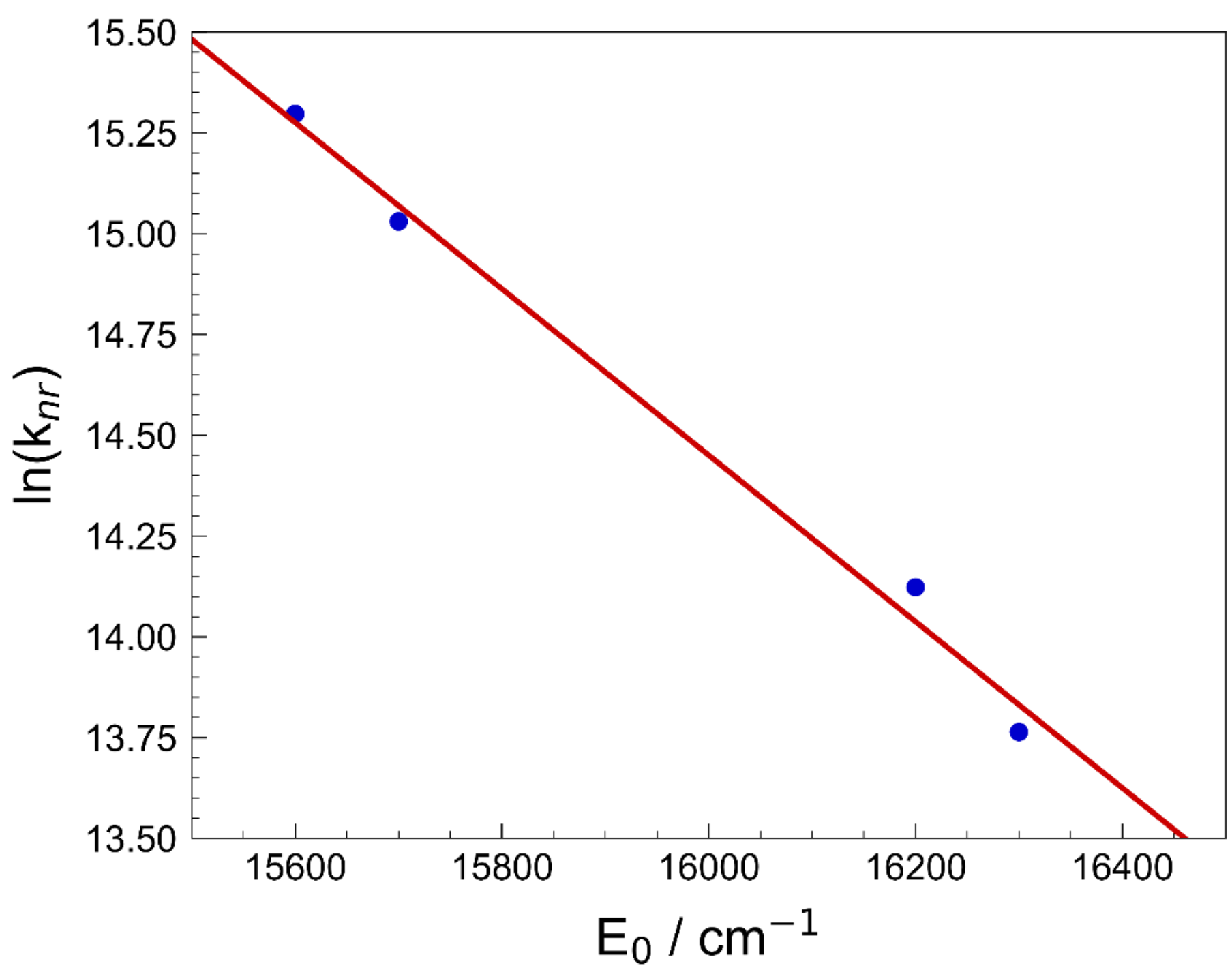

Figure S21. "Energy Gap Law" plot of ln(k_{nr}) *vs* emission energy (blue dots) and linear regression fit (solid red line).

A more thorough description of the Energy-Gap Law is:

$$\ln(k_{nr}) = \ln(\beta_0) + \ln(FC_{calc}) \qquad Eq.S15a$$

$$\beta_0 = \frac{V_k^2\sqrt{2\pi}}{\hbar \cdot (1{,}000\ cm^{-1})} \qquad Eq.S15b$$

$$\ln(FC_{calc}) = -\frac{\ln(\hbar\omega \cdot \tilde{\nu}_0)}{2 \cdot (1{,}000\ cm^{-1})^2} - S - \frac{\gamma E_0}{\hbar\omega} + \frac{(\gamma+1)^2}{16\ln(2)}\left(\frac{\Gamma}{\hbar\omega}\right)^2 \qquad Eq.S15c$$

$$\gamma = ln\left(\frac{\tilde{\nu}_0}{S \cdot \hbar\omega}\right) - 1 \qquad Eq.S15d$$

Where the Franck-Condon parameters are the same as for Eq. S10b, and V_k is the vibrationally-induced electronic coupling matrix element between the ground and ^{3}MLCT excited states. V_k is not derived from fitting the emission profile to a Franck-Condon vibrational progression, and is instead derived from the experimental nonradiative rate constants and the Franck-Condon parameters. Specifically, a plot of $ln(k_{nr})$ vs $ln(FC_{calc})$ (Figure S22) has a unity slope, with the y-intercept equal to $-ln(\beta_0)$. From Eq. S15b, V_k was determined as 215 cm^{-1}. This value is surprisingly different from the V_k previously determined for a series of Ru(II) complexes of ~1,300 cm^{-1} at 157 K.[41] However, comparison of the two datasets (Figure S22), particularly the $[Ru(bpy)_3](PF_6)_2$ values, highlights a potential temperature-dependence of electronic coupling.

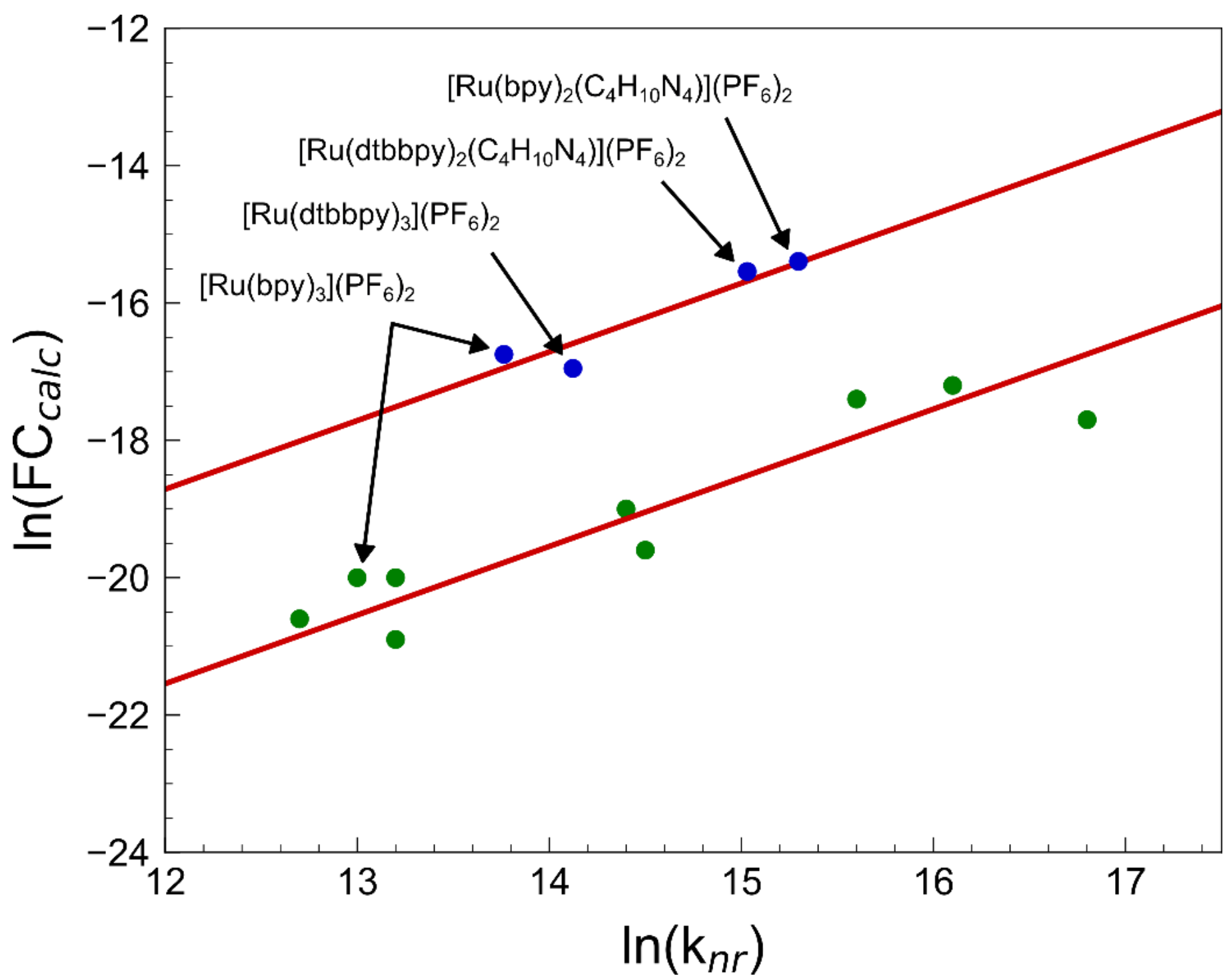

Figure S22. Plot of $ln(FC_{calc})$ vs $ln(k_{nr})$ for the investigated complexes (blue dots) and for a series of Ru(II) complexes at 157 K from reference [41] (green dots), and linear regression fits (solid red lines), with slopes fixed at 1. For the investigated complexes, $ln(\beta_0)$ = 30.7 ± 0.4.

Using the determined V_k of 215 cm^{-1}, the calculated nonradiative rates are reported in Table 3 of the main paper, as well as Table S8. There is good correlation between all experimental and calculated nonradiative decay constants, with the greatest variance for $[Ru(dtbbpy)_3](PF_6)_2$. We believe this variance may arise from experimental uncertainty in the reported emission quantum yield and/or lifetime for this complex.

These results demonstrate that the 3-4 fold enhancement of the nonradiative rate constant in the Chugaev-type dicarbene complexes relative to their corresponding homoleptic analogues can be explained without invoking thermal population of higher energy LF states, but rather appear to arise primarily from the stabilization of the ^{3}MLCT excited state as described by the main prediction of the Energy-Gap Law.

Table S8. Energy-Gap Law parameters.

Complex	k_{nr} (exp) / 10^6 s^{-1}	$ln(k_{nr})$	$ln(FC_{calc})$	k_{nr} (calc) / 10^6 s^{-1}
$[Ru(bpy)_3](PF_6)_2$	1.16	13.8	-16.8	1.16
$[Ru(bpy)_2(C_4H_{10}N_4)](PF_6)_2$	4.40	15.3	-15.4	4.50
$[Ru(dtbbpy)_3](PF_6)_2$	1.36	14.1	-17.0	0.95
$[Ru(dtbbpy)_2(C_4H_{10}N_4)](PF_6)_2$	3.37	15.0	-15.5	3.89

Time Correlated Single Photon Counting

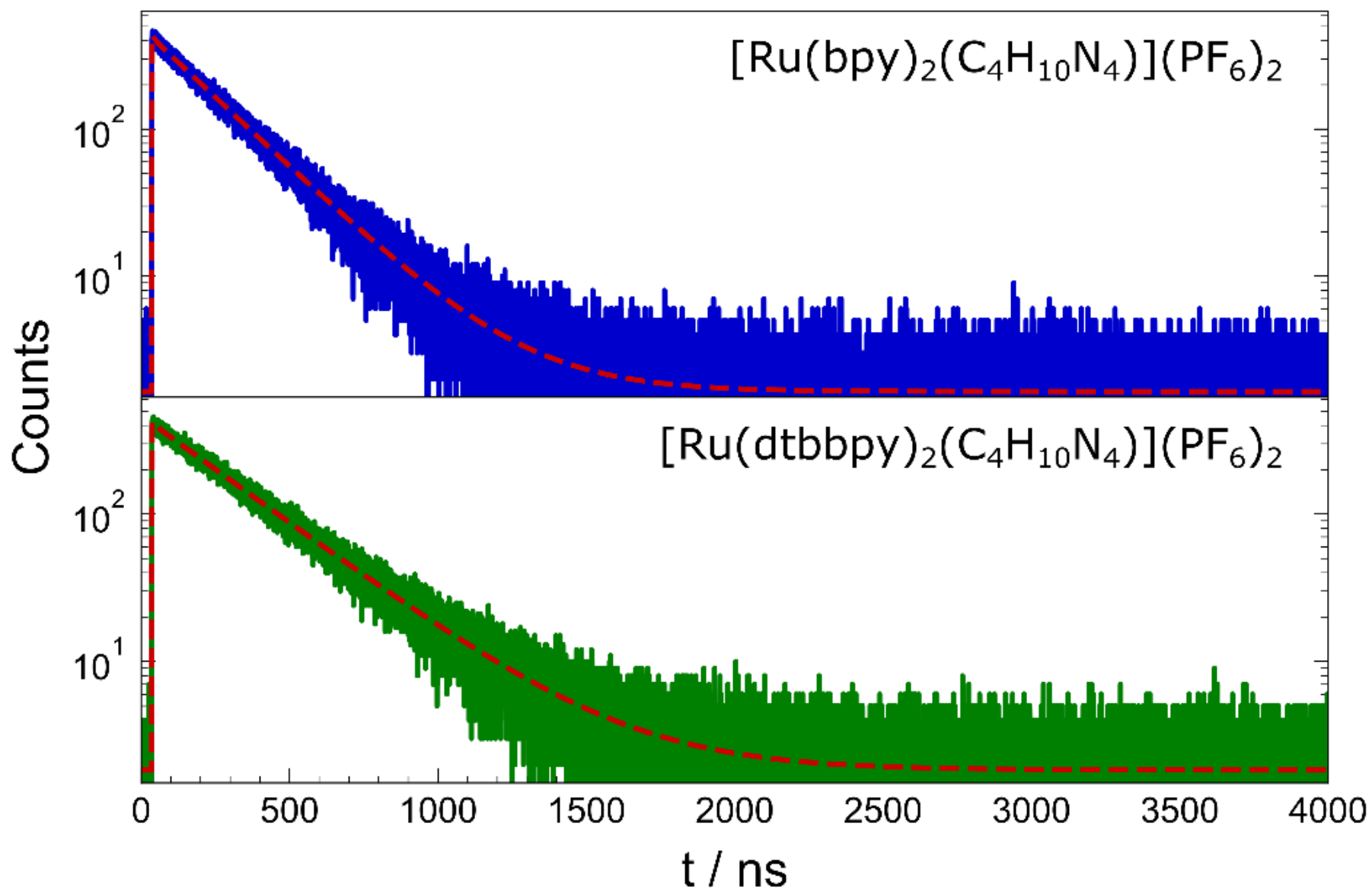

Figure S23. Decay of the emission signal of the Chugaev-type dicarbene complexes obtained from time-correlated single photon counting in de-aerated acetonitrile at room temperature (λ_{ex} = 440 nm) and corresponding monoexponential fits.

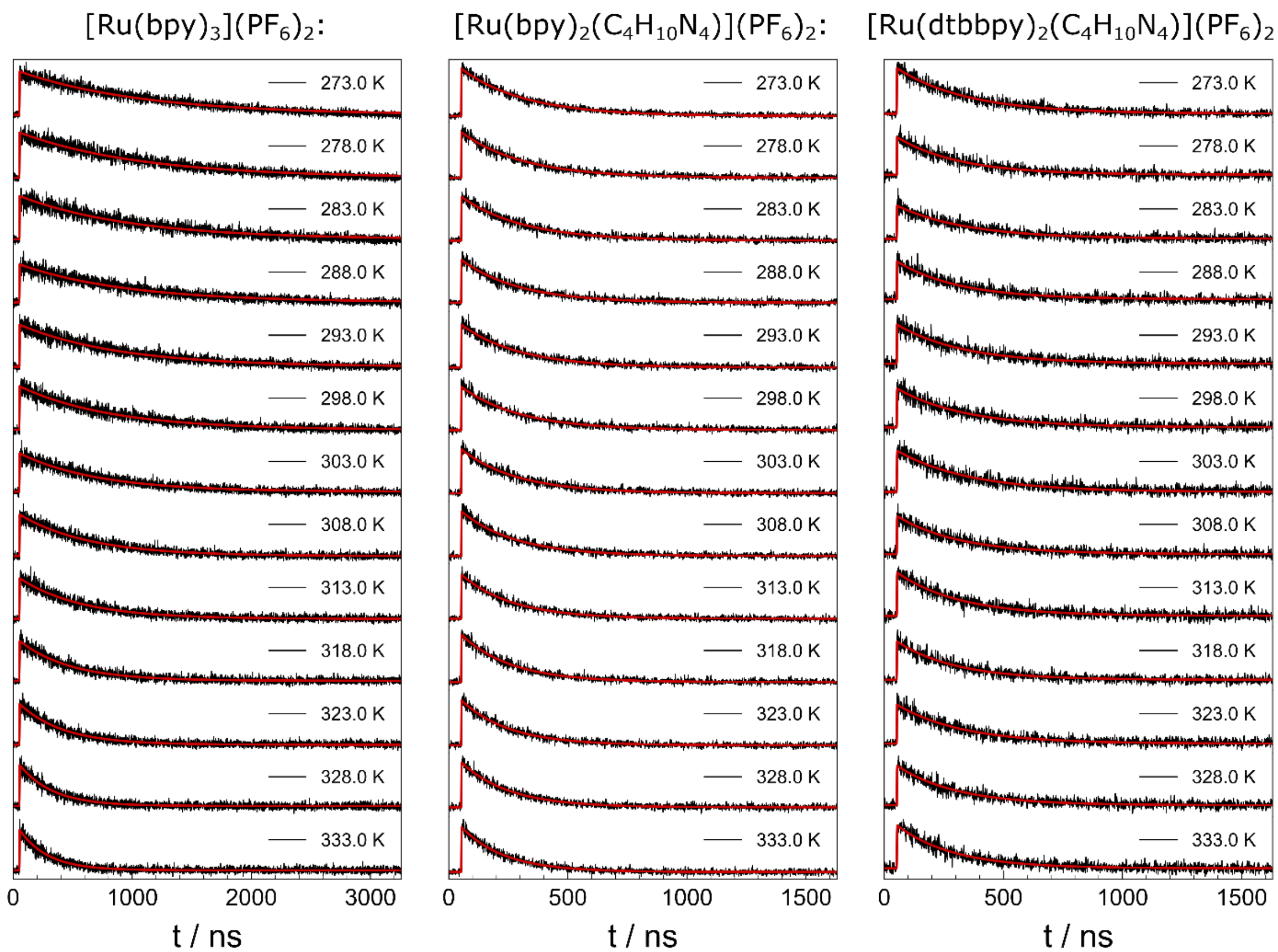

Figure S24. Decay of the emission signal of $[Ru(bpy)_3](PF_6)_2$ and the Chugaev-type dicarbene complexes obtained from time-correlated single photon counting in de-aerated acetonitrile at specified temperatures (λ_{ex} = 450 nm) and corresponding monoexponential fits.

Table S9. Lifetimes obtained from monoexponential fitting of variable temperature TCSPC decays. Uncertainties are reported to the 95% confidence interval.

Temperature / K	$[Ru(bpy)_3](PF_6)_2$	$[Ru(bpy)_2(C_4H_{10}N_4)](PF_6)_2$	$[Ru(dtbbpy)_2(C_4H_{10}N_4)](PF_6)_2$
273	1040(20)	267(4)	295(7)
278	1020(20)	261(5)	261(8)
283	950(20)	258(5)	295(9)
288	900(20)	242(5)	260(9)
293	840(20)	245(5)	281(9)
298	770(20)	248(5)	287(9)
303	660(20)	234(5)	303(9)
308	584(9)	228(5)	265(8)
313	523(9)	214(5)	262(8)
318	425(8)	209(4)	247(8)
323	367(7)	200(4)	275(8)
328	278(6)	200(4)	251(8)
333	239(6)	181(4)	237(7)

As discussed in the main paper, variable temperature emission lifetimes have previously been analyzed using both Arrhenius- (Eq. S16) and Boltzmann-like (Eq. S17) models:

$$\tau_{em} = \frac{1}{k_{MLCT} + Ae^{-\Delta E/k_B T}} \qquad Eq. S16$$

$$k_{obs} = \frac{1}{\tau_{em}} = \frac{k_{MLCT} + k_{LF}e^{-\Delta E/k_B T}}{1 + e^{-\Delta E/k_B T}} \qquad Eq. S17$$

In this work, we fitted the variable temperature emission lifetimes using both models (Figure S25, Table S10Table S11), which yield similar results.

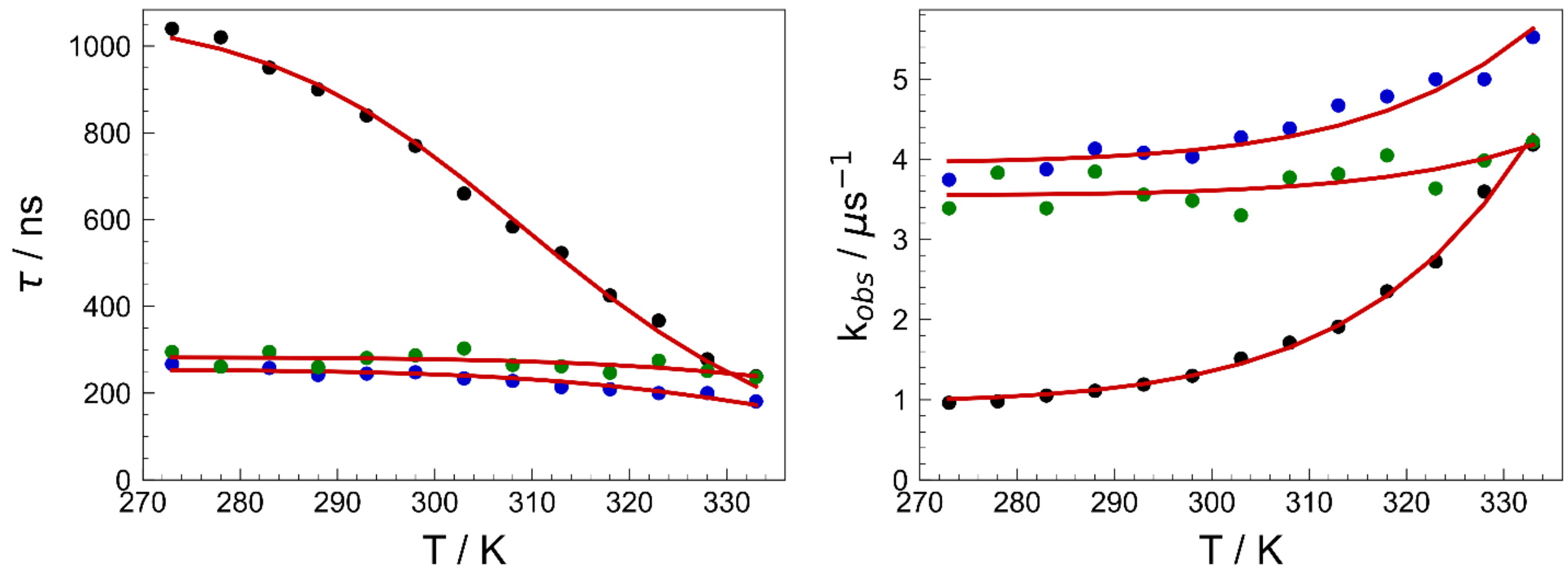

Figure S25. Emission lifetimes and rate constants (k_{obs} = 1/τ) of [Ru(bpy)$_3$](PF$_6$)$_2$ (black), [Ru(bpy)$_2$(C$_4$H$_{10}$N$_4$)](PF$_6$)$_2$ (blue) and Ru(dtbbpy)$_2$(C$_4$H$_{10}$N$_4$)](PF$_6$)$_2$ (green) obtained from variable temperature TCSPC (λ_{ex} = 450 nm), and corresponding fits (red traces) to Eq. S16 (left panel) and Eq. S17 (right panel).

Table S10. Parameters derived from least squares fitting of the variable temperature emission lifetimes to an Arrhenius-type model (Eq. S16). Uncertainties are reported to the 95% confidence interval.

Complex	k_{MLCT} / 10^6 s^{-1}	A / 10^{15} s^{-1}	ΔE / cm^{-1}
[Ru(bpy)$_3$](PF$_6$)$_2$	0.94(3)	1.97(1)	4,650(20)
[Ru(bpy)$_2$(C$_4$H$_{10}$N$_4$)](PF$_6$)$_2$	3.9(2)	3.16(1)	4,920(70)
[Ru(dtbbpy)$_2$(C$_4$H$_{10}$N$_4$)](PF$_6$)$_2$	3.5(2)	1.44(1)	5,000 (200)

Table S11. Parameters derived from least squares fitting of the variable temperature emission lifetimes to a Boltzmann-type model (Eq. S17). Uncertainties are reported to the 95% confidence interval.

Complex	k_{MLCT} / 10^6 s^{-1}	k_{LF} / 10^{15} s^{-1}	ΔE / cm^{-1}
[Ru(bpy)$_3$](PF$_6$)$_2$	0.96(5)	0.89(1)	4,490(20)
[Ru(bpy)$_2$(C$_4$H$_{10}$N$_4$)](PF$_6$)$_2$	4.0(2)	0.82(1)	4,630(50)
[Ru(dtbbpy)$_2$(C$_4$H$_{10}$N$_4$)](PF$_6$)$_2$	3.5(2)	1.02(1)	4,900(200)

Ru L_3-Edge X-Ray Spectroscopy

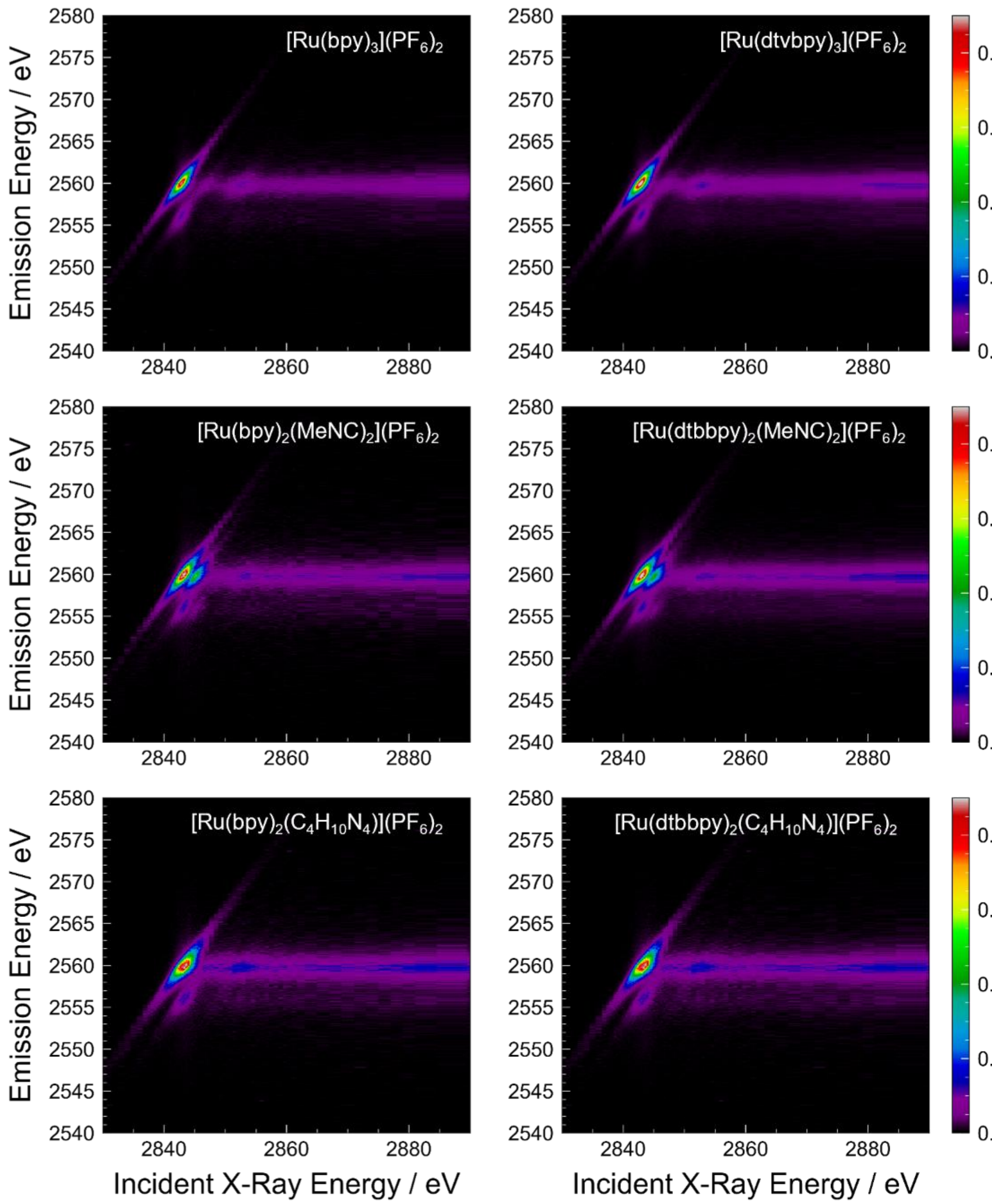

Figure S26. Normalized Ru 2p3d RIXS maps for investigated complexes.

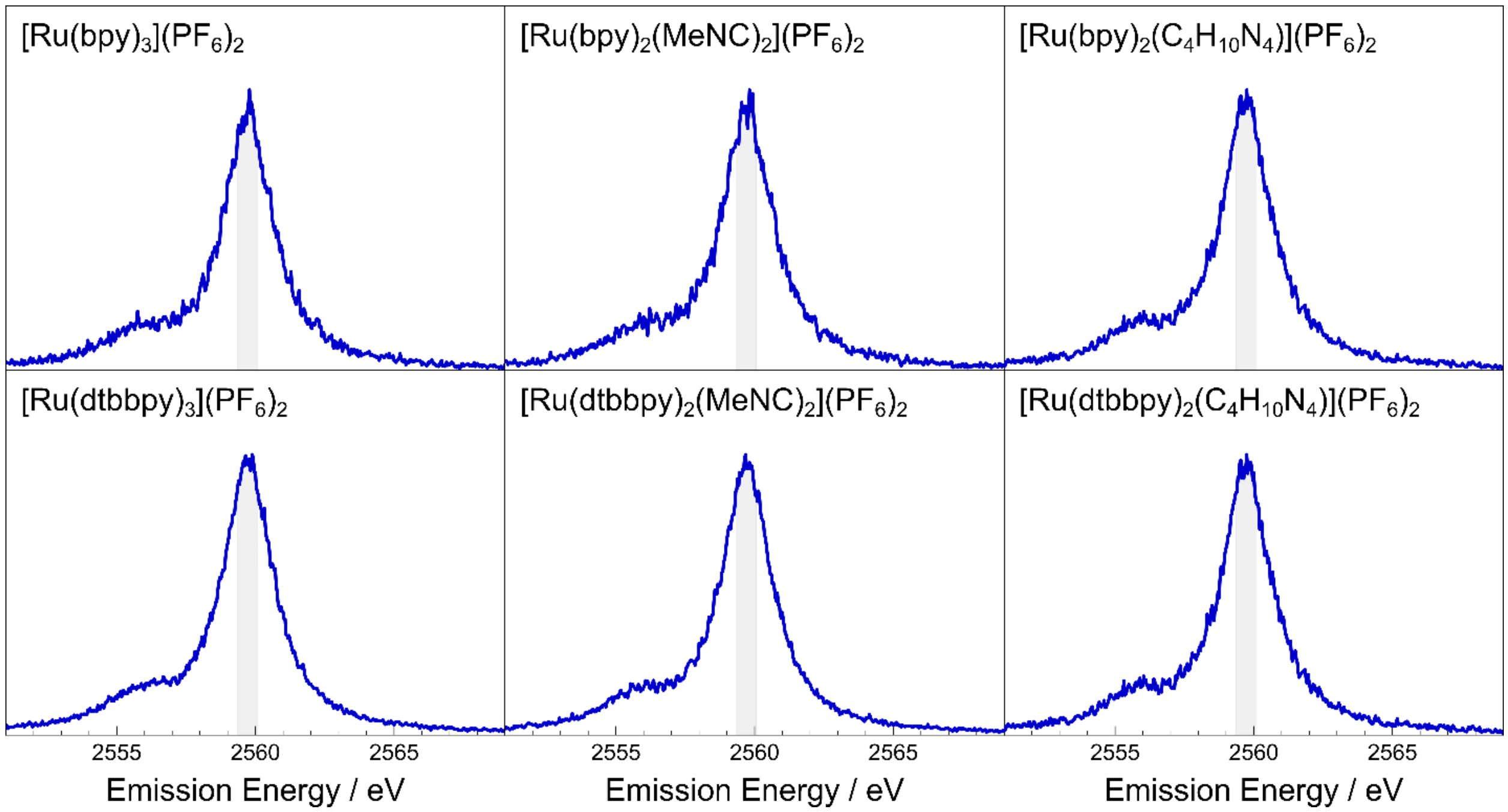

Figure S27. Non-resonant Ru Lα_1 X-ray emission spectra for investigated complexes, obtained by integrating the emission signal over incident X-ray energies above 2860 eV. Gray shaded area represents data used to generate HERFD XAS spectra.

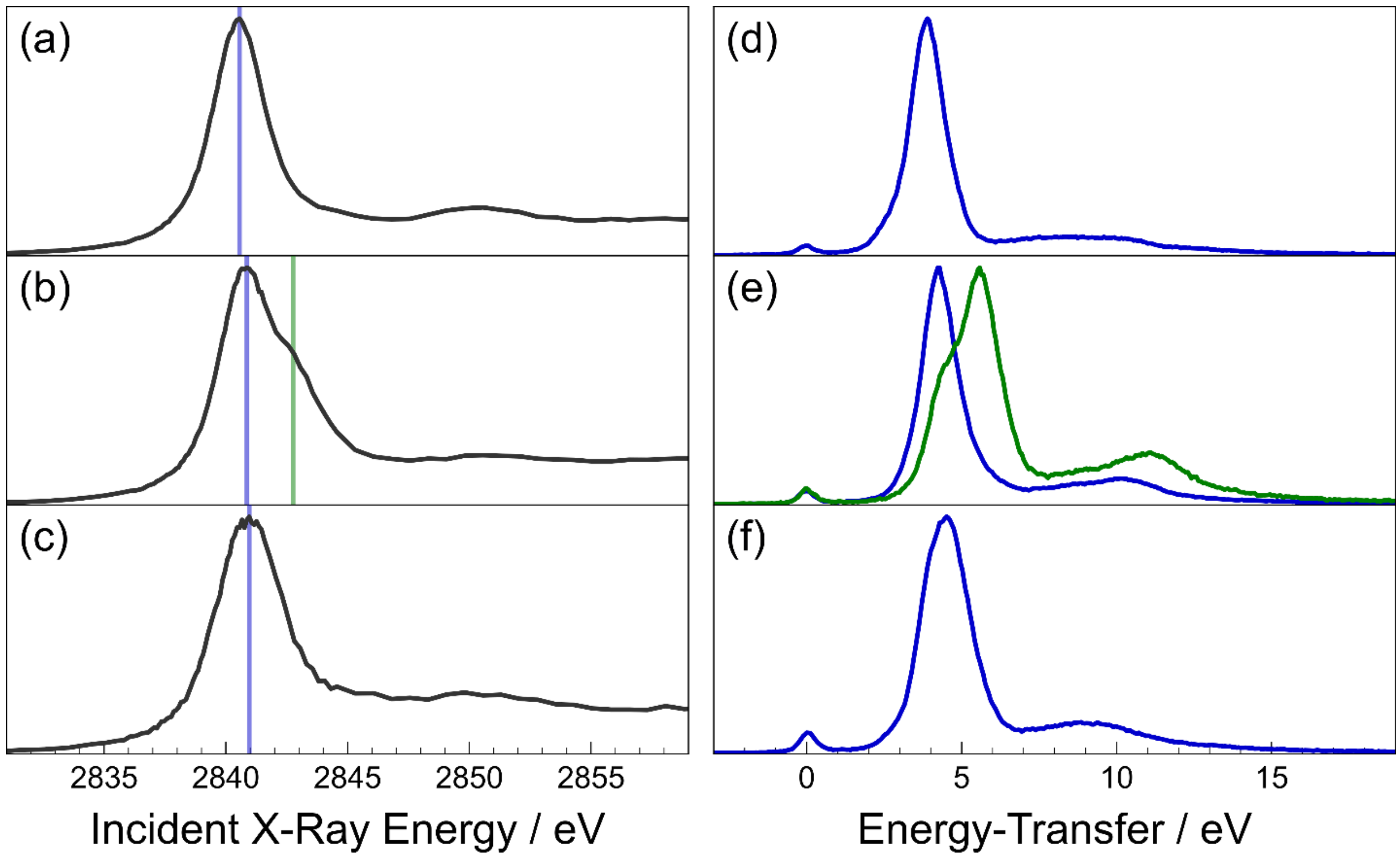

Figure S28. Ru L_3-edge X-ray absorption spectra recorded as PFY of (a) $[Ru(dtbbpy)_3](PF_6)_2$, (b) $[Ru(dtbbpy)_2(MeNC)_2](PF_6)_2$ and (c) $[Ru(dtbbpy)_2(C_4H_{10}N_4)](PF_6)_2$, and Ru 2p4d resonant inelastic X-ray scattering of (d) $[Ru(dtbbpy)_3](PF_6)_2$, (e) $[Ru(dtbbpy)_2(MeNC)_2](PF_6)_2$ and (f) $[Ru(dtbbpy)_2(C_4H_{10}N_4)](PF_6)_2$. Vertical lines in panels a-c correspond to incident X-ray energies for panels d-f, respectively.

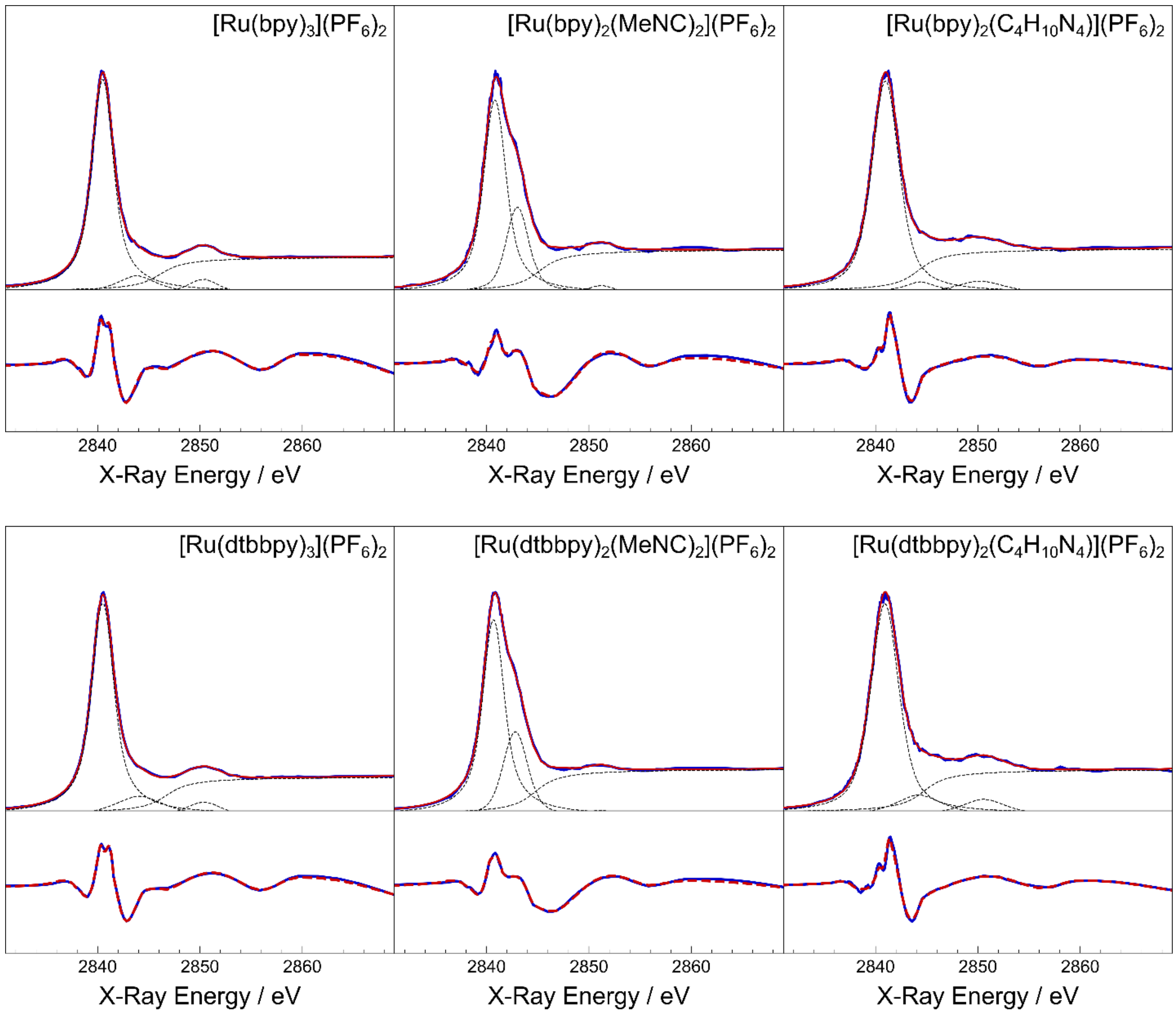

Figure S29. Ru L_3-edge XAS spectra recorded as PFY (solid blue traces) of investigated complexes with negative second derivative spectra below, and corresponding fits to a series of pseudo-Voigt peaks (red dashed traces) and individual fit contributions (black dashed traces). The edge-jump is modelled using an arctangent function. The Lorentzian width of pseudo-Voigt bands and the edge-jump are fixed to the lifetime of the Ru 2p core hole of 1.75 eV.[44]

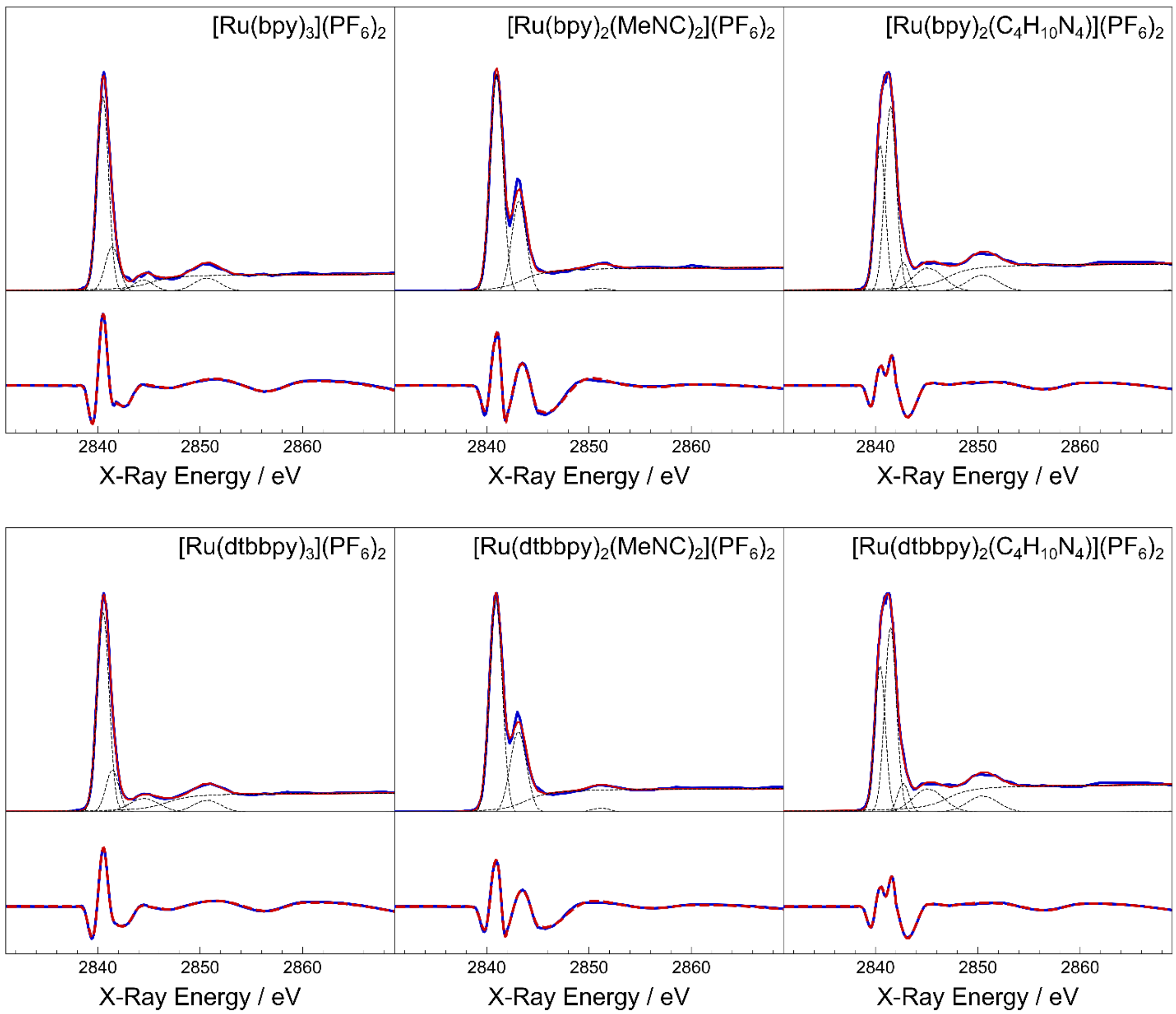

Figure S30. Ru L_3-edge XAS spectra recorded as HERFD (solid blue traces) of investigated complexes with negative second derivative spectra below, and corresponding fits to a series of Gaussian peaks (red dashed traces) and individual fit contributions (black dashed traces). The edge-jump is modelled using an arctangent function, with width fixed to the lifetime of the Ru 2p core hole of 1.75 eV.[44]

Table S12. Transition energies and full-width half-maxima derived from least squares fitting of the main Ru L_3-edge PFY XAS features to a series of pseudo-Voigt peaks. Uncertainties are reported to the 95% confidence interval.

	e_g* Resonance		t_{2g}* Resonance	
Complex	E / eV	FWHM / eV	E / eV	FWHM / eV
$[Ru(bpy)_3](PF_6)_2$	2840.5(1)	2.03(4)	2845(1)	7(5)
$[Ru(bpy)_2(MeNC)_2](PF_6)_2$	2840.8(1)	2.2(2)	2843.0(5)	2.3(6)
$[Ru(bpy)_2(C_4H_{10}N_4)](PF_6)_2$	2841.0(1)	3.00(9)	2844(3)	2(3)
$[Ru(dtbbpy)_3](PF_6)_2$	2840.5(1)	2.10(3)	2844.6(5)	7(4)
$[Ru(dtbbpy)_2(MeNC)_2](PF_6)_2$	2840.7(1)	2.02(6)	2842.8(4)	2.6(6)
$[Ru(dtbbpy)_2(C_4H_{10}N_4)](PF_6)_2$	2840.7(2)	2.7(2)	2841.7(4)	8(2)

Table S13. Transition energies and full-width half-maxima derived from least squares fitting of the main Ru L_3-edge HERFD XAS features to a series of pseudo-Voigt peaks. Uncertainties are reported to the 95% confidence interval.

	e_g* Resonance		t_{2g}* Resonance	
Complex	E / eV	FWHM / eV	E / eV	FWHM / eV
$[Ru(bpy)_3](PF_6)_2$	2840.1(1)	1.32(3)	2841.1(2)	1.3(2)
$[Ru(bpy)_2(MeNC)_2](PF_6)_2$	2840.5(1)	1.46(2)	2842.7(1)	1.62(9)
$[Ru(bpy)_2(C_4H_{10}N_4)](PF_6)_2$	2840.0(1)<br>2841.1(1)	1.21(5)<br>1.3(2)	2842.3(1)	1.0(2)
$[Ru(dtbbpy)_3](PF_6)_2$	2840.1(1)	1.4(1)	2841(2)	1.7(9)
$[Ru(dtbbpy)_2(MeNC)_2](PF_6)_2$	2840.5(1)	1.47(2)	2842.7(1)	1.84(8)
$[Ru(dtbbpy)_2(C_4H_{10}N_4)](PF_6)_2$	2840.0(1)<br>2841.1(1)	1.21(5)<br>1.3(2)	2842.3(1)	1.0(2)

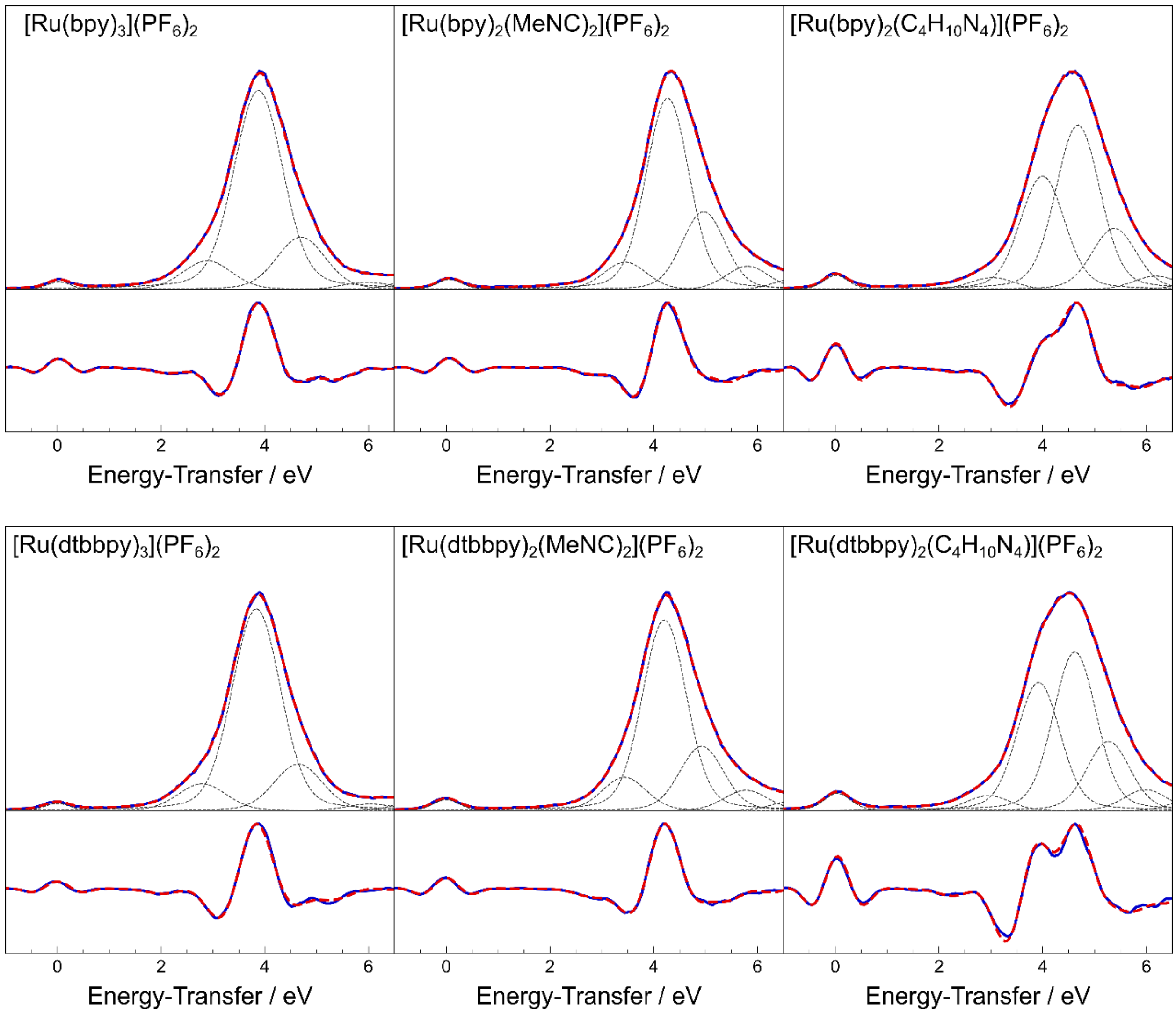

Figure S31. Ru 2p4d RIXS spectra (solid blue traces) of investigated complexes with negative second derivative spectra below, and corresponding fits to a series of pseudo-Voigt peaks (red dashed traces) and individual fit contributions (black dashed traces).

Table S14. Term energies derived from least squares fitting of the main Ru 2p4d RIXS features to a series of pseudo-Voigt peaks.

Complex	$^3T_{1g}$	$^1T_{1g}$ / $^3T_{2g}$	$^1T_{2g}$
$[Ru(bpy)_3](PF_6)_2$	2.860(9)	3.846(3)	4.669(9)
$[Ru(bpy)_2(MeNC)_2](PF_6)_2$	3.39(2)	4.204(2)	4.898(3)
$[Ru(bpy)_2(C_4H_{10}N_4)](PF_6)_2$	3.02(2)	3.981(5)<br>4.664(7) [a]	5.37(2)
$[Ru(dtbbpy)_3](PF_6)_2$	2.791(8)	3.837(2)	4.647(8)
$[Ru(dtbbpy)_2(MeNC)_2](PF_6)_2$	3.43(2)	4.206(4)	4.93(2)
$[Ru(dtbbpy)_2(C_4H_{10}N_4)](PF_6)_2$	2.91(2)	3.878(5)<br>4.577(9) [a]	5.23(2)

[a] In the Chugaev-type dicarbene complexes, the nominally $^1T_{1g}$ / $^3T_{2g}$ band is split into two distinct peaks

Photolysis Studies

Photolysis of the homoleptic and Chugaev-type dicarbene complexes was investigated by irradiating acetonitrile solutions of the investigated complexes at 450 nm with the monochromated output (10 nm slit width) of a 450 W xenon lamp and recording UV-vis and emission (λ_{ex} = 450 nm) spectra at 5 min intervals (Figure S32).

For all complexes except $[Ru(dtbbpy)_3](PF_6)_2$, SVD of the recorded UV-vis and emission spectra of the investigated complexes indicate two components contributing to the change in UV-vis spectra, and one to the change in photoluminescence spectra as a function of irradiation time (Figure S33-Figure S36). For $[Ru(dtbbpy)_3](PF_6)_2$, there was insufficient change in the UV-vis spectrum over the time course of the experiment to identify a second component.

The SVD analysis is suggestive of an R→P (reactant→photoproduct) photoreaction, whereby the reactant is luminescent and the photoproduct is not.

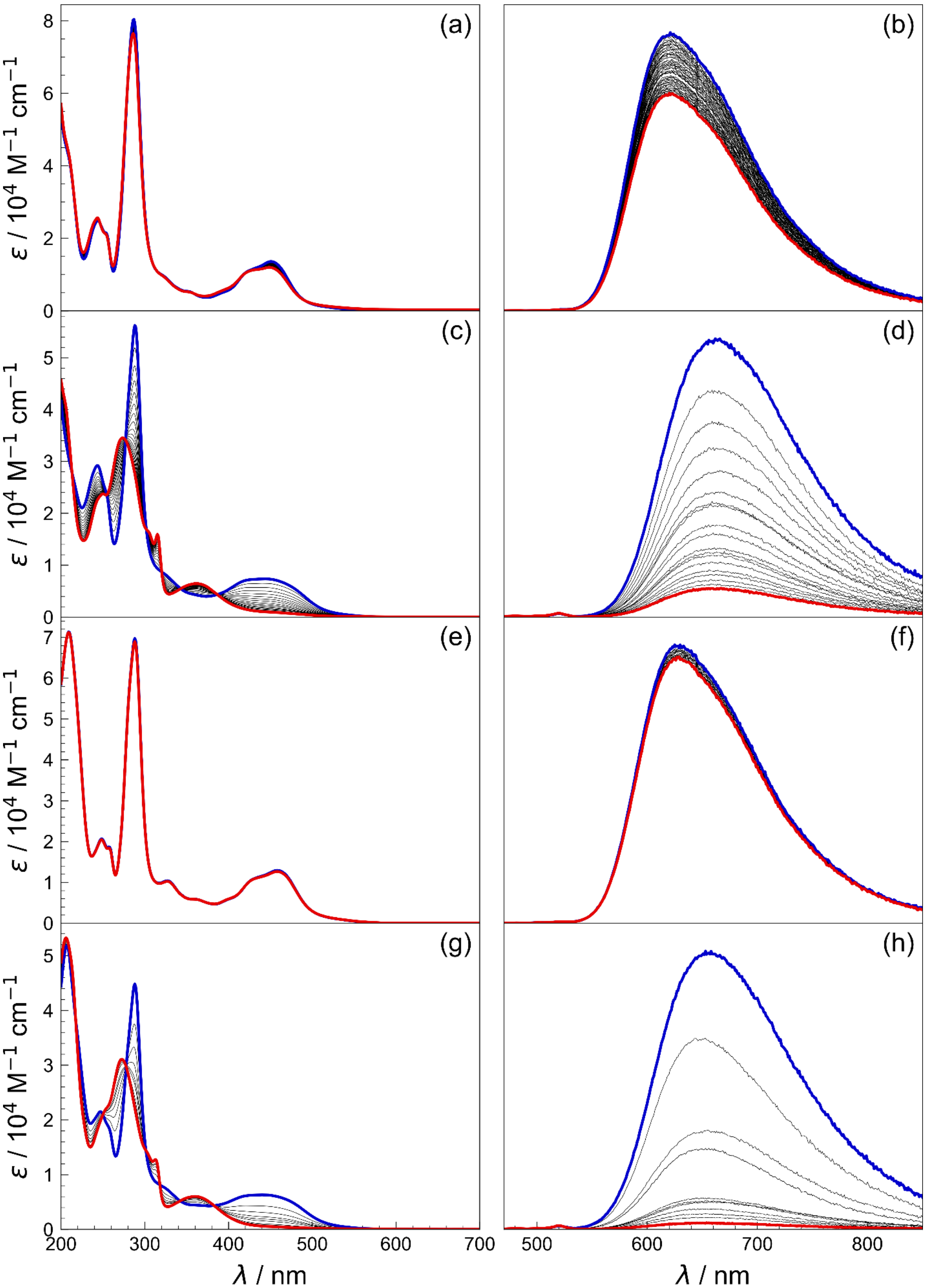

Figure S32. UV-vis electronic absorption (a,c,e,g) and emission (b,d,f,h, λ_{ex} = 450 nm) spectra of de-aerated acetonitrile solutions of $[Ru(bpy)_3](PF_6)_2$ (a,b), $[Ru(bpy)_2(C_4H_{10}N_4)](PF_6)_2$ (c,d), $[Ru(dtbbpy)_3](PF_6)_2$ (e,f), and$[Ru(dtbbpy)_2(C_4H_{10}N_4)](PF_6)_2$ (g,h) upon irradiation with 450 nm light. Solutions had an initial optical density of 0.1 at 450 nm. Spectra were recorded in 5 min intervals. Blue traces are the initial spectrum at t = 0 min, and red traces the final spectra.

For R→P photoreactions, the rate of change in concentration of the reactant can be described by:[45, 46]

$$-\frac{\partial[R]}{\partial t} = q_V \cdot \phi_q \cdot (1 - 10^{-A_t}) \cdot \frac{\varepsilon_R l[R]}{A_t} \qquad Eq.S18a$$

$$\ln\left(\frac{[R]}{[R]_0}\right) = -q_V \cdot \phi_q \cdot \varepsilon_R \cdot l \cdot \int_{t=0}^{t} \left[\frac{1 - 10^{-A_t}}{A_t}\right] dt \qquad Eq.S18b$$

Where ϕ_{PR} is the quantum yield of the photoreaction, q_V the incident volumetric photon flux (in einstein L^{-1} s^{-1}), A_t the absorbance at the irradiation wavelength at a given time, ε_R the molar absorption coefficient of the reactant at the irradiation wavelength, and *l* the optical pathlength. For ease of analysis, Eq. S18b can be generalized to:

$$\frac{[R]}{[R]_0} = e^{-k \cdot \varepsilon_R \cdot f(t)} \qquad Eq.S19a$$

$$f(t) = \int_{t=0}^{t} \left[\frac{1 - 10^{-A_t}}{A_t}\right] dt \qquad Eq.S19b$$

$$k = \phi_{PR} \cdot \phi_q \cdot l \qquad Eq.S19c$$

Because the photoproduct is non-luminescent, $\frac{[R]}{[R]_0} = \frac{I}{I_0}$, where I is the integrated emission intensity. $\frac{[R]}{[R]_0}$ can also be obtained from the right singular vectors in our SVD analysis. The only parameters not directly measured in the photoirradiation experiment are ϕ_{PR} and ϕ_q. ϕ_q can be determined through several methods, including measurement of optical power or through chemical actinometry. We chose to use a relative actinometric approach, using $[Ru(bpy)_3](PF_6)_2$ as a reference compound with a reported ϕ_{PR} of 0.0276%.[47] Using this approach:

$$\phi_{PR}(sample) = \phi_{PR}(reference)\frac{k(sample)}{k(reference)} \qquad Eq.S20$$

Simultaneous fitting of the basis kinetic traces from SVD decomposition of both the UV-vis and emission spectra of $[Ru(bpy)_3](PF_6)_2$ to the same kinetic model (Eq. S19, Figure S33-Figure S36) yields the parameters reported in Table S15.

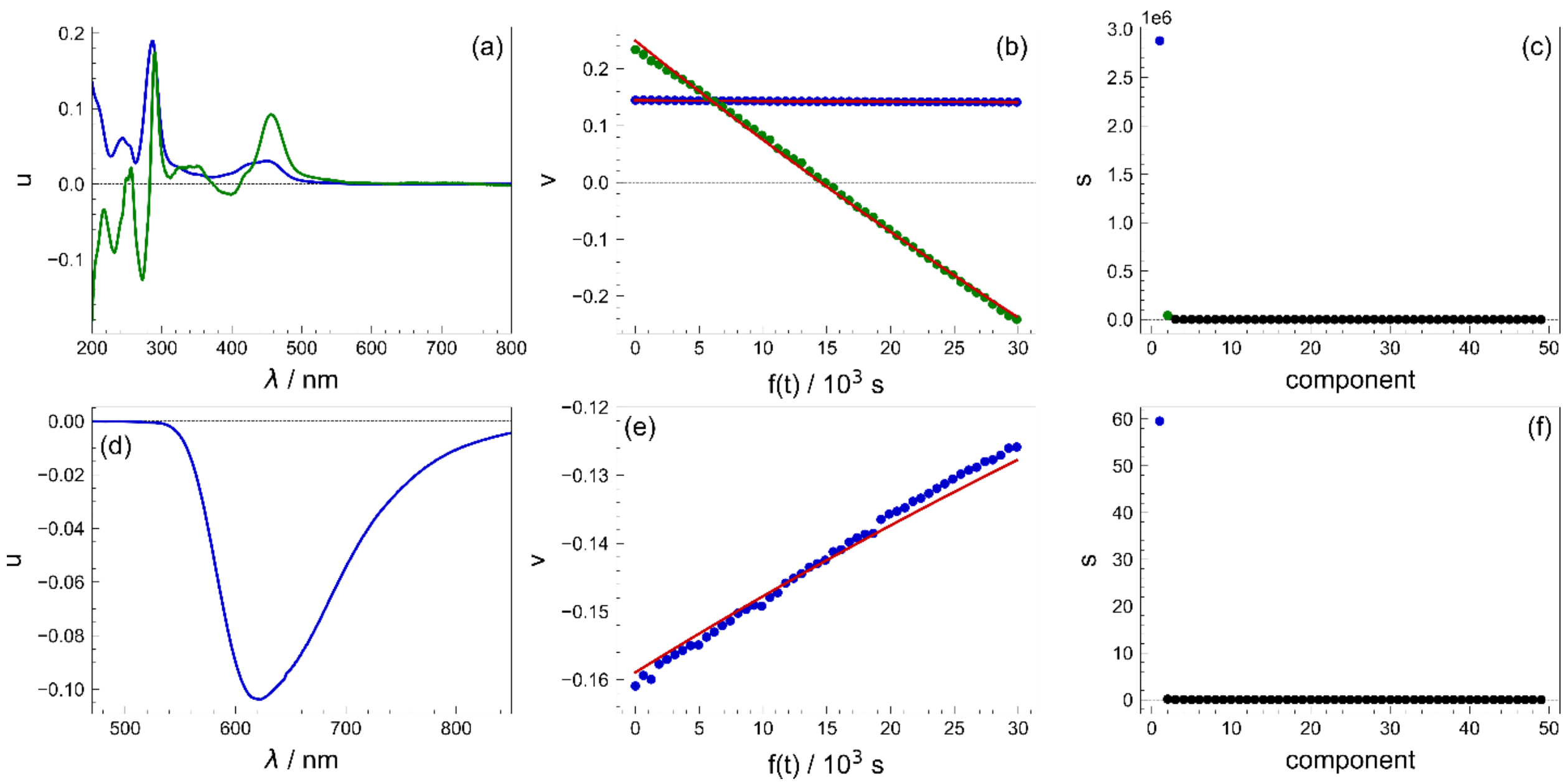

Figure S33. SVD and global fitting of the UV-vis (a-c) and emission (d-f) spectra of $[Ru(bpy)_3](PF_6)_2$ in de-aerated acetonitrile (λ_{ex} = 450 nm) upon irradiation with 450 nm light (Figure S32). (a,d) First (blue) and second (red) left singular vectors (basis spectra); (b,e) First (blue points) and second (red points) right singular vectors, and corresponding fits to a two-state kinetic model (Eq. S19, solid traces); (c,f) singular values. The colored singular values in panels (c) and (f) correspond to the colored left and right singular vectors in panels (a) and (b), and (d) and (e), respectively.

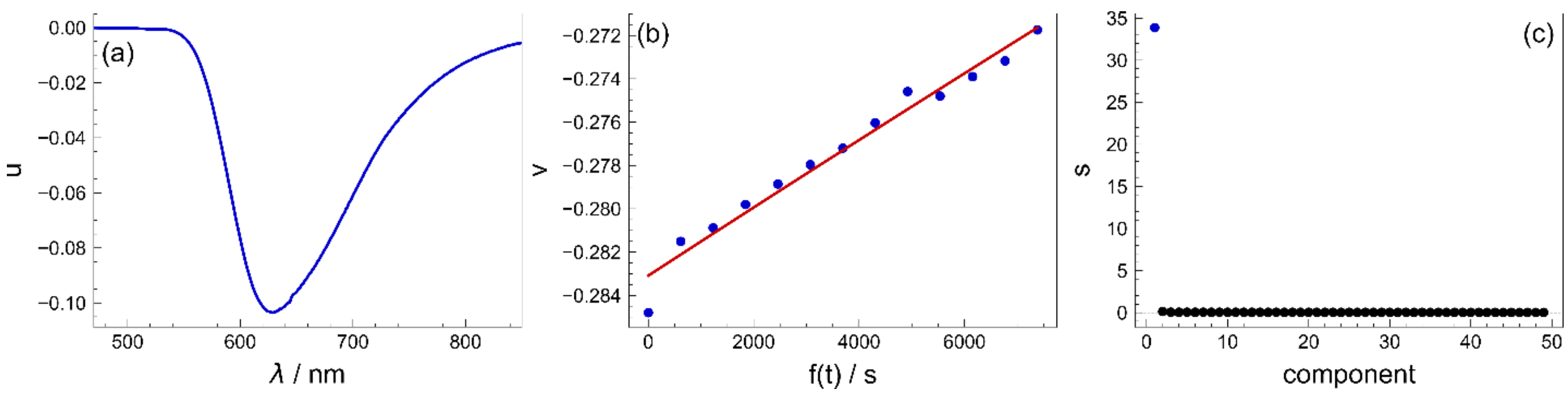

Figure S34. SVD and global fitting of the emission spectra of $[Ru(dtbbpy)_3](PF_6)_2$ in de-aerated acetonitrile (λ_{ex} = 450 nm) upon irradiation with 450 nm light (Figure S32). (a) First (blue) and second (red) left singular vectors (basis spectra); (b) First (blue points) and second (red points) right singular vectors, and corresponding fits to a two-state kinetic model (Eq. S19, solid traces); (c) singular values.

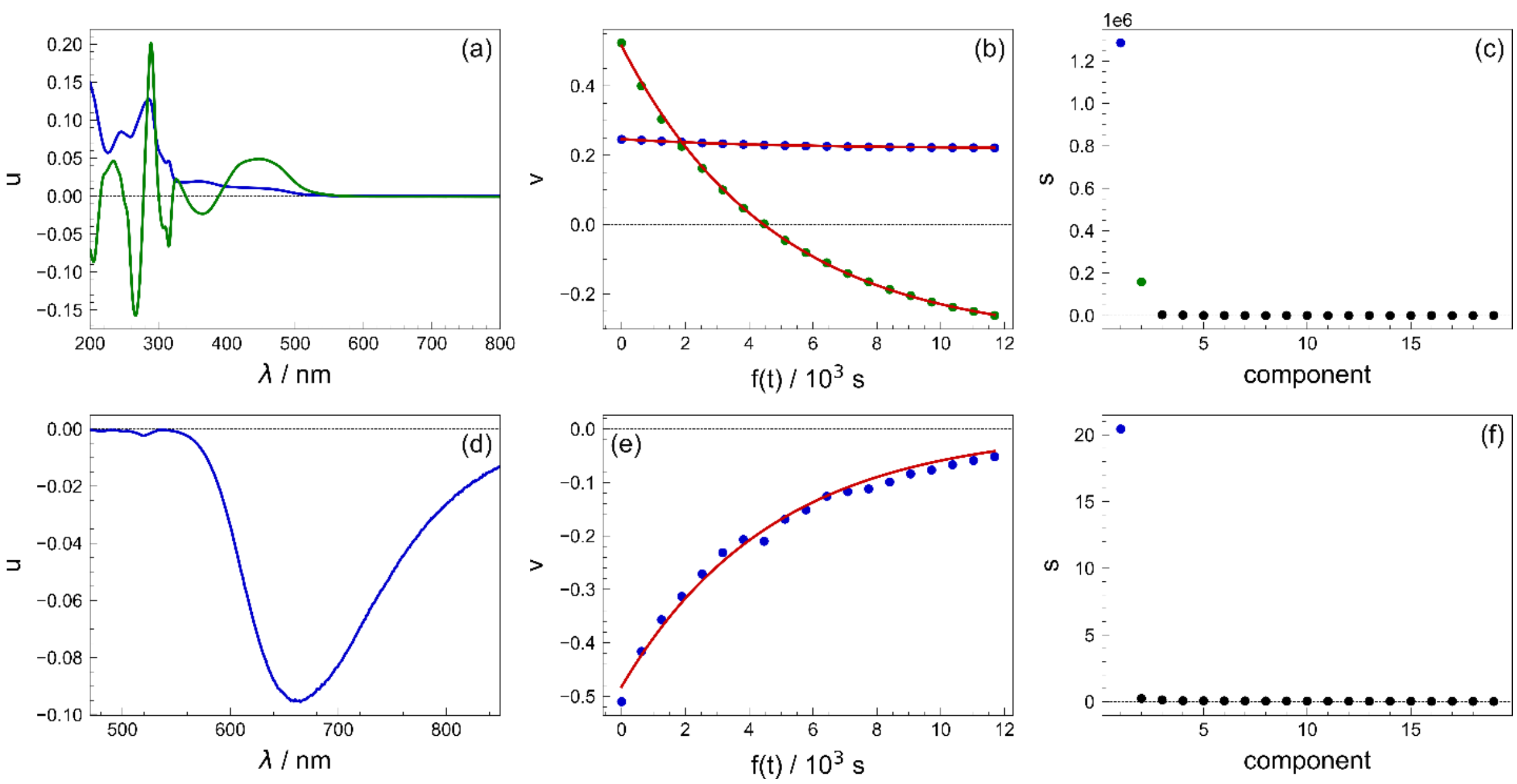

Figure S35. SVD and global fitting of the UV-vis (a-c) and emission (d-f) spectra of $[Ru(bpy)_2(C_4H_{10}N_4)](PF_6)_2$ in de-aerated acetonitrile (λ_{ex} = 450 nm) upon irradiation with 450 nm light (Figure S32). (a,d) First (blue) and second (red) left singular vectors (basis spectra); (b,e) First (blue points) and second (red points) right singular vectors, and corresponding fits to a two-state kinetic model (Eq. S19, solid traces); (c,f) singular values. The colored singular values in panels (c) and (f) correspond to the colored left and right singular vectors in panels (a) and (b), and (d) and (e), respectively.

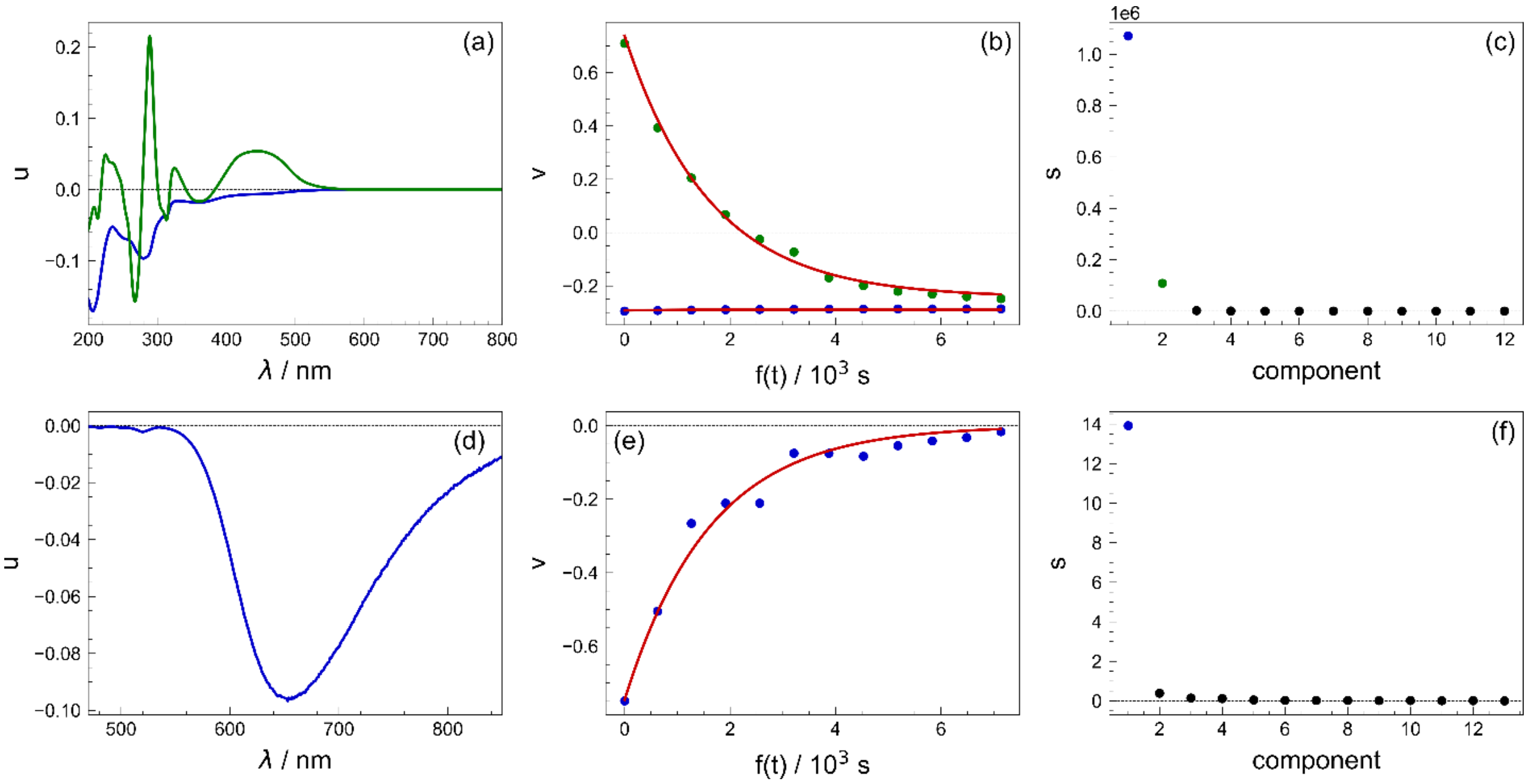

Figure S36. SVD and global fitting of the UV-vis (a-c) and emission (d-f) spectra of $[Ru(dtbbpy)_2(C_4H_{10}N_4)](PF_6)_2$ in de-aerated acetonitrile (λ_{ex} = 450 nm) upon irradiation with 450 nm light (Figure S32). (a,d) First (blue) and second (red) left singular vectors (basis spectra); (b,e) First (blue points) and second (red points) right singular vectors, and corresponding fits to a two-state kinetic model (Eq. S19, solid traces); (c,f) singular values. The colored singular values in panels (c) and (f) correspond to the colored left and right singular vectors in panels (a) and (b), and (d) and (e), respectively.

Table S15. Photoreaction quantum yields obtained from global fitting analysis of the UV-vis and emission spectra of investigated complexes in de-aerated acetonitrile (λ_{ex} = 450 nm) upon irradiation with 450 nm light to a two-state kinetic model (Eq. S20).

Complex	k / 10^{-10} E cm s^{-1}	ε_{450} / 10^3 M^{-1} cm^{-1}	ϕ_{PR} / %
$[Ru(bpy)_3](PF_6)_2$	5.36	13.6	0.0276 [a]
$[Ru(bpy)_2(C_4H_{10}N_4)](PF_6)_2$	287	7.31	1.48
$[Ru(dtbbpy)_3](PF_6)_2$	4.51	12.4	0.0232
$[Ru(dtbbpy)_2(C_4H_{10}N_4)](PF_6)_2$	991	6.24	5.11

[a] From reference [47]

From the global fit results described above, we were able to construct species associated spectra and irradiation time-dependent relative concentrations of the reactant and photoproduct using an analogous approach as in the Molar Absorption Coefficient Determination section (Figure S37-Figure S38, and Figure 7 in the main paper).

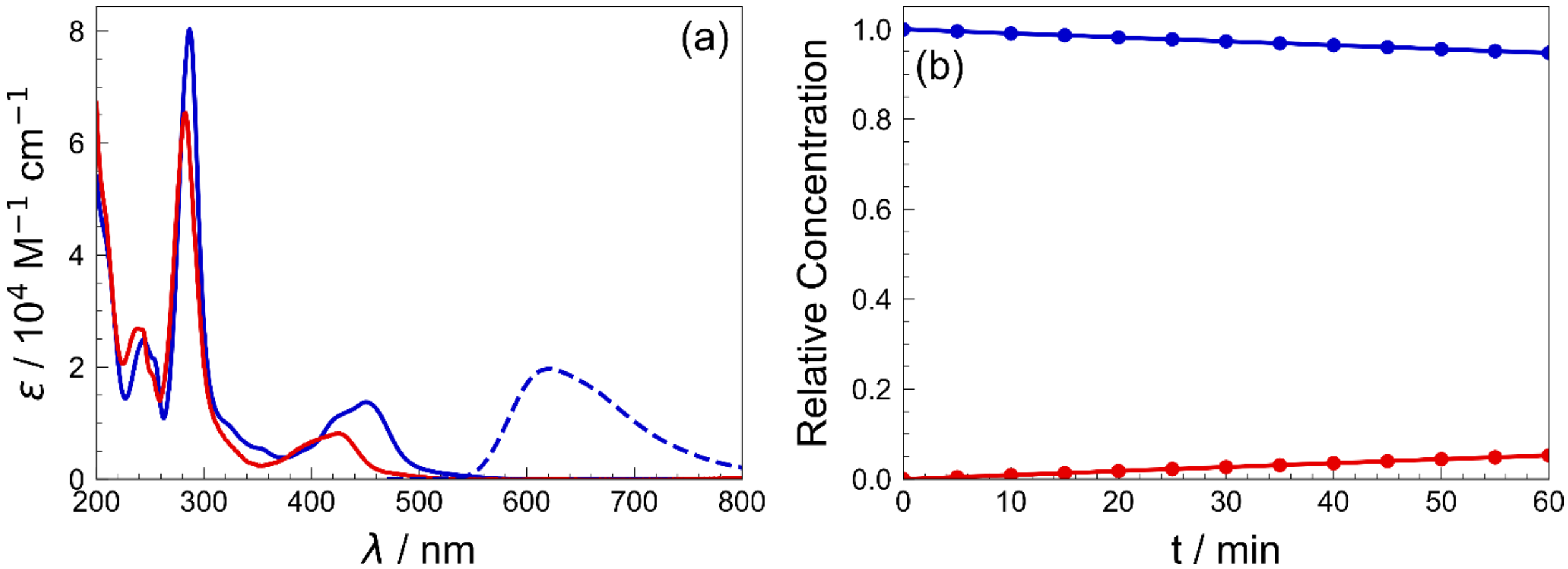

Figure S37. (a) Species associated spectra of reactant (solid blue trace = absorption SAS, dashed blue trace = emission SAS) and photoproduct (solid red trace = absorption SAS) obtained from global fitting of the UV-vis and emission spectra of $[Ru(bpy)_3](PF_6)_2$ in de-aerated acetonitrile (λ_{ex} = 450 nm) upon irradiation with 450 nm light (Figure S32 and Figure S33). (b) Relative concentrations of reactant and photoproduct over the course of the reaction obtained from global fitting.

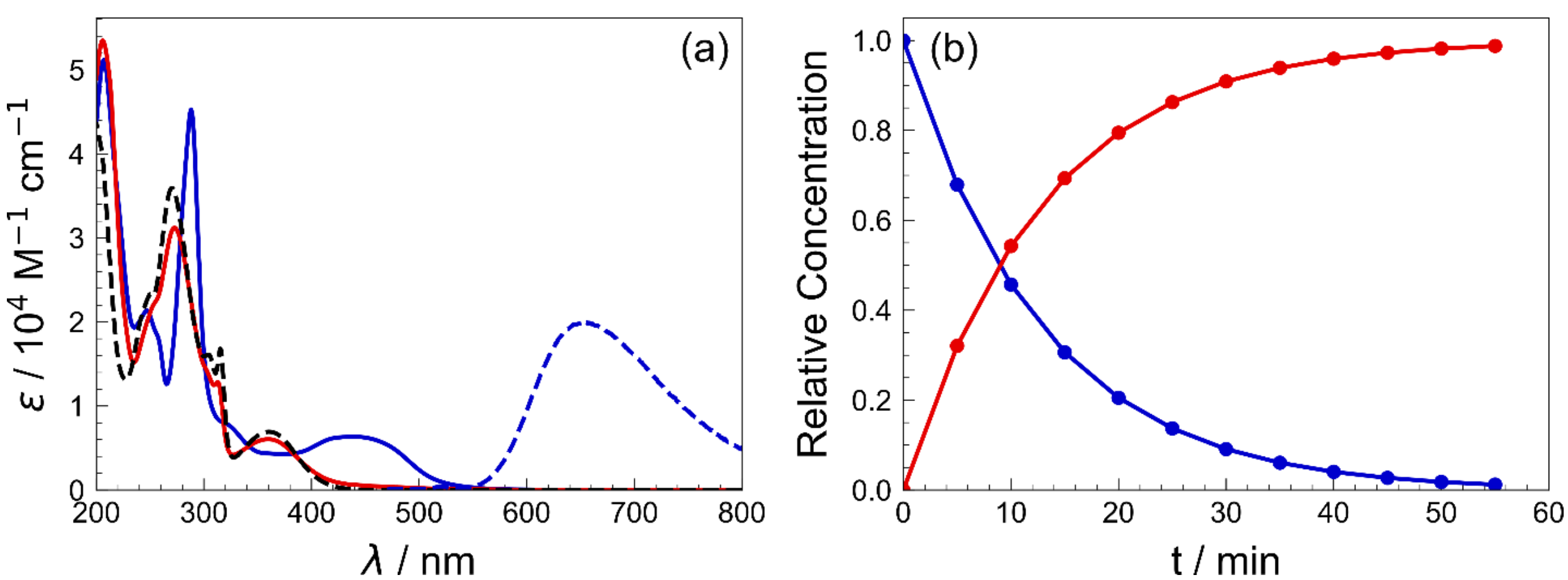

Figure S38. (a) Species associated spectra of reactant (solid blue trace = absorption SAS, dashed blue trace = emission SAS) and photoproduct (solid red trace = absorption SAS) obtained from global fitting of the UV-vis and emission spectra of $[Ru(dtbbpy)_2(C_4H_{10}N_4)](PF_6)_2$ in de-aerated acetonitrile (λ_{ex} = 450 nm) upon irradiation with 450 nm light (Figure S32 and Figure S36). Black dashed trace is the UV-vis electronic absorption spectrum of $[Ru(dtbbpy)_2(MeNC)_2](PF_6)_2$ from Figure 2 of the main paper. (b) Relative concentrations of reactant and photoproduct over the course of the reaction obtained from global fitting.

To unambiguously assign the photoproduct, photodecomposition of $[Ru(bpy)_2(C_4H_{10}N_4)](PF_6)_2$ was further investigated by irradiating a perdeuterated acetonitrile solution (10 mg mL^{-1}) in a homebuilt photoreactor comprising a 3 m strip of 460 nm LEDs, outputting an average power of 20 mW cm^{-2} at 460 nm. ^{1}H NMR spectra were then recorded at 10 min intervals (Figure 6 in the main paper, Figure S39-Figure S41).

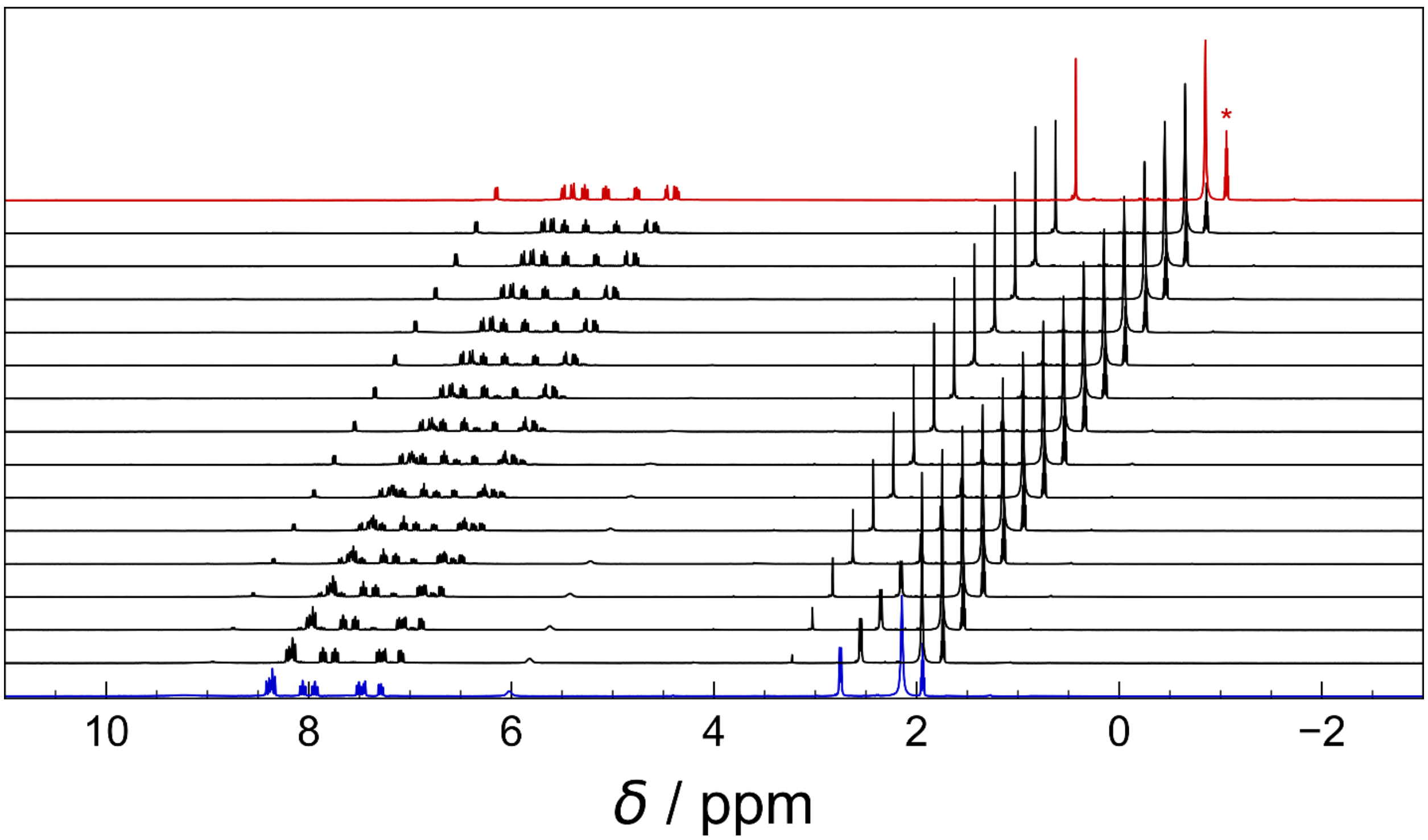

Figure S39. ^{1}H NMR spectra of $[Ru(bpy)_2(C_4H_{10}N_4)](PF_6)_2$ in CD_3CN upon irradiation with 460 nm light, showing full chemical shift range. Spectra were recorded every 10 min for 150 min. The blue trace is the initial spectrum at t = 0 min, and the red trace the final spectrum at t = 150 min. Asterisk denotes residual non-perdeuterated solvent used as an internal reference.

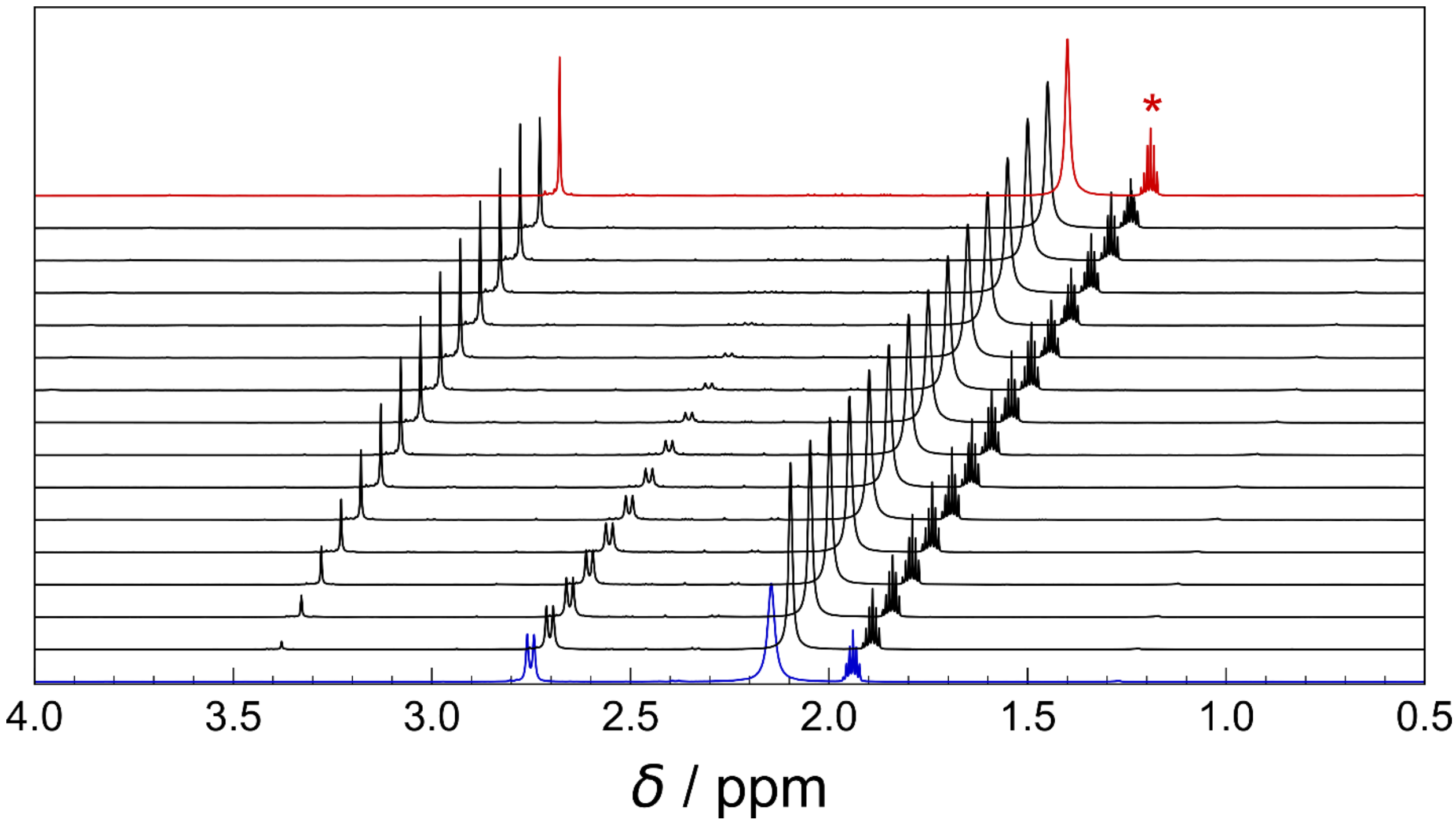

Figure S40. ^{1}H NMR spectra of $[Ru(bpy)_2(C_4H_{10}N_4)](PF_6)_2$ in CD_3CN upon irradiation with 460 nm light, focusing on high-field peaks. Spectra were recorded every 10 min for 150 min. The blue trace is the initial spectrum at t = 0 min, and the red trace the final spectrum at t = 150 min. Asterisk denotes residual non-perdeuterated solvent used as an internal reference.

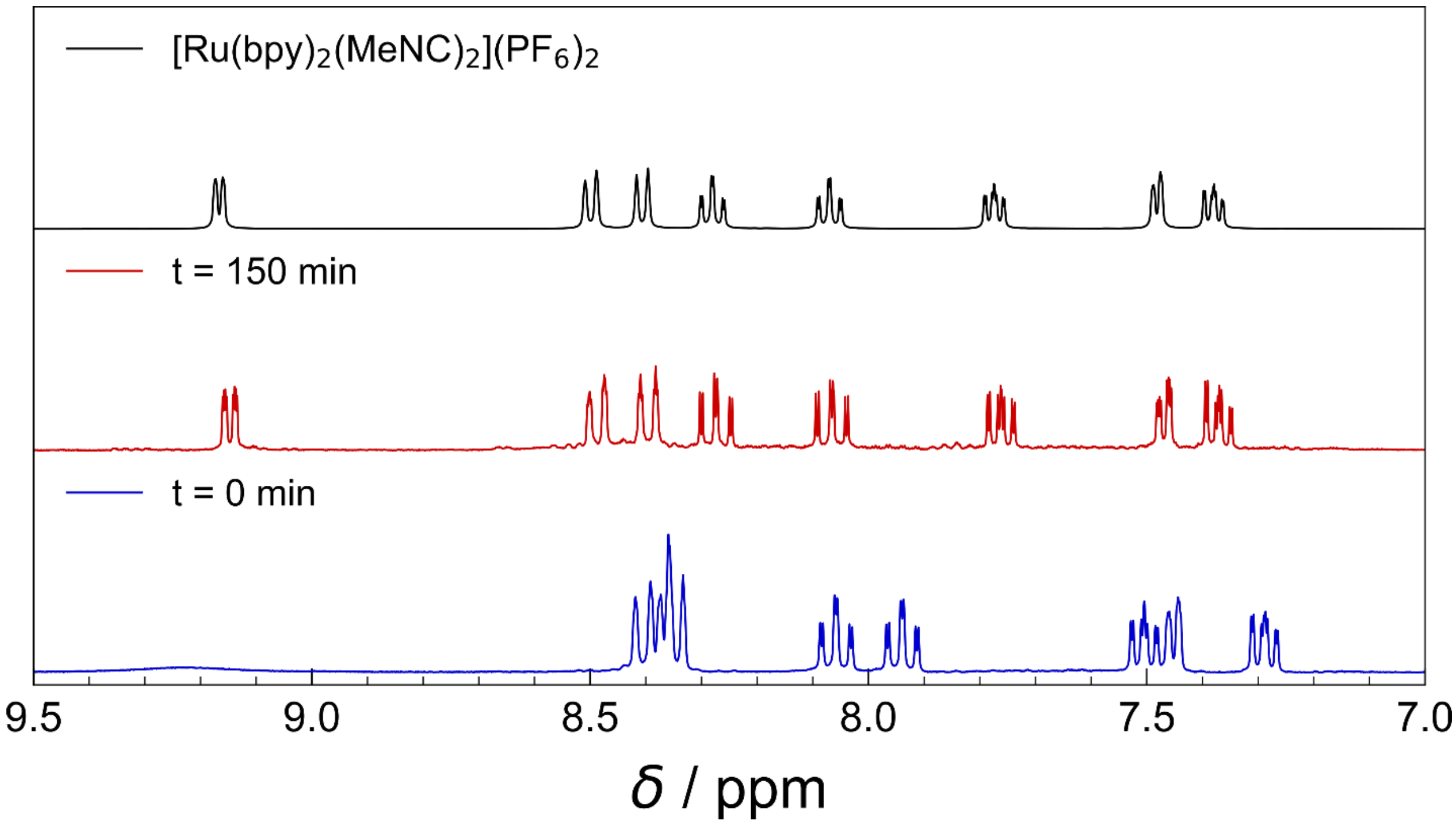

Figure S41. ^{1}H NMR spectra of $[Ru(bpy)_2(C_4H_{10}N_4)](PF_6)_2$ in CD_3CN before and after photoirradiation with 460 nm light, focusing on low-field (aromatic) peaks, and comparison with independently recorded $[Ru(bpy)_2(MeNC)_2](PF_6)_2$.

Unlike with optical spectroscopies, kinetic analysis cannot be performed on the NMR photoirradiation data, as the concentrations required to obtain satisfactory signal result in extremely high optical densities and consequently a strong primary inner filter effect and non-uniform irradiation. This results in retardation of early kinetics (Figure S42), deviating from the first order kinetics described in Eq. S19.

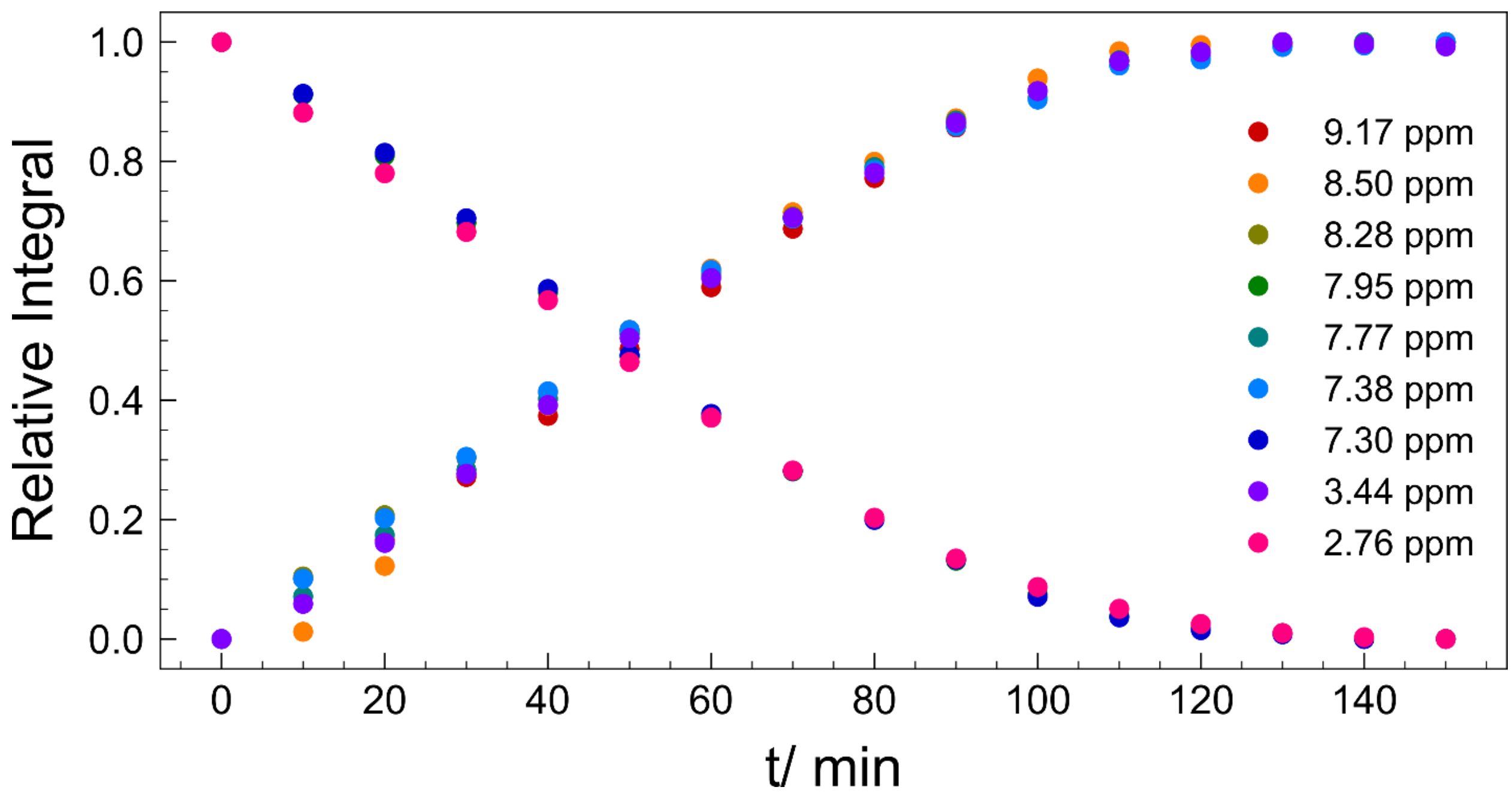

Figure S42. Relative integrals of selected ^{1}H NMR peaks over the course of the photoreaction.

Although it is challenging to apply a specific kinetic model to the NMR photoirradiation data, the uniform response of the relative ^{1}H NMR signals in Figure S42, and clean spectral changes in Figure S39-Figure S41 inspired us to perform a global fitting analysis instead by fitting the basis spectra (left singular vectors) to linear combinations of the ^{1}H NMR spectra of $[Ru(bpy)_2(C_4H_{10}N_4)](PF_6)_2$ and $[Ru(bpy)_2(MeNC)_2](PF_6)_2$ (Figure S43a-b). This yields the irradiation time-dependent relative concentrations of $[Ru(bpy)_2(C_4H_{10}N_4)](PF_6)_2$ and $[Ru(bpy)_2(MeNC)_2](PF_6)_2$ in Figure S43d.

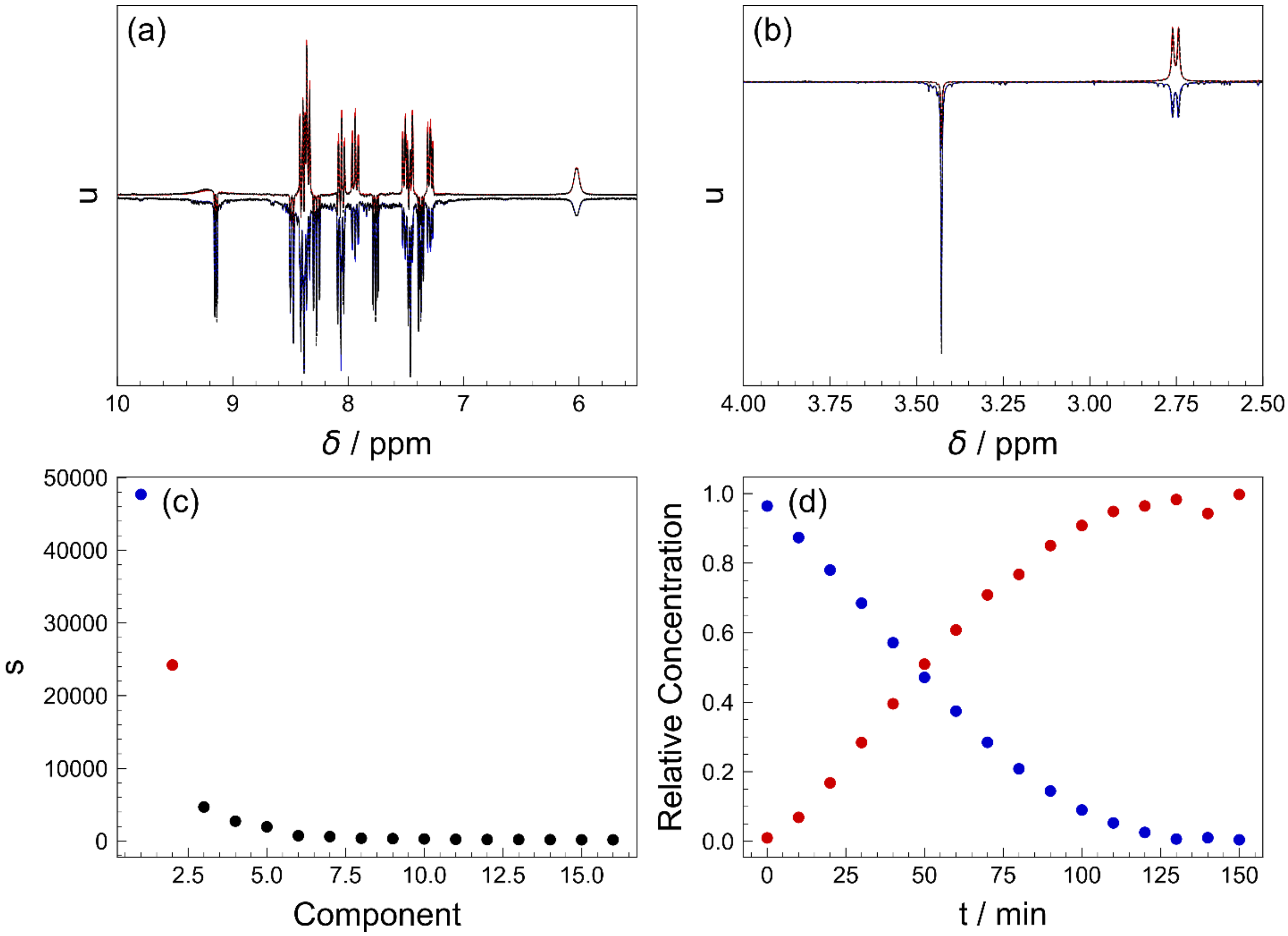

Figure S43. SVD and global fitting of the ^{1}H NMR spectra of $[Ru(bpy)_2(C_4H_{10}N_4)](PF_6)_2$ in CD_3CN upon irradiation with 460 nm light. First (solid blue traces) and second (solid red traces) left singular vectors (basis spectra), with fits to linear combinations of $[Ru(bpy)_2(C_4H_{10}N_4)](PF_6)_2$ and $[Ru(bpy)_2(MeNC)_2](PF_6)_2$ (black dashed traces), focusing on (a) the low-field peaks and (b) the high-field peaks; (c) singular values; (d) relative concentrations of $[Ru(bpy)_2(C_4H_{10}N_4)](PF_6)_2$ (blue dots) and $[Ru(bpy)_2(MeNC)_2](PF_6)_2$ (red dots) over the course of the photoreaction obtained from global fitting.

Computational Results

For all calculations, motivated by existing literature on Ru polypyridyl complexes,[48-51] we utilized the B3LYP functional with the def2-TZVP(-f) basis set (def2-ECP for Ru) and Grimme's D3-BJ dispersion correction, with an acetonitrile CPCM solvation model. As, in this work, we are not seeking to use DFT simulations to compare bpy versus dtbbpy systems, but rather to gain insight into why the Chugaev-type dicarbene complexes undergo hydrazine photorelease, we only computationally studied the simpler bpy systems.

For triplet state calculations, orbitals are labelled based on ground state assignments – i.e. the two singly occupied molecular orbitals are thus denoted HOMOα and LUMOα (Table S22-Table S24).

As the experimental approaches of comparing the energy difference between ^{3}MLCT and ^{3}LF states reported in the main paper do not probe potential energy surface minima, we sought to optimize the ^{3}LF structures using the approach described by Dixon and coworkers.[48, 49] Following this approach, we were able to optimize the geometry of the *trans* ^{3}LF state for both $[Ru(bpy)_3]^{2+}$ and $[Ru(bpy)_2(MeNC)_2]^{2+}$ (Table S20). However, multiple attempts at optimizing both the *trans* and *cis* ^{3}LF states of $[Ru(bpy)_2(C_4H_{10}N_4)]^{2+}$ were unsuccessful, consistently converging to the lowest energy ^{3}MLCT state – precluding comparison of ΔE_b (from Figure 3 in the main paper).

Ground State (S_0) DFT Results

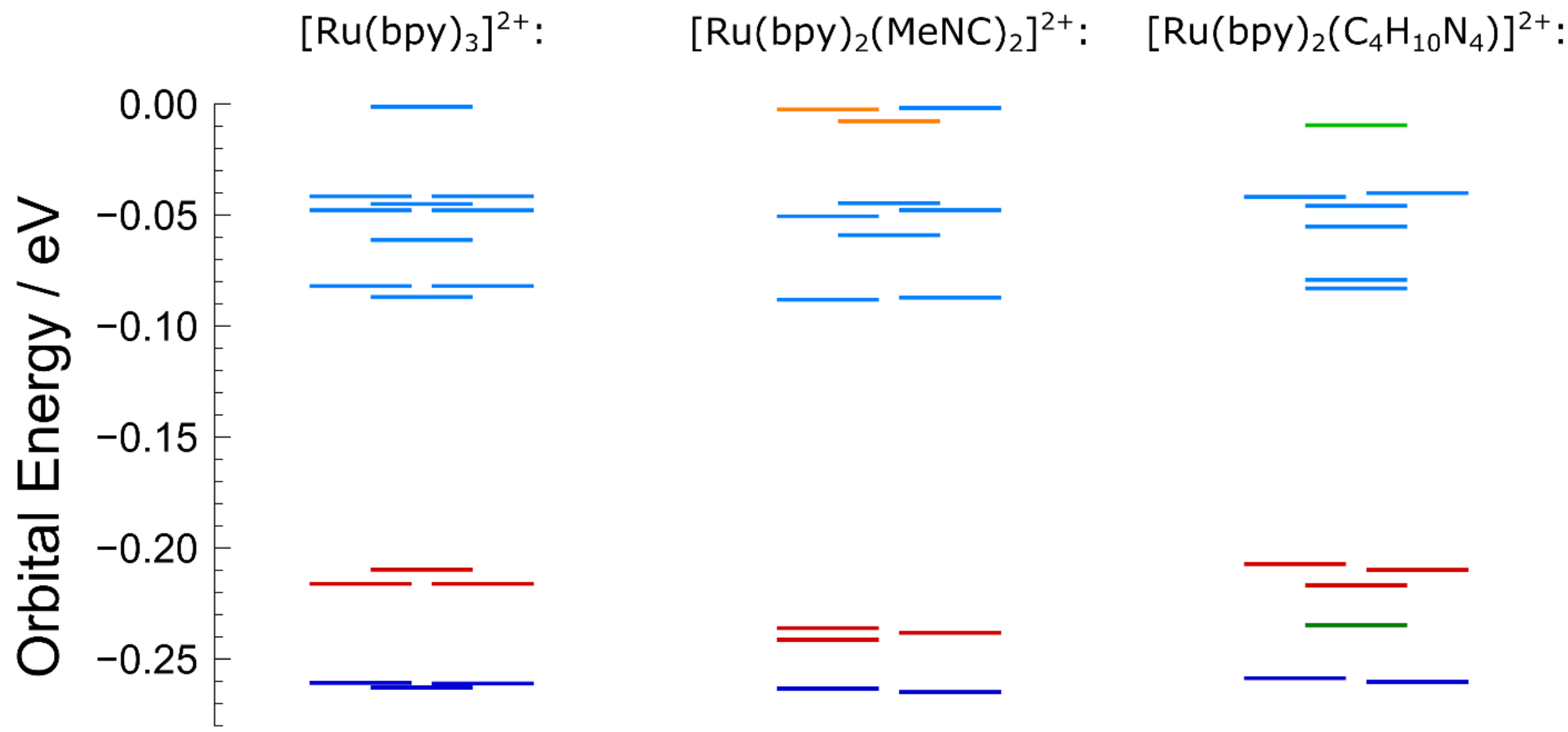

Figure S44. Orbital energies obtained from DFT calculations. From Löwdin population analysis, blue colored orbitals possess >90% bpy, red > 70% Ru, and green > 70% carbene character. Orange colored orbitals have mixed Ru-isocyanide character. Darker shades are occupied orbitals, lighter shades unoccupied.

Table S16. Highest occupied Ru- or carbene-based molecular orbitals obtained from DFT calculations for investigated complexes.

	$[Ru(bpy)_3]^{2+}$	$[Ru(bpy)_2(MeNC)_2]^{2+}$	$[Ru(bpy)_2(C_4H_{10}N_4)]^{2+}$
HOMO	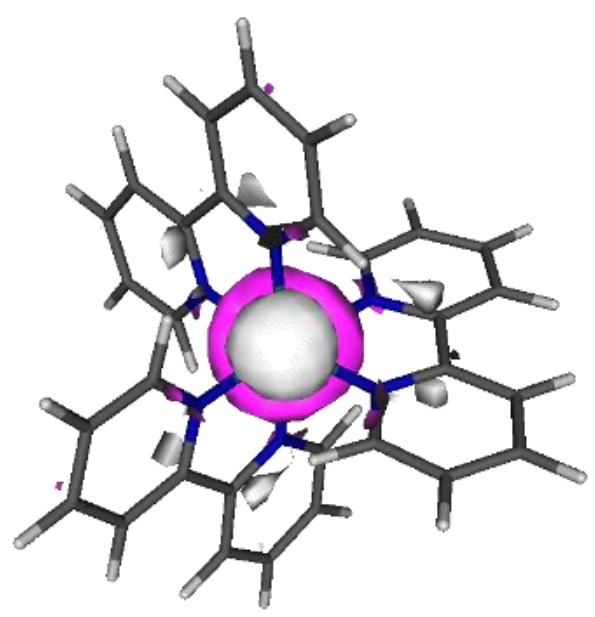	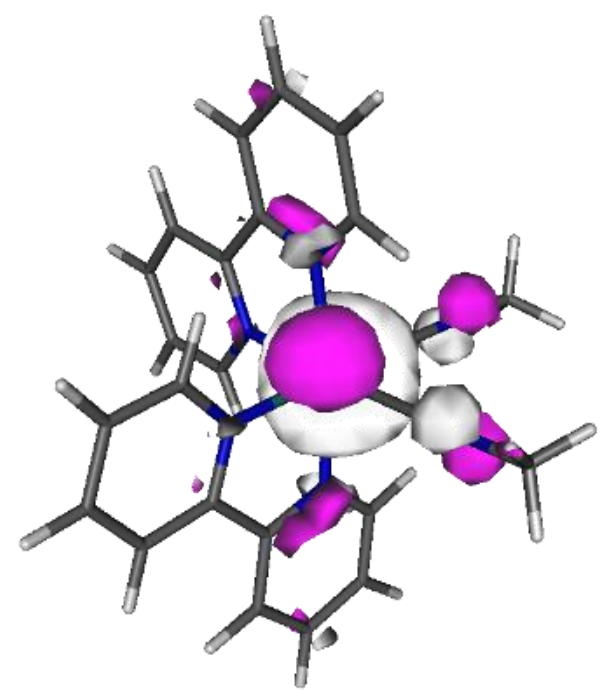	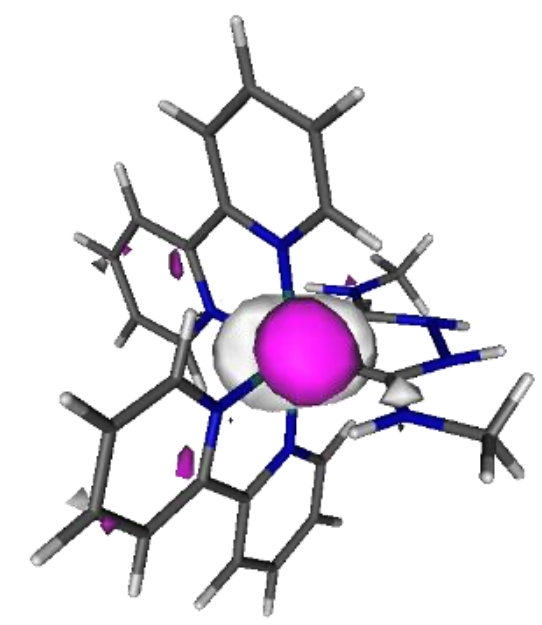
H-1	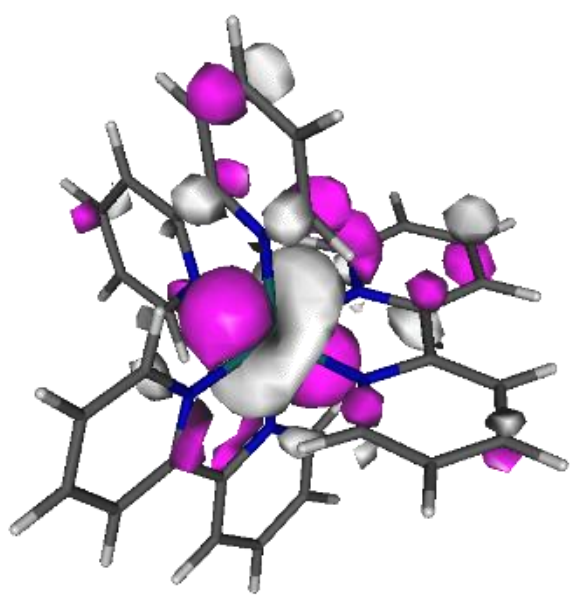	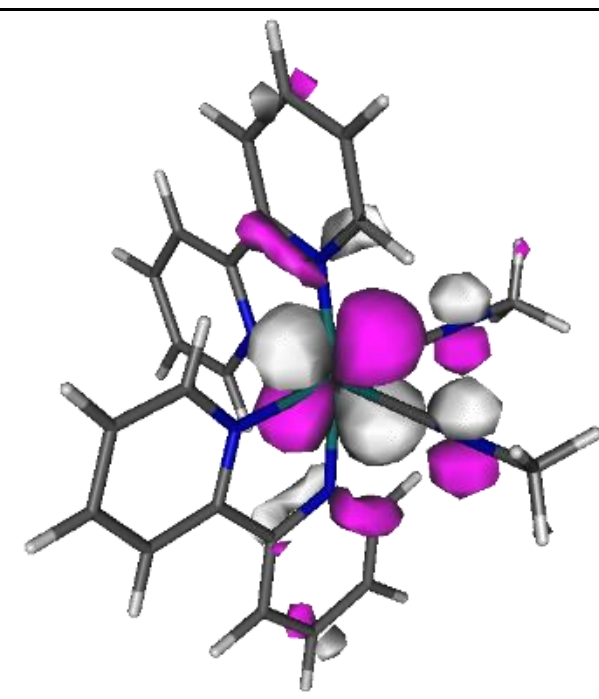	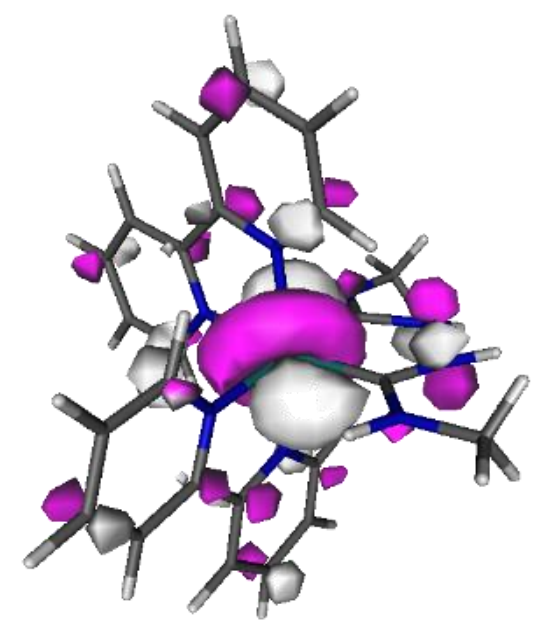
H-2	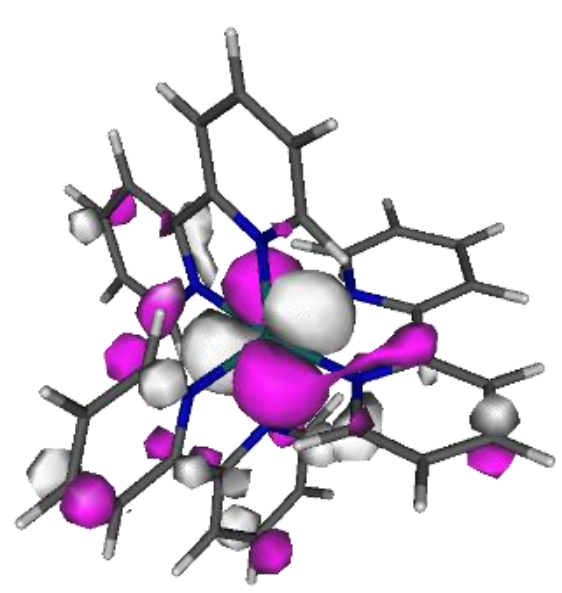	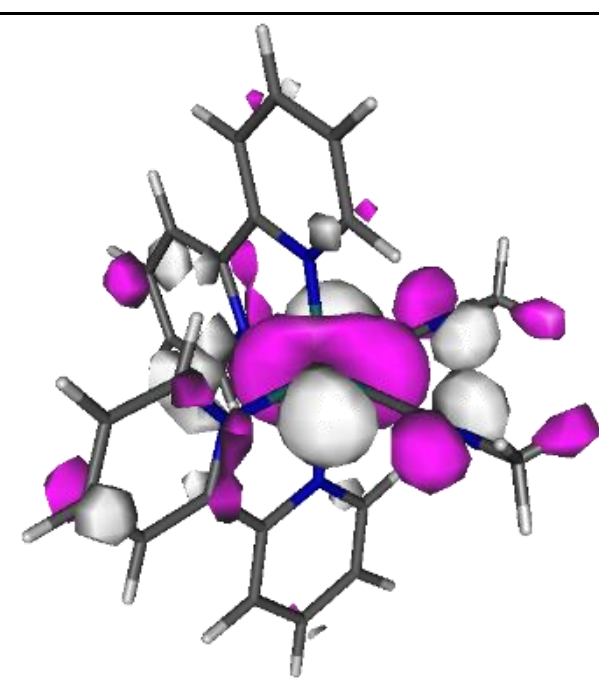	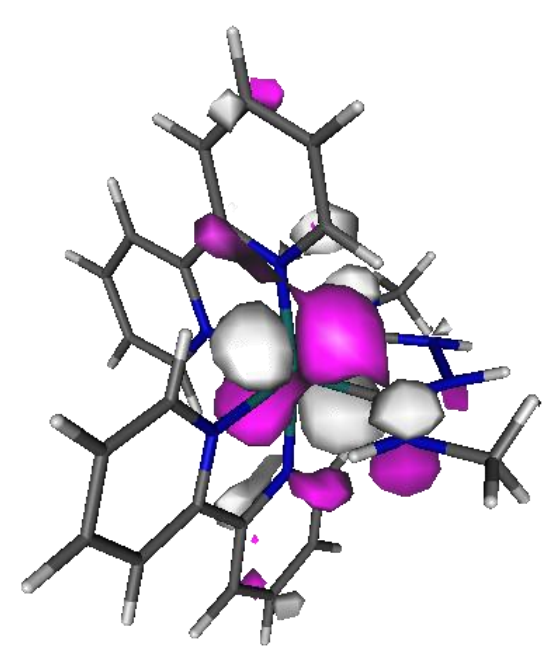
H-3	-	-	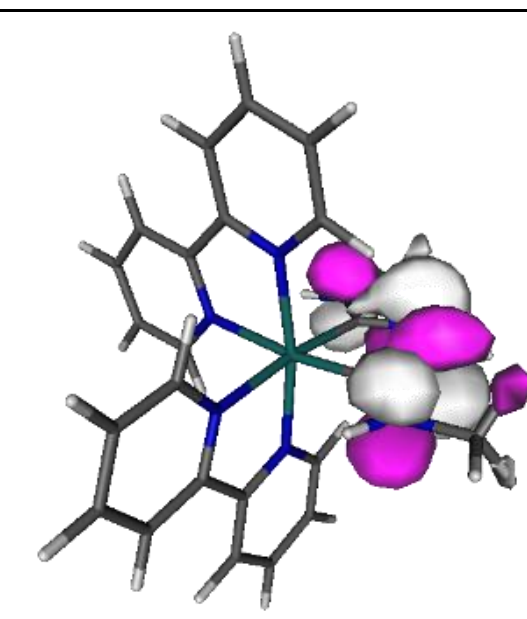

Ground State (S_0) TD-DFT Results

Table S17. TD-DFT calculated transitions for $[Ru(bpy)_3]^{2+}$. Only transitions with oscillator strengths over 0.01 are reported. Only orbital contributions greater than 10% are reported.

State	E / cm^{-1}	λ / nm	f_{osc}	Assignment (Contribution)
5	22010.7	454.3	0.023	H-1 → LUMO (78.9%)
6	22013.3	454.3	0.023	H-2 → LUMO (78.8%)
7	23214.3	430.8	0.163	H-2 → L+1 (18.5%)<br>H-2 → L+2 (22.2%)<br>H-1 → L+1 (22.2%)<br>H-1 → L+2 (18.5%)
8	23215	430.8	0.163	H-2 → L+1 (22.2%)<br>H-2 → L+2 (18.5%)<br>H-1 → L+1 (18.6%)<br>H-1 → L+2 (22.2%)
10	26400.7	378.8	0.016	HOMO → L+3 (98.2%)
11	28072.1	356.2	0.015	H-1 → L+3 (98.0%)
12	28073.6	356.2	0.015	H-2 → L+3 (98.0%)
16	30445.1	328.5	0.02	H-1 → L+6 (10.2%)<br>HOMO → L+7 (47.7%)<br>HOMO → L+8 (29.7%)
17	30446	328.5	0.02	H-2 → L+6 (10.2%)<br>HOMO → L+7 (29.7%)<br>HOMO → L+8 (47.7%)
21	31228	320.2	0.15	H-2 → L+5 (12.7%)<br>H-1 → L+4 (13.0%)<br>H-1 → L+6 (46.5%)<br>HOMO → L+8 (12.6%)
22	31229.1	320.2	0.15	H-2 → L+4 (12.7%)<br>H-2 → L+6 (46.8%)<br>H-1 → L+5 (12.8%)<br>HOMO → L+7 (12.6%)
34	35060.3	285.2	0.018	H-5 → L+1 (18.6%)
34	35060.3	285.2	0.018	H-5 → L+2 (26.5%)<br>H-4 → L+1 (24.7%)<br>H-3 → L+2 (24.8%)
35	35061.5	285.2	0.018	H-5 → L+1 (26.7%)<br>H-5 → L+2 (18.6%)<br>H-4 → L+2 (24.8%)<br>H-3 → L+1 (24.4%)
37	36425.7	274.5	0.389	H-5 → L+1 (11.0%)<br>H-5 --> L+2 (31.3%)

				H-4 → L+1 (12.3%)<br>H-3 → L+2 (12.2%)
38	36425.9	274.5	0.39	H-5 → L+1 (31.1%)<br>H-5 → L+2 (10.9%)<br>H-4 → L+2 (12.4%)<br>H-3 → L+1 (12.4%)
39	37131.5	269.3	1.449	H-5 → LUMO (20.3%)<br>H-4 → L+1 (25.2%)<br>H-3 → L+2 (25.3%)
43	39745.2	251.6	0.051	H-4 → L+3 (10.1%)<br>H-3 → L+3 (75.5%)
44	39745.7	251.6	0.051	H-4 → L+3 (75.5%)<br>H-3 → L+3 (10.1%)
45	40145.1	249.1	0.094	H-5 → L+3 (89.5%)

Table S18. TD-DFT calculated transitions for $[Ru(bpy)_2(MeNC)_2]^{2+}$. Only transitions with oscillator strengths over 0.01 are reported. Only orbital contributions greater than 10% are reported.

State	E / cm^{-1}	λ / nm	f_{osc}	Assignment (Contribution)
3	26,385.8	379	0.017	H-1 → L+1 (87.9%)
5	27,542.6	363.1	0.13	H-2 → LUMO (92.9%)
6	28,624.3	349.4	0.071	H-2 → L+1 (81.9%)<br>H-1 → LUMO (10.8%)
8	33,275.5	300.5	0.018	H-1 → L+2 (85.9%)
9	34,065.8	293.5	0.029	H-3 → LUMO (32.2%)<br>HOMO → L+3 (51.7%)
10	34,113.1	293.1	0.04	H-2 → L+2 (81.7%)
13	34,733.9	287.9	0.077	H-4 → LUMO (19.5%)<br>H-2 → L+2 (14.5%)<br>H-1 → L+3 (43.6%)<br>H-1 → L+4 (10.8%)
14	35,055.2	285.3	0.043	H-2 → L+4 (10.3%)<br>HOMO → L+4 (35.2%)<br>HOMO → L+6 (21.6%)
17	35,590.8	281	0.025	H-1 → L+4 (18.7%)<br>HOMO → L+3 (12.3%)<br>HOMO → L+5 (16.4%)<br>HOMO → L+6 (15.0%)
18	35,625.4	280.7	0.014	H-1 → L+4 (32.6%)<br>HOMO → L+5 (10.3%)<br>HOMO → L+6 (13.3%)
19	35,677.6	280.3	0.058	H-1 → L+3 (19.5%)

				H-1 → L+6 (13.1%)<br>HOMO → L+5 (37.8%)
21	36,348.1	275.1	0.38	H-2 → L+5 (23.9%)<br>H-1 → L+4 (11.5%)<br>HOMO → L+5 (32.9%)
23	37,556.8	266.3	0.345	H-4 → L+1 (13.2%)<br>H-2 → L+4 (53.0%)
24	38,184.4	261.9	0.683	H-4 → LUMO (12.5%)<br>H-3 → L+1 (10.1%)<br>H-2 → L+5 (62.5%)

Table S19. TD-DFT calculated transitions for $[Ru(bpy)_2(C_4H_{10}N_4)]^{2+}$. Only transitions with oscillator strengths over 0.01 are reported. Only orbital contributions greater than 10% are reported.

State	E / cm^{-1}	λ / nm	f_{osc}	Assignment (Contribution)
3	21,656.5	461.8	0.092	H-1 → LUMO (89.8%)
5	23,485	425.8	0.094	H-2 → L+1 (90.5%)
6	24,541.8	407.5	0.057	H-2 → LUMO (33.6%)<br>H-1 → L+1 (50.9%)
7	27,169.9	368.1	0.012	HOMO → L+2 (97.0%)
8	28,083.2	356.1	0.031	H-1 → L+2 (97.0%)
14	30,439.7	328.5	0.057	HOMO → L+5 (91.4%)
16	31,158.9	320.9	0.015	H-1 → L+5 (88.3%)
17	31,406.4	318.4	0.046	H-2 → L+3 (90.3%)
18	31,775.9	314.7	0.067	H-2 → L+5 (26.3%)<br>H-1 → L+3 (21.2%)<br>HOMO → L+4 (28.1%)
19	32,096.7	311.6	0.027	H-2 → L+4 (94.8%)
20	33,066.5	302.4	0.097	H-2 → L+5 (68.7%)<br>HOMO → L+4 (11.2%)
21	33,382.4	299.6	0.014	HOMO → L+6 (93.6%)
22	34,549.3	289.4	0.018	H-1 → L+6 (87.7%)
23	34,703.4	288.2	0.019	H-5 → L+1 (23.0%)<br>H-4 → LUMO (69.1%)
25	34,868.2	286.8	0.012	H-5 → LUMO (10.1%)<br>H-4 → L+1 (12.1%)<br>H-3 → L+2 (68.0%)
27	36,399.6	274.7	0.284	H-5 → L+1 (57.7%)<br>H-4 → LUMO (14.4%)
28	36,629.4	273	0.028	H-3 → L+3 (85.1%)

29	36,799.9	271.7	0.322	H-4 → L+1 (12.7%)<br>H-2 → L+9 (39.0%)<br>H-2 → L+14 (13.6%)
30	36,908.8	270.9	0.763	H-5 → LUMO (22.9%)<br>H-4 → L+1 (31.8%)<br>H-2 → L+9 (15.5%)

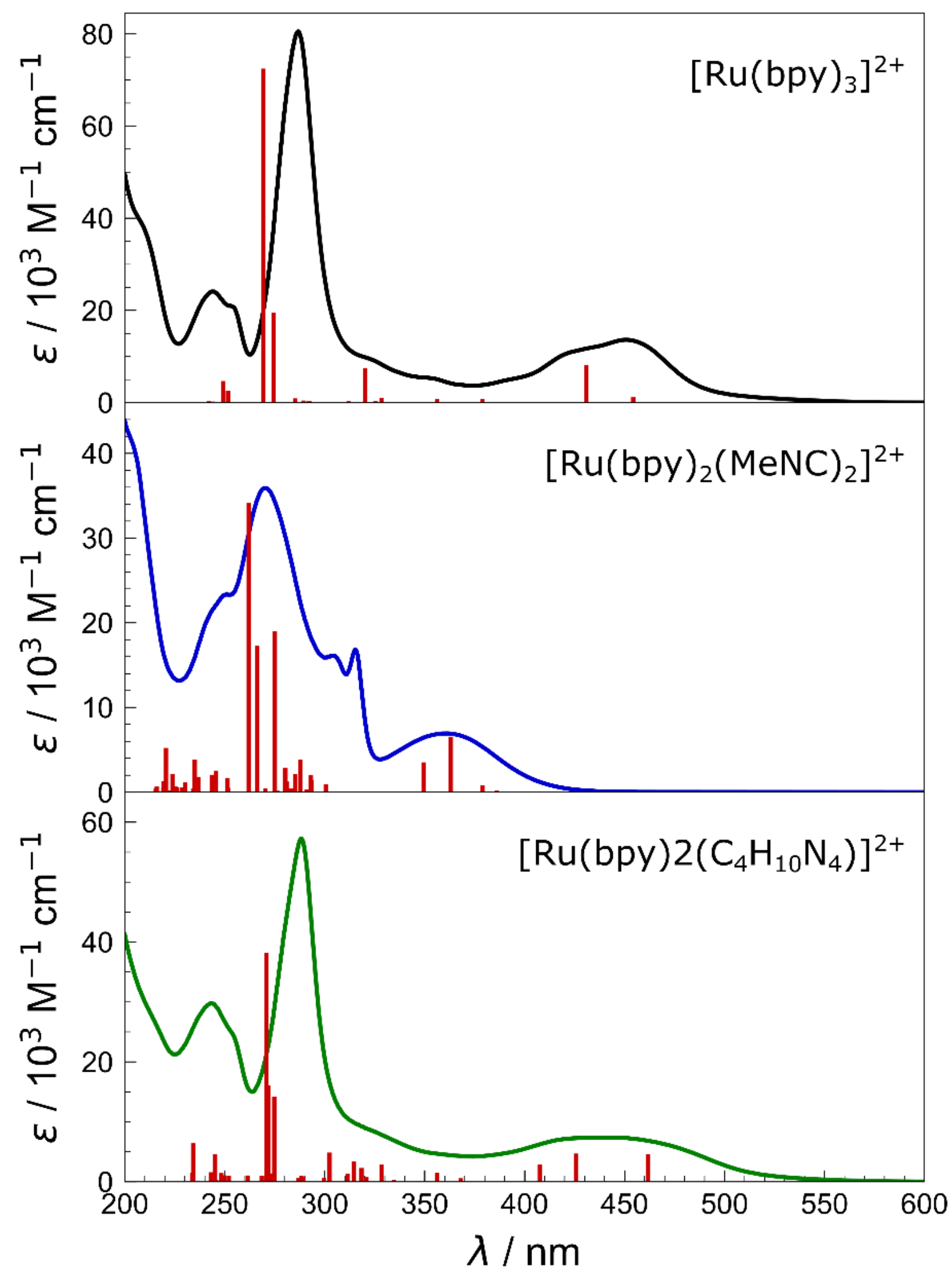

Figure S45. Electronic absorption spectra of investigated complexes in acetonitrile and corresponding TD-DFT predicted transitions.

Excited State (T_1) DFT Results

Table S20. Spin density of T_1 state obtained from DFT calculations for investigated complexes. ^{3}MLCT is the global minimum, ^{3}LF a higher energy local minimum.

	$[Ru(bpy)_3]^{2+}$	$[Ru(bpy)_2(MeNC)_2]^{2+}$	$[Ru(bpy)_2(C_4H_{10}N_4)]^{2+}$
^{3}LF	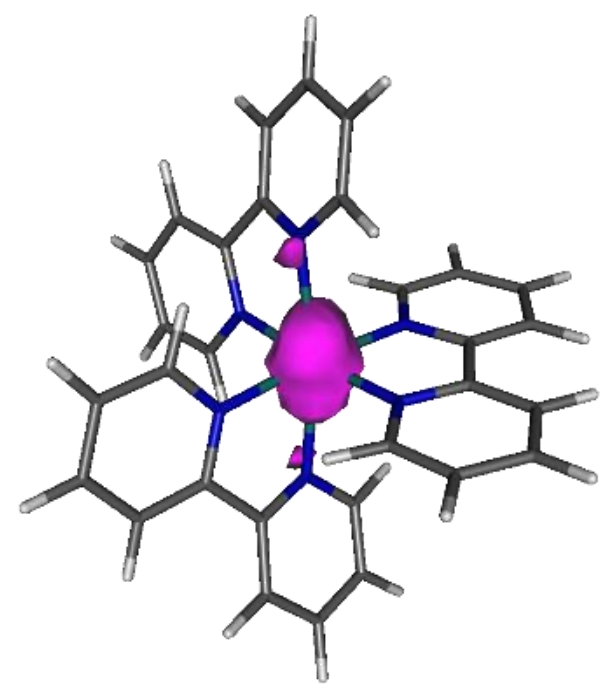	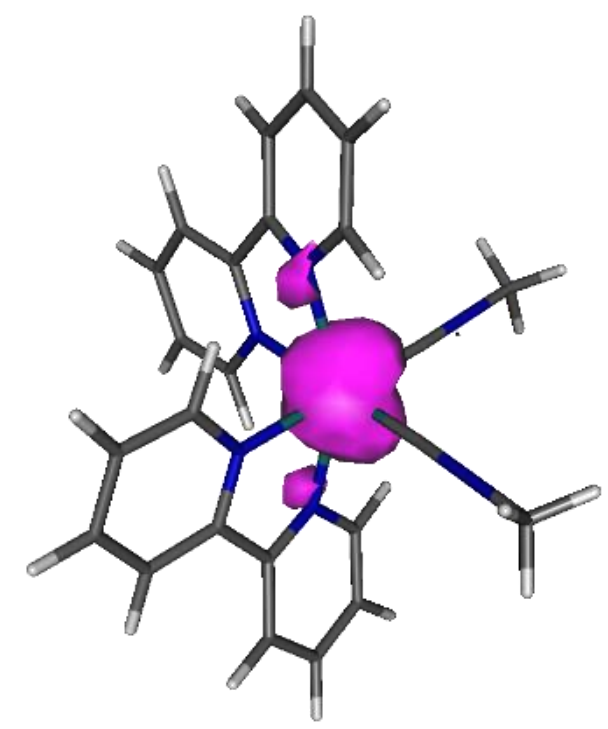	-
^{3}MLCT	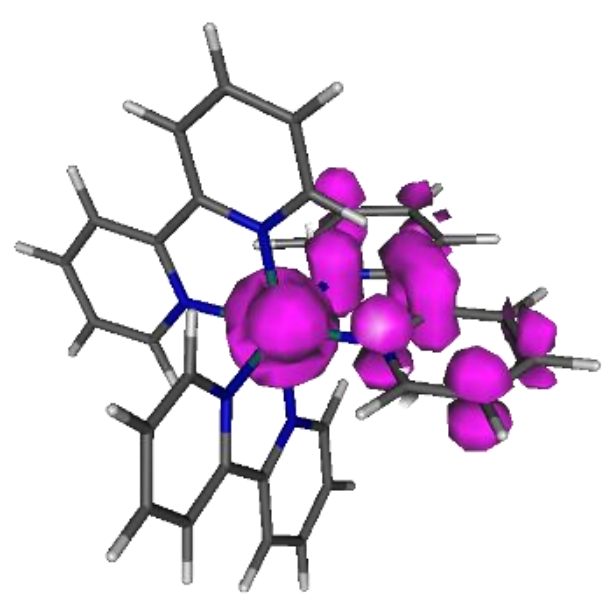	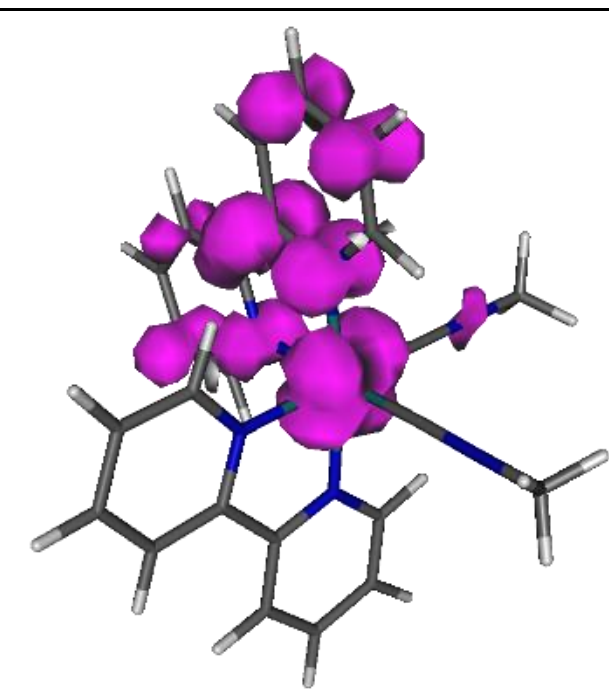	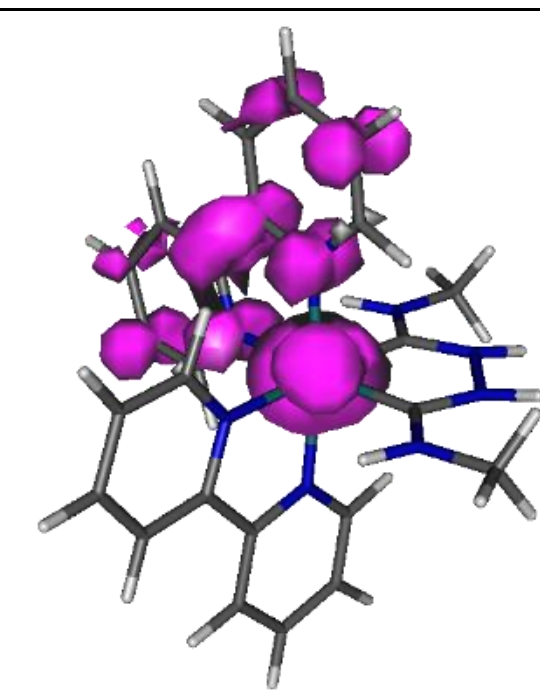

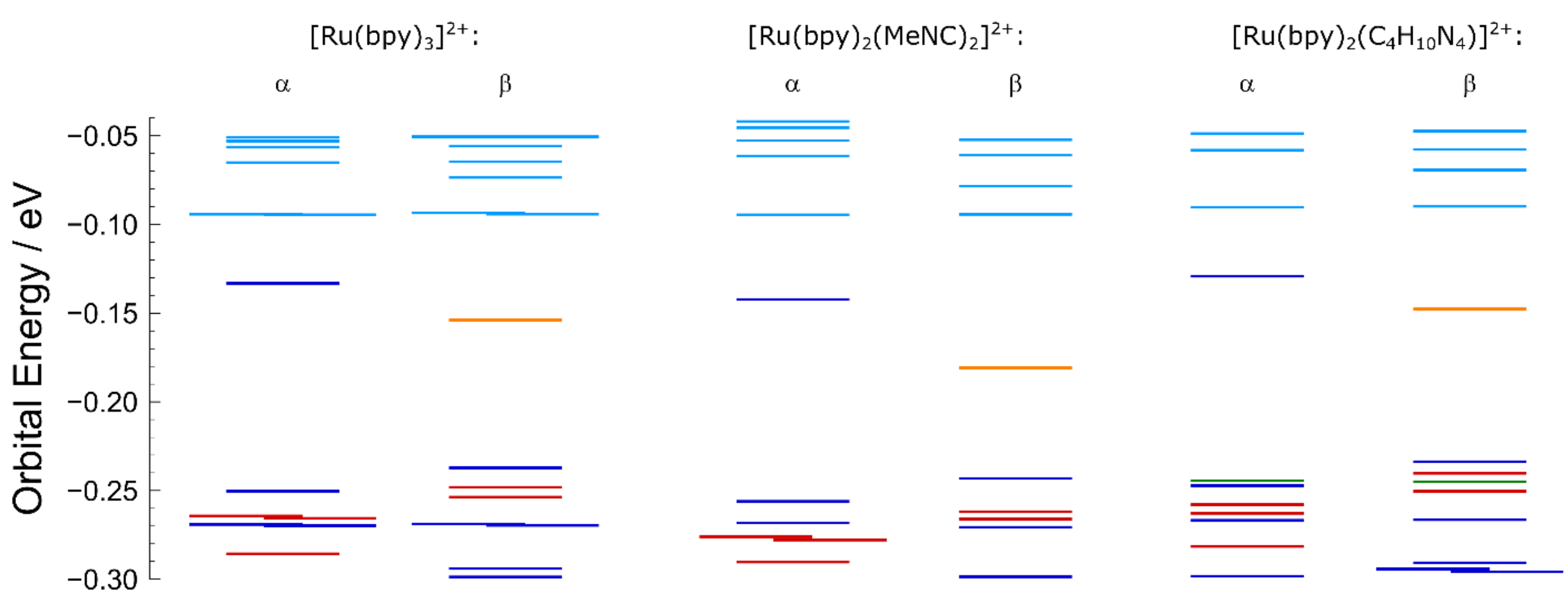

Figure S46. Orbital energies of investigated complexes in T_1 state obtained from DFT calculations. From Löwdin population analysis, dark blue colored orbitals are populated bpy orbitals, light blue unpopulated

bpy orbitals, dark red populated Ru orbitals, orange unpopulated Ru-based orbitals, and green populated carbene orbitals.

Table S21. Key orbitals in the T_1 (^3MLCT) state of $[Ru(bpy)_2(C_4H_{10}N_4)]^{2+}$ from DFT calculations. Orbital labelling is based on ground state orbital assignments.

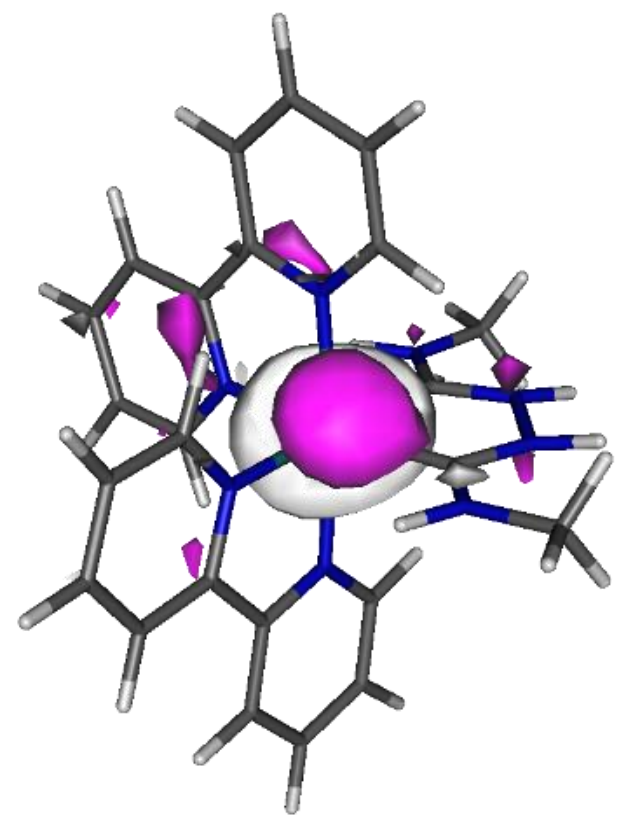 HOMOβ (unoccupied)	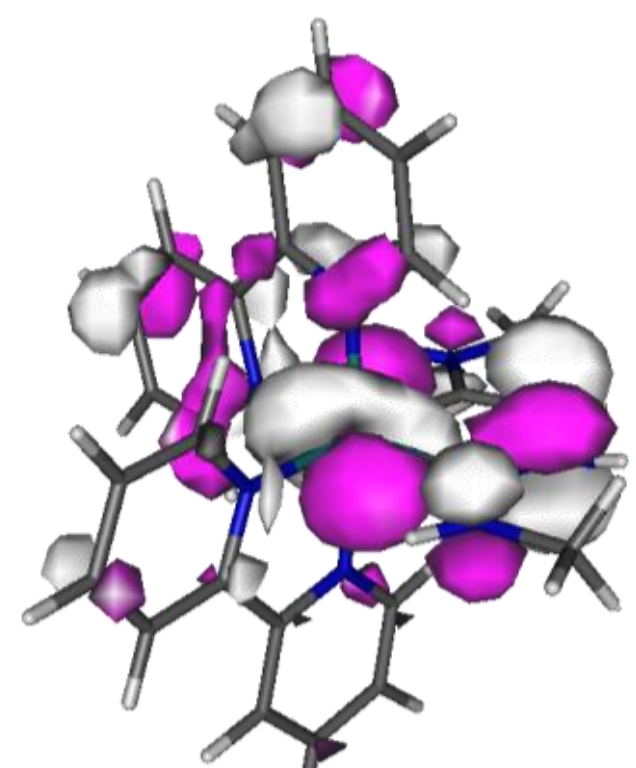 H-2β (occupied)	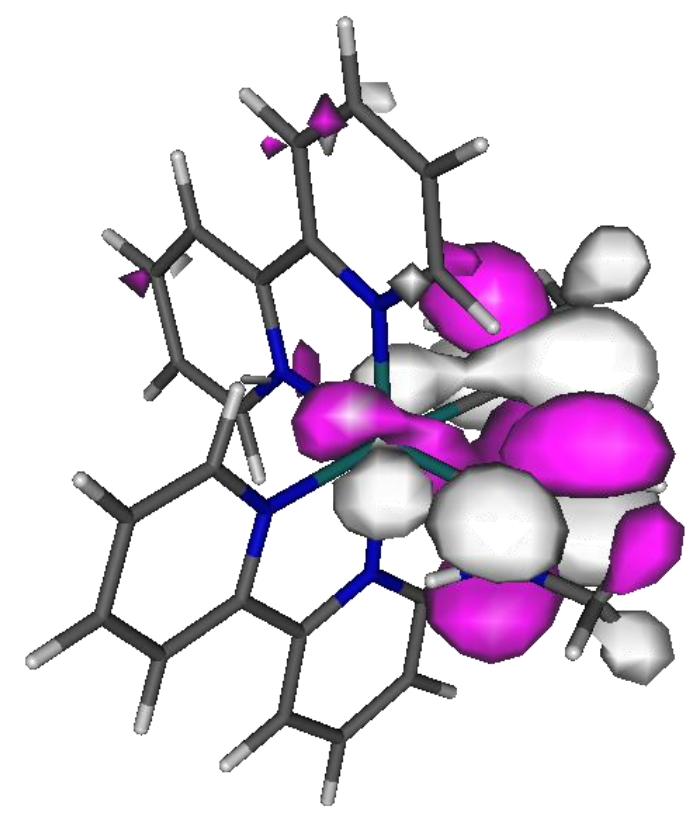 H-3β (occupied)
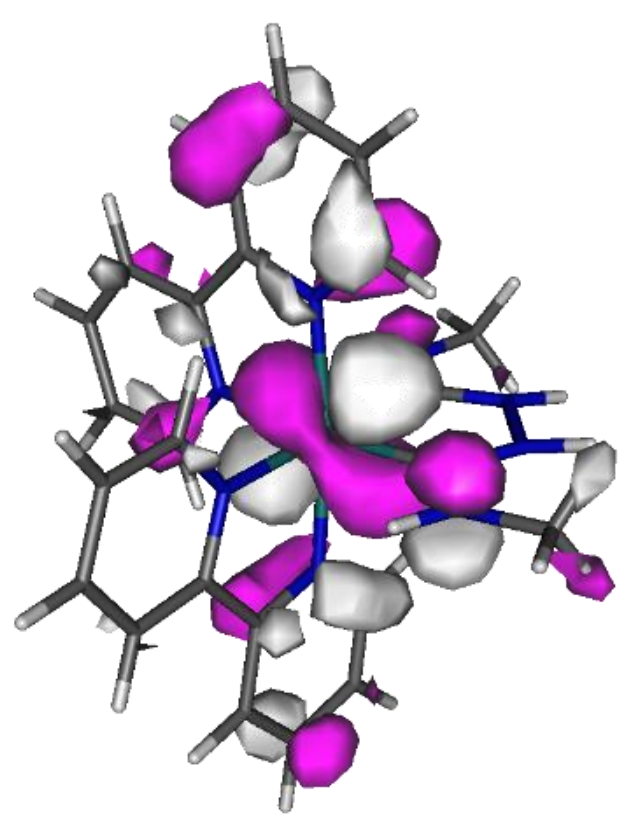 H-4β (occupied)	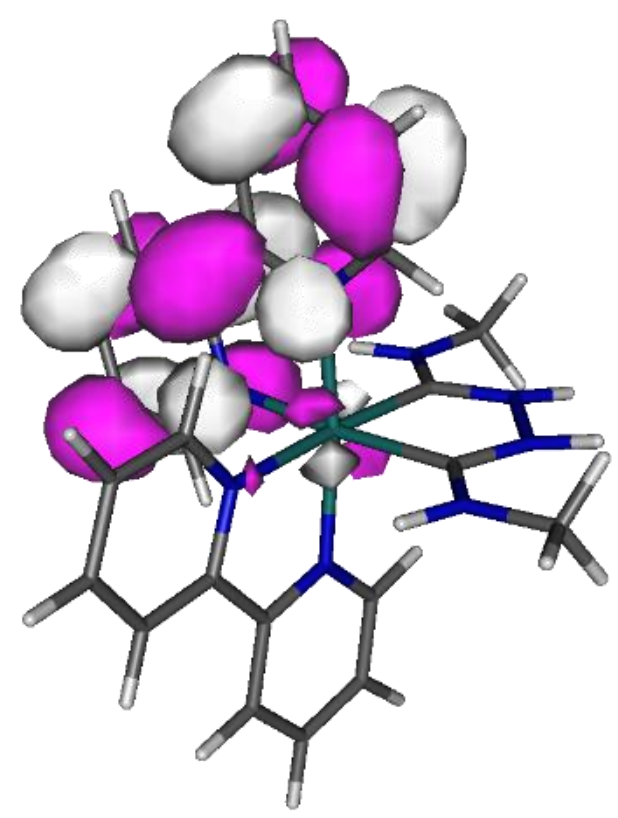 LUMOα (occupied)	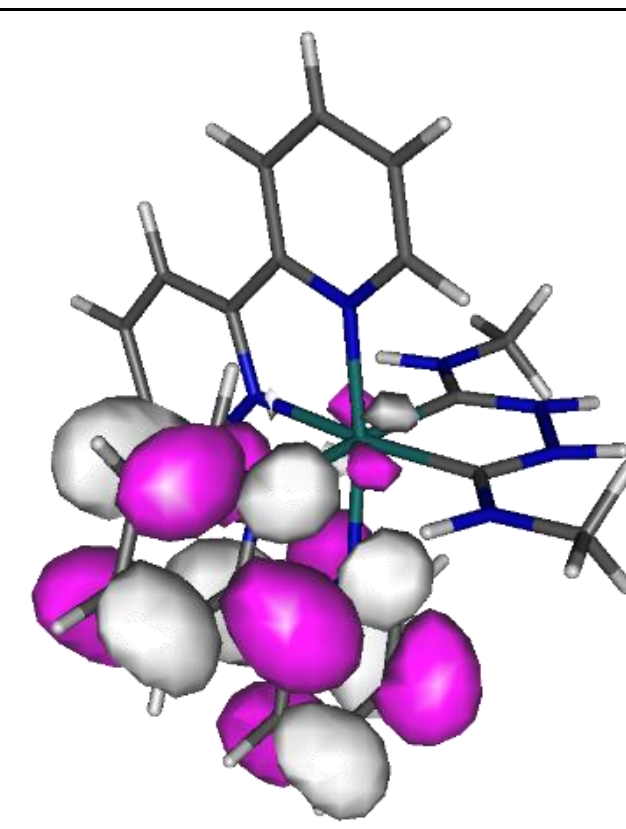 L+1α (unoccupied)
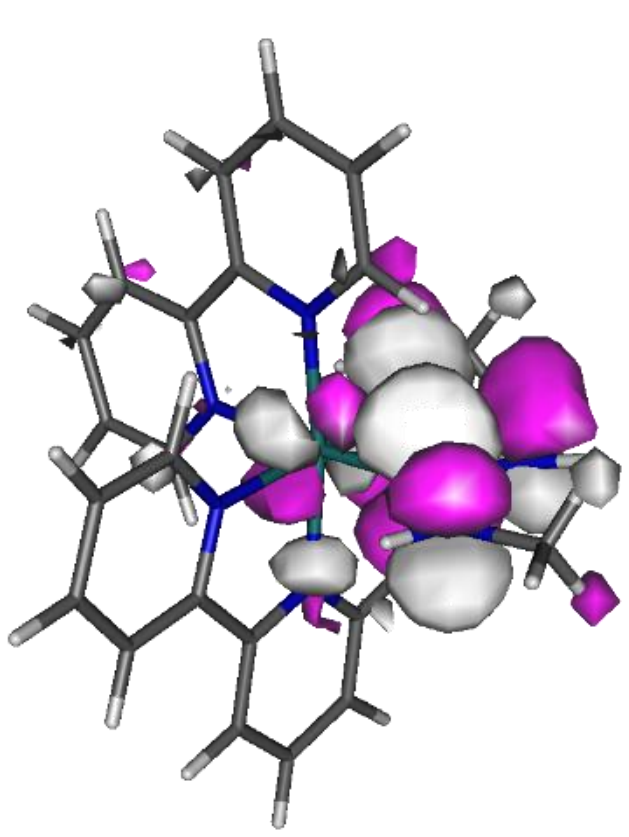 L+6α (unoccupied)	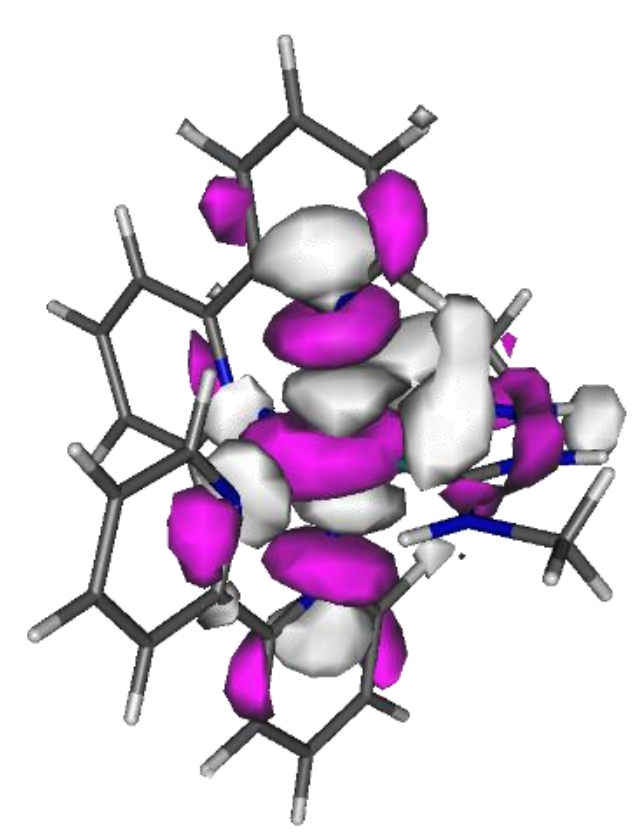 L+7α (unoccupied)	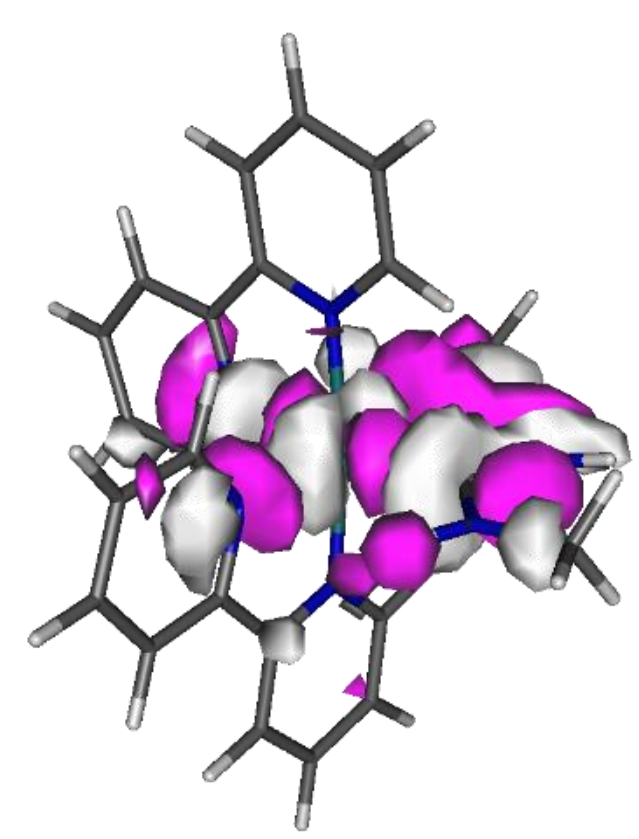 L+9α (unoccupied)

Excited State (T_1) TD-DFT Results

Table S22. TD-DFT calculated triplet excited states for $[Ru(bpy)_3]^{2+}$ from the T_1 state. Orbital labelling is based on ground state orbital assignments. Only orbital contributions greater than 10% are reported. Character is assigned based on Löwdin population analysis.

State	E / cm^{-1}	Assignment (Contribution)	Character
1	3,373.6	H-2β → HOMOβ (92.3%)	^{3}MLCT
2	3,721.1	H-3β → HOMOβ (83.6%)<br>H-1β → HOMOβ (10.7%)	^{3}MLCT
3	3,951.8	LUMOα → L+1α (32.9%)<br>LUMOα → L+2α (66.7%)	^{3}MLCT
4	3,955.7	LUMOα → L+1α (66.8%)<br>LUMOα → L+2α (32.9%)	^{3}MLCT
5	10,009.6	LUMOα → L+3α (85.4%)<br>LUMOα → L+7α (10.9%)	^{3}MLCT
6	12,058.4	LUMOα → L+7α (19.7%)<br>H-1β → HOMOβ (65.4%)	$^3\pi,\pi^*$
7	12,168.7	LUMOα → L+4α (93.7%)	^{3}MLCT
8	13,100.8	LUMOα → L+5α (65.0%)<br>LUMOα → L+8α (22.9%)	^{3}MLCT
9	13,122.6	LUMOα → L+6α (16.4%)<br>LUMOα → L+7α (47.8%)<br>H-1β → HOMOβ (17.2%)	^{3}MLCT
10	13,796.9	LUMOα → L+6α (79.6%)<br>LUMOα → L+7α (16.3%)	^{3}MLCT
11	14,129.5	LUMOα → L+5α (24.2%)<br>LUMOα → L+8α (69.6%)	^{3}MLCT
12	15,060.2	LUMOα → L+9α (97.6%)	^{3}LF
13	16,146.6	LUMOα → L+10α (97.9%)	^{3}LF

Table S23. TD-DFT calculated triplet excited states for $[Ru(bpy)_2(MeNC)_2]^{2+}$ from the T_1 state. Orbital labelling is based on ground state orbital assignments. Only orbital contributions greater than 10% are reported. Character is assigned based on Löwdin population analysis.

State	E / cm^{-1}	Assignment (Contribution)	Character
1	3,147.6	H-4β → HOMOβ (16.7%)<br>H-3β → HOMOβ (47.1%)<br>H-2β → HOMOβ (31.4%)	^{3}MLCT
2	3,986.2	H-3β → HOMOβ (35.1%)<br>H-2β → HOMOβ (53.7%)	^{3}MLCT
3	5,888.9	LUMOα → L+1α (99.5%)	^{3}MLCT
4	7,815.7	H-1β → HOMOβ (88.2%)	^{3}π,π*
5	12,366.6	LUMOα → L+2α (59.3%)<br>LUMOα → L+4α (27.9%)	^{3}MLCT
6	13,795.9	H-4β → HOMOβ (80.7%)<br>H-3β → HOMOβ (10.8%)	^{3}π,π*
7	14,040.3	LUMOα → L+2α (20.2%)<br>LUMOα → L+4α (28.6%)<br>LUMOα → L+5α (34.8%)	^{3}MLCT
8	14,449.0	LUMOα → L+2α (13.8%)<br>LUMOα → L+3α (12.6%)<br>LUMOα → L+4α (32.5%)<br>LUMOα → L+5α (36.3%)	^{3}MLCT
9	15,375.6	LUMOα → L+6α (89.8%)	^{3}LF
10	15,458.9	LUMOα → L+3α (73.6%)<br>LUMOα → L+5α (18.7%)	^{3}MLCT
11	19,007.5	LUMOα → L+7α (42.5%)<br>H-5β → HOMOβ (40.7%)	^{3}LF / ^{3}π,π*
12	19,146.8	LUMOα --. L+7α (53.8%)<br>H-5β → HOMOβ (26.3%)	^{3}LF

Table S24. TD-DFT calculated triplet excited states for $[Ru(bpy)_2(C_4H_{10}N_4)]^{2+}$ from the T_1 state. Orbital labelling is based on ground state orbital assignments. Only orbital contributions greater than 10% are reported. Character is assigned based on Löwdin population analysis.

State	E / cm^{-1}	Assignment (Contribution)	Character
1	2983.1	H-3β → HOMOβ (16.2%)<br>H-2β → HOMOβ (75.8%)	^{3}MLCT / ^{3}LLCT
2	3979.2	LUMOα → L+1α (99.7%)	^{3}MLCT
3	4526	H-4β → HOMOβ (78.4%)<br>H-1β → HOMOβ (13.6%)	^{3}MLCT
4	10603.4	LUMOα → L+2α (72.7%)<br>LUMOα → L+4α (22.5%)	^{3}MLCT
5	12256.7	LUMOα → L+2α (22.1%)<br>LUMOα → L+4α (55.3%)<br>H-1β → HOMOβ (17.6%)	^{3}MLCT
6	13036.8	LUMOα → L+3α (72.2%)<br>LUMOα → L+5α (15.3%)	^{3}MLCT
7	13286.6	LUMOα → L+4α (17.6%)<br>H-1β → HOMOβ (59.9%)	$^3\pi,\pi^*$
8	13796.6	LUMOα → L+3α (17.9%)<br>LUMOα → L+5α (75.3%)	^{3}MLCT
9	13922.8	H-3β → HOMOβ (80.3%)<br>H-2β → HOMOβ (17.6%)	^{3}LLCT
10	16819	LUMOα → L+6α (82.6%)<br>LUMOα → L+7α (14.8%)	^{3}MLCT<br>(Ru→carbene)
11	17347.2	LUMOα → L+6α (15.2%)<br>LUMOα → L+7α (82.2%)	^{3}LF
12	19922.5	H-5β → HOMOβ (98.7%)	$^3\pi,\pi^*$
13	21491.6	LUMOα → L+9α (91.5%)	^{3}LF

XYZ Coordinates of Geometry-Optimized Structures

$[Ru(bpy)_3]^{2+}$ S_0 State:

Ru	0.05957042774594	0.42033815319611	0.77993152534705
H	0.56040742975219	-0.22283014887602	-5.08235863809011
C	0.46364757958600	-0.07225701981048	-4.01567635259398
N	0.21409767909575	0.30443889335493	-1.28012639199162
C	0.42412214346767	-1.15905498576689	-3.15910626986625
C	0.37673977564268	1.20655985601111	-3.48314464351561
C	0.25483673664088	1.35248301568460	-2.11374545710134
C	0.29817820510488	-0.94853426659742	-1.79056143482893
H	0.48960939157838	-2.16239916542196	-3.55154804832900
H	0.40286266059787	2.08502912832438	-4.11239443014459
H	0.18242195719921	2.32950792778483	-1.66079569974996
C	0.23862948970266	-2.03029779338323	-0.80171092712150
C	0.11970364280956	-3.90491896473369	1.19509586884474
C	0.28651459118689	-3.38063253899612	-1.12899339245778
N	0.13587458697240	-1.62622088692651	0.48807767778297
C	0.07559605880249	-2.54829045960492	1.45805834669113
C	0.22714583442477	-4.32891963748500	-0.12215114142977
H	0.36789797614288	-3.68817547800909	-2.16044974192436
H	-0.00518688777157	-2.17731721235013	2.46846814746516
H	0.26379892442048	-5.38254295790297	-0.36367396401021
H	0.06919717339728	-4.60848162899687	2.01413785922559
H	-2.36424624639627	0.29357320662417	-1.15023717153602
C	-2.82617033695318	0.22878032128790	-0.17689011612552
C	-3.89462798733439	0.07941001931884	2.32623268579398
C	-4.19730913985953	0.11339981975373	-0.04247619454454
N	-2.00180278737221	0.27260518329340	0.87833770338713
C	-2.52372284687747	0.19810188830942	2.12719228392645
C	-4.74160448344375	0.03678176588306	1.23204545392692
H	-4.81882359952084	0.08446867588315	-0.92634585596727
H	-5.80999963233702	-0.05445803477590	1.37364555509773
H	-4.29644074601999	0.02195315985147	3.32636579307600
N	-0.25064107911530	0.35688642954047	2.82418993198455
C	-0.88481924030363	0.28010012237849	5.52175334107100
C	-1.54426893694261	0.25992812369978	3.21734609525905
C	0.71138730705915	0.41878065675492	3.75444997941997
C	0.43638903543020	0.38203243985393	5.10891137629499
C	-1.88345899890772	0.21898322717802	4.56493597214850
H	1.72527197319093	0.49457474105445	3.39201454516079
H	1.24934832362249	0.43362893593097	5.81941572251846
H	-2.91767284517693	0.14124622991426	4.86397668523191

H	-1.13571554991882	0.24887529947413	6.57335838153845
H	5.85453468198395	1.60283551304498	0.98950839620188
C	4.80258579198608	1.35422734650054	0.95212748085257
N	2.10413475559303	0.72587292522214	0.85895095084928
C	3.84773421367973	2.35547487655167	0.99985880558024
C	4.38734355344310	0.03338691560618	0.85528287335383
C	3.03254259151249	-0.23867218546097	0.80989484331291
C	2.49966973186882	2.01912190253525	0.95112743660931
H	4.14856257899101	3.38955060723598	1.07278330068468
H	5.09626731583134	-0.78154014338359	0.81400025127668
H	2.66829340854359	-1.25214308677343	0.73721917805735
C	1.41145045456771	3.00149225742991	0.99872547202414
C	-0.75455766240322	4.68093164180851	1.05645971396050
C	1.61291976787320	4.37244577749774	1.11255547611238
N	0.16198081350309	2.48230064880399	0.91620040814978
C	-0.89145558555438	3.30956863907183	0.94683042654597
C	0.52061792996472	5.22240244124215	1.14153421001624
H	2.61354208862950	4.77189374145238	1.17726802725821
H	-1.86543359819975	2.84982139305301	0.87634742846159
H	0.66416898739234	6.29085811162847	1.22892553518532
H	-1.63707937852874	5.30476163622993	1.07475972564291

$[Ru(bpy)_3]^{2+}$ T_1 State:

Ru	0.06058318845963	0.41656012217448	0.77912594483915
H	0.68361179744632	-0.17158202726241	-5.06618317268856
C	0.57042281189243	-0.03509030151886	-3.99927376005844
N	0.27664969018036	0.30446455936968	-1.27772614590037
C	0.47889096546591	-1.13693093547383	-3.16509544208556
C	0.51640912061239	1.23840388947675	-3.45109786515252
C	0.37262377816579	1.36933512211053	-2.08278901617169
C	0.33054856651313	-0.94518623023119	-1.79813575736974
H	0.51955026981483	-2.13484571815261	-3.57390965638311
H	0.58540490306188	2.12388934384384	-4.06647101227539
H	0.32542608183947	2.33771990952733	-1.60916398001822
C	0.22580248010050	-2.03568679090627	-0.82428489438281
C	0.01124141725020	-3.92060184899655	1.15760697232921
C	0.25504422465576	-3.38240617706406	-1.16066878231910
N	0.09450747886891	-1.64652561901628	0.46568753254911
C	-0.01588391900576	-2.56571698700065	1.43105444140155
C	0.14834454529430	-4.33375496369363	-0.15980113570657
H	0.36069943311280	-3.68536780497420	-2.19102032982743
H	-0.11919206403131	-2.19878697713656	2.44111250282995
H	0.17172517845892	-5.38612938722332	-0.40801842977721
H	-0.07556991404737	-4.62988635242423	1.96818921985403
H	-2.36567105496956	0.13693233784946	-1.13725795593679
C	-2.82969831829476	0.11269080351996	-0.16267623016935
C	-3.89520433281425	0.06757921538280	2.35162047442833
C	-4.19935712849360	-0.00915888810727	-0.02034628150502
N	-2.01507350678184	0.21643900643594	0.89315477781021
C	-2.52758440241966	0.19220923565887	2.14584016051722
C	-4.73896076141135	-0.03174451237194	1.25778778337742
H	-4.82228668473062	-0.08413764636145	-0.90008288176409
H	-5.80642732570575	-0.12609171886839	1.40389099740825
H	-4.29662834332452	0.05178202806110	3.35324522424435
N	-0.25308338377435	0.40887187490315	2.82646735846697
C	-0.85695813857158	0.40206195650968	5.51768969073697
C	-1.54291098415502	0.30161605135585	3.22636984795870
C	0.72305643442295	0.51545081756029	3.73586528396573
C	0.45924264157980	0.51116951055814	5.09256188771504
C	-1.86729070472307	0.29756511184289	4.57610640780320
H	1.72925779828559	0.59823580784242	3.35481001559647
H	1.27661351182631	0.59522780895502	5.79430192394082
H	-2.89668854163893	0.21242798143636	4.88865815695714
H	-1.09709452776969	0.39840845859241	6.57219351118074
H	5.84027143117637	1.59193962471969	0.92540951542556

C	4.78584138730816	1.34989112782475	0.90026268020159
N	2.08651699764042	0.69914469731421	0.84735023771142
C	3.85232972161024	2.34395351976025	0.98283774640981
C	4.36687925889342	0.00107488059108	0.77468954872031
C	3.02265292406334	-0.26435725368285	0.74558946790800
C	2.47080455863053	2.03327545356469	0.95062129951919
H	4.16002635455393	3.37614012735480	1.06877409943547
H	5.07751098880686	-0.80940392076788	0.70437504751394
H	2.65618126962428	-1.27679721550177	0.65512772792488
C	1.42263400102428	2.97549813571593	1.00353185282064
C	-0.80363543357510	4.64297759122706	1.13339483339630
C	1.58901463443936	4.37592673433139	1.13465505063171
N	0.13252443921112	2.45790375247707	0.92720625023950
C	-0.92872730670173	3.28383164940154	1.00898472004272
C	0.49960879152534	5.19802820711685	1.19560833763310
H	2.58704705629048	4.78621211076126	1.18884476410238
H	-1.90141814434149	2.81661248999863	0.95433336017934
H	0.63138971597299	6.26753478296054	1.29499895473080
H	-1.68698492679802	5.26293043864955	1.17998211903469

$[Ru(bpy)_3]^{2+}$ ^{3}LF State:

Ru	0.06222740176736	0.40483365611346	0.80175605046187
H	1.54947851206530	-0.26329944262685	-5.07818050330553
C	1.13567334675026	-0.10667697995361	-4.09086730915686
N	0.10009186187480	0.28832206666512	-1.55717366064446
C	0.93321556559052	-1.18294764632069	-3.24302302790028
C	0.81366325347551	1.17320730468038	-3.65243939903103
C	0.30519047363857	1.32420568306490	-2.37432340894588
C	0.40624164860566	-0.95332590474410	-1.97385166139472
H	1.19752172017944	-2.18179852012132	-3.55723551332644
H	0.95441980725963	2.03814341333820	-4.28593778703203
H	0.04780738969059	2.30033080118110	-1.98202974636908
C	0.17829799465966	-2.03121069527890	-0.99530363937916
C	-0.23423651287083	-3.93926030900095	0.93722582011551
C	0.09123966663416	-3.37128323811302	-1.36056608833452
N	0.05334772427116	-1.65732410578166	0.29903043973920
C	-0.15173721493166	-2.59286436368855	1.23654572292658
C	-0.11406725476433	-4.33455523925156	-0.38853364109137
H	0.16850129755465	-3.65400589081934	-2.39962933500177
H	-0.24568063886575	-2.23467080420518	2.25175640356465
H	-0.18649325363750	-5.37832633079710	-0.66330655985548
H	-0.39382027317607	-4.65538089507881	1.73087108537109
H	-2.37725377552801	0.01998794589915	-0.94572040004613
C	-2.84414462908483	-0.02108316866679	0.02785991760050
C	-3.90017538862311	-0.11303108595593	2.53523635079510
C	-4.19989319341137	-0.24790926516768	0.16864229247261
N	-2.03239475753502	0.16575637944251	1.07716744006399
C	-2.54497222280758	0.12671703286459	2.32829114703066
C	-4.73634690477638	-0.29933266193679	1.44832392327577
H	-4.81396984159213	-0.38500078333330	-0.71016058017755
H	-5.79076710525585	-0.48836809352447	1.59898820038716
H	-4.29319734146841	-0.16760565866345	3.53923302319878
N	-0.29512659896948	0.23404198406418	3.11870165382236
C	-1.03883484645713	0.87635751557847	5.70162092926766
C	-1.59679365330806	0.34640874024401	3.43478887253520
C	0.63082745449189	0.43886526888880	4.05754845399173
C	0.30471804173006	0.75322167487368	5.36540981559235
C	-2.00186512259718	0.67511648720511	4.72675868804329
H	1.66206732209036	0.34316155210955	3.74081528484695
H	1.08544592735642	0.89986774020228	6.09897571050998
H	-3.04945027491424	0.78706779608610	4.96395058143983
H	-1.33292176102255	1.13557803197167	6.70998660104737
H	5.84789934464676	1.63482033511318	0.57357078880947

C	4.79630573718668	1.38312991043536	0.59822517944127
N	2.10240403017595	0.74588998823782	0.66641928511860
C	3.84974625722456	2.37063026685541	0.80912122974163
C	4.37669891494437	0.07119776525656	0.42157898797261
C	3.02382937147738	-0.20621500647809	0.46045881348642
C	2.50225425886000	2.03099087902329	0.84041571440911
H	4.15636640110335	3.39554863979440	0.95250722637243
H	5.08139077428605	-0.73103499608309	0.25451143667934
H	2.65446444827327	-1.21168363277986	0.32657764674838
C	1.42195056085266	2.99129107374354	1.05975641454062
C	-0.74566760106582	4.62339186714632	1.46431654371710
C	1.61621089878662	4.36033762818734	1.20407548722951
N	0.17764562719866	2.45281927620746	1.11494082048758
C	-0.87674319873708	3.25616617295425	1.31528638750427
C	0.52332740051549	5.18480340666342	1.40754398594573
H	2.61039470548479	4.77744579154477	1.15135806018540
H	-1.84419508209155	2.77852130123162	1.35353072232388
H	0.66073092441969	6.25185558503376	1.51890533857130
H	-1.62628661763029	5.22989075646931	1.62161078360738

$[Ru(bpy)_2(MeNC)_2]^{2+}$ S_0 State:

Ru	-0.21063833805817	0.42124992371030	0.62263484158813
H	-0.33848992118347	-0.10295597087839	-5.28787601270035
C	-0.30654669422643	0.02576016925324	-4.21446279691783
N	-0.22809443851038	0.34256923440527	-1.46270493288021
C	-0.23299747537519	-1.07842843553271	-3.38277106972156
C	-0.33669561513779	1.29165465532439	-3.64908159878018
C	-0.29331524109490	1.40767891375475	-2.27198303518762
C	-0.19412783379401	-0.89913764366974	-2.00465924335346
H	-0.20408224502086	-2.07142320024914	-3.80403515659772
H	-0.39066350389892	2.18373453985583	-4.25679945247638
H	-0.31303963689069	2.37479954580052	-1.79405948912794
C	-0.11445448882756	-2.01350475215419	-1.04893779219706
C	0.05526910391677	-3.95604949167341	0.88129692201382
C	-0.07938568303773	-3.35073497480689	-1.42753511083883
N	-0.07259293073195	-1.66188950086650	0.25505284097878
C	0.01388882480271	-2.60873782219725	1.19335472356101
C	0.00503068023582	-4.33119406352642	-0.45292980119692
H	-0.11834016245559	-3.62721970091257	-2.46977239111195
H	0.05121712064214	-2.27106191492056	2.21825272476044
H	0.03213119905156	-5.37503431376239	-0.73514697254456
H	0.12499441065788	-4.68723799062614	1.67410265095327
H	-2.79752166303715	0.28306239268637	-1.15935978854540
C	-3.18690951824543	0.11733071360759	-0.16600383062757
C	-4.06697699221371	-0.28726980044174	2.38135406502239
C	-4.53552541838865	-0.10901861728877	0.04413077281601
N	-2.30308798243221	0.14360633642509	0.83538538978746
C	-2.72493109213662	-0.05137712214536	2.10428760014148
C	-4.98089959962432	-0.31647912522973	1.34111435918890
H	-5.21351973365863	-0.12075658931003	-0.79741995761874
H	-6.02771002985031	-0.49832355714902	1.54393098747658
H	-4.39785197564983	-0.44601444789030	3.39606744937388
N	-0.42014763004821	0.23016193718053	2.69005248167429
C	-0.89598331699900	-0.08775938335419	5.40133055603479
C	-1.67995532195677	0.00693899461882	3.13675586900457
C	0.58433285234241	0.29799083861488	3.57316939286448
C	0.38893095045270	0.14164223222753	4.93297856642017
C	-1.93859439293008	-0.15397172514781	4.49315589439262
H	1.56806673305373	0.48051237688046	3.16920996219389
H	1.23493170942959	0.20307648481687	5.60273706314877
H	-2.94601018875208	-0.32809124630172	4.83828420102439
H	-1.08681925634174	-0.21271412035150	6.45858260615851
C	1.73116720399437	0.59593820649339	0.58797215730053

C	-0.40540826153468	2.35356835511762	0.79834535203662
N	2.88686076019433	0.68340513105263	0.56126314561439
N	-0.53670800606466	3.50008311013465	0.90904496672527
H	4.73052884163163	-0.01724809873072	1.14697306525188
C	4.30462373529151	0.79473644524374	0.55873828789307
H	4.59065724842101	1.75164584377250	0.99377396171511
H	4.66896166369048	0.73474216913991	-0.46601144786578
H	-1.50257811077363	5.23824227791286	0.37973875960989
C	-0.68327299622508	4.91036366384970	1.01848048527715
H	0.24161188169115	5.39330361054245	0.70516423082169
H	-0.89950422439329	5.17101250669532	2.05379154746572

[Ru(bpy)$_2$(MeNC)$_2$]$^{2+}$ T$_1$ State:

Ru	-0.22690212294089	0.40050282115458	0.65477643113707
H	-0.22302646165118	-0.10544483650333	-5.27554924413211
C	-0.21429256828732	0.01743956969945	-4.20108626726695
N	-0.19767900004673	0.31914280714232	-1.45627401316545
C	-0.17798751810772	-1.09366081320550	-3.37538020315134
C	-0.23525636010233	1.28200027557532	-3.63248653631371
C	-0.22147559647434	1.39319350344779	-2.25446177426886
C	-0.16991017017477	-0.92011639482914	-1.99756681769587
H	-0.15484553359483	-2.08556748908724	-3.79951911561545
H	-0.25923735123321	2.17679292640578	-4.23774696413510
H	-0.23307049599814	2.35885641816438	-1.77258568113171
C	-0.12734225867643	-2.02995579601787	-1.03798511156369
C	-0.04770890019947	-3.95931830869077	0.91157338874473
C	-0.10539906569757	-3.36883626739379	-1.40518780989248
N	-0.11268640130638	-1.67272682357735	0.26541974399972
C	-0.07267721254737	-2.61072113615785	1.21677129472276
C	-0.06593446391310	-4.34256725057492	-0.42079944439996
H	-0.12016230063960	-3.64988626197488	-2.44689585017294
H	-0.05655103094593	-2.26293499364077	2.23820290607346
H	-0.04938847622355	-5.38874344725597	-0.69454212652228
H	-0.01433107604848	-4.68556850275370	1.71103504872174
H	-2.78113127991535	0.23607446189274	-1.18558614644336
C	-3.17394541070804	0.07964600935573	-0.19098569860712
C	-4.08799057723808	-0.28933238132113	2.37013682814112
C	-4.51778885814305	-0.14391778300847	0.01106008893892
N	-2.28937140350559	0.12448656189905	0.81215305816922
C	-2.71174031383394	-0.06166564091028	2.11682413784127
C	-4.97702856535803	-0.33047612314572	1.33161802163580
H	-5.19166122377852	-0.16697213174223	-0.83282763553003
H	-6.02755187348691	-0.50537885732799	1.52338921563257
H	-4.42630881564944	-0.43092400130157	3.38589706011753
N	-0.38413095472603	0.19142624197694	2.64313190287887
C	-0.89772778612917	-0.04540642849768	5.37600236674130
C	-1.70889352138100	-0.01080130987832	3.10330308529466
C	0.62467143223090	0.30095819116308	3.54862381128374
C	0.42790001706948	0.18427229854284	4.88999980224462
C	-1.93318556235697	-0.13405867428441	4.49519419377182
H	1.60816942098471	0.47538447848095	3.13900195508802
H	1.26646044820018	0.26665896318073	5.56579386605786
H	-2.94015788077727	-0.29422723812329	4.85184079852938
H	-1.07561382385269	-0.14055382359785	6.43869787881881
C	1.75385499548518	0.59981063498201	0.57021557827968

C	-0.44125730588292	2.38303658761989	0.86706947925917
N	2.89955045897776	0.70082615706434	0.49074477425594
N	-0.58156248386255	3.51750494187044	0.99613984511344
H	4.77310594948542	0.01787825502504	0.97957553818427
C	4.31499696331156	0.82949465295661	0.41650277199623
H	4.60537974612893	1.78834710919361	0.84305099341970
H	4.62175486521123	0.77761298009530	-0.62677546711634
H	-1.58474169607209	5.24552412833251	0.53801027185553
C	-0.74596012561356	4.92188824617378	1.15252651311510
H	0.16802783553727	5.42243302530656	0.83793030813886
H	-0.95043330554200	5.13374746810027	2.20098694892175

$[Ru(bpy)_2(MeNC)_2]^{2+}$ ^{3}LF State:

Ru	-0.15108977439116	0.48625206547411	0.61865814932622
H	0.25542400414498	-0.11057784908926	-5.39431942845857
C	0.02670648138670	-0.00975504686954	-4.34176162532643
N	-0.53331844182973	0.24369645693551	-1.65021306935130
C	0.15312399671869	-1.10018389404440	-3.49562789004062
C	-0.38812655411501	1.21029961546162	-3.82202194613990
C	-0.65429671809149	1.29436595283753	-2.46642620142873
C	-0.14252694003180	-0.94249081800768	-2.14490961584796
H	0.49267577998830	-2.05128917128854	-3.87790493962580
H	-0.50487443908489	2.08188811342381	-4.45085259619948
H	-0.97708575037703	2.22117556681778	-2.00940394077117
C	-0.02887364973686	-2.02770437207794	-1.15758254231714
C	0.20820432842609	-3.94780189300697	0.78149169438667
C	0.00644235002180	-3.36890471539822	-1.52419741723095
N	0.03445679146374	-1.65399551341198	0.13952386657401
C	0.15326728214550	-2.59795892042689	1.07952518072738
C	0.12736155600598	-4.34063466962003	-0.54588207980714
H	-0.07505721996920	-3.64870378090288	-2.56375283462603
H	0.20400098235541	-2.25380381571089	2.09986021351594
H	0.15270185778956	-5.38749210406713	-0.81735580953914
H	0.30856461708060	-4.66727144786558	1.58180563414961
H	-2.82102923123289	0.40480279344043	-1.03049985632150
C	-3.21056710791466	0.23496941209432	-0.03979556148850
C	-4.09634987616756	-0.20107923463649	2.49351173845422
C	-4.56314536405631	0.02887100669573	0.16552024210947
N	-2.31903522561407	0.23398551921449	0.95703447975536
C	-2.75168532615321	0.02698813934231	2.21996561191768
C	-5.01364864746616	-0.19878564722240	1.45711353844342
H	-5.23908975894233	0.04490491427067	-0.67772266841140
H	-6.06195601045125	-0.37685114491933	1.65623718730745
H	-4.41645332727426	-0.39183465546398	3.50671490760199
N	-0.46003873206630	-0.07575976049143	2.84918594117978
C	-0.98238243799248	0.24222370104353	5.54148287172251
C	-1.72696803943240	0.05217540097239	3.27569481125280
C	0.54135322793739	-0.05243214981695	3.73272290709831
C	0.32431845652210	0.10049084820870	5.09101222512437
C	-2.02195877523361	0.21939060235161	4.62540742233808
H	1.53978488991574	-0.16334280779387	3.32903582795027
H	1.16183090766575	0.10656288288641	5.77459179715730
H	-3.04287346772527	0.34577008723485	4.95396416904989
H	-1.19051903016887	0.37500818887822	6.59473434238416
C	1.82205108699479	0.59279178754965	0.43951252886063

C	-0.43627359497858	2.42180996732130	0.93169975475014
N	2.97243788634312	0.58256183534947	0.32963181486356
N	-0.66955697179279	3.53712012321763	1.12514532379797
H	4.78596058784416	-0.27410260605671	0.77271499814946
C	4.38864940759843	0.56263628860731	0.19969574260178
H	4.79556145684279	1.49968053129422	0.57652321514895
H	4.64521177228028	0.44379474576691	-0.85235415024532
H	-1.11952633289442	5.40545810666352	0.40351669646163
C	-0.96267104600311	4.90872137221338	1.36026831438704
H	-0.12457158983074	5.36820268233955	1.88203370718211
H	-1.86471832645334	4.98132931028213	1.96601031744679

$[Ru(bpy)_2(C_4H_{10}N_4)]^{2+}$ S_0 State:

Ru	7.90329843428381	4.99301561364723	4.14664876248089
N	6.70745969316839	5.17362409480492	5.83683805956616
N	8.90773562966944	6.50811322309262	5.26565360724733
N	5.85737007903757	3.46520581833174	2.53069494260014
H	5.50188532672654	4.37130548267408	2.27403736283644
N	7.40756313947240	2.23186372639300	3.65148317343165
H	6.83629793861603	1.39459954903984	3.66417240994374
C	8.30985252021171	6.85160315746666	6.42866876929689
C	5.59549730228672	4.45939737835447	6.06783131981782
H	5.33529984391772	3.72648304672720	5.32001313496975
C	7.08181986074587	6.11034294451161	6.74471310567347
C	10.03554623355860	7.13086674727044	4.90313642655281
H	10.47408679884190	6.82019531275273	3.96597792912042
C	4.80927045266386	4.64072156883080	7.18956806676971
H	3.92273294296546	4.03640815176094	7.32145943974363
C	6.92339447386686	3.45119199855368	3.32355927575880
C	8.84794332293261	7.83769605077986	7.25044278824417
H	8.36358400807279	8.10449895038507	8.17743076681038
C	6.33007731999355	6.33799378306696	7.89310303647976
H	6.63888667797604	7.08884409632971	8.60390080184162
C	10.62188763377300	8.11804975382228	5.67432423321840
H	11.53399300255790	8.59083737503497	5.33780665939124
C	10.01526914446680	8.47759442378057	6.87035096921756
H	10.44444770071680	9.24570656638919	7.49967908832563
C	5.18271221686852	5.59940506246400	8.12122362174632
H	4.59125756762195	5.76970896105804	9.01069235033722
C	5.17092219864635	2.29688440584121	1.99749148580046
H	4.37090851660196	2.63570352143997	1.34427741969406
H	4.73550018627984	1.69438281388424	2.79930617008419
H	5.85779663636978	1.67266098650702	1.42138184212510
N	9.95042796820910	3.46745190966550	5.76337993780381
H	10.30532537177020	4.37378099054396	6.01999472390745
N	8.40118669095024	2.23248080913868	4.64313811707657
H	8.97307085611939	1.39564512045364	4.63087226359936
C	8.88435133886518	3.45234038493139	4.97058798955564
C	10.63757486676110	2.29967068818123	6.29688721594893
H	11.43709256887330	2.63907899691827	6.95040295081904
H	11.07377576591540	1.69747739640097	5.49525337880045
H	9.95092885002904	1.67493358035722	6.87271186661938
N	9.09908040391925	5.17344936308923	2.45629738446451
N	6.89879600210929	6.50773905680750	3.02715040990280
C	7.49759215689432	6.85240127691360	1.86496340361582

C	10.21049259292920	4.45846192017574	2.22487928413481
H	10.47014546027020	3.72486413809706	2.97221653484129
C	8.72544441402939	6.11085343246996	1.54883622253450
C	5.77076225363991	7.13025857021055	3.38943658584708
H	5.33131284586728	6.81853385853029	4.32581264637250
C	10.99691348495590	4.63983490780753	1.10330530079794
H	11.88294561644450	4.03485010495353	0.97110180145876
C	6.96052648906759	7.83975888888364	1.04403417438844
H	7.44581527015990	8.10756640729157	0.11782066644376
C	9.47750387827132	6.33870764916057	0.40069076868680
H	9.16924281405622	7.09023846033325	-0.30962987796752
C	5.18533864053227	8.11855513974498	2.61900137390144
H	4.27302735964880	8.59109636274794	2.95530483872832
C	5.79311741066450	8.47955865186850	1.42398751978257
H	5.36475816715402	9.24869561466495	0.79535928814010
C	10.62435347418580	5.59944808918872	0.17223220684497
H	11.21606318479540	5.76989566547475	-0.71703499617548

[Ru(bpy)$_2$(C$_4$H$_{10}$N$_4$)]$^{2+}$ T$_1$ State:

Ru	7.90111137793118	5.01591094565685	4.13703375159422
N	6.72261758942090	5.15046147609996	5.81756553631836
N	8.91853886556986	6.50826841842345	5.20810218336256
N	5.97710167510248	3.43329961101805	2.38110002669463
H	5.66477746809765	4.33972514025867	2.07100165807850
N	7.41596002176285	2.22246933594623	3.66859576943882
H	6.96205104769931	1.32799560423544	3.52736673961711
C	8.31311435979900	6.82735143447068	6.41829407222349
C	5.63949335330932	4.39465989103437	6.08462915058167
H	5.37881733164304	3.65973411214100	5.33640210296621
C	7.14160566764735	6.10632939938646	6.74109313228607
C	10.02059252998850	7.17254621299023	4.81679819451233
H	10.43429175171930	6.88125483212579	3.86092711568627
C	4.88507873159676	4.54126152223458	7.22011806140226
H	4.01794969887918	3.91594021452903	7.37516216821660
C	6.96572081868937	3.40806378599537	3.25456900639549
C	8.89225745481451	7.83763137207898	7.22746871292785
H	8.43478814591802	8.08888103946389	8.17356162489174
C	6.38518261349978	6.28399542966866	7.92637792831014
H	6.69591937016764	7.03074568236141	8.64255343482112
C	10.61044593444040	8.15634393867759	5.57041610195823
H	11.50001000245890	8.65399133833677	5.21247120848551
C	10.02052497870880	8.48958322842164	6.81523761187073
H	10.45966192661810	9.25914017851132	7.43700507896069
C	5.27587408168200	5.52303963168255	8.16491480116232
H	4.70267056792807	5.66642346454857	9.07164202337029
C	5.30206904829933	2.26157367658461	1.83572372021711
H	4.52229165399507	2.59989233775451	1.15912784906743
H	4.84684431792883	1.67156199714459	2.63465973316352
H	6.00566645979004	1.63357160506903	1.28491094671072
N	9.81359756571795	3.50645263248287	5.91938958936817
H	10.11551005878910	4.42940729874288	6.19030380951229
N	8.36794339455649	2.24168533944874	4.68553436826640
H	8.81310695962693	1.35134742870174	4.87216430850211
C	8.82309345347002	3.43967720524660	5.05171440416419
C	10.48525016987030	2.36166496781474	6.52234174260530
H	11.26828399746110	2.73126420072775	7.17834728705863
H	10.93590657334870	1.72966386559933	5.75358617998582
H	9.78044658061742	1.76594219477123	7.10649010177284
N	9.05945399860171	5.16506196718105	2.40113265313278
N	6.91553364177435	6.56566411948274	3.05255845788715
C	7.48700895390512	6.89060271228259	1.87131988190113

C	10.13612053277410	4.41085518187233	2.14514956257036
H	10.38253254041250	3.65783874052738	2.87836191353245
C	8.68810170618256	6.12281184446538	1.51762401026960
C	5.82147262837393	7.21348264869549	3.46550787483188
H	5.41543451150611	6.90845704203724	4.41888024015790
C	10.90388598904070	4.58314095878506	1.00906892685763
H	11.76704194345560	3.95309036199186	0.84859269865244
C	6.94702035289444	7.89149080742580	1.07203175278401
H	7.40381866831337	8.14747111074318	0.12836020206336
C	9.42051635271539	6.33973505220581	0.35668292285305
H	9.12169100529928	7.10798717080300	-0.33940020610363
C	5.23635445028910	8.21691350090788	2.71507449996275
H	4.35126711754728	8.71581050372225	3.08336595814805
C	5.81063987263287	8.55885079852101	1.49852673462110
H	5.37943148874218	9.33898806889477	0.88570735581144
C	10.53962761877290	5.56631159463745	0.10041653486642
H	11.11814202820150	5.72921582443127	-0.79880221132791

NMR Spectroscopy

$[Ru(bpy)_2(MeNC)_2](PF6)_2$

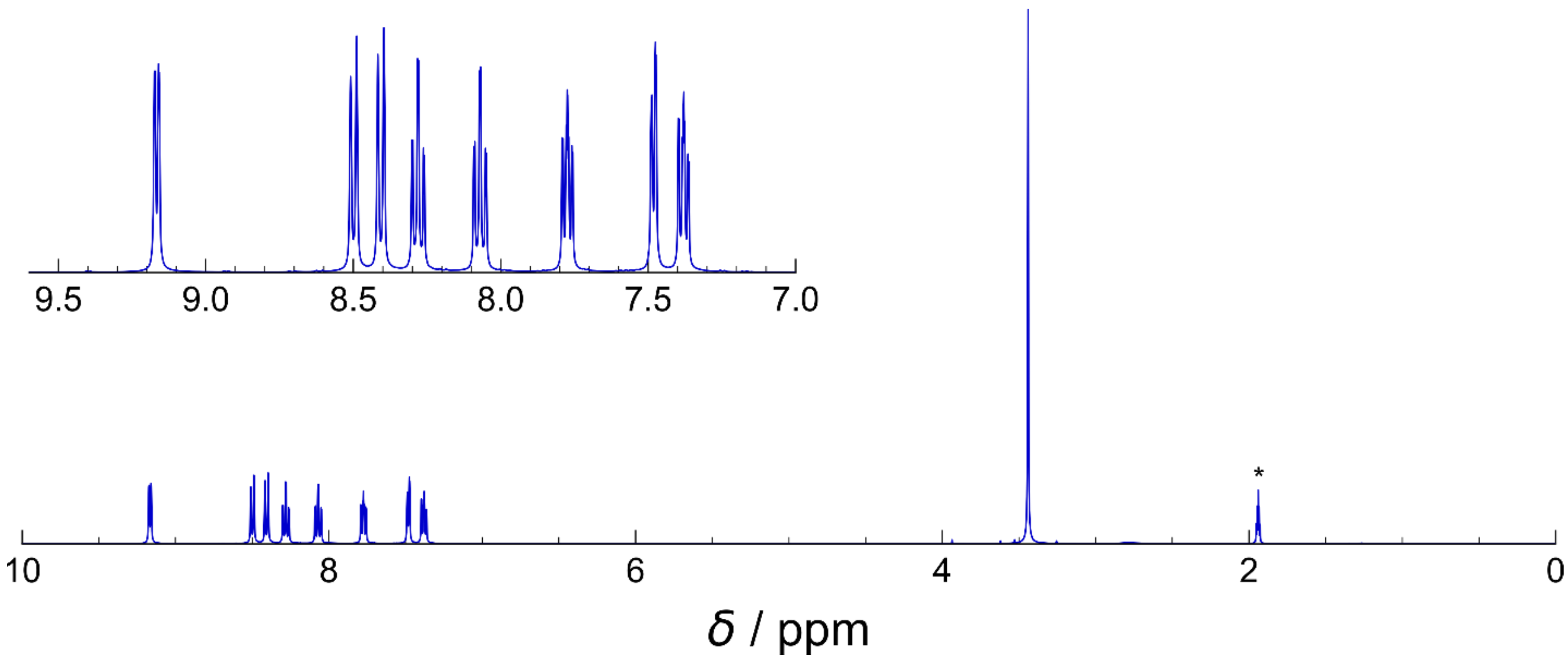

Figure S47. ^{1}H NMR spectrum of $[Ru(bpy)_2(MeNC)_2](PF_6)_2$ recorded in CD_3CN (400 MHz). Asterisk denotes residual non-perdeuterated solvent. Inset: Magnification of aromatic region.

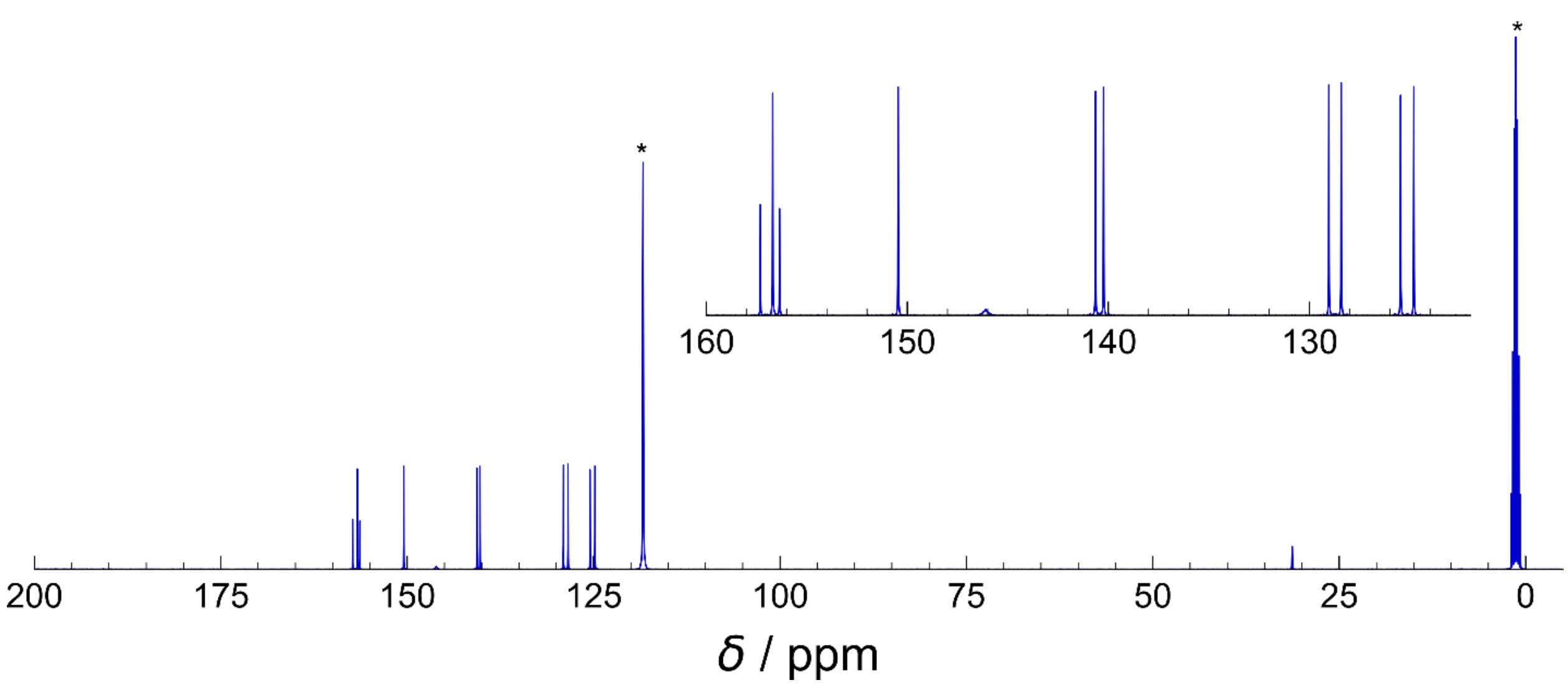

Figure S48. ^{13}C NMR spectrum of $[Ru(bpy)_2(MeNC)_2](PF_6)_2$ recorded in CD_3CN (101 MHz). Asterisk denotes residual non-perdeuterated solvent. Inset: Magnification of aromatic region.

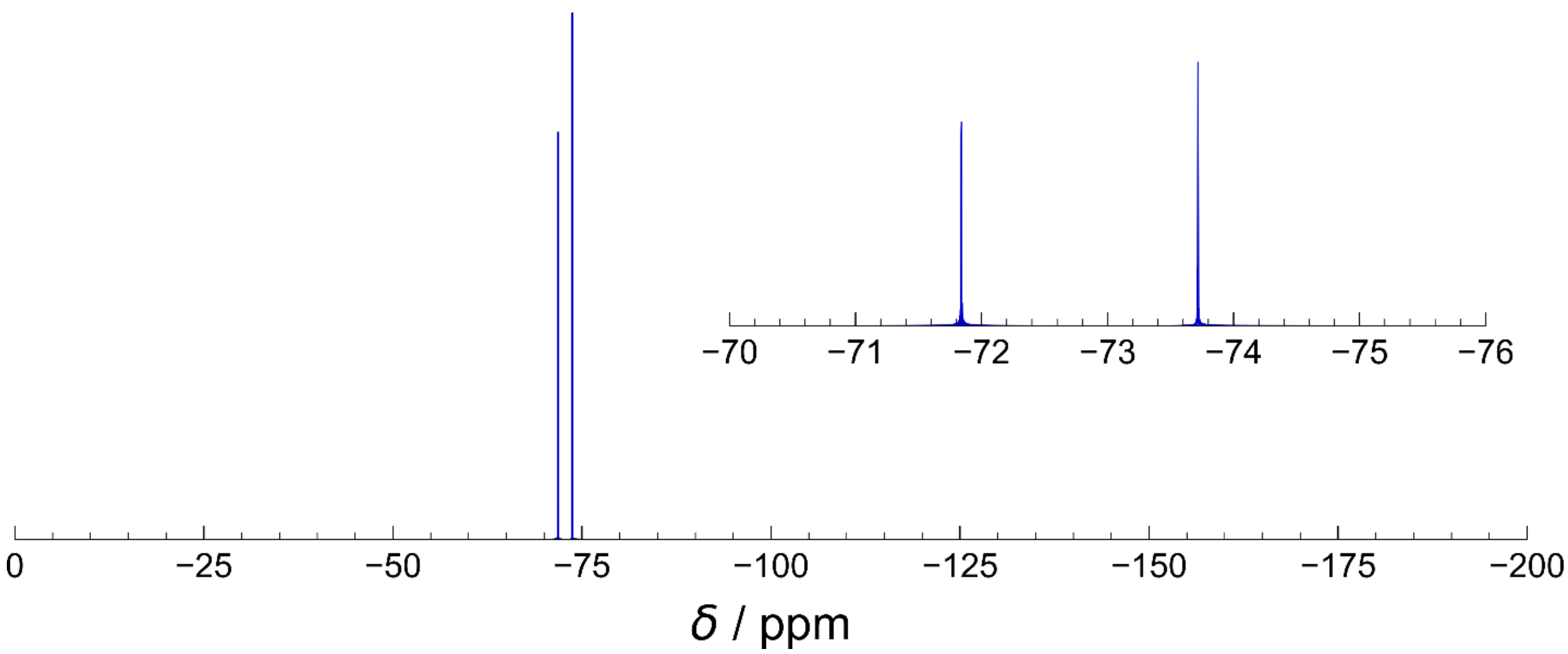

Figure S49. ^{19}F NMR spectrum of $[Ru(bpy)_2(MeNC)_2](PF_6)_2$ recorded in CD_3CN (376 MHz). Inset: Magnification of region of interest.

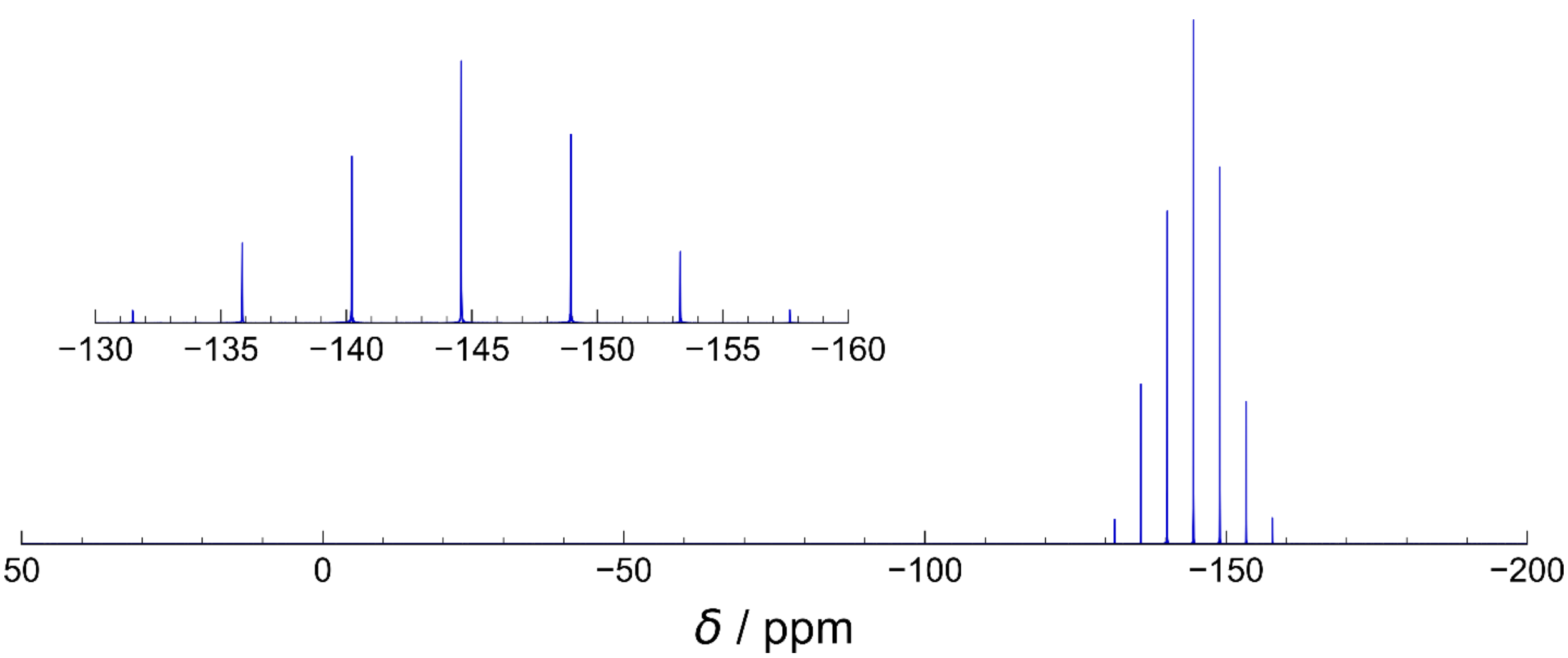

Figure S50. ^{31}P NMR spectrum of $[Ru(bpy)_2(MeNC)_2](PF_6)_2$ recorded in CD_3CN (162 MHz). Inset: Magnification of region of interest.

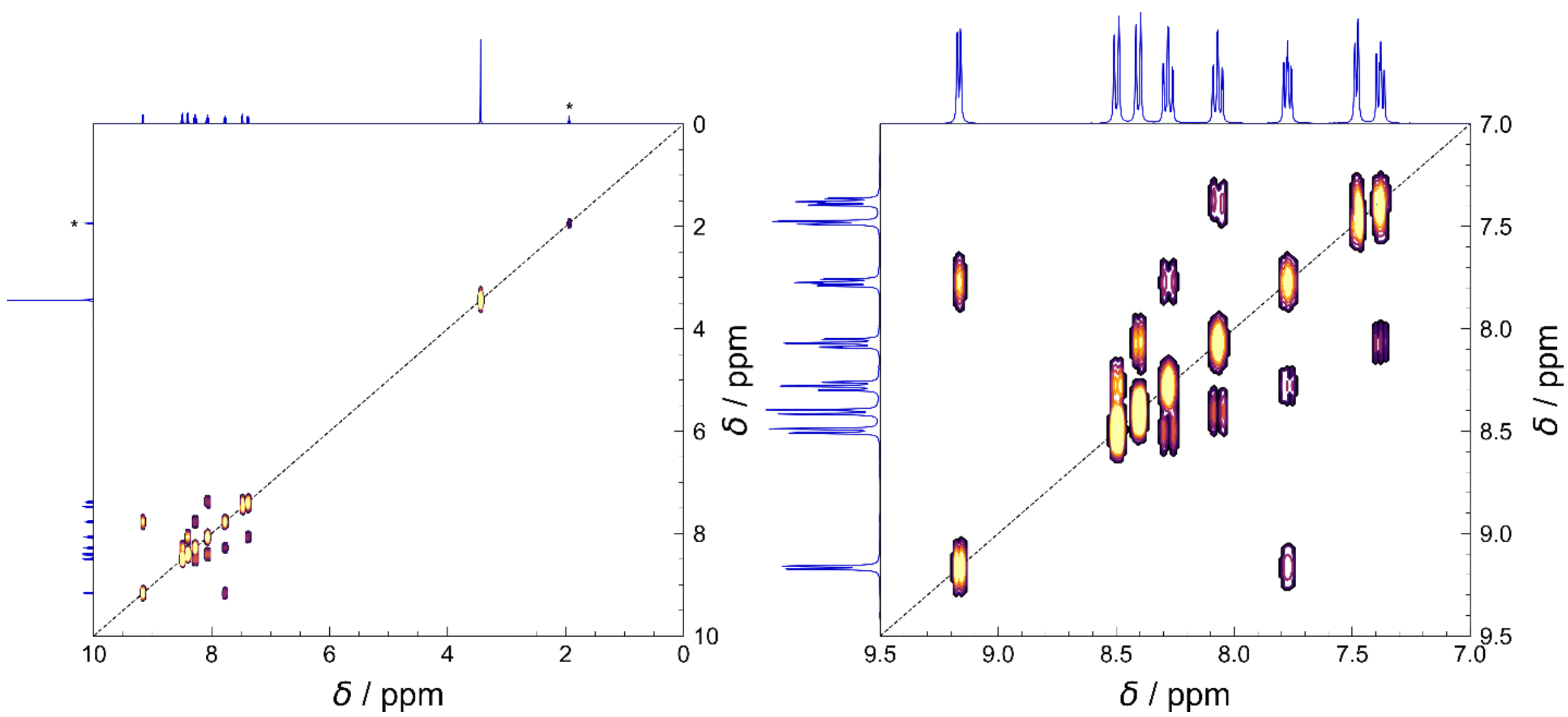

Figure S51. ^1H-^1H COSY spectrum of $[Ru(bpy)_2(MeNC)_2](PF_6)_2$ recorded in CD_3CN. Left: full spectrum; Right: magnification of aromatic region. Asterisk denotes residual non-perdeuterated solvent.

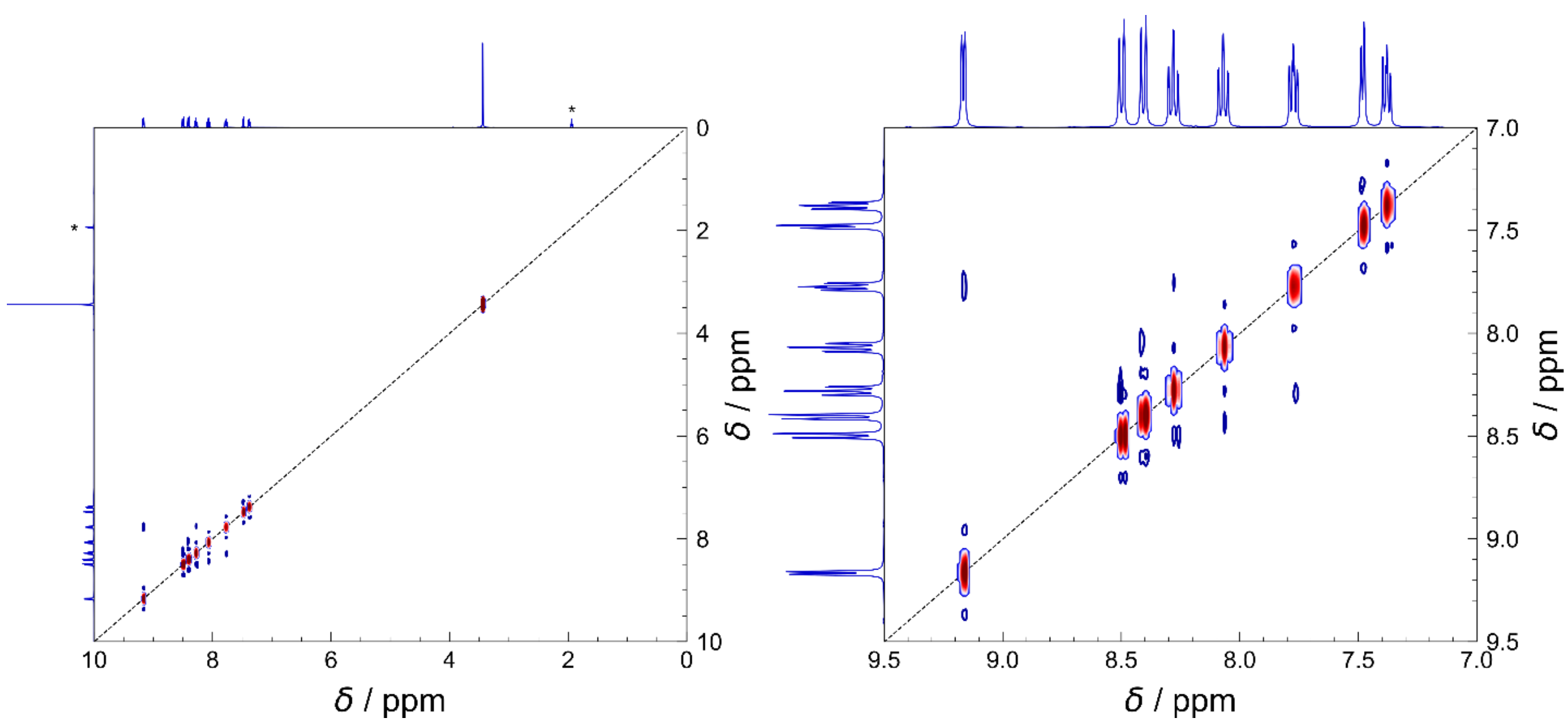

Figure S52. ^1H-^1H NOESY spectrum of $[Ru(bpy)_2(MeNC)_2](PF_6)_2$ recorded in CD_3CN. Left: full spectrum; Right: magnification of aromatic region. Asterisk denotes residual non-perdeuterated solvent.

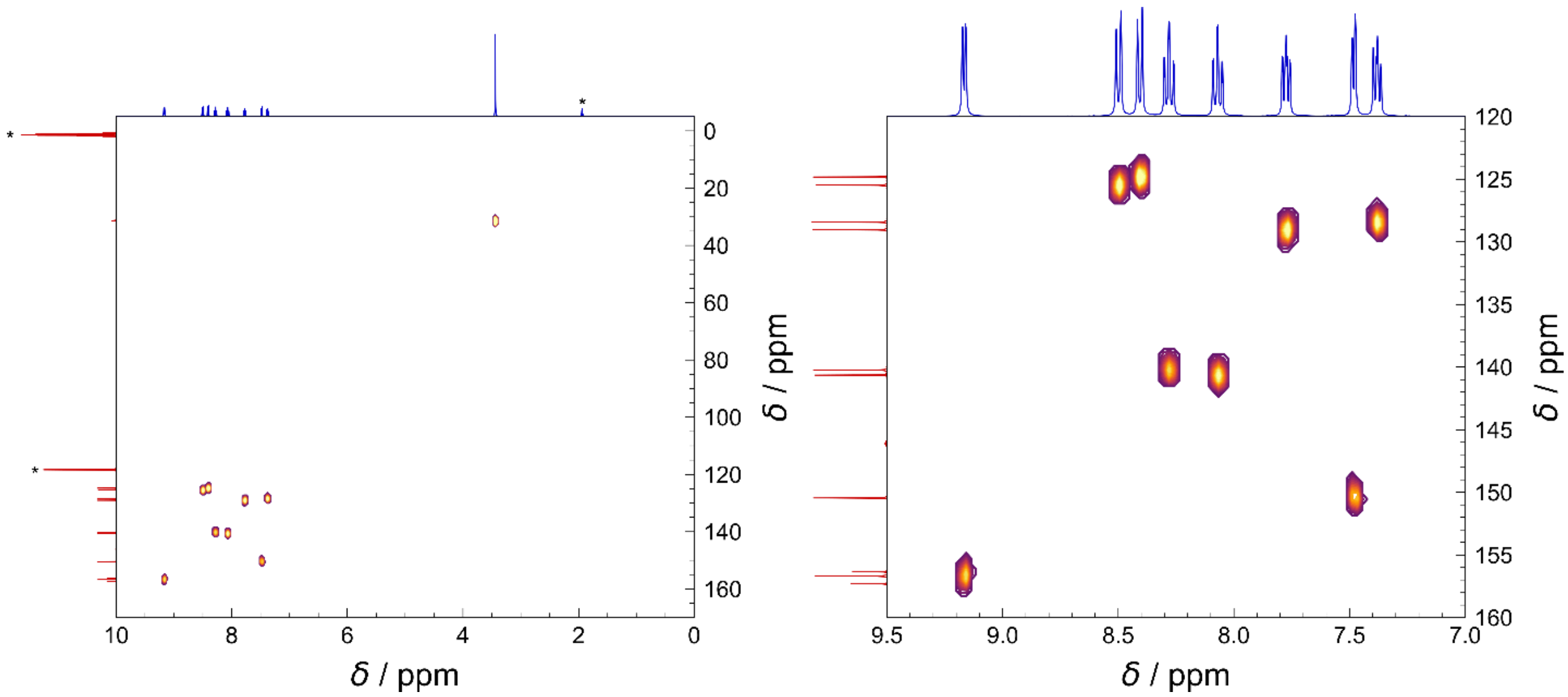

Figure S53. ^{1}H-^{13}C HSQC spectrum of $[Ru(bpy)_2(MeNC)_2](PF_6)_2$ recorded in CD_3CN. Left: full spectrum; Right: magnification of aromatic region. Asterisk denotes residual non-perdeuterated solvent.

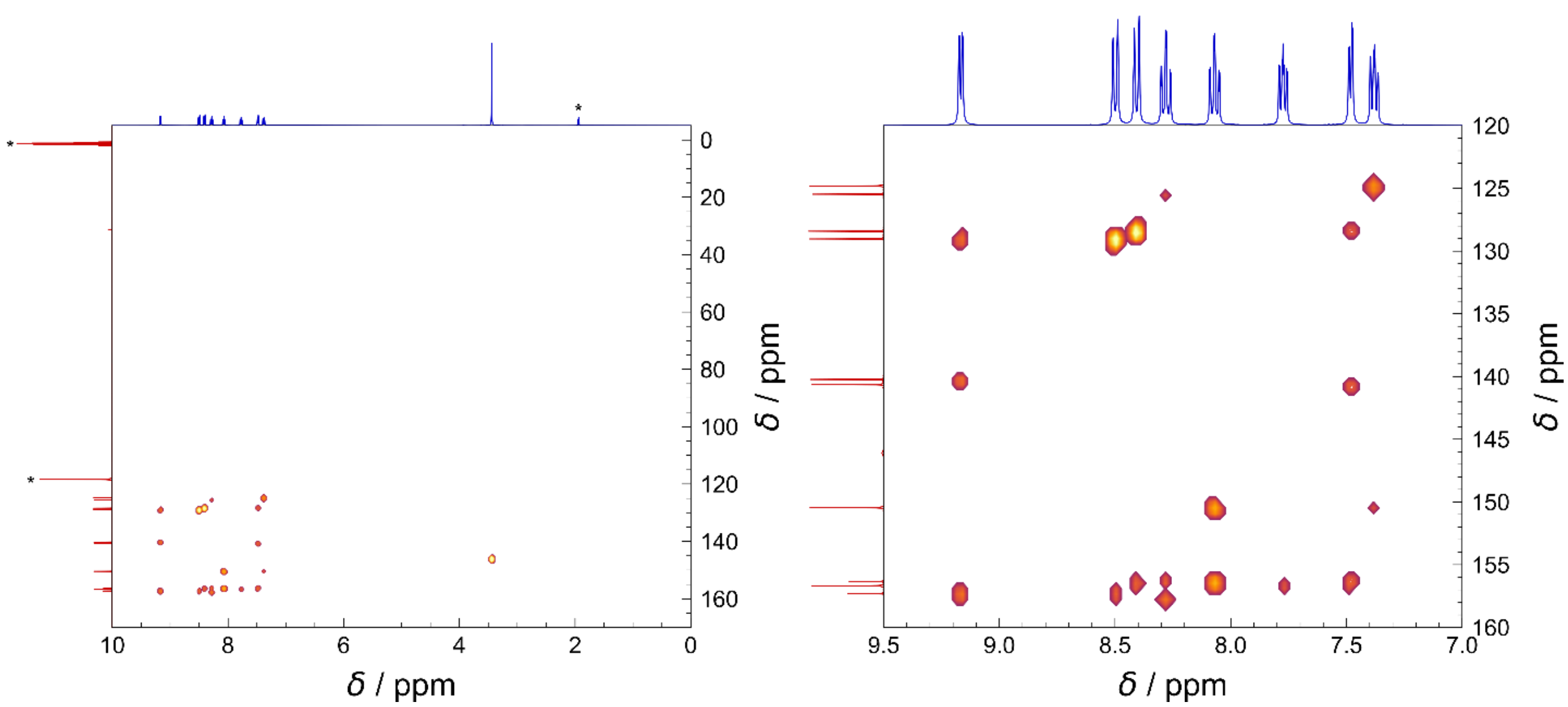

Figure S54. ^{1}H-^{13}C HMBC spectrum of $[Ru(bpy)_2(MeNC)_2](PF_6)_2$ recorded in CD_3CN. Left: full spectrum; Right: magnification of aromatic region. Asterisk denotes residual non-perdeuterated solvent.

[Ru(bpy)2(C4H10N4)2](PF6)2

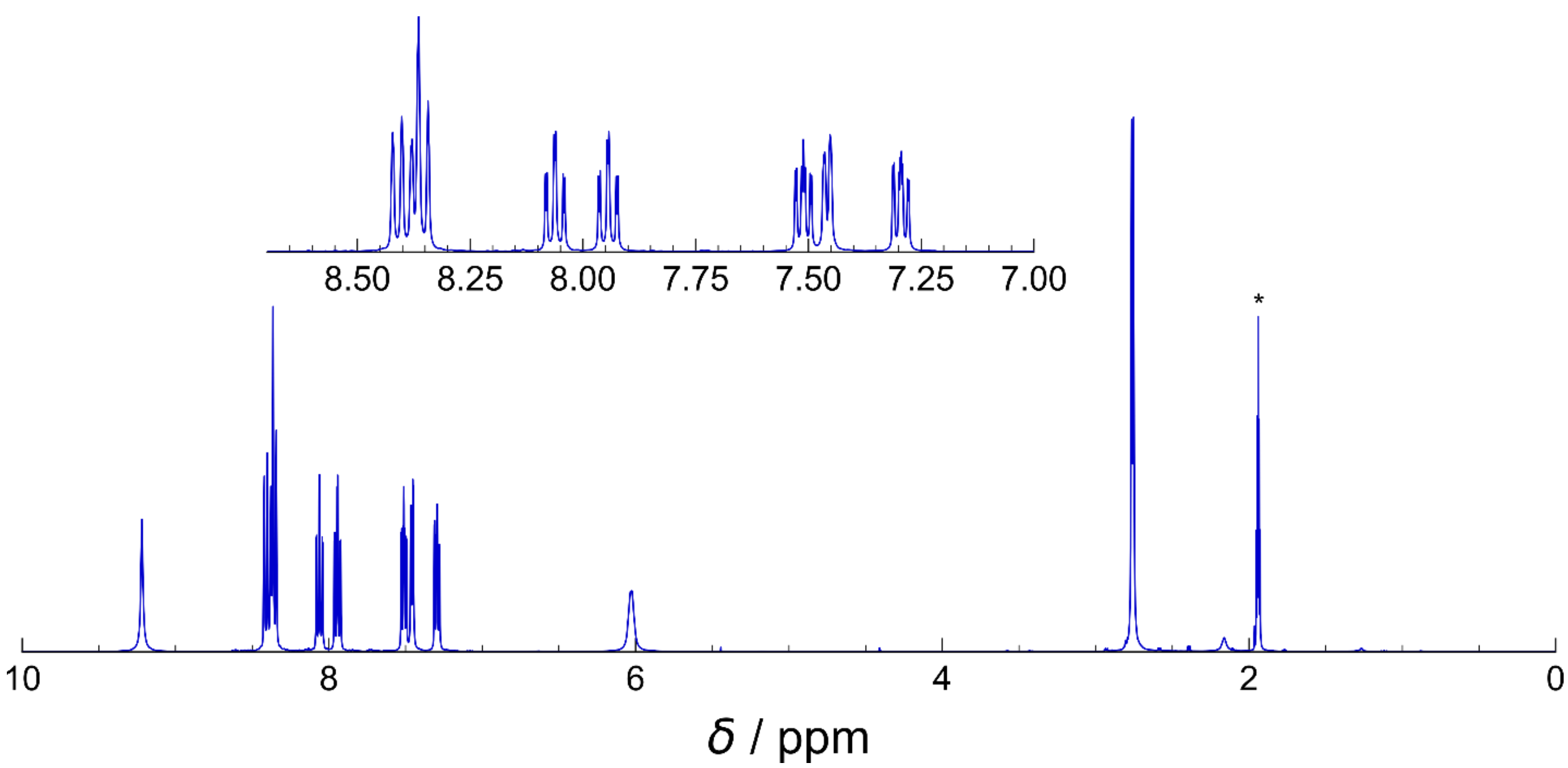

Figure S55. ^{1}H NMR spectrum of [Ru(bpy)$_2$(C$_4$H$_{10}$N$_4$)](PF$_6$)$_2$ recorded in CD$_3$CN (400 MHz). Asterisk denotes residual non-perdeuterated solvent. Inset: Magnification of aromatic region.

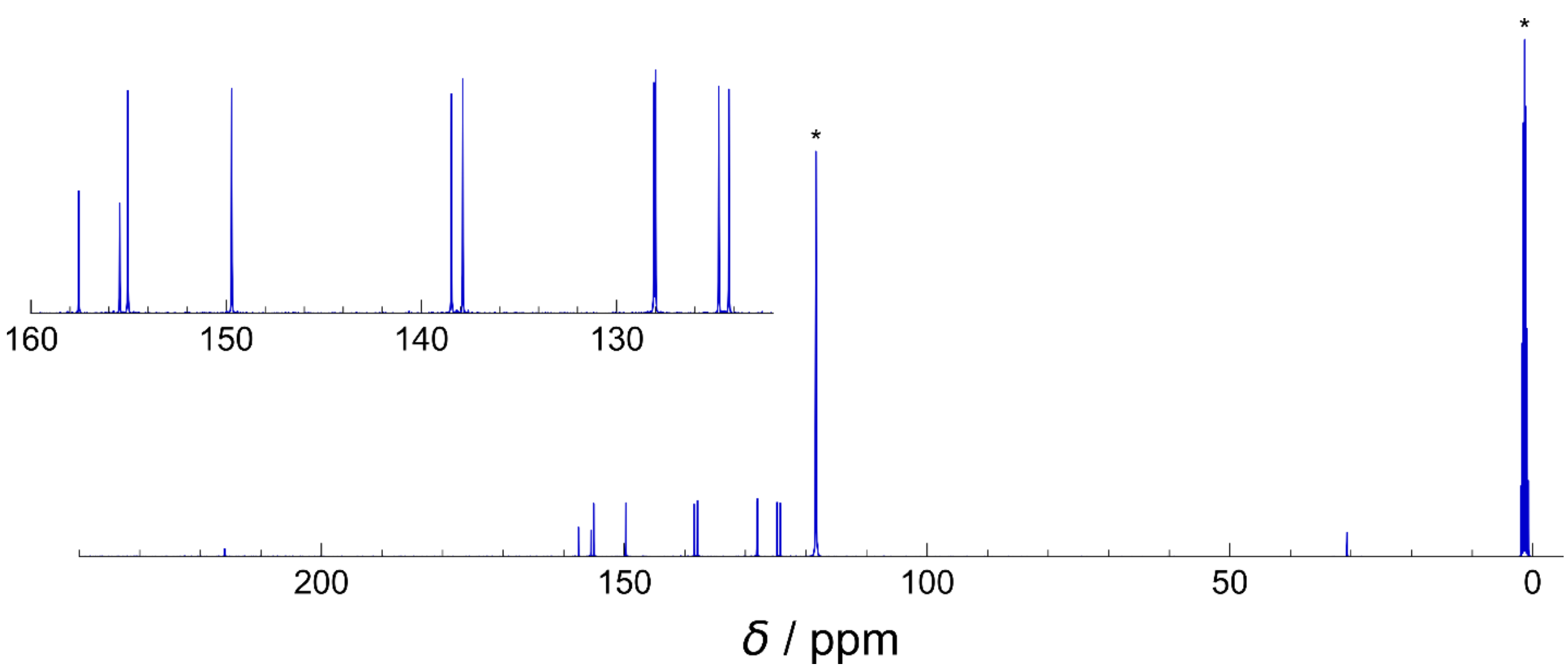

Figure S56. ^{13}C NMR spectrum of [Ru(bpy)$_2$(C$_4$H$_{10}$N$_4$)](PF$_6$)$_2$ recorded in CD$_3$CN (101 MHz). Asterisk denotes residual non-perdeuterated solvent. Inset: Magnification of aromatic region.

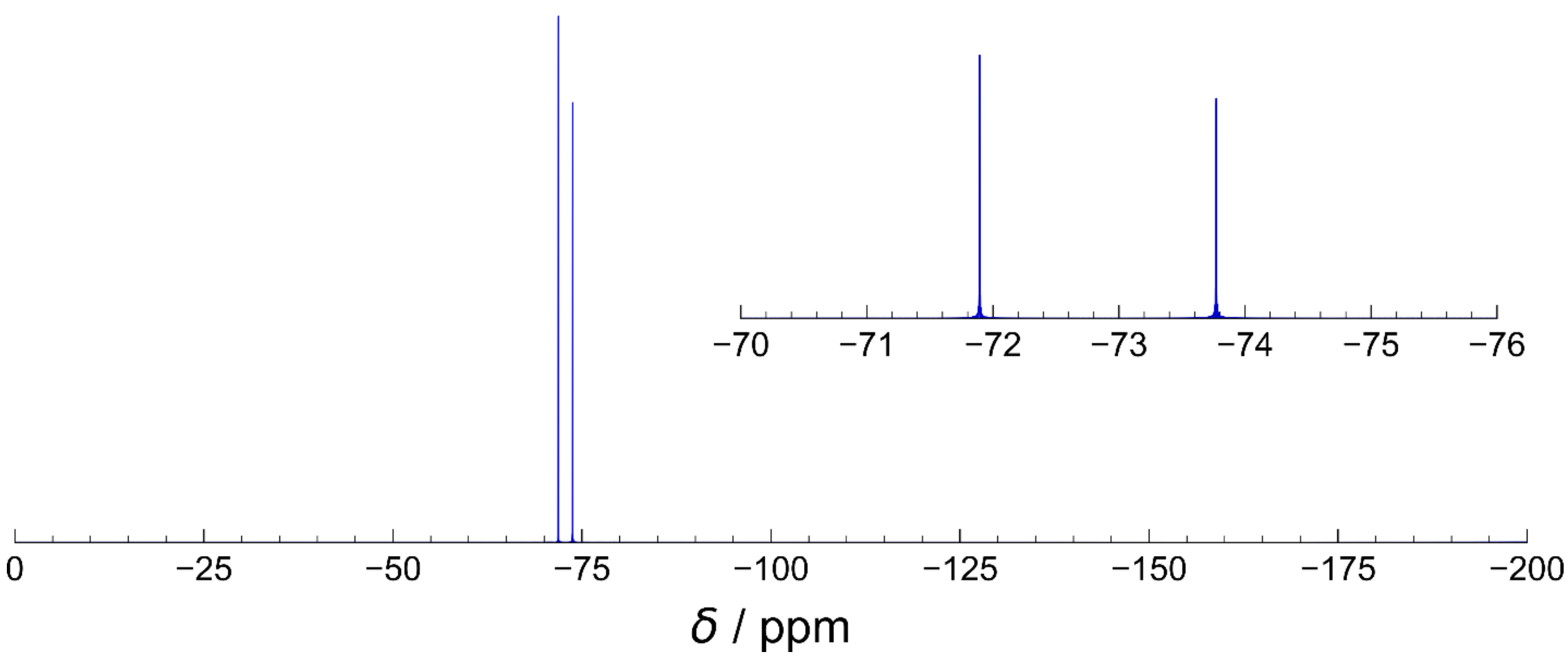

Figure S57. ^{19}F NMR spectrum of $[Ru(bpy)_2(C_4H_{10}N_4)](PF_6)_2$ recorded in CD_3CN (376 MHz). Inset: Magnification of region of interest.

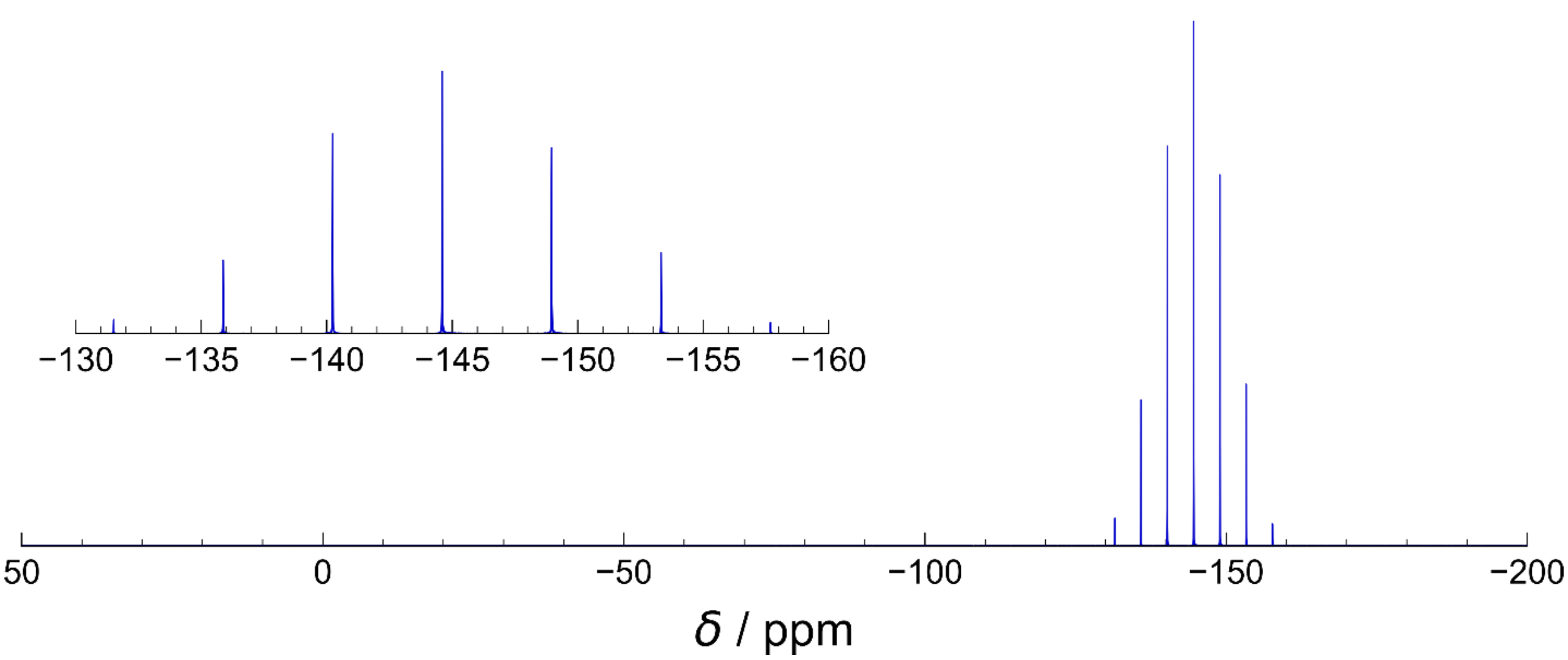

Figure S58. ^{31}P NMR spectrum of $[Ru(bpy)_2(C_4H_{10}N_4)](PF_6)_2$ recorded in CD_3CN (162 MHz). Inset: Magnification of region of interest.

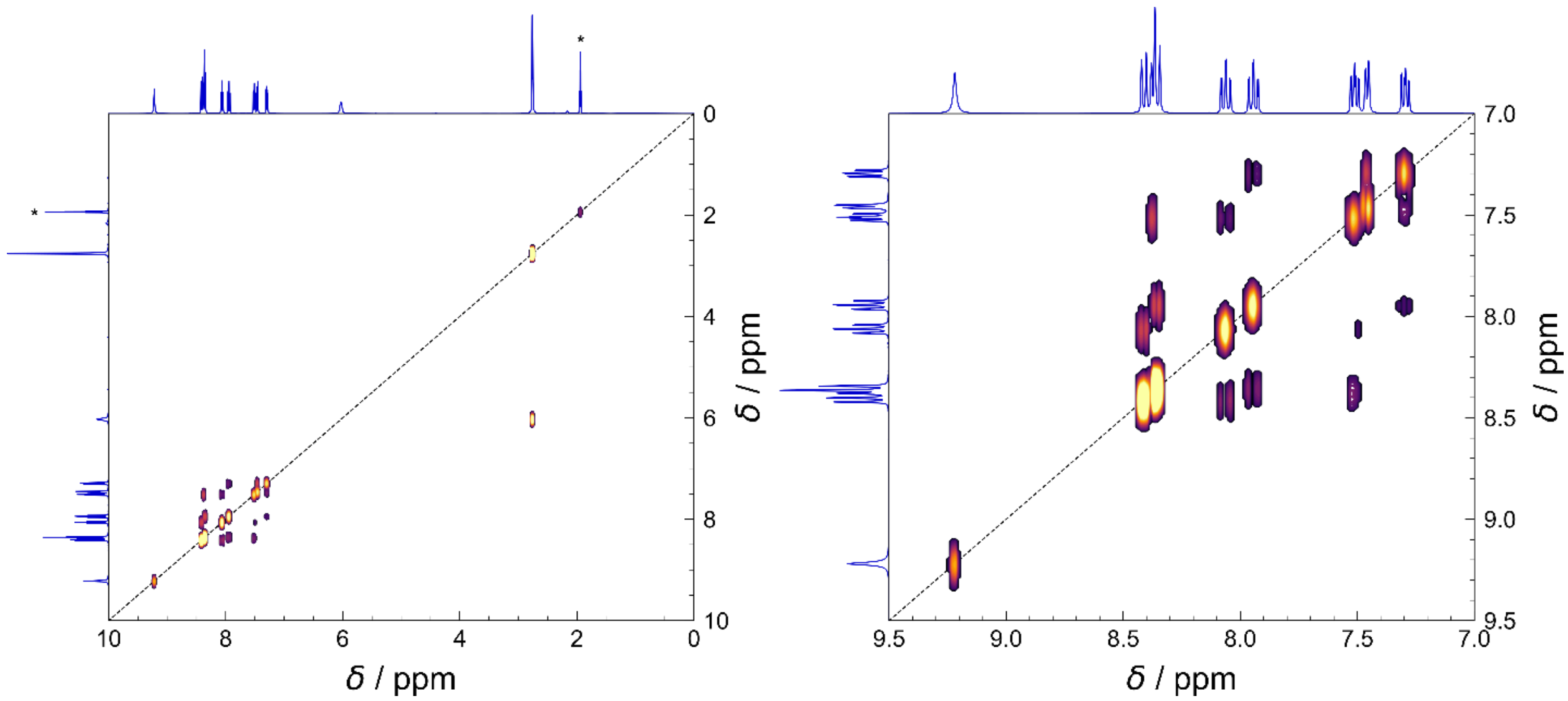

Figure S59. ^1H-^1H COSY spectrum of $[Ru(bpy)_2(C_4H_{10}N_4)](PF_6)_2$ recorded in CD_3CN. Left: full spectrum; Right: magnification of aromatic region. Asterisk denotes residual non-perdeuterated solvent.

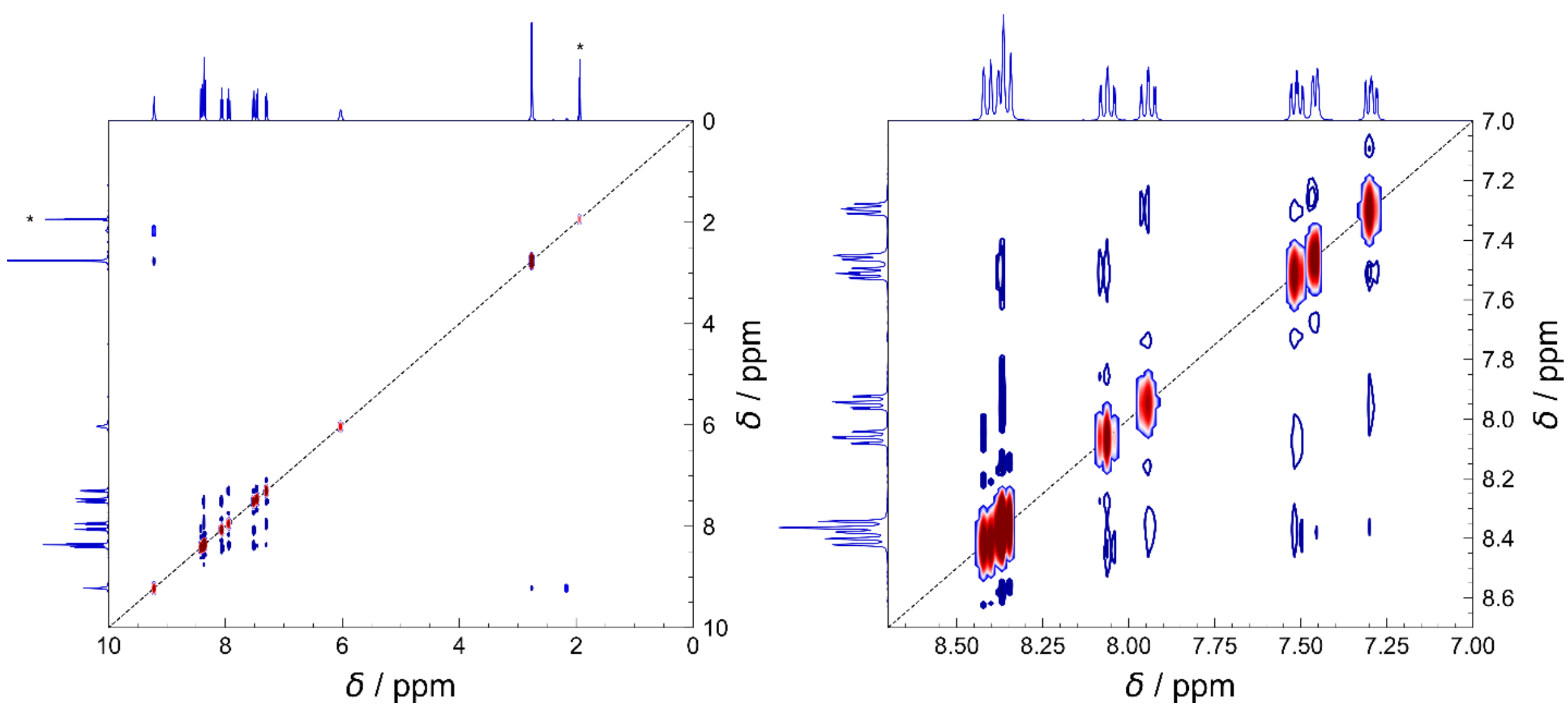

Figure S60. ^1H-^1H NOESY spectrum of $[Ru(bpy)_2(C_4H_{10}N_4)](PF_6)_2$ recorded in CD_3CN. Left: full spectrum; Right: magnification of aromatic region. Asterisk denotes residual non-perdeuterated solvent.

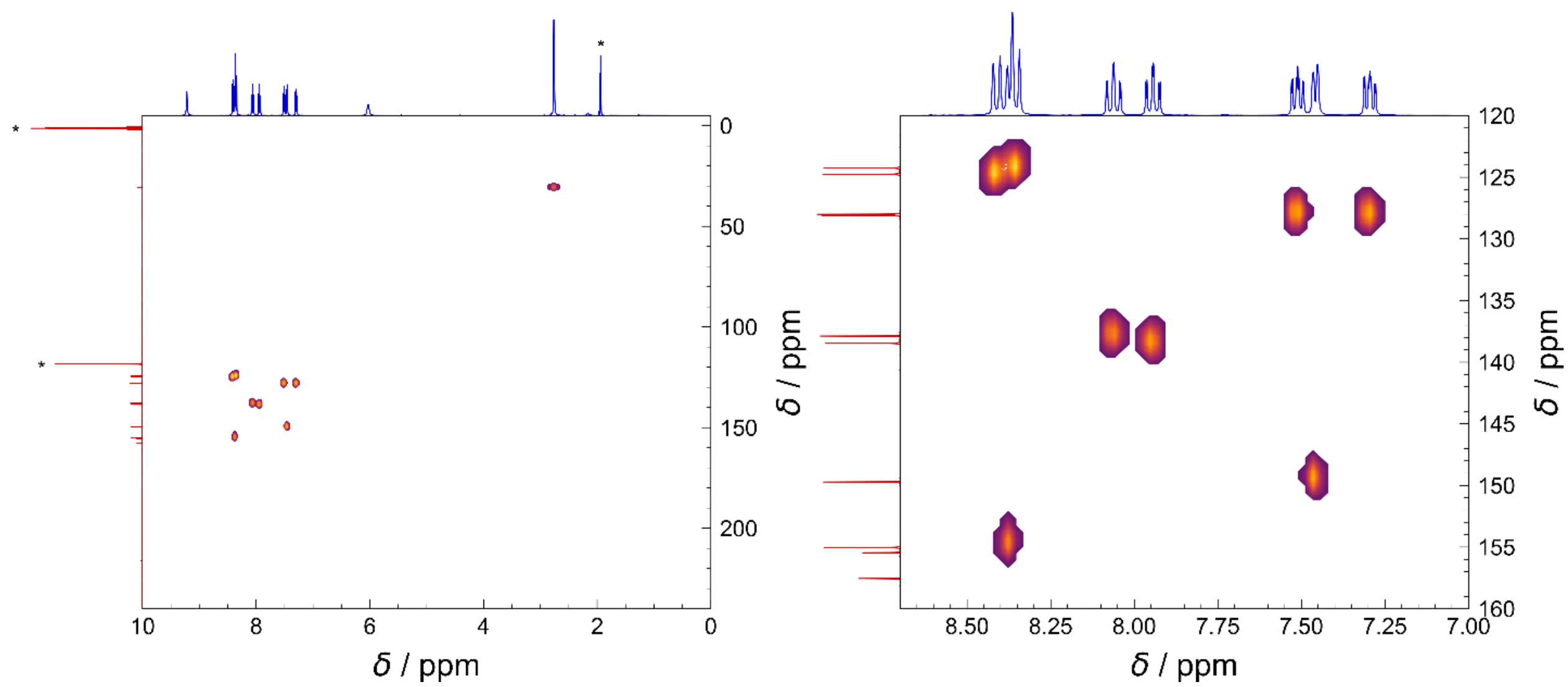

Figure S61. ^{1}H-^{13}C HSQC spectrum of $[Ru(bpy)_2(C_4H_{10}N_4)](PF_6)_2$ recorded in CD_3CN. Left: full spectrum; Right: magnification of aromatic region. Asterisk denotes residual non-perdeuterated solvent.

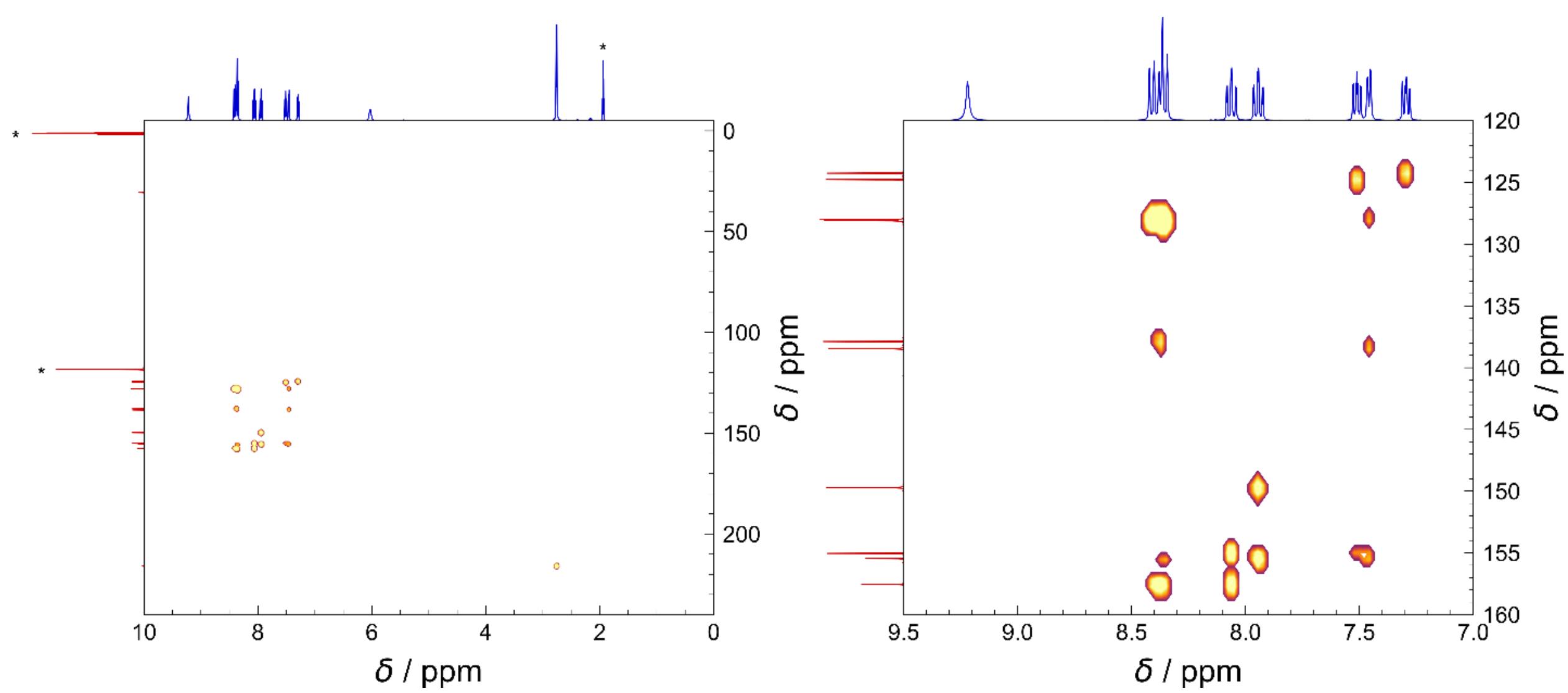

Figure S62. ^{1}H-^{13}C HMBC spectrum of $[Ru(bpy)_2(C_4H_{10}N_4)](PF_6)_2$ recorded in CD_3CN. Left: full spectrum; Right: magnification of aromatic region. Asterisk denotes residual non-perdeuterated solvent.

[Ru(dtbbpy)$_2$(MeNC)$_2$](PF6)$_2$

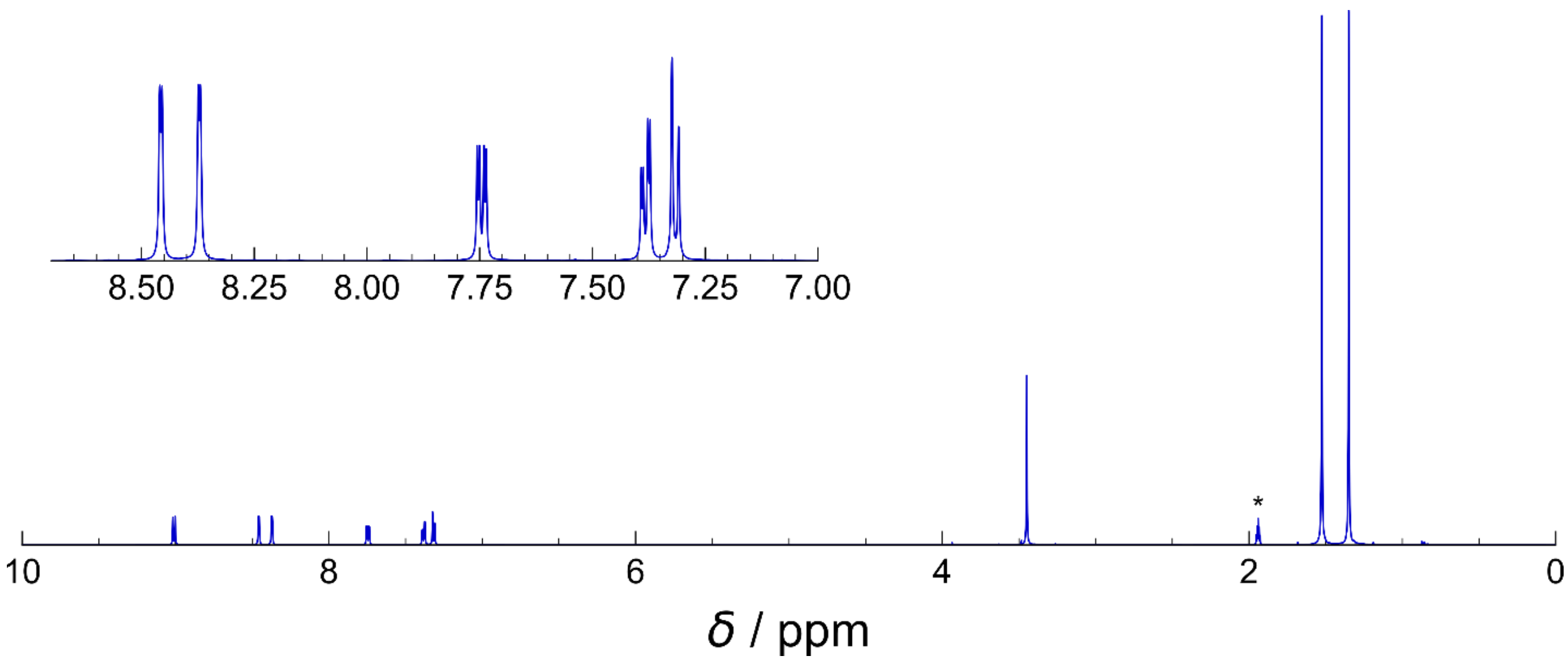

Figure S63. ^{1}H NMR spectrum of [Ru(dtbbpy)$_2$(MeNC)$_2$](PF$_6$)$_2$ recorded in CD_3CN (400 MHz). Asterisk denotes residual non-perdeuterated solvent. Inset: Magnification of aromatic region.

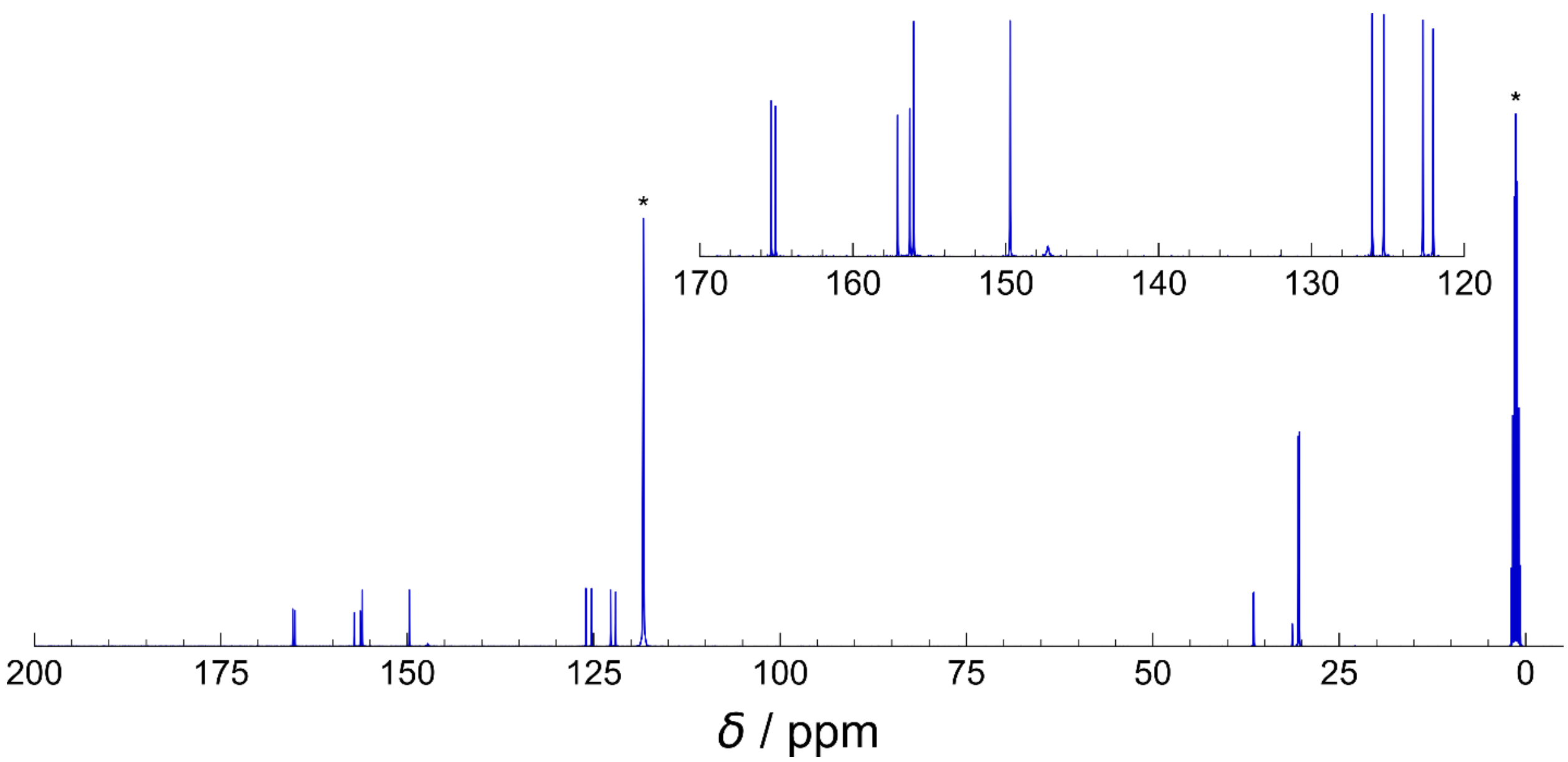

Figure S64. ^{13}C NMR spectrum of [Ru(dtbbpy)$_2$(MeNC)$_2$](PF$_6$)$_2$ recorded in CD_3CN (101 MHz). Asterisk denotes residual non-perdeuterated solvent. Inset: Magnification of aromatic region.

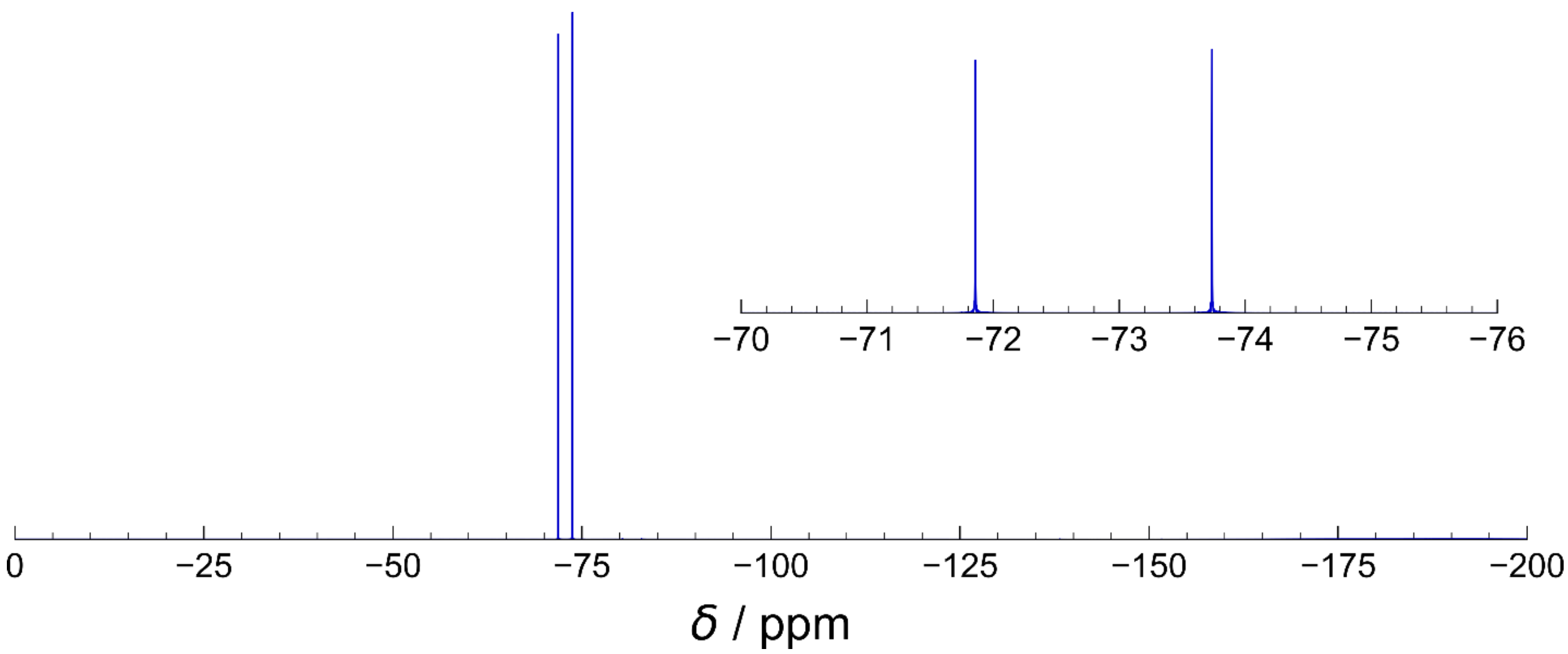

Figure S65. ^{19}F NMR spectrum of $[Ru(dtbbpy)_2(MeNC)_2](PF_6)_2$ recorded in CD_3CN (376 MHz). Inset: Magnification of region of interest.

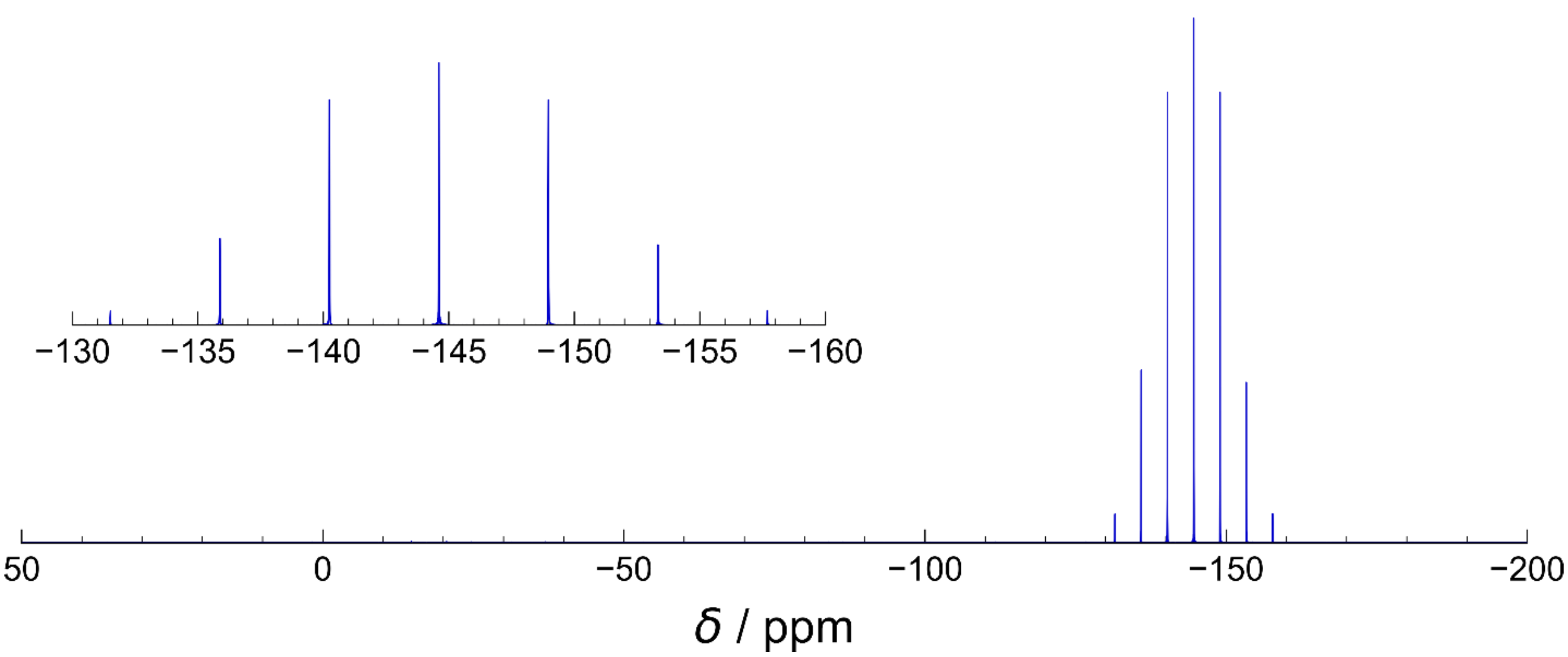

Figure S66. ^{31}P NMR spectrum of $[Ru(dtbbpy)_2(MeNC)_2](PF_6)_2$ recorded in CD_3CN (162 MHz). Inset: Magnification of region of interest.

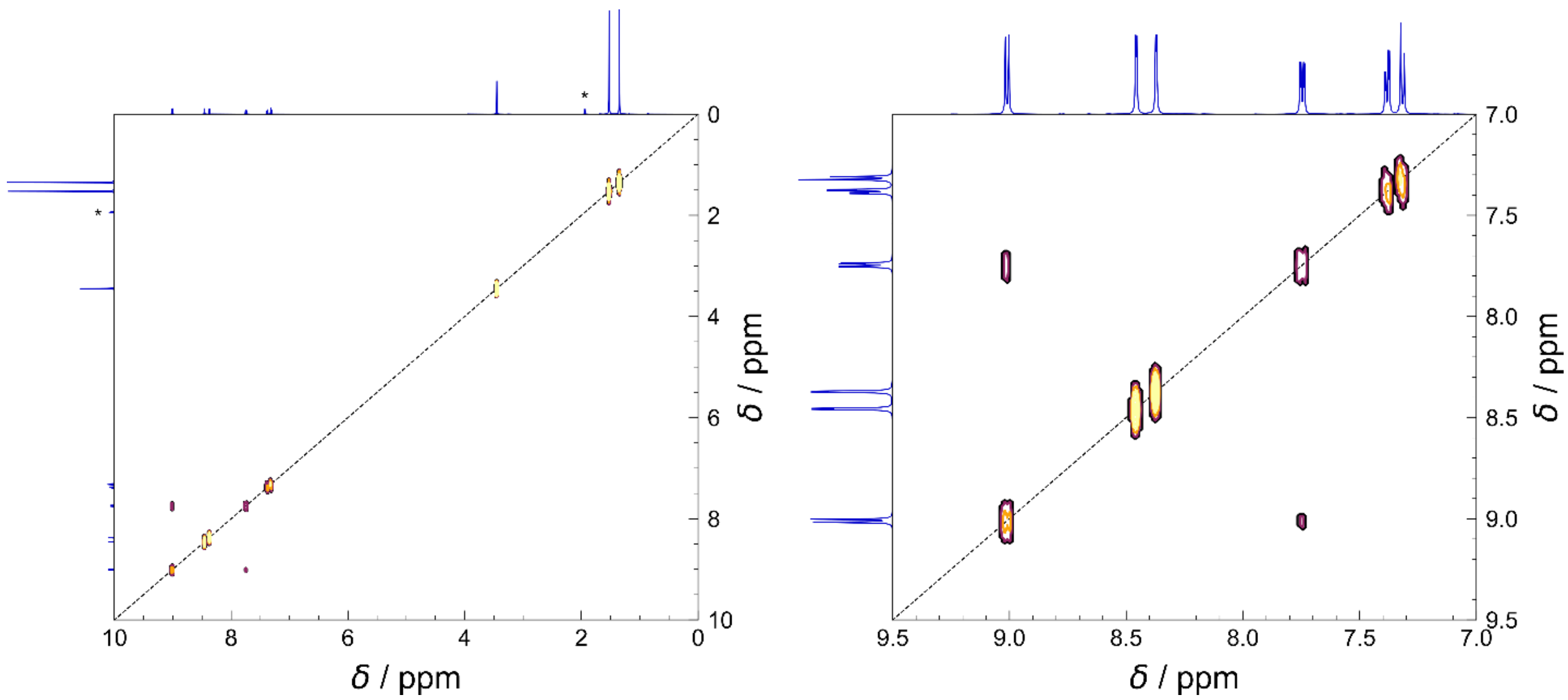

Figure S67. ^1H-^1H COSY spectrum of $[Ru(dtbbpy)_2(MeNC)_2](PF_6)_2$ recorded in CD_3CN. Left: full spectrum; Right: magnification of aromatic region. Asterisk denotes residual non-perdeuterated solvent.

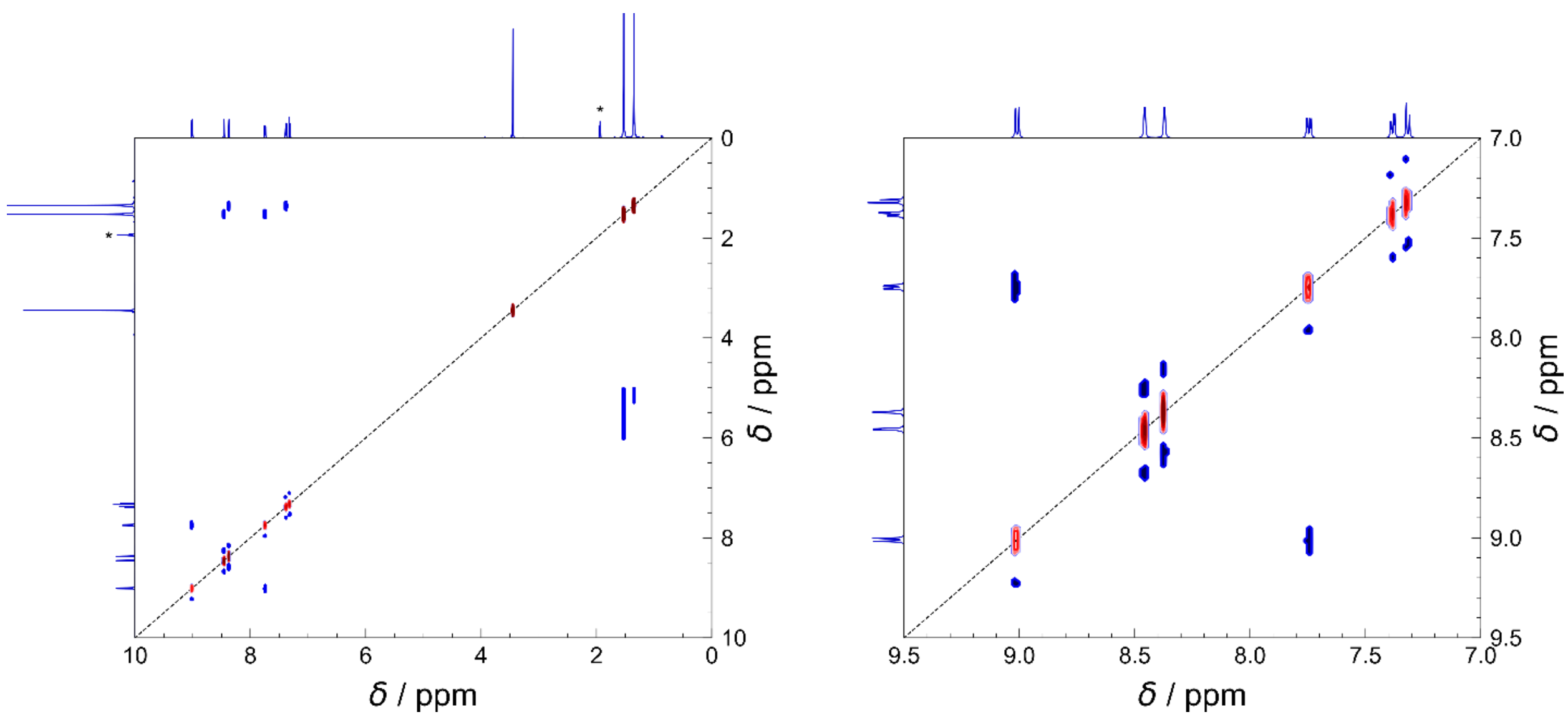

Figure S68. ^1H-^1H NOESY spectrum of $[Ru(dtbbpy)_2(MeNC)_2](PF_6)_2$ recorded in CD_3CN. Left: full spectrum; Right: magnification of aromatic region. Asterisk denotes residual non-perdeuterated solvent.

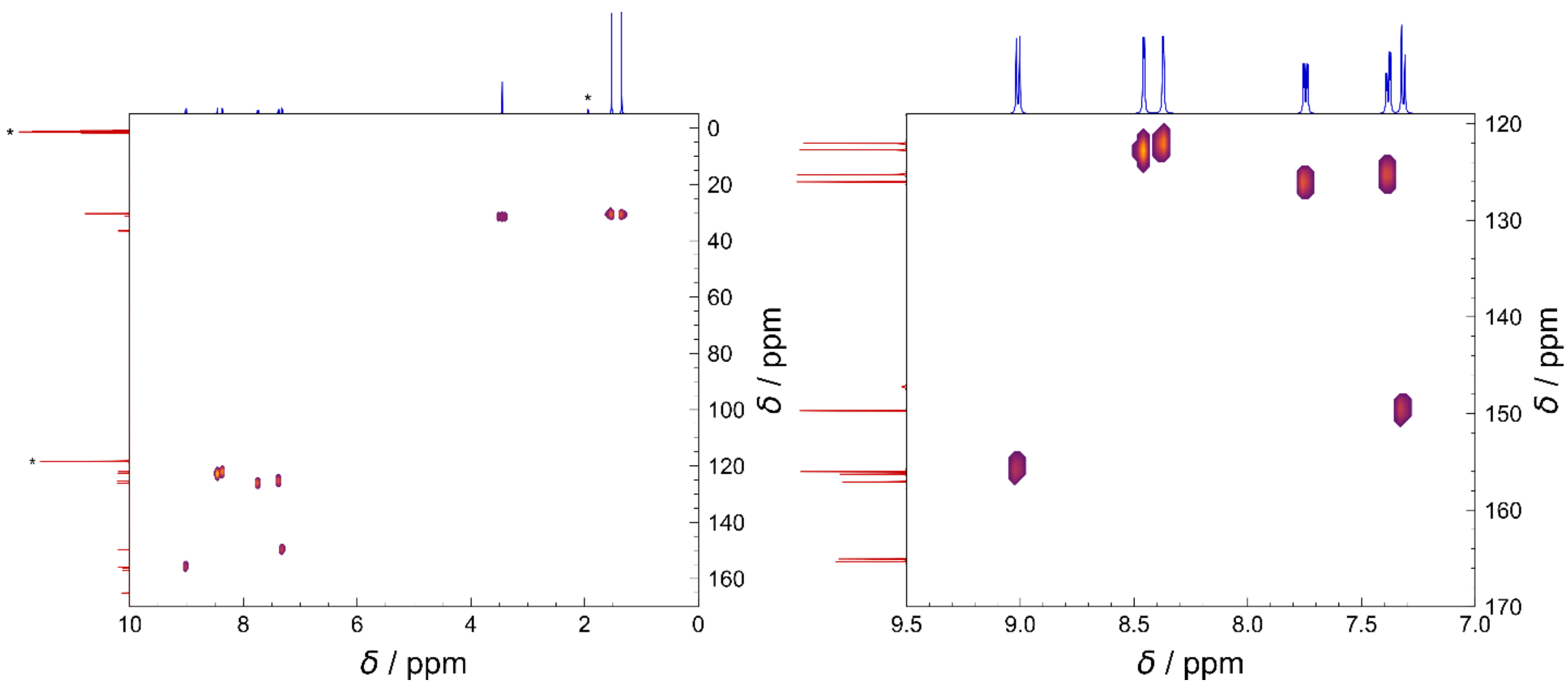

Figure S69. ^{1}H-^{13}C HSQC spectrum of $[Ru(dtbbpy)_2(MeNC)_2](PF_6)_2$ recorded in CD_3CN. Left: full spectrum; Right: magnification of aromatic region. Asterisk denotes residual non-perdeuterated solvent.

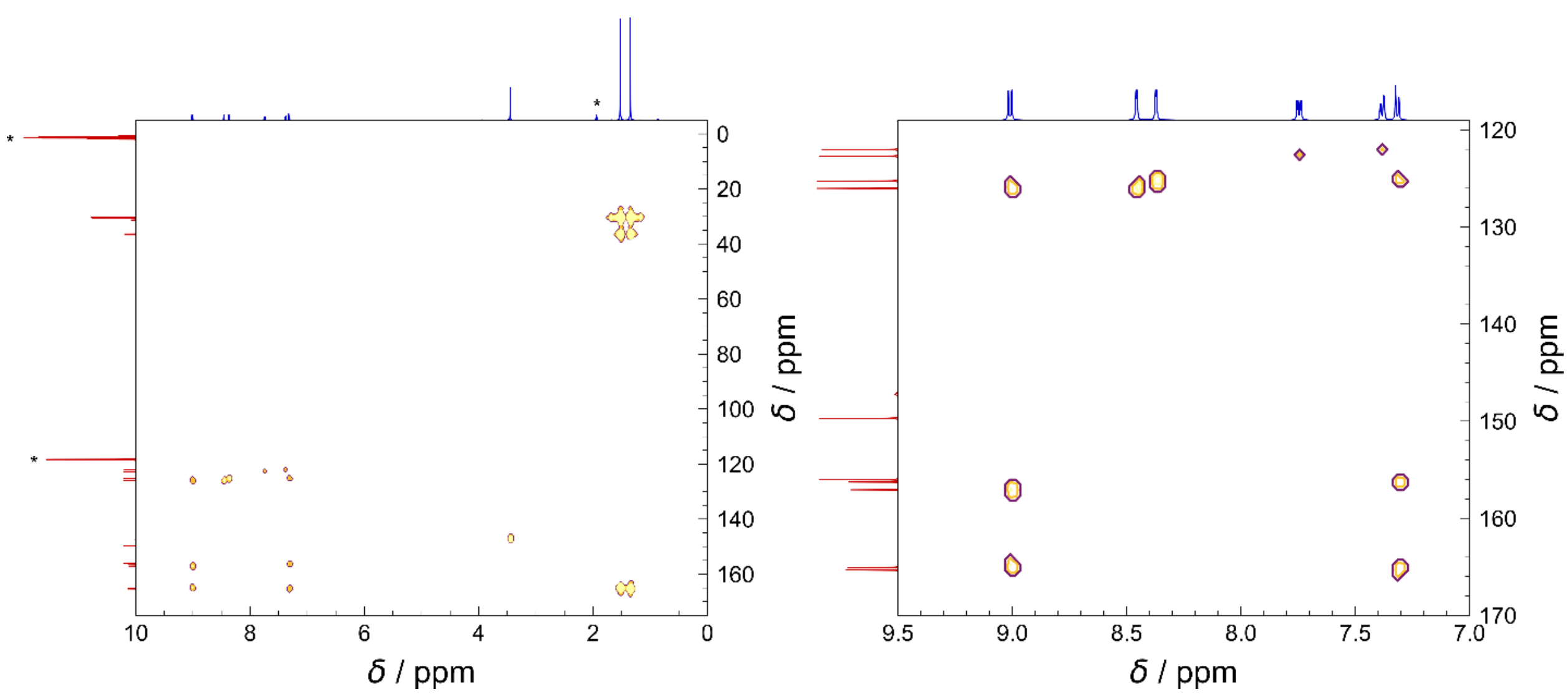

Figure S70. ^{1}H-^{13}C HMBC spectrum of $[Ru(dtbbpy)_2(MeNC)_2](PF_6)_2$ recorded in CD_3CN. Left: full spectrum; Right: magnification of aromatic region. Asterisk denotes residual non-perdeuterated solvent.

[Ru(dtbbpy)$_2$(C$_4$H$_{10}$N$_4$)$_2$](PF6)$_2$

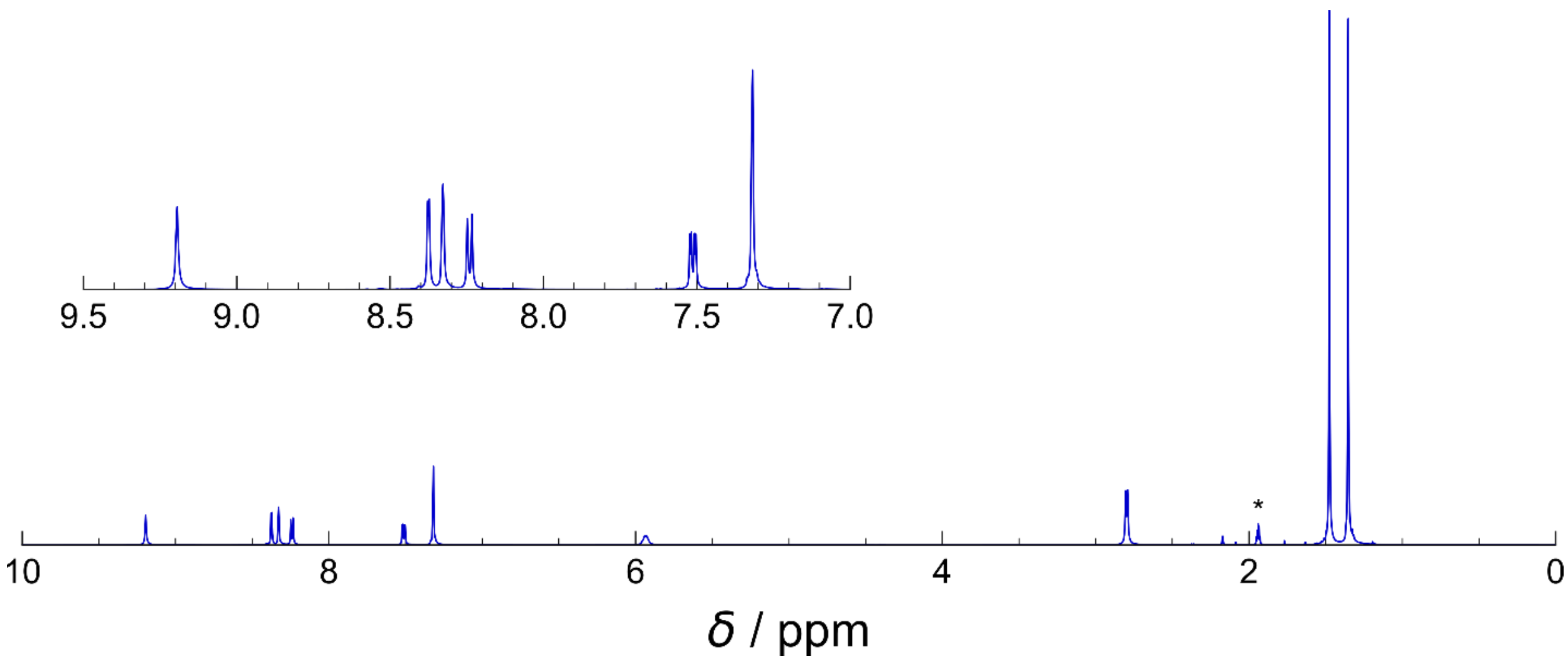

Figure S71. ^{1}H NMR spectrum of [Ru(dtbbpy)$_2$(C$_4$H$_{10}$N$_4$)](PF$_6$)$_2$ recorded in CD$_3$CN (400 MHz). Asterisk denotes residual non-perdeuterated solvent. Inset: Magnification of aromatic region.

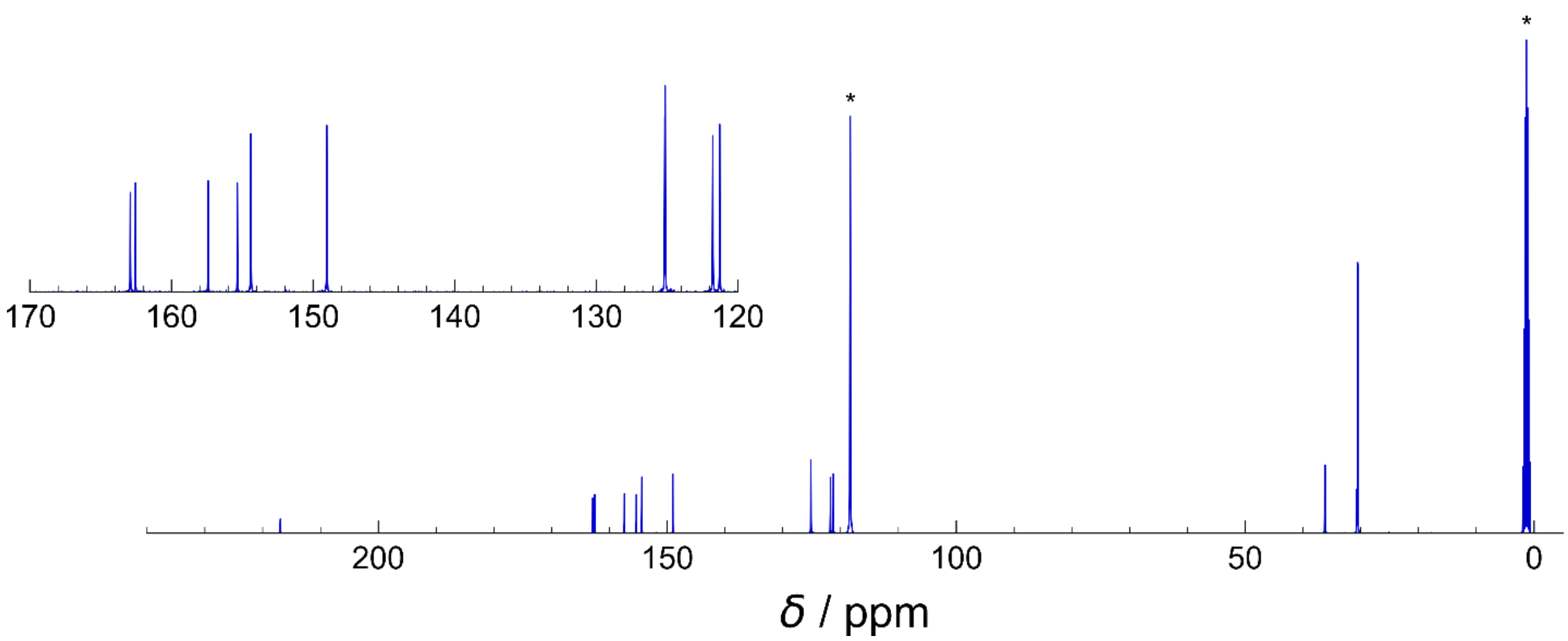

Figure S72. ^{13}C NMR spectrum of [Ru(dtbbpy)$_2$(C$_4$H$_{10}$N$_4$)](PF$_6$)$_2$ recorded in CD$_3$CN (101 MHz). Asterisk denotes residual non-perdeuterated solvent. Inset: Magnification of aromatic region.

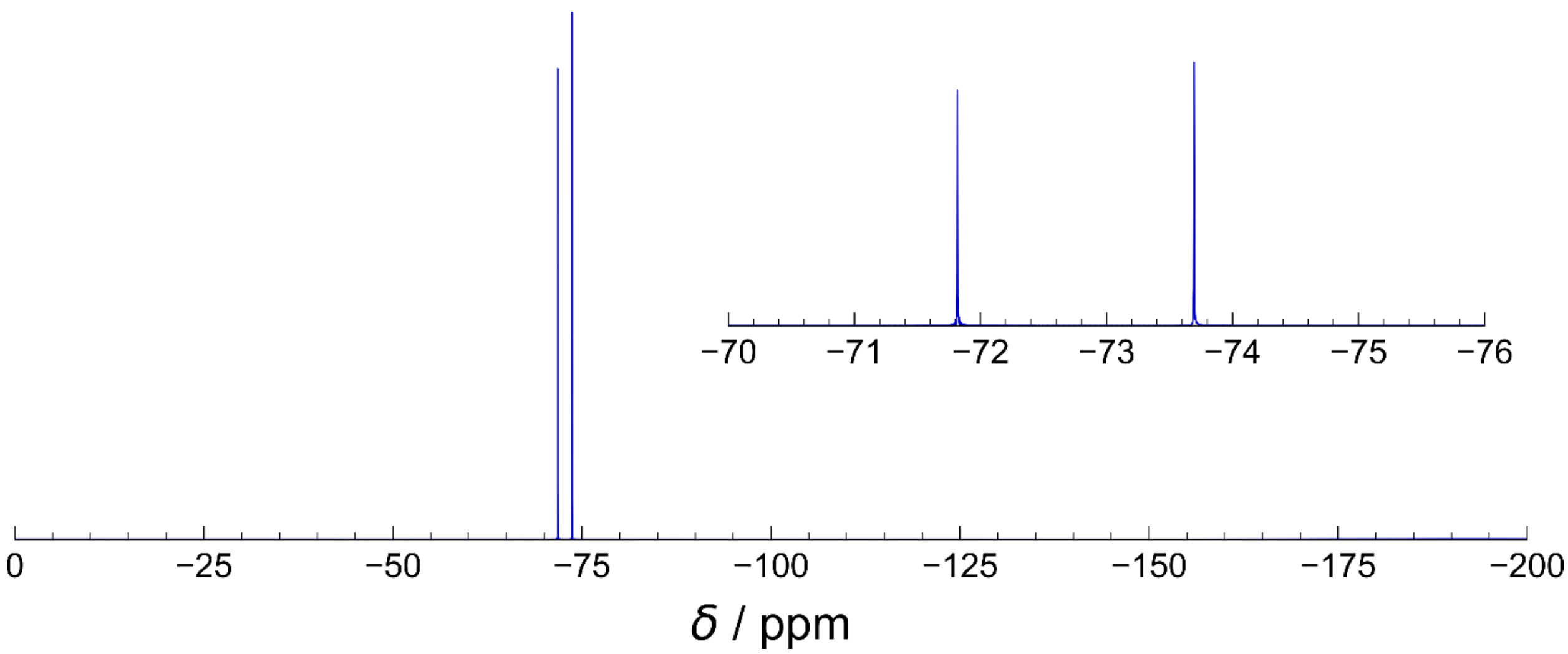

Figure S73. ^{19}F NMR spectrum of $[Ru(dtbbpy)_2(C_4H_{10}N_4)](PF_6)_2$ recorded in CD_3CN (376 MHz). Inset: Magnification of region of interest.

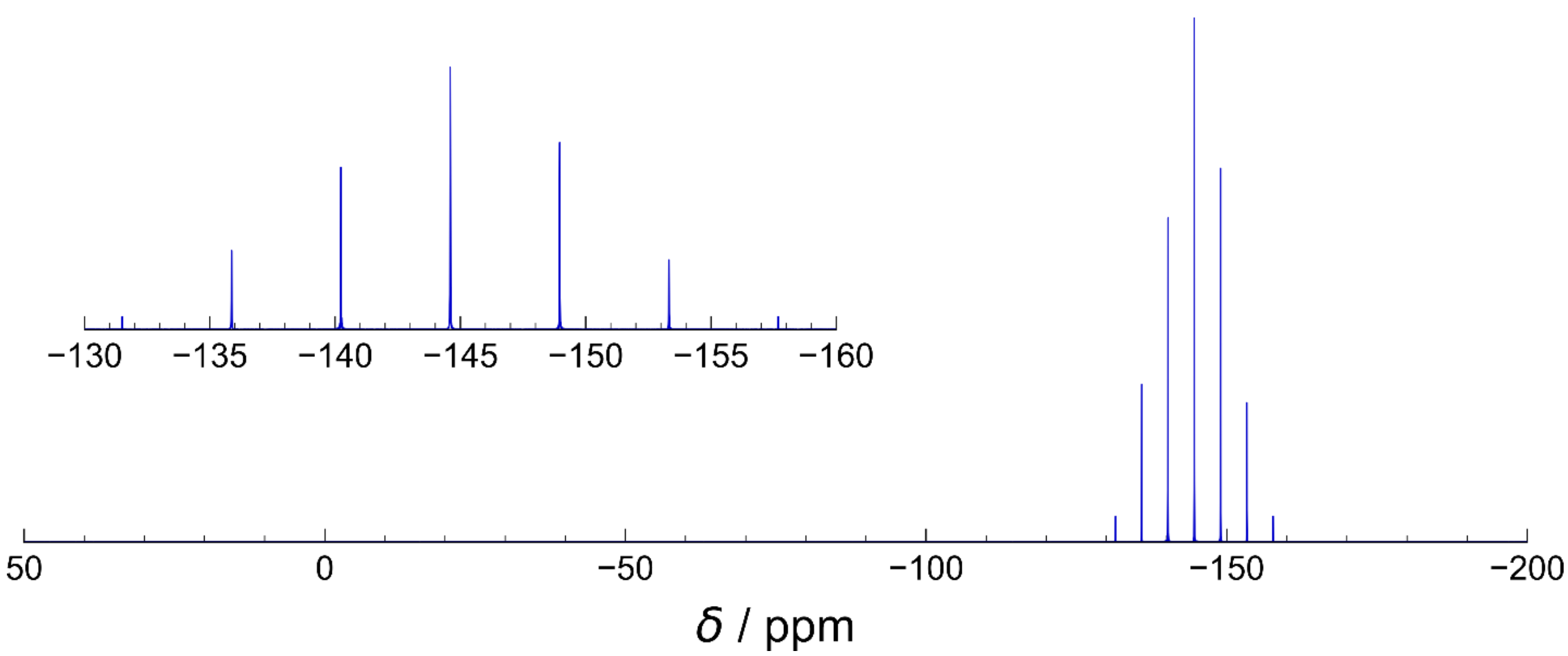

Figure S74. ^{31}P NMR spectrum of $[Ru(dtbbpy)_2(C_4H_{10}N_4)](PF_6)_2$ recorded in CD_3CN (162 MHz). Inset: Magnification of region of interest.

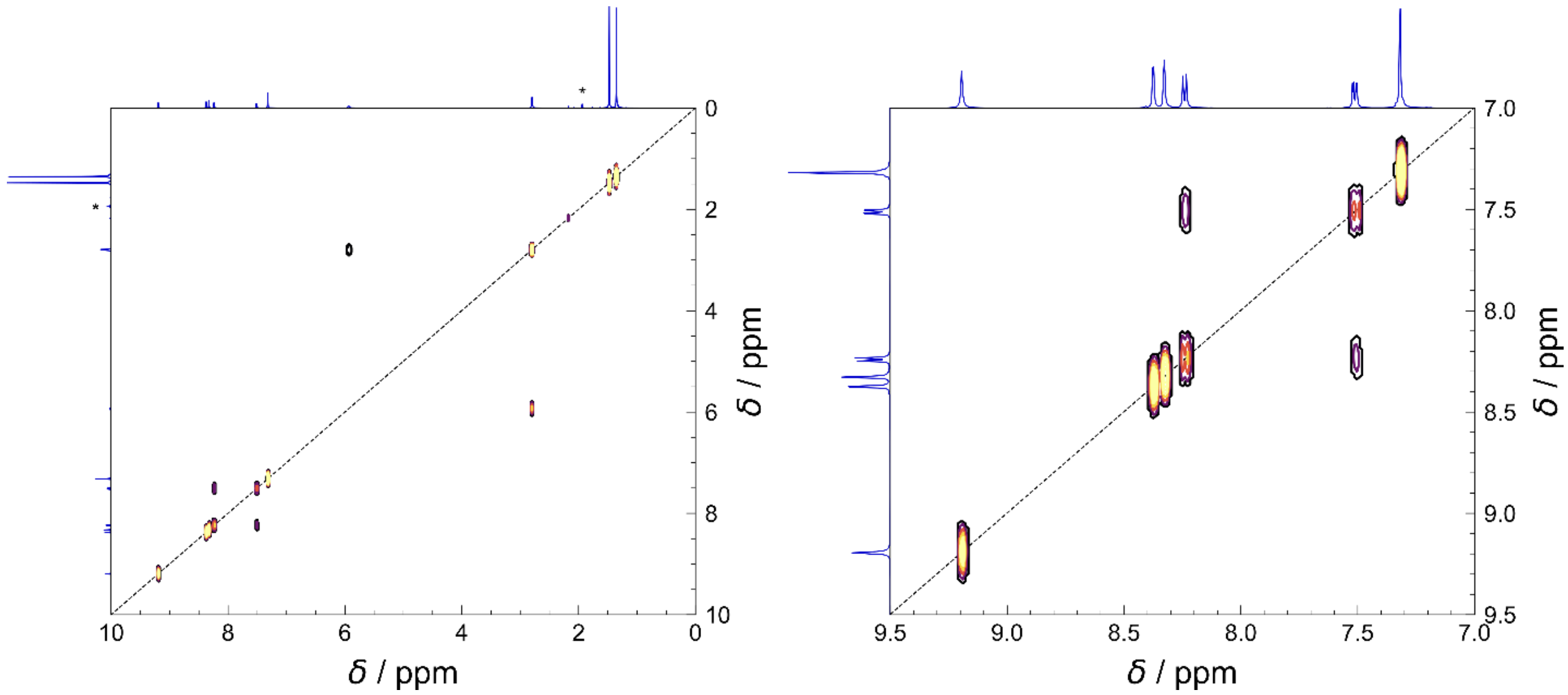

Figure S75. ^1H-^1H COSY spectrum of $[Ru(dtbbpy)_2(C_4H_{10}N_4)](PF_6)_2$ recorded in CD_3CN. Left: full spectrum; Right: magnification of aromatic region. Asterisk denotes residual non-perdeuterated solvent.

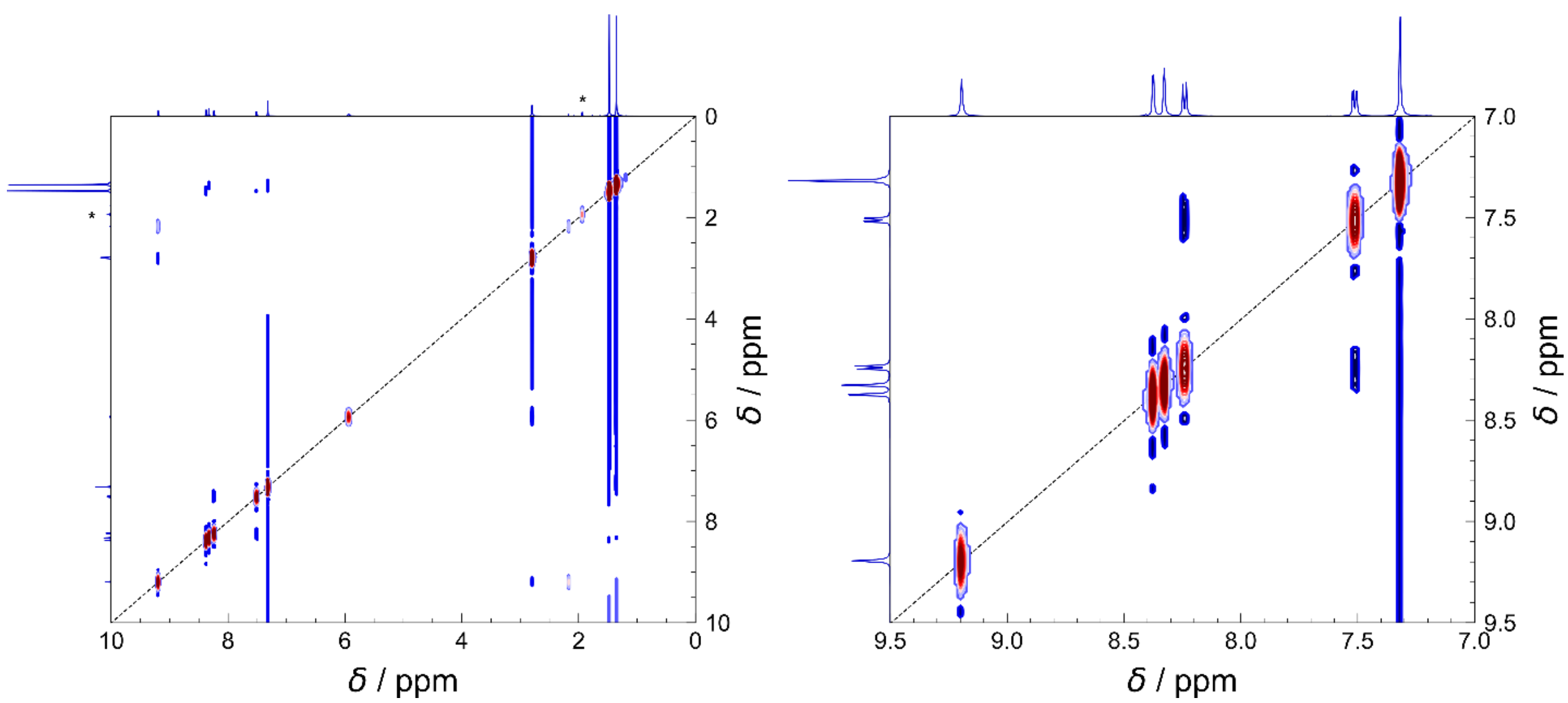

Figure S76. ^1H-^1H NOESY spectrum of $[Ru(dtbbpy)_2(C_4H_{10}N_4)](PF_6)_2$ recorded in CD_3CN. Left: full spectrum; Right: magnification of aromatic region. Asterisk denotes residual non-perdeuterated solvent.

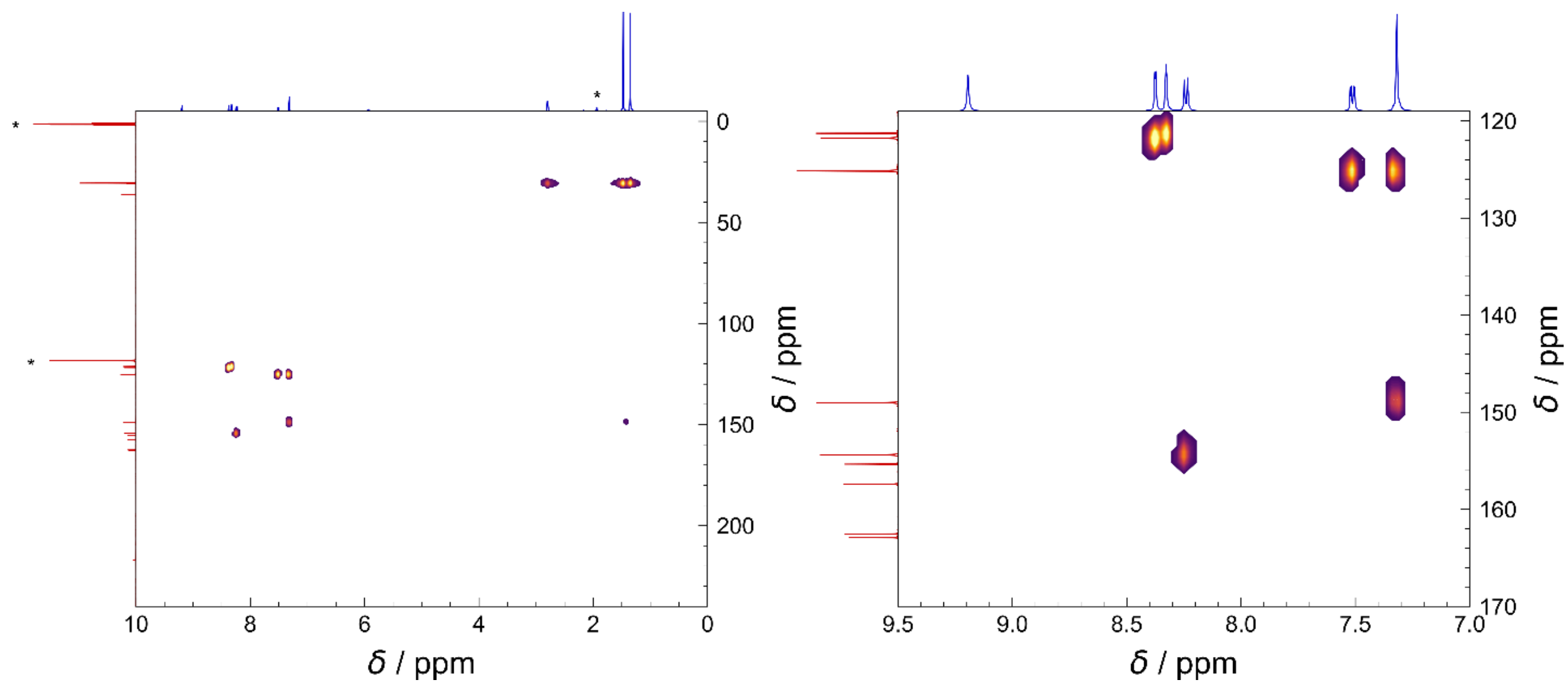

Figure S77. ^{1}H-^{13}C HSQC spectrum of [Ru(dtbbpy)$_2$($C_4H_{10}N_4$)](PF_6)$_2$ recorded in CD_3CN. Left: full spectrum; Right: magnification of aromatic region. Asterisk denotes residual non-perdeuterated solvent.

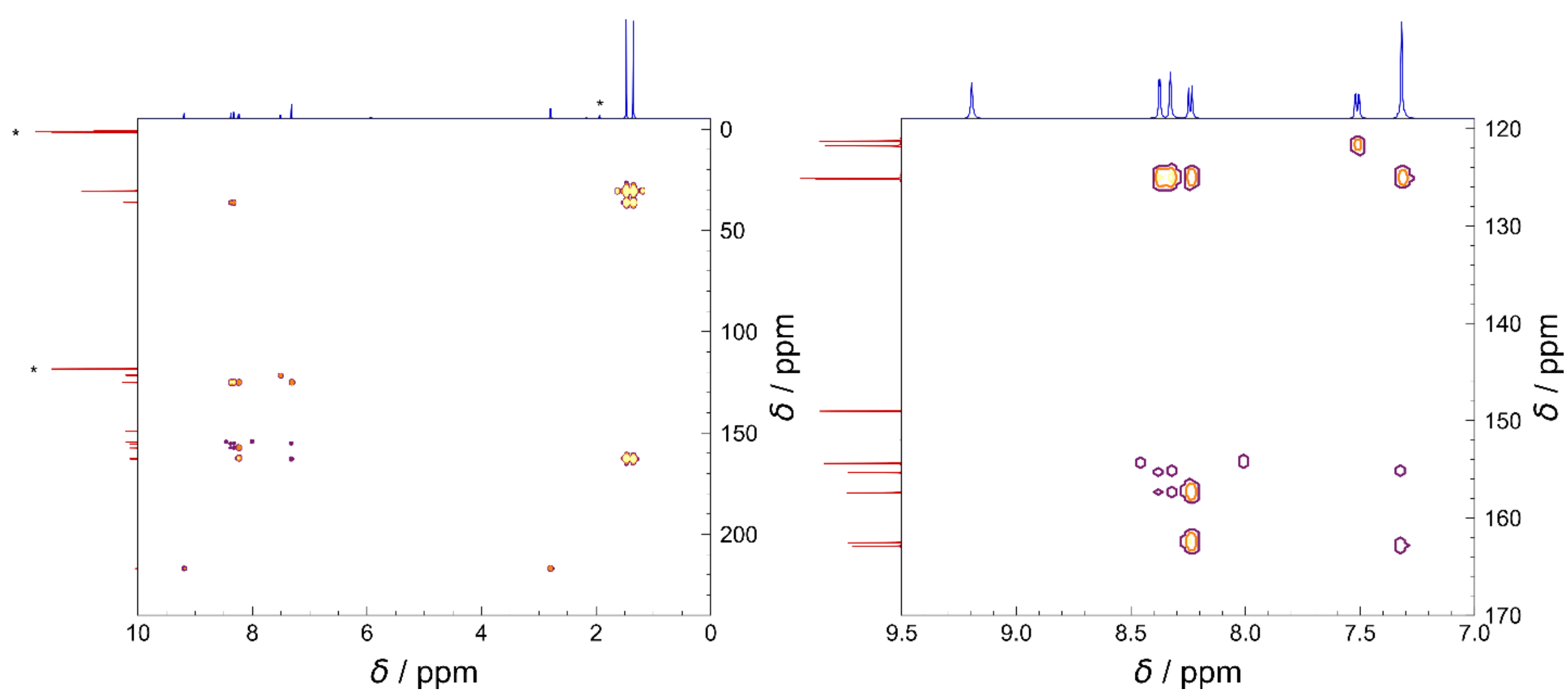

Figure S78. ^{1}H-^{13}C HMBC spectrum of [Ru(dtbbpy)$_2$($C_4H_{10}N_4$)](PF_6)$_2$ recorded in CD_3CN. Left: full spectrum; Right: magnification of aromatic region. Asterisk denotes residual non-perdeuterated solvent.

References

(1) Abe, T.; Shinozaki, K. Tuning of Luminescence Spectra of Neutral Ruthenium(II) Complexes by Crystal Waters. *Inorg. Chem.* **2005**, *44* (4), 849-851. DOI: 10.1021/ic049169u.
(2) Aihara, Y.; Sato, K.; Shinozaki, K. Optical Resolution, Determination of Absolute Configuration, and Photoracemization of *cis*-$RuL_2(CN)_2$ (L = 2,2′-Bipyridine and Its Analogues). *Inorg. Chem.* **2016**, *55* (17), 8387-8395. DOI: 10.1021/acs.inorgchem.6b00772.
(3) Gottlieb, H. E.; Kotlyar, V.; Nudelman, A. NMR chemical shifts of common laboratory solvents as trace impurities. *J. Org. Chem.* **1997**, *62* (21), 7512-7515.
(4) Fulmer, G. R.; Miller, A. J. M.; Sherden, N. H.; Gottlieb, H. E.; Nudelman, A.; Stoltz, B. M.; Bercaw, J. E.; Goldberg, K. I. NMR Chemical Shifts of Trace Impurities: Common Laboratory Solvents, Organics, and Gases in Deuterated Solvents Relevant to the Organometallic Chemist. *Organometallics* **2010**, *29* (9), 2176-2179. DOI: 10.1021/om100106e.
(5) Babij, N. R.; McCusker, E. O.; Whiteker, G. T.; Canturk, B.; Choy, N.; Creemer, L. C.; Amicis, C. V. D.; Hewlett, N. M.; Johnson, P. L.; Knobelsdorf, J. A.; Li, F.; Lorsbach, B. A.; et al. NMR Chemical Shifts of Trace Impurities: Industrially Preferred Solvents Used in Process and Green Chemistry. *Org. Process Res. Dev.* **2016**, *20* (3), 661-667. DOI: 10.1021/acs.oprd.5b00417.
(6) Dolomanov, O. V.; Bourhis, L. J.; Gildea, R. J.; Howard, J. A. K.; Puschmann, H. *OLEX2*: a complete structure solution, refinement and analysis program. *J. Appl. Cryst.* **2009**, *42* (2), 339-341. DOI: 10.1107/s0021889808042726.
(7) Sheldrick, G. M. *SHELXT* – Integrated space-group and crystal-structure determination. *Acta Crystallogr., Sect. A: Found. Adv.* **2015**, *71* (1), 3-8. DOI: 10.1107/s2053273314026370.
(8) Sheldrick, G. SHELXTL. *Acta Crystallogr., Sect. C: Struct. Chem* **2015**, *71*, 3-8.
(9) Walfort, B.; Gartmann, N.; Afshani, J.; Rosspeintner, A.; Hagemann, H. Effect of excitation wavelength (blue vs near UV) and dopant concentrations on afterglow and fast decay of persistent phosphor SrAl2O4:Eu2+,Dy3+. *J. Rare Earths* **2022**, *40* (7), 1022-1028. DOI: 10.1016/j.jre.2021.07.014.
(10) Nowak, S. H.; Armenta, R.; Schwartz, C. P.; Gallo, A.; Abraham, B.; Garcia-Esparza, A. T.; Biasin, E.; Prado, A.; Maciel, A.; Zhang, D.; Day, D.; Christensen, S.; et al. A versatile Johansson-type tender x-ray emission spectrometer. *Rev. Sci. Instrum.* **2020**, *91* (3), 033101. DOI: 10.1063/1.5121853.
(11) Gawelda, W.; Johnson, M.; De Groot, F. M. F.; Abela, R.; Bressler, C.; Chergui, M. Electronic and Molecular Structure of Photoexcited $[Ru^{II}(bpy)_3]^{2+}$ Probed by Picosecond X-ray Absorption Spectroscopy. *J. Am. Chem. Soc.* **2006**, *128* (15), 5001-5009. DOI: 10.1021/ja054932k.
(12) Virtanen, P.; Gommers, R.; Oliphant, T. E.; Haberland, M.; Reddy, T.; Cournapeau, D.; Burovski, E.; Peterson, P.; Weckesser, W.; Bright, J.; Van Der Walt, S. J.; Brett, M.; et al. SciPy 1.0: fundamental algorithms for scientific computing in Python. *Nat. Methods* **2020**, *17* (3), 261-272. DOI: 10.1038/s41592-019-0686-2.
(13) Harris, C. R.; Millman, K. J.; Van Der Walt, S. J.; Gommers, R.; Virtanen, P.; Cournapeau, D.; Wieser, E.; Taylor, J.; Berg, S.; Smith, N. J.; Kern, R.; Picus, M.; et al. Array programming with NumPy. *Nature* **2020**, *585* (7825), 357-362. DOI: 10.1038/s41586-020-2649-2.
(14) Neese, F. Software update: The ORCA program system—Version 5.0. *WIREs Comp. Mol. Sci.* **2022**, *12* (5). DOI: 10.1002/wcms.1606.
(15) Becke, A. D. A new mixing of Hartree–Fock and local density-functional theories. *J. Chem. Phys.* **1993**, *98* (2), 1372-1377. DOI: 10.1063/1.464304.
(16) Becke, A. D. Density-functional thermochemistry. III. The role of exact exchange. *J. Chem. Phys.* **1993**, *98* (7), 5648-5652. DOI: 10.1063/1.464913.
(17) Becke, A. D. Density functional calculations of molecular bond energies. *J. Chem. Phys.* **1986**, *84* (8), 4524-4529. DOI: 10.1063/1.450025.
(18) Lee, C.; Yang, W.; Parr, R. G. Development of the Colle-Salvetti correlation-energy formula into a functional of the electron density. *Phys. Rev. B* **1988**, *37* (2), 785-789. DOI: 10.1103/physrevb.37.785.

(19) Neese, F. An improvement of the resolution of the identity approximation for the formation of the Coulomb matrix. *J. Comp. Chem.* **2003**, *24* (14), 1740-1747. DOI: 10.1002/jcc.10318.
(20) Neese, F.; Wennmohs, F.; Hansen, A.; Becker, U. Efficient, approximate and parallel Hartree–Fock and hybrid DFT calculations. A 'chain-of-spheres' algorithm for the Hartree–Fock exchange. *Chem. Phys.* **2009**, *356* (1-3), 98-109. DOI: 10.1016/j.chemphys.2008.10.036.
(21) Grimme, S.; Antony, J.; Ehrlich, S.; Krieg, H. A consistent and accurate *ab initio* parametrization of density functional dispersion correction (DFT-D) for the 94 elements H-Pu. *J. Chem. Phys.* **2010**, *132* (15), 154104. DOI: 10.1063/1.3382344.
(22) Grimme, S.; Ehrlich, S.; Goerigk, L. Effect of the damping function in dispersion corrected density functional theory. *J. Comp. Chem.* **2011**, *32* (7), 1456-1465. DOI: https://doi.org/10.1002/jcc.21759 (acccessed 2026/06/10).
(23) Schäfer, A.; Horn, H.; Ahlrichs, R. Fully optimized contracted Gaussian basis sets for atoms Li to Kr. *J. Chem. Phys.* **1992**, *97* (4), 2571-2577. DOI: 10.1063/1.463096 (acccessed 2/12/2025).
(24) Schäfer, A.; Huber, C.; Ahlrichs, R. Fully optimized contracted Gaussian basis sets of triple zeta valence quality for atoms Li to Kr. *J. Chem. Phys.* **1994**, *100* (8), 5829-5835. DOI: 10.1063/1.467146 (acccessed 2/12/2025).
(25) Eichkorn, K.; Treutler, O.; Öhm, H.; Häser, M.; Ahlrichs, R. Auxiliary basis sets to approximate Coulomb potentials (Chem. Phys. Letters 240 (1995) 283-290). *Chem. Phys. Lett.* **1995**, *242* (6), 652-660. DOI: 10.1016/0009-2614(95)00838-u.
(26) Eichkorn, K.; Weigend, F.; Treutler, O.; Ahlrichs, R. Auxiliary basis sets for main row atoms and transition metals and their use to approximate Coulomb potentials. *Theor. Chem. Acc.: Theory, Computation, and Modeling (Theor. Chim. Acta)* **1997**, *97* (1-4), 119-124. DOI: 10.1007/s002140050244.
(27) Weigend, F. Accurate Coulomb-fitting basis sets for H to Rn. *Phys. Chem. Chem. Phys.* **2006**, *8* (9), 1057. DOI: 10.1039/b515623h.
(28) Barone, V.; Cossi, M. Quantum Calculation of Molecular Energies and Energy Gradients in Solution by a Conductor Solvent Model. *J. Phys. Chem. A* **1998**, *102* (11), 1995-2001. DOI: 10.1021/jp9716997.
(29) Marenich, A. V.; Cramer, C. J.; Truhlar, D. G. Universal Solvation Model Based on Solute Electron Density and on a Continuum Model of the Solvent Defined by the Bulk Dielectric Constant and Atomic Surface Tensions. *J. Phys. Chem. B* **2009**, *113* (18), 6378-6396. DOI: 10.1021/jp810292n.
(30) Allouche, A. R. Gabedit—A graphical user interface for computational chemistry softwares. *J. Comp. Chem.* **2011**, *32* (1), 174-182. DOI: 10.1002/jcc.21600.
(31) Ishida, H.; Tobita, S.; Hasegawa, Y.; Katoh, R.; Nozaki, K. Recent advances in instrumentation for absolute emission quantum yield measurements. *Coord. Chem. Rev.* **2010**, *254* (21-22), 2449-2458. DOI: 10.1016/j.ccr.2010.04.006.
(32) Suzuki, K.; Kobayashi, A.; Kaneko, S.; Takehira, K.; Yoshihara, T.; Ishida, H.; Shiina, Y.; Oishi, S.; Tobita, S. Reevaluation of absolute luminescence quantum yields of standard solutions using a spectrometer with an integrating sphere and a back-thinned CCD detector. *Phys. Chem. Chem. Phys.* **2009**, *11* (42), 9850. DOI: 10.1039/b912178a.
(33) Ito, A.; Meyer, T. J. The Golden Rule. Application for fun and profit in electron transfer, energy transfer, and excited-state decay. *Phys. Chem. Chem. Phys.* **2012**, *14* (40), 13731. DOI: 10.1039/c2cp41658a.
(34) Caspar, J. V.; Westmoreland, T. D.; Allen, G. H.; Bradley, P. G.; Meyer, T. J.; Woodruff, W. H. Molecular and electronic structure in the metal-to-ligand charge-transfer excited states of d6 transition-metal complexes in solution. *J. Am. Chem. Soc.* **1984**, *106* (12), 3492-3500. DOI: 10.1021/ja00324a017.
(35) Yersin, H.; Otto, H.; Zink, J. I.; Gliemann, G. Franck-Condon analysis of transition-metal complexes. *J. Am. Chem. Soc.* **1980**, *102* (3), 951-955.
(36) Thompson, D. W.; Ito, A.; Meyer, T. J. [Ru(bpy)3]2+* and other remarkable metal-to-ligand charge transfer (MLCT) excited states. *Pure Appl. Chem.* **2013**, *85* (7), 1257-1305. DOI: 10.1351/pac-con-13-03-04.
(37) Kunnus, K.; Li, L.; Titus, C. J.; Lee, S. J.; Reinhard, M. E.; Koroidov, S.; Kjær, K. S.; Hong, K.; Ledbetter, K.; Doriese, W. B.; O'Neil, G. C.; Swetz, D. S.; et al. Chemical control of competing electron

transfer pathways in iron tetracyano-polypyridyl photosensitizers. *Chem. Sci.* **2020**, *11* (17), 4360-4373. DOI: 10.1039/c9sc06272f.
(38) Dongare, P.; Myron, B. D. B.; Wang, L.; Thompson, D. W.; Meyer, T. J. $[Ru(bpy)_3]^{2+*}$ revisited. Is it localized or delocalized? How does it decay? *Coord. Chem. Rev.* **2017**, *345*, 86-107. DOI: 10.1016/j.ccr.2017.03.009.
(39) Caspar, J. V.; Meyer, T. J. Photochemistry of MLCT excited states. Effect of nonchromophoric ligand variations on photophysical properties in the series cis-$Ru(bpy)_2L_2^{2+}$. *Inorg. Chem.* **1983**, *22* (17), 2444-2453. DOI: 10.1021/ic00159a021.
(40) Kober, E. M.; Caspar, J. V.; Lumpkin, R. S.; Meyer, T. J. Application of the energy gap law to excited-state decay of osmium (II)-polypyridine complexes: calculation of relative nonradiative decay rates from emission spectral profiles. *J. Phys. Chem.* **1986**, *90* (16), 3722-3734.
(41) Barqawi, K. R.; Murtaza, Z.; Meyer, T. J. Calculation of relative nonradiative decay rate constants from emission spectral profiles: polypyridyl complexes of ruthenium (II). *J. Phys. Chem.* **1991**, *95* (1), 47-50.
(42) Caspar, J. V.; Kober, E. M.; Sullivan, B. P.; Meyer, T. J. Application of the energy gap law to the decay of charge-transfer excited states. *J. Am. Chem. Soc.* **1982**, *104* (2), 630-632.
(43) Caspar, J. V.; Meyer, T. J. Application of the energy gap law to nonradiative, excited-state decay. *J. Phys Chem.* **1983**, *87* (6), 952-957.
(44) Van Kuiken, B. E.; Huse, N.; Cho, H.; Strader, M. L.; Lynch, M. S.; Schoenlein, R. W.; Khalil, M. Probing the Electronic Structure of a Photoexcited Solar Cell Dye with Transient X-ray Absorption Spectroscopy. *J. Phys. Chem. Lett.* **2012**, *3* (12), 1695-1700. DOI: 10.1021/jz300671e.
(45) Lees, A. J. A Photochemical Procedure for Determining Reaction Quantum Efficiencies in Systems with Multicomponent Inner Filter Absorbances. *Anal. Chem.* **1996**, *68* (1), 226-229. DOI: 10.1021/ac9507653.
(46) Marcolongo, J. P.; Schmidt, J.; Levin, N.; Slep, L. D. A chemometric approach for determining the reaction quantum yields in consecutive photochemical processes. *Phys. Chem. Chem. Phys.* **2017**, *19* (32), 21373-21381. DOI: 10.1039/c7cp03619a.
(47) Schmid, L.; Kerzig, C.; Prescimone, A.; Wenger, O. S. Photostable Ruthenium(II) Isocyanoborato Luminophores and Their Use in Energy Transfer and Photoredox Catalysis. *JACS Au* **2021**, *1* (6), 819-832. DOI: 10.1021/jacsau.1c00137.
(48) Soupart, A.; Alary, F.; Heully, J.-L.; Elliott, P. I. P.; Dixon, I. M. Exploration of Uncharted ^{3}PES Territory for $[Ru(bpy)_3]^{2+}$: A New ^{3}MC Minimum Prone to Ligand Loss Photochemistry. *Inorg. Chem.* **2018**, *57* (6), 3192-3196. DOI: 10.1021/acs.inorgchem.7b03229.
(49) Eastham, K.; Scattergood, P. A.; Chu, D.; Boota, R. Z.; Soupart, A.; Alary, F.; Dixon, I. M.; Rice, C. R.; Hardman, S. J. O.; Elliott, P. I. P. Not All ^{3}MC States Are the Same: The Role of ^{3}MCcis States in the Photochemical N∧N Ligand Release from $[Ru(bpy)_2(N∧N)]^{2+}$ Complexes. *Inorg. Chem.* **2022**, *61* (49), 19907-19924. DOI: 10.1021/acs.inorgchem.2c03146.
(50) Soupart, A.; Alary, F.; Heully, J.-L.; Elliott, P. I. P.; Dixon, I. M. Theoretical Study of the Full Photosolvolysis Mechanism of $[Ru(bpy)_3]^{2+}$: Providing a General Mechanistic Roadmap for the Photochemistry of $[Ru(N^\wedge N)_3]^{2+}$-Type Complexes toward Both Cis and Trans Photoproducts. *Inorg. Chem.* **2020**, *59* (20), 14679-14695. DOI: 10.1021/acs.inorgchem.0c01843.
(51) Hernández-Castillo, D.; Nau, R. E. P.; Schmid, M. A.; Tschierlei, S.; Rau, S.; González, L. Multiple Triplet Metal-Centered Jahn-Teller Isomers Determine Temperature-Dependent Luminescence Lifetimes in $[Ru(bpy)_3]^{2+}$. *Angewandte Chemie International Edition* **2023**, *62* (48). DOI: 10.1002/anie.202308803.
